# Isolation and Characterization of *Pseudomonas chlororaphis* Strain ST9; Rhizomicrobiota and in Planta Studies

**DOI:** 10.3390/plants10071466

**Published:** 2021-07-17

**Authors:** Iris Bertani, Elisa Zampieri, Cristina Bez, Andrea Volante, Vittorio Venturi, Stefano Monaco

**Affiliations:** 1 International Centre for Genetic Engineering and Biotechnology, 34149 Trieste, Italy; bertani@icgeb.org (I.B.); bez@icgeb.org (C.B.); 2 Council for Agricultural Research and Economics-Research Centre for Cereal and Industrial Crops, s.s. 11 to Torino, km 2.5, 13100 Vercelli, Italy; elisa.zampieri@ipsp.cnr.it (E.Z.); andrea.volante@crea.gov.it (A.V.); 3 Institute for Sustainable Plant Protection, National Research Council, Strada delle Cacce 73, 10135 Turin, Italy; 4 Council for Agricultural Research and Economics-Research Centre for Vegetable and Ornamental Crops, Corso Inglesi 508, 18038 Sanremo, Italy

**Keywords:** rice, plant growth promoting bacteria, qPCR, microbiota, inoculum persistence, genome analysis

## Abstract

The development of biotechnologies based on beneficial microorganisms for improving soil fertility and crop yields could help to address many current agriculture challenges, such as food security, climate change, pest control, soil depletion while decreasing the use of chemical fertilizers and pesticides. Plant growth-promoting (PGP) microbes can be used as probiotics in order to increase plant tolerance/resistance to abiotic/biotic stresses and in this context strains belonging to the *Pseudomonas chlororaphis* group have shown to have potential as PGP candidates. In this study a new *P. chlororaphis* isolate is reported and tested for (i) in vitro PGP features, (ii) whole-genome sequence analysis, and (iii) its effects on the rhizosphere microbiota composition, plant growth, and different plant genes expression levels in greenhouse experiments. Results showed that *P. chlororaphis* ST9 is an efficient rice root colonizer which integrates into the plant resident-microbiota and affects the expression of several plant genes. The potential use of this *P. chlororaphis* strain as a plant probiotic is discussed.

## 1. Introduction

The agriculture of the 21st century has several challenges to face. Among them are the increase in population and the growing demand for food in the context of climate change, soil depletion, competition between different land uses, and the need to reduce chemical fertilizers and pesticides. The use of beneficial microorganisms in crop production is an appealing solution as they can have beneficial effects on soil and plant by improving soil fertility, plant yields and reducing the use of agrochemicals [1]. The development of next-generation sequencing methodologies has led to numerous studies on plant microbiomes documenting that plants are colonized and live in association with a large number of microorganisms. Many of these, indicated as PGP (plant growth-promoting) microbes, play important roles in plant health and resistance to biotic and abiotic stresses [2,3,4,5]. PGP microbes allow the reduction of fertilizers requirements, improving crop nutrient use efficiency (nitrogen, phosphate, etc.), and protecting the host plant by pathogens invasion through niche exclusion mechanisms and antibacterial/antifungal compound production [6,7]. PGP microorganisms can also elicit transcriptional changes in hormone-, defense- and cell wall- related genes [8], increase root length [9], and activate auxin-response genes that reinforce plant growth [10]. In the last decades, the interest in developing PGP probiotic microorganisms has increased intending to reduce chemical fertilization [11] and pesticide use, both in conventional and organic farming, to offer healthier food and improving the sustainability of crop production [12]. One class of microorganisms that has been studied for many years for their application is known as plant growth-promoting rhizobacteria (PGPR) [13]. Among the group of PGPR, strains belonging to the *Pseudomonas chlororaphis* species have been found in association with a wide range of plants, both mono- and dicotyledonous, and both wild and cultivated [14]. *P. chlororaphis* is currently classified into four subspecies, namely *chlororaphis*, *aureofaciens*, *aurantiaca*, and *piscium* [15,16]. Several strains of *P. chlororaphis* have shown potential for application as plant probiotics [17,18,19] due to their rhizosphere colonization abilities and plant-associated beneficial phenotypes such as chemotaxis and motility [20], biofilm formation [21], P solubilization [22], ACC deaminase [23], IAA production [24,25] and biocontrol. *P. chlororaphis* strains produce different antifungal compounds such as Prn (pyrrolnitrin), PCN (phenazine-1-carboxamide), PCA (phenazine-1-carboxylic acid), 2-OH-PHZ (2-hydroxyphenazine), HPR (2-hexyl-5-propyl-alkylresorcinol) and HCN (hydrogen cyanide). These molecules inhibit the growth of various phytopathogens belonging to the *Fusarium* group [26,27] and different species of *Colletotrichum, Phytophthora*, *Pythium*, *Sclerotinia, Magnaporthe oryzae* [28] and *Rhizoctonia* [29], protecting plants such as maize [30], tomato [31]. Besides, good formulation protocols have been developed and products containing *P. chlororaphis* strains have been produced and commercialized [32]. In this work, we report the isolation and characterization of *P. chlororaphis* strain ST9. This strain was able to colonize and persist in rice roots for the entire rice vegetative cycle and studies are presented of its effect on the root microbiota, on plant growth and on the expression of several plant genes. The potential use of this *P. chlororaphis* strain as a plant probiotic for rice cultivation is discussed.

## 2. Results

### 2.1. Identification and Characterization of P. chlororaphis ST9

A culture collection of bacterial isolates was generated from two soil samples as described in the Materials and Methods section. It was of interest to then focus our studies on a new isolate of *P. chlororaphis* called strain ST9, since this species is used in microbial inoculants for agriculture and is known to have several plant beneficial properties. The genome sequence of *P. chlororaphis* ST9 was determined and its chromosome was 6.7 Mb long with a GC content of 63%; according to the RASTtk annotation scheme it contains 6185 predicted protein-coding sequences (CDSs) and 83 RNAs. Phylogenetic analyses based on MSLA using 6 loci (16S rRNA, *recA*, *gyrB*, *rpoD*, *carA* and *atpD;* [33]) and 16 sequences of *P. chlororaphis* isolates revealed that strain ST9 belongs to the *Pseudomonas chlororaphis* subsp. *aurantiaca* group (Supplementary Figure S1). Similar to other *P. chlororaphis* strains, its genome possesses several loci encoding for anti-bacterial/anti-fungal compounds including operons for the biosynthesis of phenazine, two R-tailocins bacteriocines and pyrrolnitrin. Other loci potentially involved in microbial pathogen antagonism, cell-cell signal interferences, niche colonization, plant protection and plant-beneficial traits were identified and reported in Table 1. In summary, *P. chlororaphis* ST9 possesses many features that can potentially make this strain a plant probiotic and biocontrol agent.

**Figure 1:**
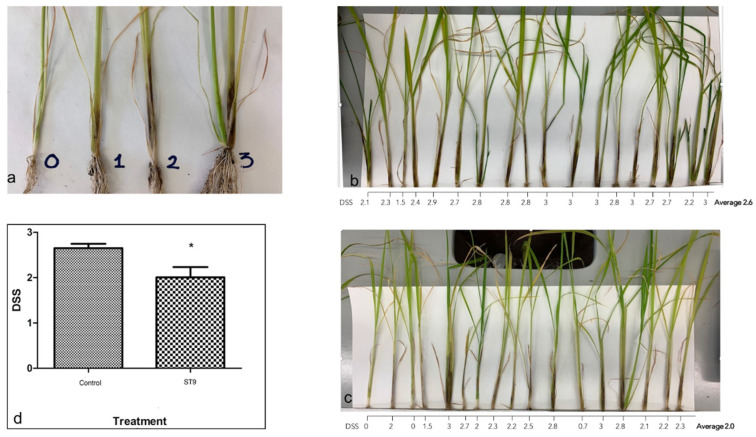
Biocontrol activity of *P. chlororaphis* ST9 against *Dickeya zeae*. (**a**) Disease Severity Score Scale; (**b**,**c**) control and ST9 treated plants respectively, 7 days after *D. zeae* infection: the average of the DSS (Disease Severity Score) assigned to each plant by 7 different persons is reported; (**d**) graph reporting the average of the DSS assigned to the control and ST9 treated plants: the difference proved to be significant (Mann–Whitney *t*-test: * *p*-value < 0.05).

In order to assess the possible PGP potential of *P. chlororaphis* ST9, several in vitro and in vivo phenotype tests, summarized in Table 2, were performed. Among the performed tests, the ability of ST9 to inhibit the growth of a wide variety of plant pathogens, including *Dickeya zeae*, *Pseudomonas fuscovaginae*, *Magnaporthe oryzae*, *Aspergillus nidulans* and *Fusarium graminearum* was observed in vitro (Supplementary Figure S2). An in planta biocontrol assay was also performed: ST9 treated plants displayed a slight but significant decrease of the severity of the disease as reported in Figure 1.

Other PGP-related in vitro phenotypes such as lipolytic and proteolytic activities and motility were observed. In summary, this isolate possesses several phenotypic abilities which can be of importance in plant colonization and PGP.

### 2.2. P. chlororaphis ST9 Root Colonization Ability, Persistence and Effect on the Rhizosphere Microbiota

**Figure 2:**
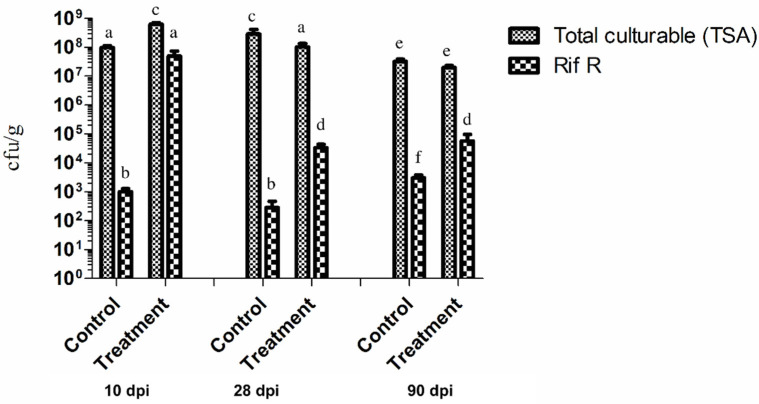
Rice root colonization by *P. chlororaphis* ST9 was evaluated at three time points: 10, 28 and 90 dpi (days post inoculation). The average of the cfu/g of fresh weight root of 10 plants for each treatment and time point is presented. Each sample was plated on TSA to count all cultivable bacteria, and on TSArif to count just the rifampicin resistant CFUs, mainly *P. chlororaphis* ST9. Statistical analysis: a–f letters indicate statistical differences: same letter means no significant difference (*t*-test, non-parametric, Mann–Whitney, *p*-value < 0.05).

The ability of *P. chlororaphis* ST9 to colonize and persist in the rhizosphere of rice plants was evaluated. Rice seeds were inoculated with *P. chlororaphis* ST9, grown in the greenhouse and roots were then collected at T1 (10 dpi), T2 (28 dpi) and T4 (90 dpi) together with untreated controls for ST9 strain CFU counts. Results are summarized in Figure 2, which shows that the level of colonization after 10 days was significantly high (10^7^ cfu/g of root, -wet weight) then it decreased and stabilized around a value of 10^4^ cfu/g at 90 dpi. Colonies grown on TSA rifampicin plates were randomly chosen for identification through 16S rRNA gene amplification and sequencing to confirm their identity as *P. chlororaphis* ST9. Rifampicin-resistant colonies present in the untreated plants resulted to be a mixture of different bacteria: some of them were part of an analogue experiment carried out in concomitance (data not shown). These results evidenced that under the conditions tested, *P. chlororaphis* ST9 was an efficient root colonizer and able to persist in the rhizosphere.

**Figure 3:**
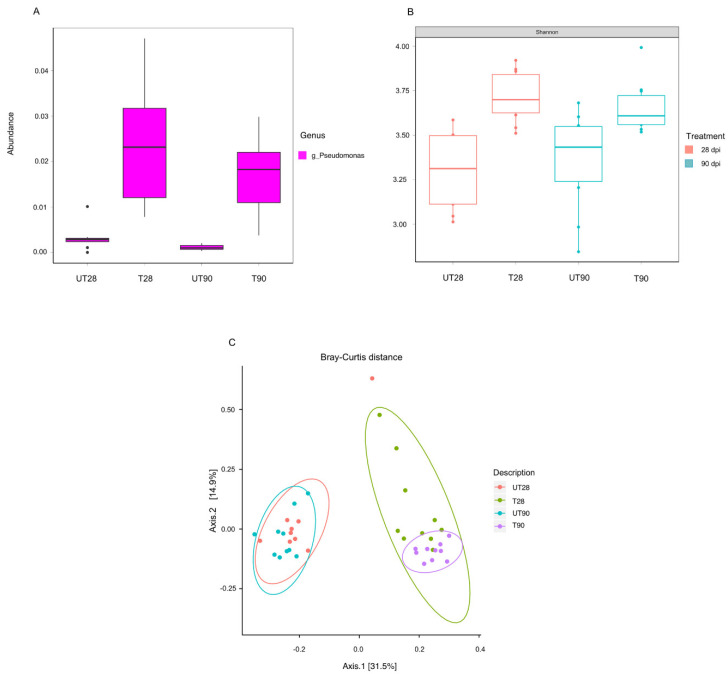
Microbiota analysis. (**A**) *Pseudomonas* genus abundance according to the plant treatment and time point; the Welch two sample *t*-test was used to compare untreated versus treated samples at 28 dpi (*p*-value = 0.0006793) and at 90 dpi (*p*-value = 0.0003417). (**B**) Alpha diversity (Shannon index) at community level in accordance with the treatment and the time point; the Wilcoxon rank sum test was used, *p*-value for untreated versus treated samples both at 28 and 90 dpi results < 0.01. (**C**) Principal component analysis of the samples in accordance to the treatment and the time point, similarity between the different communities was evaluated using the Bray–Curtis test; the bacterial communities of the untreated plants cluster together and differently from the bacterial communities colonizing the ST9 treated samples (*p*-value < 0.001).UT28: untreated samples at 28 dpi; T28: ST9 treated samples at 28 dpi; UT90: untreated samples at 90 dpi; T90: ST9 treated samples at 90 dpi.

**Figure 4:**
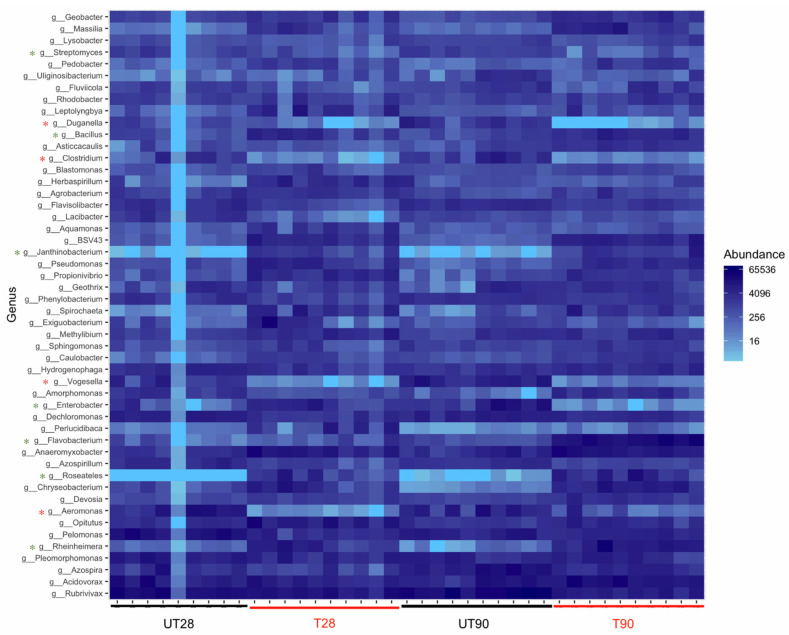
Heatmap showing the relative abundance (% of sequencing reads) of the 50 predominant genera. Rows are bacterial genera. Columns are samples. Colors indicate taxa with a higher (blue) or lower (light blue) relative abundance in each sample. Genera showing a different distribution and abundance level between the samples are highlighted with *: green if enriched in the treated samples, red if enriched in the untreated ones. UT28: untreated samples at 28 dpi; T28: ST9 treated samples at 28 dpi; UT90: untreated samples at 90 dpi; T90: ST9 treated samples at 90 d.

The possible effects of strain ST9 colonization on the rhizosphere microbial diversity were also investigated. 16S-rRNA community profiling was used to compare the microbial root community at 28 and 90 dpi in ST9 treated and untreated plants. The richness and diversity values of the bacterial communities after normalization are shown in Figure 3. The *Pseudomonas* genus was significantly more abundant (*p*-value < 0.001) in the *P. chlororaphis* ST9 inoculated plants as presented in Figure 3A. The presence of *P. chlororaphis* ST9 was confirmed by finding its 16S V3–V4 region sequence among the reads obtained by the NGS experiment. Significant differences (*p*-value = 0.000086) in Shannon alpha diversity (Figure 3B) were observed between inoculated and un-inoculated samples either at 28 or 90 dpi. Beta diversity analysis based on Bray Curtis distance (Figure 3C) was performed to compare the microbial community compositions of the two different tested conditions. Results highlighted differences of the microbial populations in samples that received different treatments. When *P. chlororaphis* was inoculated, several genera resulted differently distributed as evidenced by the heatmap (Figure 4 and in Supplementary Figures S3 and S4). Among the genera more abundant in untreated samples were *Duganella*, *Clostridium*, *Aeromonas*, *Enterobacter and Vogesella*; the majority of them are known to be animal/human pathogens, while only a few species, such as *Clostridium puniceum* [43] and *Enterobacter cloacae* [44], are correlated with plant diseases. On the other hand, genera such as *Janthinobacterium*, known for its antifungal features [45], *Flavobacterium*, often reported as plant growth-promoting rhizobacterium [46], *Bacillus*, *Paenibacillus*, *Bradyrhizobium* and others known for their PGP potential [47,48,49,50,51,52] were significantly more abundant in the *P. chlororaphis* ST9 treated communities.

### 2.3. Plant Gene Expression and Phenotypic Analysis of P. chlororaphis Inoculated Plants

**Figure 5:**
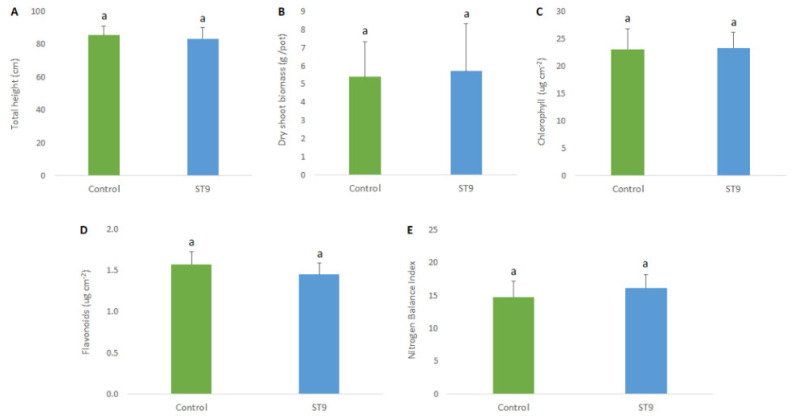
Total height (**A**), dry shoot biomass (**B**), chlorophyll (**C**) and flavonoid content (**D**), and NBI (**E**) in control un-inoculated and in ST9 inoculated plants. a indicate statistical differences. No statistical significance was observed (*t*-test, JMP7 (JMP^®^, Version 7. SAS Institute Inc., Cary, NC, USA, 1989-202).

In order to determine the effects of *P. chlororaphis* ST9 on plant growth, several phenotypic parameters were assayed. Statistical analyses were carried out on chlorophyll, flavonoid content and nitrogen balance index (NBI) as physiological parameters. In addition, plant height and dry shoot biomass at 90 dpi (after the flowering stage) were also established. Results did not show any statistical differences between control and ST9 inoculated plants [p(F) < 0.05] (Figure 5), although a tendency on higher NBI and lower flavonoid contents was measured in inoculated plants compared with the control. It was concluded that under the conditions tested, no significant plant beneficial effect was observed upon seed inoculation of *P. chlororaphis* ST9.

In order to investigate the effect on PGPR beneficial plant-bacteria interactions, RT-qPCR was performed on 14 genes selected for their role in ethylene and auxin pathways (Table 3).

**Figure 6:**
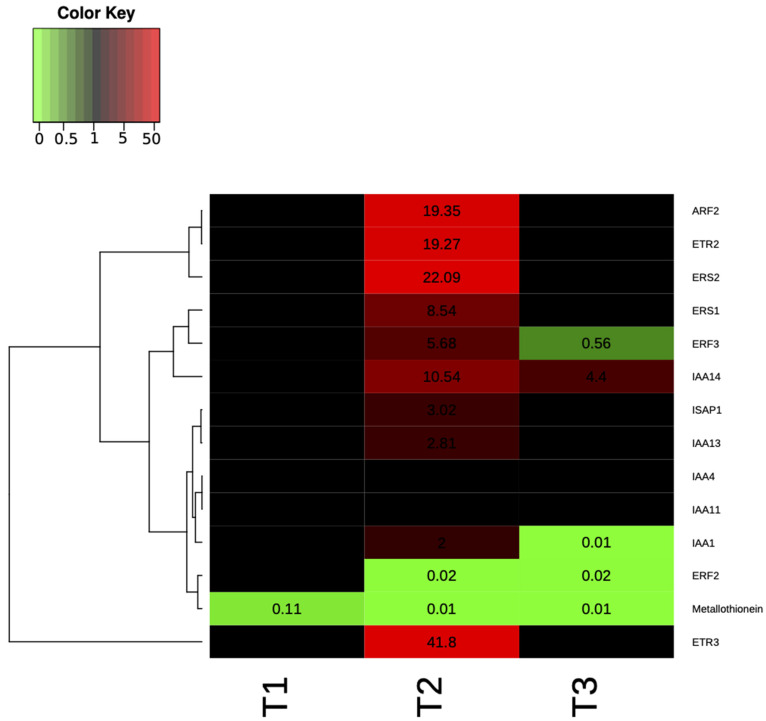
Heat map representation of the transcript levels (as result of the fold change calculated following 2^−ΔΔCT^) coupled to a hierarchical clustering in ST9 inoculated plants. Each column represents a time point, while each row represents a gene. Expression levels are coloured green for low intensities and red for high intensities (see scale at the top left corner). The black cells represent genes not significantly different from those of the untreated samples.

**Figure 7:**
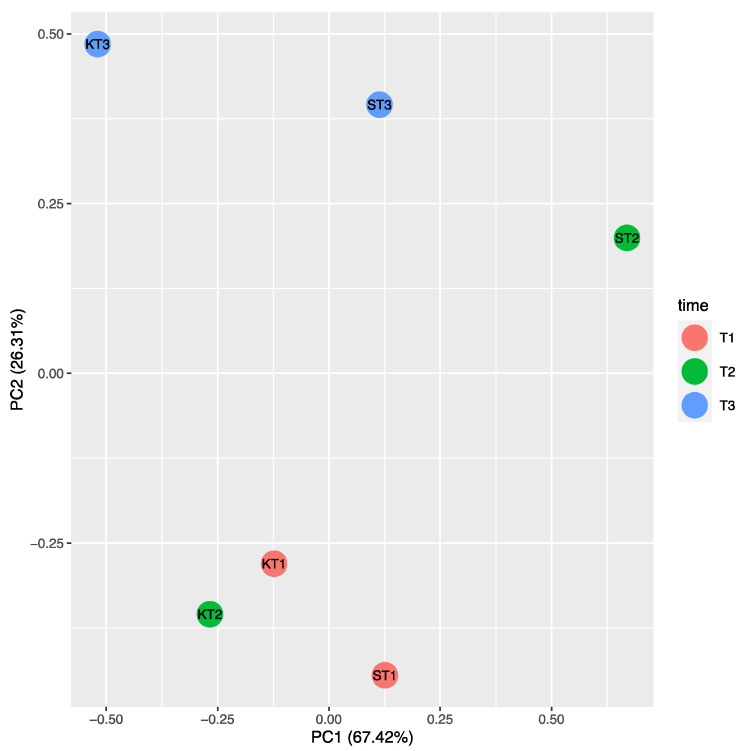
PCA carried out on ΔCT of untreated control (K) and ST9 inoculated plants (S) at each time point (T1 = 10 dpi; T2 = 28 dpi; T3 = 40 dpi). The cumulative variability percentage is given by the sum of 67.42% for PC1 and 26.31% for PC2, resulting in 93.73%. KT2 and ST2 are clearly separated in the space, unlike the other time point.

In Supplementary Table S2 the fold change was shown for genes that were significantly and not significantly differentially expressed, while in Figure 6 the heat map representation of the transcript levels coupled to a hierarchical clustering in ST9 inoculated plants is presented. At T2, significant differences in the expression of some loci were evidenced when compared to the other two time points. The most up- and down-regulated genes at T2 were *OsETR3* and *Osmetallothionein*, respectively. At T1, except for *Osmetallothionein,* there were no significantly differentially regulated genes. At T3 the most up-regulated gene was *OsIAA14*, while *OsERF3* was the most down-regulated. The only genes that were never significantly differentially expressed were *OsIAA11* and *OsIAA4.* The PCA on ΔCT data of control (un-inoculated) and ST9 inoculated samples for each replicate and time point showed, without outlier observations, a clear distinction between control and inoculated samples at T2, where we have found the highest number of differently regulated genes (Figure 7). On the other hand, there was no separation between data at T1 and at T3, where we have found a reduced number of regulated genes. In summary, several loci displayed different levels of gene expression upon inoculation with strain ST9; one gene was regulated at T1, while 12 and five genes were regulated at T2 and T3, respectively.

## 3. Discussion

This study presents the identification and characterization of a new *P. chlororaphis* strain. The work performed included root colonization and persistence, the effect on the rhizosphere microbiota, effect on targeted plant gene expression and a greenhouse plant growth promotion test. Results showed that *P. chlororaphis* ST9 is an efficient rice root colonizer: when inoculated it is integrated into the plant resident-microbiota affecting the expression of several plant genes.

After inoculation, *P. chlororaphis* was able to persist in the root compartment for 90 days without decreasing its abundance below a concentration of 10^4^ cfu/g of root. An initial good root colonization rate then slowly decreased in the later stages of plant growth has also been observed for other PGPR strains. For example, in Chaudhary et al. (2013) [57] *Azotobacter* strain ST24 colonized well the wheat rhizosphere decreasing during growth and reaching a density of 10^4^ cfu/mL at 90 days after sowing. Similarly, Solanki and Garg (2014) [58] reported that *Azotobacter* on *Brassica campestris* persisted at 30 and 60 day post-inoculation being approximately 2 × 10^4^ cfu per plant at 60 dpi. Mosquito et al. (2020) [59] demonstrated that a *Kosakonia* sp. strain inoculated in rice plants was persistent at 30 dpi, while it was undetectable at 60 and 90 dpi. The effect of strain ST9 inoculation on the total bacterial population evidenced an increase in biodiversity with enrichment of certain bacterial genera (e.g., *Janthinobacterium*, *Flavobacterium*, *Bacillus*, *Paenibacillus*, *Bradyrhizobium*) which are known to establish a beneficial association with plants [47,48,49,50,51,52].

Regardless of the efficient rhizosphere colonization ability, no plant beneficial effect on the rice inoculated with strain ST9 was observed under the condition tested here. In order to fully test potential PGP properties of strain ST9, more experimentation and conditions need to be tested, including challenging rice with biotic and abiotic stresses. The differences between the physiological parameters of the inoculated and uninoculated plants are not statistically significant; however, their tendency could be an indication of a decreased stress condition in ST9 inoculated plants. A similar trend was also observed by Andreozzi et al. (2019) [60] where NBI was significantly lower in the uninoculated control with respect to the *Herbaspirillum huttiense* RCA24 + *Enterobacter cloacae* RCA25 inoculated Baldo rice plants.

Gene expression studies were performed with 14 rice loci related to ethylene and auxin pathways together with genes coding for a metallothionein-like protein and a multiple stress-responsive zinc-finger protein; a role for these genes during rice-PGPR interaction has been demonstrated [53,54,56,61]. Genes *OsERS1*, *OsERS2*, *OsETR2*, *OsETR3*, homologs to *Arabidopsis thaliana* ethylene receptors, were transcribed at a higher level in the *P. chlororaphis* ST9 inoculated plants. Similarly, Vargas et al. (2012) [53] also observed an increase in expression of these loci when inoculated with *Azospirillum brasilense* and *Burkholderia kururiensis*. In their study, rice varieties having more BNF (biological nitrogen fixation) capacities showed higher bacterial colonization as well as up-regulation of ethylene receptors genes. In our study, these loci were significantly up-regulated at 28 dpi indicating that the Baldo rice variety displays good nitrogen-fixing capacity as previously reported [60]. The *OsERF2* and *OsERF3* genes, encoding for transcriptional factors related to ethylene, were also differentially expressed in ST9 inoculated plants indicating that this strain could be involved in the regulation of ethylene hormone levels. Indole-3-acetic acid (IAA) pathway modulation is important during an effective plant colonization, both by pathogenic and by nonpathogenic organisms [56]. In *P. chlororaphis* inoculated plants it was established that loci *OsIAA1*, *OsIAA13* and *OslAA14* were up-regulated at T2, *OsIAA1* was down-regulated at T3, while *OsIAA11* and *OsIAA4* were not differently transcribed in the three considered time points. Some of these trends are in accordance and others in contrast with previous observations [56,61]; this could be due to the different timing and experimental conditions applied. Expression of genes associated with defense can be also affected by bacterial presence in the rhizosphere [56]. *OsISAP1*, encoding for a multiple stress-responsive zinc-finger protein, was up-regulated at T2, suggesting an activation of the plant defense mechanisms. The gene coding for a metallothionein, which is a metal-binding protein involved in metal homeostasis, was always down-regulated at each time point in agreement with previous data [56]. The plant defense system could be primed by *P. chlororaphis* ST9 maintaining a low level of stress; future studies on the response to biotic stress of strain ST9 inoculated plant are needed in order to determine whether the changes in the expression levels of these loci provides immunity against microbial pathogens. 

## 4. Material and Methods

### 4.1. Strain Isolation, Growth and Identification

It was of interest to identify new bacterial isolates with PGP potential. Strains were recovered from one gram of uncultivated bulk soil from two distinct but very close sites, located in Padriciano, Trieste, Italy (45°39′32″ N, 13°50′28″ E). They were resuspended in 5 mL of PBS (phosphate buffer solution) and serial dilutions were performed and plated on 1/6 Tryptic Soy medium (BD, Sparks, MD, USA), solidified with 1.5% agar. Plates were incubated at 28 °C for 72 h and colonies were counted: the amount of colony-forming units of bacteria in both sites was of 1.3 × 10^5^ cfu/gr. Approximately 150 isolates showing distinct colony morphology (color and texture), were isolated through further streaking and also characterized for some biochemical features (KOH, catalase and oxidase activities). Bacteria showing different morphological and biochemical profiles were stored individually at −80 °C constituting a bacterial collection of 63 isolates. In vitro testing was then performed for P solubilization, IAA production and antagonistic activity against the plant pathogen *Dickeya zeae*. Sixteen isolates having at least 2 independent in vitro PGP-related phenotypes were identified by 16S rRNA sequencing (data not shown): briefly, primers fD1 and rR2 were used for the amplification of the 16S rRNA gene and primers 518F and 800R (Supplementary Table S1) were used for the sequencing control (Eurofins, Ebersberg, Germany). *P. chlororaphis* ST9 was one of these isolates and it was chosen for the rice inoculation experiments based on the activities observed in the primary screening and for the literature on its PGP and biocontrol (BC) potential.

### 4.2. In Vitro Phenotypic Characterization

The presence of lipolytic and proteolytic activities was determined by streaking the bacterial isolates on 1/6 TSA (Tryptic Soy Agar) medium amended with 1% Glyceryl tributyrin [62] and 2% of powder milk [63], respectively. Exopolysaccharide (EPS) production was tested by streaking the bacterial isolates on yeast extract mannitol medium [64] and indole acetic acid (IAA) production was verified as described by [65] using the Salkowski reagent. The 1-aminocyclopropane-1-carboxylic acid (ACC) deaminase activity was detected using M9 minimal medium with ACC as a unique N source [66], while the ability to solubilize P was verified using the NPRBB growth medium [67]. N-acyl homoserine lactone (AHL) production was assessed by T-streak technique, using the biosensor *Chromobacterium violaceum* CV026 after incubation for 1–2 days [68]. Motility was checked on M8 medium plates with 0.3% (swimming) or 0.5% (swarming) agar [69]. Anti-bacterial activity was tested by streaking plant pathogens *Dickeya zeae* and *Pseudomonas fuscovaginae* adjacent to a 24 h old streak of *P. chlororaphis* ST9 on LB medium; plates were incubated for 48 h at 28 °C and then evaluated. Antifungal activity was tested by streaking strain ST9 on TSA medium plates and placing, at 2 cm distance, a fragment of PDA agar contaminated with either *Magnaporthe oryzae, or Fusarium graminearum* or *Aspergillus nidulans.* Plates were checked after 7 days of incubation at 28 °C. Plant growth promotion traits were tested on 300 surface-sterilized rice seeds. In detail, 16 h pre-germinated seeds were submerged for 2 h in a ST9 bacterial suspension (OD_600_ 0.5) or PBS, as control; after inoculum application seeds were rinsed with sterile water and allowed to germinate in the dark at 30 °C. One hundred seeds were evaluated for emergence after 3 days, 100 seeds were grown for 10 days and then used to measure coleoptile length and the growth of the last 100 seeds was interrupted after 12 days for dry mass weight.

### 4.3. Generation of a Rifampicin-Resistant Spontaneous Mutants

The *P. chlororaphis* ST9 strain was grown in 1/6 TS medium for 16 h at 30 °C with 100 rpm shaking. A 1/100 dilution of the overnight culture was put in 1/6 TS medium with 15 µg/mL rifampicin and the culture was grown again in the same growth conditions. The procedure was repeated, increasing the concentration of Rifampicin (25, 50 and 100 µg/mL), and the final culture was plated on 1/6 TS. One colony was chosen and streaked on TSArif100; its identity, *P. chlororaphis* ST9, was confirmed again by 16S rRNA sequencing.

### 4.4. Bacterial Genomic DNA Extraction and Sequencing

Genomic DNA extraction for genome sequencing was performed with the pronase Sarkosyl lysis method [70]. Two micrograms of DNA, quantified through Nanodrop (Thermo Scientific, Waltham, MA, USA) and checked by gel electrophoresis, were used for genome sequencing by the Exeter Sequencing Service (University of Exeter, Exeter, UK). The sequence was carried out using the Illumina technique with the Hiseq 2500 platform with the 125 base pair-paired end system. Reads were assembled using SPAdes 3.9.03 [71]. The assembled *P. chlororaphis* ST9 genome was uploaded in the RAST Annotation Server [34] and was automatically annotated using the RASTtk annotation scheme [72]. The genome sequence is presented as a unique contig and it is available on the RAST server https://rast.nmpdr.org/?page=JobDetails&job=828746 (accessed on 21 February 2020); “guest” as login and password).

### 4.5. P. chlororaphis ST9 Taxonomy

Multi-locus Sequence Analysis (MLSA) was performed using 5 housekeeping genes: *recA* (recombinase A), *gyrB* (DNA gyrase subunit B), *rpoD* (RNA polymerase sigma factor), *carA* (Carbamoyl-phosphate synthase small chain) and *atpD* (ATP synthase subunit beta), plus the 16S rRNA locus. Sequences of these loci were obtained from the ST9 genome sequence. For the phylogenetic analysis, we concatenated the gene sequences in the following order: 16S-*recA-gyrB-rpoD-carA-atpD* resulting in a single sequence. For the same genes, orthologue sequences from 16 *P. chlororaphis* species were obtained from the NCBI database and chained. The used sequences are reported in Supplementary Materials 1. As an outgroup, we used the orthologue concatenate from *Pseudomonas putida KT2440 and P. fluorescens Pf-01*. The phylogenetic analysis was performed using the NGPhylogeny.fr public platform [73].

### 4.6. Plant Inoculation

Seeds of *O. sativa* L. cv. Baldo were surface-sterilized with 50% sodium hypochlorite solution (commercial bleach) for 60 min, rinsed with sterile water and incubated in a wet dark environment for seven days at 30 °C for germination. Two growth conditions (treatments) were considered: with and without the ST9 inoculation. The roots of 70 seedlings were soaked for 60 min in an ST9 RifR bacterial solution at 0.5 of OD_600_ (treated or inoculated plants), while the control was performed soaking the roots of 70 seedlings in a sterile PBS solution (untreated or un-inoculated plants). The inoculated and the control untreated seedlings were transferred to plastic tubes containing 0.4% water-agar and Hoagland solution for 72 h and then transplanted into pots in the greenhouse.

### 4.7. Greenhouse Experiments

For each of the two treatments, fourteen plastic pots (23 cm × 21 cm) were used, filled with non-sterile paddy field soil (47.8% sand, 9.4% clay, 42.8% silt, pH 6.4, organic matter 1.45%) taken from the experimental rice field of CREA-CI in Vercelli (VC, Italy). Pots were placed in the greenhouse under uncontrolled temperature, light and humidity parameters following the natural season trend from June to September 2019. In each pot, five seedlings, undergoing the same treatment, were sown. Plants were watered every day with tap water and kept in a greenhouse for 90 days, following the natural photoperiod. Four time points were considered: T1, 10 days post-inoculation (dpi); T2, 28 dpi; T3, 40 dpi and T4, 90 dpi. At T1, T2 and T3 ten plants per treatment were sampled, washed and stored at −80 °C for gene expression analysis. At T1, T2 and T4 ten plants per treatment were sampled and utilized for bacterial counting and microbiota analysis. A timeline of the experiment is presented in Supplementary Figure S5. The remaining 20 plants were used for physiological and morphological evaluations. In particular, nitrogen balance index (NBI), an indicator of the plant nitrogen status at the beginning of the flowering stage [74] was calculated as the ratio between chlorophyll (CHL) and flavonoid (FLA) concentration recorded by the DUALEX 4 Scientific (Dx4) chlorophyll meter (Force-A, Paris, France) [75]. Measurements were carried out on both adaxial and abaxial faces of the panicle leaf for each plant. The total height was also measured for each plant as well as the dry weight of shoots (obtained after 48 h at 65 °C).

### 4.8. Colonization Counts

The roots of 10 rice plants for each treatment at each time point were cleaned from the soil and washed thoroughly under tap water before processing them. For T1, complete roots were macerated, while for T2 and T4, 600 mg of root samples were macerated and resuspended in 2 mL of PBS. Serial dilutions, up to 10^−5^, were made and 100 μL of each dilution was plated in triplicate on TSA (Tryptic soy agar) and TSA with rifampicin (50 µg/L). Plates were incubated at 28 °C for 48 h and the emerged colonies were counted considering the dilution factor and the starting material weight in order to determine the number of total cultivable CFUs (colony forming units) and of rifampicin-resistant CFUs present in 1 g of roots.

### 4.9. In Vivo Biocontrol Activity of P. chlororaphis ST9 against Dickeya Zeae

Baldo rice seeds were surfaced sterilized and germinated for one week. Thirty seedling roots were submerged in PBS (control plants) while the other 30 were submerged in an ST9 bacterial suspension (OD_600_ = 0.5) for 60 min (ST9 treated plants). Seedlings were transplanted in soil (five seedlings per pot) and one week after transplantation 1 mL of an ST9 bacterial suspension (OD_600_ = 0.3) was inoculated directly into the soil, near the stem emergence site, of the already ST9 treated plants as a second inoculum. Twenty days after transplantation, 1 μL of *D. zeae* bacterial suspension OD_600_ = 0.3 was injected, at the base of the stem, in the control and ST9 treated plants. 10 plants of each treatment were used as control and injected with PBS. Seven days after infection lesions were scored. Lesions assume diverse phenotypes: very dark for few centimeters, light brown for longer fragment or discontinuous blackening. A Disease Severity Score Scale was set and a Disease Severity Score (DSS) was scored for each plant by 7 different persons.

### 4.10. Bacterial Genomic DNA Extraction, 16S rRNA Gene Amplicon Library Preparation and Sequencing

Five hundred mg of root samples from 10 ST9-treated and untreated plants at T2 and T4 time points were used to extract DNA and perform microbiome analysis. DNA was extracted using the PowerSoil Kit (Qiagen, Hilden, Germany) following supplier instructions. For the 16S amplicon libraries preparation, 12.5 ng of DNA, quantified using Nanodrop, was used for each sample to prepare the 16S rRNA amplicon libraries. Library preparation was done using the Illumina methodology following the 16S Metagenomic Sequencing Library Preparation protocol (https://emea.support.illumina.com/content/dam/illumina-support/documents/documentation/chemistry_documentation/16s/16s-metagenomic-library-prep-guide-15044223-b.pdf, accessed on 17 July 2021). Sequencing was performed at CBM scrl (Trieste, Italy) with MiSeq sequencing platform.

### 4.11. Microbiome Sequence Analysis

FASTQ files were demultiplexed using the QIIME 1.9.1 split_libraries_fastq.py script and then analyzed using DADA2 v1.4.0 [76] adapting the methods from the DADA2 Pipeline Tutorial (1.4) and including dereplication, singletons and chimera removing (sample inference was performed using the inferred error model, while chimeric sequences were removed using the removeBimeraDenovo function). The Greengenes (GG) database [77], giving a final Operational Taxonomic Unit (OTU) table, was employed to assign bacterial taxonomy using the assign Taxonomy function with a 97% sequence similarity. The resulting OTUs were clustered at genus taxonomic level obtaining the final profile and abundance of bacterial taxa in the different samples. Statistical analysis was performed using the vegan package version 2.5–4 [78] and phyloseq package [79] in R version 3.5.2 [80]. Relative abundances of OTUs between samples were calculated. To test the differential representation of microbial taxa in diverse samples the Deseq2 package [81] was used.

### 4.12. RNA Extraction and cDNA Conversion

The RNA from three biological replicates for each thesis at each time point was extracted using the RNeasy Plant Mini Kit (Qiagen, Hilden, Germany), according to the manufacturer’s instructions. After the RNA extraction, any amount of DNA was removed using DNase (RQ1 RNase-Free DNase, Promega, Madison, WI, USA) and measured using Qubit (Invitrogen, ThermoFisher Scientific, Waltham, MA, USA). The absence of genomic DNA was verified through PCR on RNA with the primers for the reference gene [55]. Total RNA was used for each sample to synthesize the cDNA, according to the SuperScript II Reverse Transcriptase (Invitrogen, ThermoFisher Scientific, Waltham, MA, USA) procedure using random primers.

### 4.13. Primer Selection

The genes analyzed in this study (Table 3) were selected based on their regulation and role during the interaction between PGPR and rice. In particular, in addition to gene coding for a metallothionein—like protein and a multiple stress-responsive zinc-finger protein [56], genes involved in ethylene and auxin pathways were considered [53,54,56]. Before RT-qPCR, all primers (Supplementary Table S1) were tested in silico on PRIMERBLAST and in PCR reactions on genomic DNA extracted from Baldo rice. The DNA extraction was performed using the DNeasy Plant Mini Kit (Qiagen, Hilden, Germany) according to the manufacturer’s instructions.

### 4.14. Gene Expression Analysis

Quantitative RT-PCR was carried out with 7500 Fast Real Time Systems (Applied Biosystem, ThermoFisher Scientific, Waltham, MA, USA). Each PCR reaction was conducted on a total volume of 10 μL, containing 1 μL cDNA, 5 μL SYBR Green Reaction Mix and 0.3 µL of each primer (10 μM) using a 96-well plate. The used primers are listed in ST1. The following PCR program, which includes the calculation of a melting curve, was used: 95 °C for 10 min, 40 cycles of 95 °C for 15 s, 60 °C for 1 min, 95 °C for 15 s, 60 °C for 1 min, and 95 °C for 15 s. All the reactions were performed for three biological and technical replicates. The baseline range and CT (cycle threshold) values were automatically calculated using the 7500 Fast Real Time Systems software. In order to compare data from different PCR runs or cDNA samples, the CT values of all the genes were normalized to the CT value of *OsACT1*, the reference gene. The candidate gene expression was normalized to that of the reference gene by subtracting the Ct value of the reference gene from the Ct value of the candidate gene efficiency correction, from the equation 2^−ΔΔCT^ [82], where ΔΔCT represents the ΔCT sample−ΔCT control.

### 4.15. Data Analysis

The statistical analysis of the phenotyping data was done using JMP7 (SAS Institute Inc., Cary, NC, USA, 1989-2019). Concerning gene expression, statistical analyses were carried out using REST 2009, version 2.0.13 (Qiagen, Hilden, Germany) [83], considering 0.05 as the *p*-value. Only significant expression values were considered and visualized as heat maps by a custom R (version 3.6.3) script (command “heatmap.2”). In order to reduce the data set dimension, a PCA (Principal Component Analysis) was carried out on ΔCT data for each biological replicate through R (version 3.6.3) (CRAN package “ggfortify”). The input files for R analysis were tabular filed in.csv including ID# genes and ID# samples.

## 5. Conclusions

Inoculated PGPR must interact or compete with other microorganisms in the rhizosphere microbiome and this can cause the short persistence of the inoculated bacteria possibly affecting its probiotic effects [2]. The ability of *P. chlororaphis* ST9 to successfully colonize and persist in the rhizosphere is an important trait for its possible use as bioinoculant for agriculture. Furthermore, the plethora of secondary metabolites and antimicrobial activities encoded in its genome and a biocontrol test against *D. zeae* infection make *P. chlororaphis* ST9 a potential candidate for biotic stress tolerance tests upon its inoculation. Plant gene expression analysis demonstrated that the presence of *P. chlororaphis* ST9 positively affects the expression of some plant hormonal pathways such as IAA and ethylene; further investigation should be carried out on rice defense genes in order to better clarify the type of interaction. *P. chlororaphis* strains are considered safe for the environment and human health (EPA, 2009) and their use in agriculture has been permitted through the application of live microorganism formulations [84,85] and via the production and purification of metabolites [35,86]. Future experiments with *P. chlororaphis* ST9 will reveal its full potential as a bioinoculant for PGP and its possible role in tolerance to abiotic and biotic stresses.

## Figures and Tables

**Table 1:** *P. chlororaphis* genome mining for PGP and biocontrol related genetic loci. ST9 genes ID were assigned by the RAST Annotation Server [34] during automatic annotation and can be used for retrieve gene sequences from the RAST server (https://rast.nmpdr.org/?page=JobDetails&job=828746, accessed on 21 February 2020); “guest” as login and password).

System/Compound	Target/Beneficial Effect	References	ST9 Genes
**Antagonism**			
Phenazine 1-carboxylic acid (PCA)	Antifungal redox-active antibiotic	[26]	7 genes cluster From fig|286.2086.peg.5005 to fig|286.2086.peg.5011 phzABCDEFG
2-hydroxy phenazine	Fungistatic and bacteriostatic	[35]	fig|286.2086.peg.5012; phzO
Tailocine R	Antibacterial Persistence within the rhizosphere microbiome	[36]	40 genes cluster From fig|286.2086.peg.1042 to fig|286.2086.peg.1082
Pirrolnitrin	Antifungal compound	[37,38]	4 genes cluster From fig|286.2086.peg.3071 to fig|286.2086.peg.3074; prnA, prnB, prnC, prnD
2-hexyl, 5-propyl resorcinol (HRP)	Antifungal compound	[21,39]	5 genes cluster From fig|286.2086.peg.4110 to fig|286.2086.peg.4114; darA, darB, darC, darR, darS
Hydrogen cyanide	Metalloenzymes inhibitor and antifungal	[37]	2 genes cluster fig|286.2086.peg.2368 and fig|286.2086.peg.2369
**Signal Interferences**			
Acyl-homoserine lactone acylase	Quorum quenching		fig|286.2086.peg.2769
N-acyl-L-homoserine lactone synthetase	Quorum sensing	[40]	3 systems fig|286.2086.peg.5003 and fig|286.2086.peg.5004; phzI, phzR fig|286.2086.peg.4930 and fig|286.2086.peg.4931; aurI, aurR fig|286.2086.peg.2434 and fig|286.2086.peg.2435; luxI, luxR
**Niche Colonization**			
poly-beta-1,6-N-acetyl-D-glucosamine	Biofilm adhesin	[41]	4 genes cluster From fig|286.2086.peg.20 to fig|286.2086.peg.24 pgaA, pgaB, pgaC, pgaD
Motility and chemotaxis	Flagella	[41]	6 genes cluster From fig|286.2086.peg.1427 to fig|286.2086.peg.1433 flgF, flgG, flgH, flgI, flgK, flgL 36 gene cluster From fig|286.2086.peg.1451 to fig|286.2086.peg.1486 9 gene cluster From fig|286.2086.peg.4398 to fig|286.2086.peg.4406
**Plant Protection**			
Chitinase	insecticidal	[42]	5 genes fig|286.2086.peg.1868 and fig|286.2086.peg.1869 fig|286.2086.peg.3224 and fig|286.2086.peg.3225 fig|286.2086.peg.1868 4987 chitin binding protein and chitinases
Proteases	insecticidal	[42]	fig|286.2086.peg.3194 aprX/prtA and other related genes
Lipases	insecticidal	[42]	fig|286.2086.peg.3118 phospholipase C and others related genes
rebB	insecticidal	[42]	2 genes fig|286.2086.peg.35 and fig|286.2086.peg.36 rebB-like protein (refractile inclusion bodies)
**Beneficial Activities to Plant**			
Phosphate solubilization	P solubilization enzyme		fig|286.2086.peg.702; alkD
Achromobactine	Fe chelation and competition		13 genes cluster From fig|286.2086.peg.3101 to fig|286.2086.peg.3113 Achromobactin synthesis, receptor, transport

**Table 2:** Phenotypic characterization of PGP features of *P. chlororaphis* ST9.

	In Vitro PGP Activity							In Vitro Antagonistic Activity					In Vivo Activity			
	Protease	Lipase	IAA Production	P Solubilization	ACC Deaminase	EPS Production	Motility	*Dickeya zeae*	*Pseudomonas fuscovaginae*	*Aspergillus nidulans*	*Fusarium graminearum*	*Magnaporthe oryzae*	Emergence 4 dpi	Coleoptile Length 10 dpi	Dry Weight Biomass 12 dpi	Bacterial Foot Rot Disease Control
*P. chlororaphis ST9*	+	+	-	+	-	-	+	+	+	+	+	+	-	+ *	-	+ **

* Non-parametric Mann–Whitney *t*-test; *p* value < 0.0001. ** Non-parametric Mann–Whitney *t*-test; *p* value < 0.05.

**Table 3:** List of the genes considered for plant gene expression.

Gene Name	Putative Function	Reference
*OsERS1*	Ethylene response sensor 1	[53]
*OsERS2*	Ethylene response sensor 2	[53]
*OsETR2*	Ethylene responsive 2	[53]
*OsETR3*	Ethylene responsive 3	[53]
*OsIAA1*	Auxin-responsive protein IAA1-like	[54]
*OsIAA4*	Auxin-responsive protein IAA4	[54]
*OsIAA11*	Auxin-responsive protein IAA11	[54]
*OsIAA13*	Auxin-responsive protein IAA13-like	[54]
*OsIAA14*	Auxin-responsive protein IAA14-like	[54]
*OsACT1*	Actin 1	[55]
*OsARF2*	Similar to auxin response factor 2	[56]
*OsERF2*	Similar to ethylene response factor 2	[56]
*OsERF3*	Similar to ethylene response binding factor 3	[56]
*OsISAP1*	Multiple stress-responsive zinc-finger protein	[56]
*Osmetallo-tionein*	Metallothionein-like protein type 1	[56]

## Data Availability

The genome sequence is presented as a unique contig and it is available on the RAST server (https://rast.nmpdr.org/?page=JobDetails&job=828746 (accessed 21 February 2020); “guest” as login and password).

## Supplementary Materials

The following are available online at https://www.mdpi.com/article/10.3390/plants10071466/s1, Figure S1: Phylogenetic tree reconstructed by the MSLA method, Figure S2: In vitro antimicrobial activity of *P. chlororaphis* ST9Phylogenetic tree reconstructed by the MSLA method, Figure S3: Differential representation of OTUs between ST9 inoculated and control samples at 28 dpi, Figure S4: Differential representation of OTUs between ST9 inoculated and control samples at 90 dpi, Figure S5: Time line of the sampling and use of the samples, Table S1: List of the primers used in this study, Table S2: Fold change at each time point, Supplementary Material 1: Sequences used for the taxonomic analysis of *P. chlororaphys* ST9.
