# Supplementary material for: Isolation and Characterization of Pseudomonas chlororaphis Strain ST9; Rhizomicrobiota and in Planta Studies
Source: Plants (Basel). 2021 Jul 17;10(7):1466. doi: 10.3390/plants10071466 (PMC8309335; doi:10.3390/plants10071466)
Supplement: Supplementary file 1 [file plants-10-01466-s001.zip › plants-1293920-supplementary.pdf]

Isolation and characterization of *Pseudomonas chlororaphis* strain ST9; rhizomicrobiota and *in planta* studies

Iris Bertani[1§], Elisa Zampieri[2,3§], Cristina Bez[1], Andrea Volante[2,4], Vittorio Venturi[1*] and Stefano Monaco[2*]

[1] International Centre for Genetic Engineering and Biotechnology, 34149 Trieste, Italy; bertani@icgeb.org (I.B.); bez@icgeb.org (C.B.)
[2] Council for Agricultural Research and Economics-Research Centre for Cereal and Industrial Crops, s.s. 11 to Torino, km 2.5, 13100 Vercelli, Italy; elisa.zampieri@ipsp.cnr.it (E.Z.); andrea.volante@crea.gov.it (A.V.)
[3] Institute for Sustainable Plant Protection, National Research Council, Strada delle Cacce 73, 10135 Turin, Italy
[4] Council for Agricultural Research and Economics-Research Centre for Vegetable and Ornamental Crops, Corso Inglesi 508, 18038 Sanremo, Italy (present address)
* Correspondence: venturi@icgeb.org (V.V.); stefano.monaco@crea.gov.it (S.M.)
§ Equal Contribution

Keywords: rice, plant growth promoting bacteria, qPCR, microbiome, inoculum persistence, genome analysis

This PDF file includes:

- Supplementary Figures S1-S5
- Supplementary Tables S1 and S2
- Supplementary Material S1
- Reference

Supplementary Figures

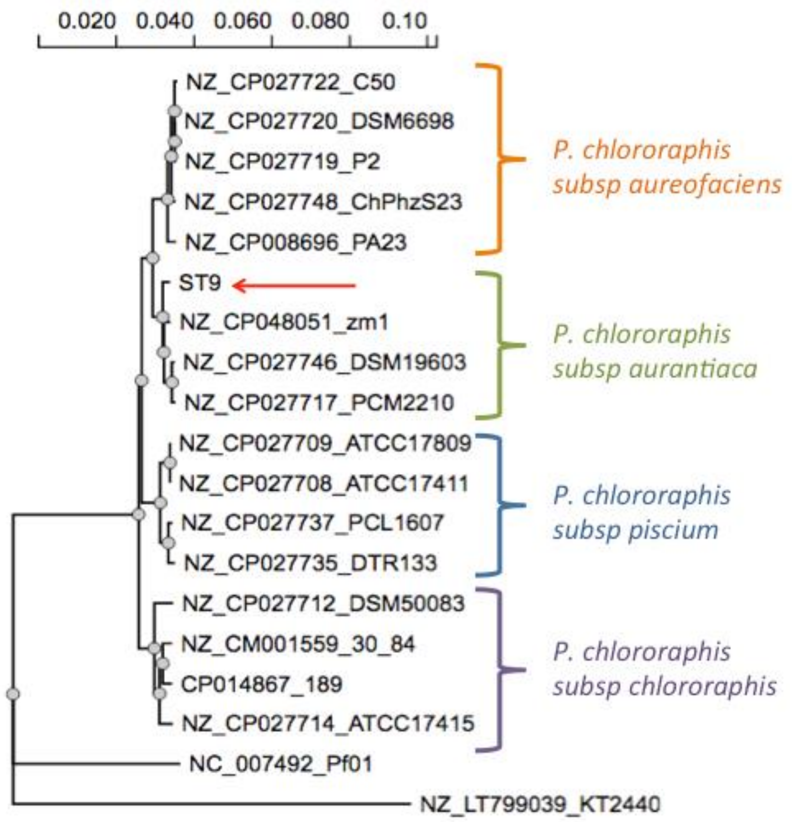

Supplementary Figure S1. Phylogenetic tree reconstructed by the MSLA method based on six concatenated gene sequences (16S rRNA, *recA*, *gyrB*, *rpoD*, *carA*, *atpD* – 9371 nt-) of 17 *Pseudomonas chlororaphis* strains. The strains *P. putida* KT2440 and *P. fluorescens* Pf01 served as outgroups. The 4 subspecies of *P. chlororaphis* cluster separately and ST9 is part of the *aurantiaca* subspecie. The phylogenetic analysis was performed using the NGPhylogeny.fr public platform.

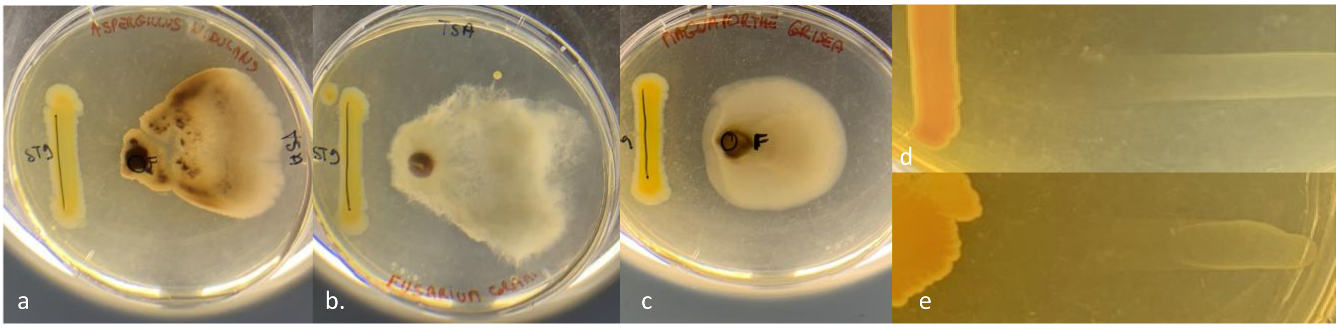

Supplementary Figure S2. *In vitro* antimicrobial activity of *P. chlororaphis* ST9. The antifungal activity was tested against a. *Aspergillus nidulans*, b. *Fusarium graminearum* and c. *Magnaporthe oryzae*. The antibacterial activity was verified against d. *Dickeya zeae* and e. *Pseudomonas fuscovaginae*.

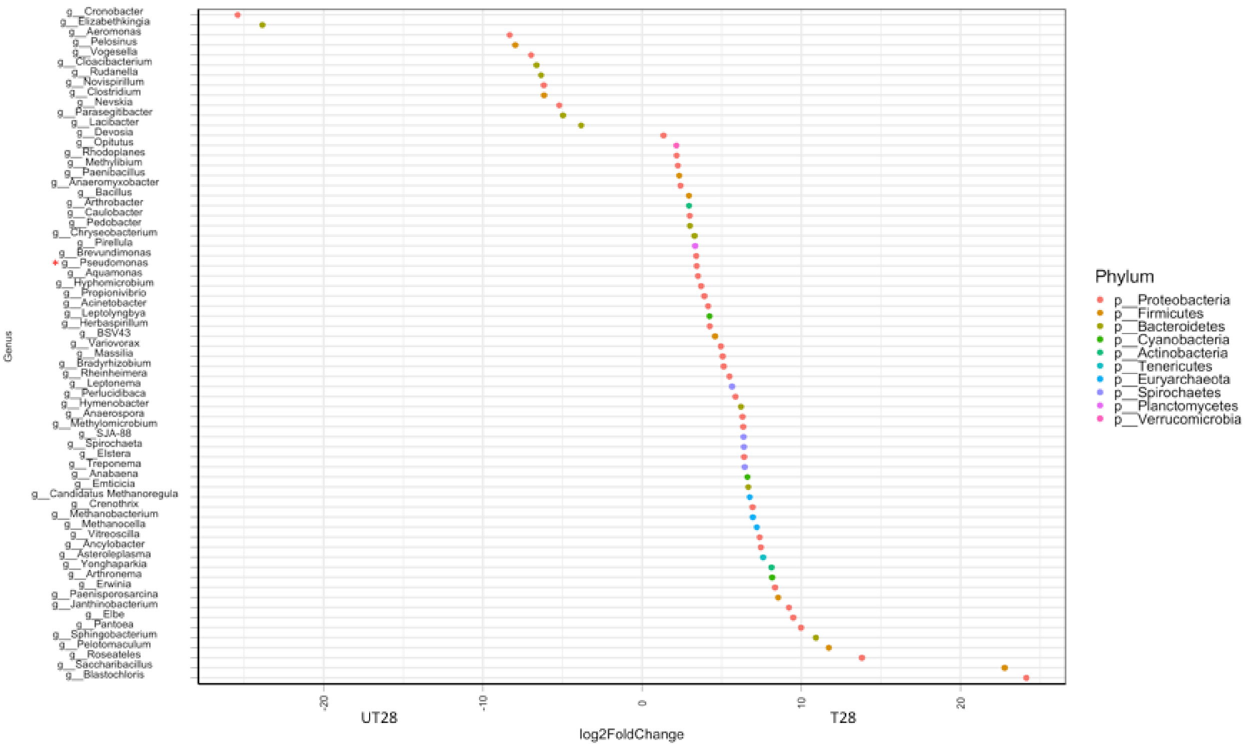

Supplementary Figure S3. Differential representation of OTUs between ST9 inoculated and control samples at 28 days post inoculation.

Differential abundance of OTUs between the two groups of tested samples was assessed by fitting a local regression model with a negative binomial distribution to the data and testing for differential abundance with a likelihood ratio test as implemented in the R package DESeq2 [1] in conjunction with the phyloseq package. Taxa are represented as dots in the graph of fold change. A negative log2Foldchange indicates taxa more abundant in untreated samples, while a positive log2Foldchange indicates taxa more abundant in treated samples. Samples with a *p*-value less than 0.0001 and mean representation over all samples higher than 1 are shown. UT28: untreated samples at 28 dpi; T28: ST9 treated samples at 28 dpi.

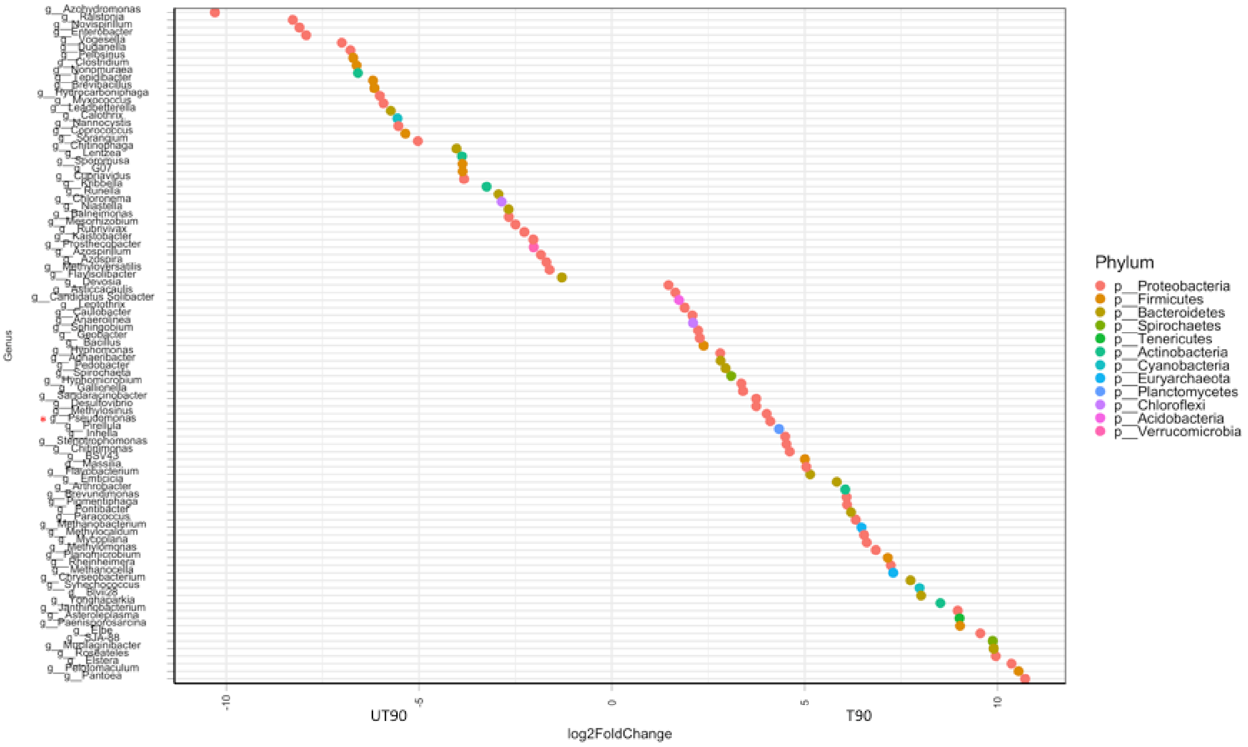

Supplementary Figure S4. Differential representation of OTUs between ST9 inoculated and control samples at 90 days post inoculation. Differential abundance of OTUs between the two groups of tested samples was assessed by fitting a local regression model with a negative binomial distribution to the data and testing for differential abundance with a likelihood ratio test as implemented in the R package DESeq2 [1] in conjunction with the phyloseq package. Taxa are represented as dots in the graph of fold change. A negative log2Foldchange indicates taxa more abundant in untreated samples, while a positive log2Foldchange indicates taxa more abundant in treated samples. Samples with a *p*-value less than 0.0001 and mean representation over all samples higher than 1 are shown. UT90: untreated samples at 90 dpi; T90: ST9 treated samples at 90 dpi.

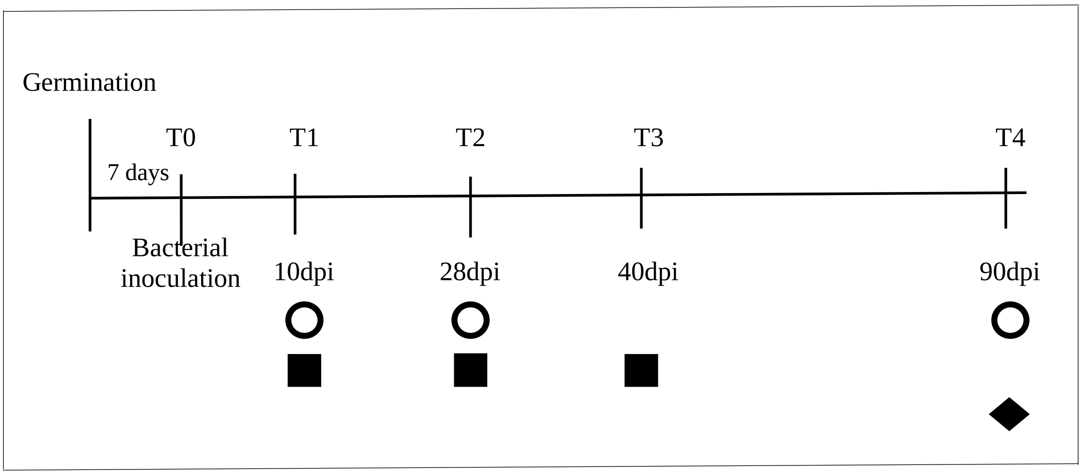

Supplementary Figure S5. Time line of the sampling and use of the samples: ○ bacterial counting and microbiome analysis, ■ plant gene expression analysis, ◆ plant physiological and morphological evaluations.

Supplementary Tables

Table S1: List of the primers used in this study

Primer name	Primer sequence (5'-3')	Gene name	Putative function	Reference
fD1	AGAGTTTGATCCTGGCTCAG	*16SrRNA*	16S rRNA amplification	[2]
rP2	ACGGCTACCTTGTTACGACTT			
518F	CCAGCAGCCGCGGTAATACG	*16SrRNA*	16S rRNA sequencing	[2]
800R	TACCAGGGTATCTAATCC			
OsERS1f	GAAAGGTCAGGCTTCTCTGAAATC	*OsERS1*	Ethylene response sensor 1	[3]
OsERS1r	ATGCCGTCGATCAATTTACAGTAG			
OsERS2f	CCTCGGGTTCGCTACCAAT	*OsERS2*	Ethylene response sensor 2	[3]
OsERS2r	GCATGGCGATGGCATCAT			
OsETR2f	CTTTAGCAGCACTGGGAGATGA	*OsETR2*	Ethylene responsive 2	[3]
OsETR2r	TGAGAACCATGAGGCTCTTTCA			
OsETR3f	CGAGCTGGCGCGAATT	*OsETR3*	Ethylene responsive 3	[3]
OsETR3r	TTAGACAAACAGACCTCCAGCAAA			
OsIAA1f	ACCAAGAGCCGCTCAATGAG	*OsIAA1*	auxin-responsive protein IAA1-like	[4]
OsIAA1r	ATCACACGTGGGCGAACATC			
OsIAA4f	GCTCTTGCTGGATGGGTATGA	*OsIAA4*	auxin-responsive protein IAA4	[4]
OsIAA4r	AGGTGATGGGCGTCTTGAAC			
OsIAA11f	AGTTGTCCATGGCGTTCCA	*OsIAA11*	auxin-responsive protein IAA11	[4]
OsIAA11r	TGCTCTCCTTCAGCTGCTGAT			
OsIAA13f	CAAGGATGGTGACTGGATGCT	*OsIAA13*	auxin-responsive protein IAA13-like	[4]
OsIAA13r	GATCCTCAAGCGTTTGCATGA			
OsIAA14f	CCGTCGCCTATGAGGACAA	*OsIAA14*	auxin-responsive protein IAA14-like	[4]
OsIAA14r	TTATCCGCAGCTTCTTGCAA			

OsACT1f<br>OsACT1r	GTATCCATGAGACTACATACAACT<br>TACTCAGCCTTGGCAATCCACA	*OsACT1*	Actin 1	[5]
ARF2-like f<br>ARF2-like r	GGCCTGAATCAAGTTGGAGATC<br>CTATCTGGCCGCGGAATAGTT	*OsARF2*	Similar to auxin response factor 2	[6]
ERF2-like f<br>ERF2-like r	CCGGCAAGGGTAGAGATGGT<br>TCAACATCGAAATCCCAAGAACT	*OsERF2*	Similar to ethylene response factor 2	[6]
ERF3-like f<br>ERF3-like r	TGCAGCAGCCTATGCAGATC<br>CGCGAGGACACTGCTTGAT	*OsERF3*	Similar to ethylene response binding factor 3	[6]
OsISAP1 f<br>OsISAP1 r	GCAATCCTCATCACACAGCAA<br>CCCTCTTGGTCTCAGGCTCTCT	*OsISAP1*	Multiple stress-responsive zinc-finger protein	[6]
Metallo-thionein f<br>Metallo-thionein r	CAAACTGCTCCTGCGGAAAG<br>ACGACGGTGGCCTTGGT	*Osmetallo-tionein*	metallothionein-like protein type 1	[6]

Table S2: Fold change at each time point

**Gene**	**Time**		
	T1	T2	T3
OsERF2	0.22	0.02*	0.02*
OsMetallothionein	0.11*	0.01*	0.01*
OsIAA1	0.22	0.002*	0.01*
OsIAA11	0.21	0.05	0.43
OsIAA13	2.54	2.81*	0.3
OsIAA14	1.8	10.54*	4.4*
OsISAP1	1.16	3.02*	0.62
OsETR3	1.72	41.8*	1.88
OsARF2	*0,69*	*19,35**	*1,16*
ERS1	0,3	8,54*	2,04
OsERS2	0,87	22,09*	2,46
OsERF3	0,81	5,68*	0,56*
OsIAA4	0,37	3,22	6,3
OsETR2	0,73	19,27*	2,19

*: statistically significant different (P< 0.05)

Supplementary Materials (SM)

Supplementary Material 1
Sequences used for the taxonomic analysis of *P. chlororaphys* ST9.

NCBI Reference Sequence: NZ_CP014867.1
Strain: 189
Chained genes: 16S rRNA, recA, gyrB, rpoD, carA, atpD
>NZ_CP014867.1_189
GAACTGAAGAGTTTGATCATGGCTCAGATTGAACGCTGGCGGCAGGCCTAACACATGC
AAGTCGAGCGGTAGAGAGAAGCTTGCTTCTCTTGAGAGCGGCGGACGGGTGAGTAATG
CCTAGGAATCTGCCTGGTAGTGGGGGATAACGTCCGGAAACGGACGCTAATACCGCAT
ACGTCCTACGGGAGAAAGCAGGGGACCTTCGGGCCTTGCGCTATCAGATGAGCCTAGG
TCGGATTAGCTAGTTGGTGAGGTAATGGCTCACCAAGGCGACGATCCGTAACTGGTCT
GAGAGGATGATCAGTCACACTGGAACTGAGACACGGTCCAGACTCCTACGGGAGGCA
GCAGTGGGGAATATTGGACAATGGGCGAAAGCCTGATCCAGCCATGCCGCGTGTGTGA
AGAAGGTCTTCGGATTGTAAAGCACTTTAAGTTGGGAGGAAGGGTACTTGCCTAATAC
GTGAGTATTTTGACGTTACCGACAGAATAAGCACCGGCTAACTCTGTGCCAGCAGCCG
CGGTAATACAGAGGGTGCAAGCGTTAATCGGAATTACTGGGCGTAAAGCGCGCGTAGG
TGGTTCGTTAAGTTGGATGTGAAATCCCCGGGCTCAACCTGGGAACTGCATCCAAAAC
TGGCGAGCTAGAGTATGGTAGAGGGTGGTGGAATTTCCTGTGTAGCGGTGAAATGCGT
AGATATAGGAAGGAACACCAGTGGCGAAGGCGACCACCTGGACTGATACTGACACTG
AGGTGCGAAAGCGTGGGGAGCAAACAGGATTAGATACCCTGGTAGTCCACGCCGTAA
ACGATGTCAACTAGCCGTTGGGAGCCTTGAGCTCTTAGTGGCGCAGCTAACGCATTAA
GTTGACCGCCTGGGGAGTACGGCCGCAAGGTTAAAACTCAAATGAATTGACGGGGGCC
CGCACAAGCGGTGGAGCATGTGGTTTAATTCGAAGCAACGCGAAGAACCTTACCAGGC
CTTGACATCCAATGAACTTTCCAGAGATGGATTGGTGCCTTCGGGAACATTGAGACAG
GTGCTGCATGGCTGTCGTCAGCTCGTGTCGTGAGATGTTGGGTTAAGTCCCGTAACGAG
CGCAACCCTTGTCCTTAGTTACCAGCACGTAATGGTGGGCACTCTAAGGAGACTGCCG
GTGACAAACCGGAGGAAGGTGGGGATGACGTCAAGTCATCATGGCCCTTACGGCCTGG
GCTACACACGTGCTACAATGGTCGGTACAGAGGGTTGCCAAGCCGCGAGGTGGAGCTA
ATCCCACAAAACCGATCGTAGTCCGGATCGCAGTCTGCAACTCGACTGCGTGAAGTCG
GAATCGCTAGTAATCGCGAATCAGAATGTCGCGGTGAATACGTTCCCGGGCCTTGTAC
ACACCGCCCGTCACACCATGGGAGTGGGTTGCACCAGAAGTAGCTAGTCTAACCTTCG
GGAGGACGGTTACCACGGTGTGATTCATGACTGGGGTGAAGTCGTAACAAGGTAGCCG
TAGGGGAACCTGCGGCTGGATCACCTCCTTAAATGGACGACAACAAGAAGAAAGCCTT
GGCTGCGGCCCTGGGTCAGATCGAACGTCAATTCGGCAAGGGTGCCGTAATGCGTATG
GGCGATCACGACCGCCAGGCGATCCCGGCCATTTCCACTGGCTCTCTGGGTCTGGACAT
CGCACTCGGCATCGGCGGCCTGCCAAAGGGTCGTATTGTTGAAATCTACGGTCCGGAA
TCGTCCGGTAAAACCACCCTGACCCTGTCCGTGATTGCCCAGGCACAGAAGATGGGCG
CCACTTGCGCCTTCGTCGACGCCGAGCATGCACTGGACCCGGAATACGCCGGCAAGCT
GGGGGTCAACGTTGACGACCTGCTGGTTTCCCAGCCGGACACCGGTGAACAGGCACTG
GAAATCACCGATATGCTGGTGCGCTCCAATGCCATCGACGTGATCGTGATCGACTCCGT
GGCGGCACTGGTGCCCAAGGCCGAGATCGAAGGCGAGATGGGCGACATGCACGTGGG
CCTGCAGGCCCGCCTGATGTCCCAGGCGCTACGCAAGATCACCGGCAACATCAAGAAC
GCCAACTGCCTGGTGATCTTCATCAACCAGATCCGTATGAAAATCGGCGTGATGTTCGG
CAGCCCGGAAACCACCACCGGTGGTAACGCGCTGAAGTTCTACGCTTCGGTTCGTCTG
GATATCCGTCGTACTGGCGCGGTGAAGGAAGGTGACGAAGTCGTCGGTAGCGAAACCC
GGGTCAAGATCGTCAAGAACAAGGTGGCTCCACCGTTCCGCCAGGCTGAATTCCAGAT

CCTGTACGGCAAGGGTATCTACCTGAACGGCGAGATCATCGATCTGGGCGTGCTGCAT
GGTTTCCTCGAGAAGTCCGGTGCCTGGTACAGCTACCAGGGCAACAAGATCGGTCAGG
GCAAGGCCAACTCGGCCAAGTTCCTGCAGGACAACCCGGAAATCGGTAATGCCCTCGA
GAAGCAGATTCGCGACAAGCTGCTGGCTCCGACCGCTGATGTCAAAGCTTCGCCGGTC
AACGAGACCATCGATGACATGGCCGACGCGGATATCTGAATGAGCGAAGAAAACACG
TACGACTCGAGCAGCATTAAAGTGCTGAAAGGTTTGGATGCCGTACGCAAACGTCCCG
GTATGTACATTGGTGACACCGACGATGGCAGCGGTCTGCACCATATGGTGTTCGAGGT
GGTCGATAACTCGATCGACGAAGCTCTGGCCGGCCATTGCGACGACATCAGCATCATC
ATCCACCCGGACGAATCCATTACCGTGCGTGACAACGGTCGCGGCATCCCGGTAGACG
TGCATAAAGAAGAAGGCGTTTCCGCAGCCGAGGTCATCATGACTGTGCTGCACGCCGG
CGGTAAGTTCGACGATAACTCCTACAAAGTATCCGGCGGTCTGCACGGTGTGGGTGTG
TCGGTAGTGAACGCCCTGTCCGAAGAGCTGGTCCTGACCGTTCGCCGCAGTGGCAAGA
TCTGGGAACAGACCTACGTTCACGGTGTGCCTCAGGCGCCTATGGCGATCGTCGGTGA
CAGCGAAACCACCGGTACCCAGATTCACTTCAAGGCTTCCAGCGAGACCTTCAAGAAC
ATCCACTTCAGCTGGGACATCCTGGCCAAGCGGATTCGTGAACTGTCCTTCCTCAACTC
CGGTGTCGGTATCGTTCTGAAGGACGAGCGCAGCGGCAAGGAAGAGCTGTTCAAGTAC
GAAGGCGGCTTGCGTGCGTTCGTTGAATACCTGAACACCAACAAGACTGCGGTCAACC
AGGTGTTCCACTTCAACGTGCAGCGTGAAGATGGCATCGGCGTGGAAATCGCCCTGCA
GTGGAACGACAGCTTCAACGAGAACCTGCAGTGCTTCACCAACAACATTCCGCAGCGC
GACGGCGGCACCCACCTGGTGGGCTTCCGTTCGGCGCTGACGCGTAACCTGAACAACT
ACATCGAGCAGGAAGGCCTGGCGAAGAAGCACAAGGTCGCCACCACCGGTGACGATG
CCCGCGAAGGCCTGACCGCGATCATTTCGGTCAAGGTGCCGGATCCGAAGTTCAGCTC
CCAGACCAAAGACAAGCTGGTGTCTTCCGAAGTGAAGACCGCGGTCGAACAGGAAAT
GGGTAAGTACTTCTCCGACTTCCTGCTGGAAAACCCGAACGAAGCCAAACTGGTGGTC
GGCAAGATGCTCGACGCCGCCCGTGCCCGTGAAGCGGCGCGTAAAGCCCGTGAGATGA
CCCGTCGTAAAGGCGCGCTGGATATCGCCGGCCTGCCGGGCAAACTGGCGGACTGCCA
GGAAAAAGACCCTGCCCTTTCCGAACTCTACCTGGTGGAAGGTGACTCTGCTGGCGGC
TCCGCCAAGCAGGGACGCAACCGTAAGACCCAGGCGATCCTGCCGCTCAAGGGCAAG
ATCCTCAACGTCGAGAAAGCGCGCTTCGACAAGATGATTTCCTCGCAAGAGGTCGGCA
CCTTGATCACTGCACTCGGTTGCGGCATCGGCCGCGAAGAGTACAACATCGACAAGCT
GCGTTATCACAACATCATCATCATGACCGACGCCGACGTCGACGGTTCGCACATCCGT
ACCCTACTCCTGACCTTCTTCTTCCGTCAGTTGCCGGAGCTGATCGAGCGTGGCTACAT
CTACATCGCTCAACCGCCGCTGTACAAGGTCAAGAAAGGCAAGCAAGAGCAATACATC
AAAGACGACGACGCCATGGAAGAGTACATGACGCAGTCGGCCCTGGAAGATGCGAGC
CTGCACCTGAACGAAGAAGCACCGGGTATTTCCGGCGAGGCGCTGGAGCGCCTGGTGA
ACGACTTCCGCATGGTCATGAAGACCCTCAAGCGTCTGTCGCGCCTCTACCCTCAAGAG
CTGACCGAGCACTTCATCTACCTGCCGGCCGTGAGCCTGGAGCAACTCTCCGATCACGC
GGCGATGCAGGATTGGCTGGCCCAATATGAAGTCCGCCTGCGCACCGTCGAGAAGTCC
GGCCTGGTCTACAAGGCCAGCCTGCGTGAAGACCGTGAACGTAATGTGTGGCTGCCAG
AGGTCGAACTGATCTCCCACGGCCTGTCGAACTACGTCACCTTCAACCGCGACTTCTTC
GGCAGCAATGACTACAAGACCGTCGTCACCCTCGGCGCTCAACTGAGCTCCCTGCTGG
ACGAAGGTGCTTATATTCAGCGCGGCGAGCGCAAGAAGGCGGTGACCGAGTTCAAGG
AAGCCCTGGACTGGCTGATGACCGAAAGCACCAAGCGCCATACCATCCAGCGATACAA
AGGTCTGGGTGAGATGAACCCGGATCAGCTGTGGGAAACCACCATGGACCCAAGCGTG
CGCCGCATGCTCAAGGTCACTATCGAAGACGCCATCGGCGCCGACCAGATCTTCAACA
CCCTGATGGGTGATGCGGTCGAGCCTCGTCGTGACTTCATCGAAAGCAACGCCCTGGC
GGTATCCAACCTGGACTTCTGAATGTCCGGAAAAGCGCAACAGCAGTCTCGCCTCAAA
GAGTTGATCAGCCGTGGTCGTGAGCAGGGTTACCTGACTTACGCGGAGGTCAACGACC
ACCTGCCGGAGGATATTTCAGATCCGGAACAGGTGGAAGACATCATCCGCATGATCAA
CGACATGGGGATCAACGTATTCGAGAGTGCTCCGGATGCGGATGCCCTTTTGTTGGCC
GAAGCCGATACCGACGAAGCAGCAGCTGAAGAAGCCGCCGCAGCGTTGGCGGCTGTG

GAAACCGACATTGGTCGCACTACCGACCCAGTGCGTATGTACATGCGCGAAATGGGTA
CGGTAGAGCTGCTCACACGTGAAGGCGAAATCGAAATCGCCAAGCGTATCGAAGAGG
GCATCCGTGAAGTGATGGGCGCGATCGCGCACTTCCCTGGCACGGTTGAGCACATCCT
CTCCGAATACACTCGCGTCACCACCGAAGGTGGTCGCCTGTCCGACGTCCTGAGCGGTT
ACATCGACCCGGACGACGGCATCGCGCCGCCTGCCGCCGAAGTACCACCGCCTGTCGA
TGCCAAGGCCGCGAAAGCGGACGACGACACCGACGACGATGACGCCGAAGCCAGTGA
CGACGAAGAAGAAGCCGAAAGCGGTCCGGATCCGGTCATCGCAGCCCAGCGCTTTGGC
GCCGTTGCCGACCAGATGGAAATCACTCGCAAGGCGCTGAAAAAGCACGGTCGTGAAC
ACAAGCAAGCCCTGGCTGAAATGCTGGCCCTGGCTGAACTGTTCATGCCGATCAAACT
GGTTCCGAAGCAATTCGAAGGCCTGGTTGAACGCGTTCGTAGCGCCCTGGATCGCCTG
CGTCAGCAAGAGCGCGCGATCATGCAACTCTGTGTTCGTGATGCTCGCATGCCACGCG
CCGACTTCCTGCGCCAGTTCCCTGGCAATGAAGTGGACGAAAGCTGGTCCGATGCACT
GGCCAAAGGCAAGGCCAAGTACGCCGAAGCCATCGGTCGCCTGCAACCGGACATCATC
CGTTGCCAGCAGAAGCTGACCGCGCTCGAGACCGAGACCGGCCTGACGATCGCCGAGA
TCAAGGACATCAACCGTCGCATGTCGATCGGCGAGGCCAAGGCCCGTCGCGCGAAGAA
AGAGATGGTCGAAGCCAACTTGCGCCTGGTGATCTCCATCGCCAAGAAGTACACCAAC
CGTGGCCTGCAGTTCCTGGACCTGATCCAGGAAGGCAACATCGGCTTGATGAAAGCGG
TAGACAAGTTCGAATACCGTCGCGGCTACAAGTTCTCGACTTATGCCACCTGGTGGATC
CGTCAGGCGATCACTCGCTCGATCGCCGACCAGGCCCGCACCATCCGTATTCCGGTGC
ACATGATCGAGACGATCAACAAGCTCAACCGTATTTCCCGGCAGATGCTGCAGGAAAT
GGGTCGCGAACCGACCCCGGAAGAGCTGGGCGAACGCATGGAAATGCCTGAGGACAA
GATCCGCAAGGTATTGAAGATCGCTAAAGAGCCGATCTCCATGGAAACCCCGATCGGT
GATGACGAAGACTCCCATCTGGGCGACTTCATCGAAGACTCGACCATGCAGTCGCCAA
TCGATGTCGCCACCGTTGAGAGCCTCAAGGAAGCGACTCGCGAAGTTCTCTCCGGCCT
CACTGCCCGTGAAGCCAAGGTACTGCGCATGCGCTTCGGCATCGACATGAATACCGAC
CACACCCTCGAGGAAGTCGGTAAGCAGTTCGACGTTACCCGTGAGCGGATTCGCCAGA
TCGAAGCCAAGGCGCTGCGCAAGCTGCGCCACCCGACGAGAAGCGAGCACCTGCGCTC
CTTCCTCGACGAGTAATTGACTAAGCCAGCCATACTCGCCCTTGCTGATGGCAGCATTT
TTCGCGGCGAAGCCATTGGAGCCGACGGTCAAACCGTTGGTGAGGTGGTGTTTAACAC
CGCAATGACCGGCTATCAGGAAATCCTTACCGATCCTTCCTACGCCCAACAGATCGTTA
CCCTGACTTACCCGCATATCGGCAATACCGGCACCACGCCGGAAGACGCCGAGTCCGA
TCGTGTCTGGTCGGCCGGTCTGGTGATTCGCGACCTGCCACTGGTTGCGAGCAACTGGC
GTAACACCCTGTCCCTGTCCGATTACCTGAAAGCCAACAACGTTGTGGCGATCGCCGGT
ATCGACACCCGTCGCCTGACGCGCATCCTGCGCGAGAAAGGCGCGCAGAACGGCTGCA
TCATAGCCGGCGACAACATTTCCGACGAAGCGGCGATTGCCGCAGCGCGCGGCTTCCC
TGGCCTGAAAGGCATGGATTTGGCGAAGGTCGTCAGCACCAAGGAAAGCTACGAGTG
GCGCTCCAGCGTCTGGAGCCTGAAGACCGACAGTCATCCGACTATCGAGGCTTCCGAG
CTGCCGTACCACGTGGTCGCCTACGACTACGGCGTCAAGCTGAACATCCTGCGCATGCT
GGTCGAGCGCGGTTGCCGCGTGACCGTAGTGCCTGCGCAAACTTCGGCCAGCGACGTC
CTGGCACTCAAGCCTGACGGTGTGTTCCTGTCCAACGGCCCTGGCGACCCCGAGCCTTG
CGATTACGCCATCCAGGCGATCAAGGATGTGCTGGAAACCGAGATCCCGGTCTTCGGT
ATCTGCCTGGGCCACCAACTGCTGGCACTGGCCTCCGGCGCCAAGACGGTGAAGATGG
GCCACGGCCACCACGGTGCCAACCACCCGGTCCAGGACCTGGACAGCGGTGTAGTGAT
GATCACCAGCCAGAACCACGGTTTTGCGGTGGACGAAGCCACCCTGCCAGGCAACGTG
CGGGCGATCCACAAATCGCTGTTCGACGGCACCCTGCAAGGCATCGAACGTACCGACA
AGAGCGCATTCAGCTTCCAGGGCCACCCTGAAGCGAGCCCGGGCCCGAACGATGTGGC
GCCGCTGTTCGATCGTTTCATCAACGAGATGGCCAAGCGACGCTGAATGAGTAGCGGA
CGTATCGTTCAAATCATCGGCGCCGTTATCGACGTGGAATTTCCACGCGACAGCGTACC
GAGCATCTACGACGCCTTGAAGGTTCAAGGCGCCGAAACCACTCTGGAAGTTCAGCAG
CAGCTGGGCGACGGCGTGGTACGTACCATTGCGATGGGCTCCACCGAGGGCTTGAAGC
GCGGTCTGGACGTCAACAACACTGGCGCAGCCATCTCCGTACCGGTCGGTAAAGCGAC

CCTGGGCCGGATCATGGACGTACTGGGCAACCCGATCGACGAAGCTGGTCCGATCGGC
GAAGAAGAGCGTTGGGGCATTCACCGTCCTGCGCCGTCCTTCGCTGAACAAGCTGGCG
GCAACGACCTCCTGGAAACCGGCATCAAGGTTATCGACCTGGTTTGCCCGTTCGCCAA
GGGCGGTAAAGTCGGTCTGTTTGGTGGTGCCGGTGTGGGCAAAACCGTAAACATGATG
GAACTGATCCGTAACATCGCCATCGAGCACAGCGGTTATTCCGTGTTCGCCGGTGTGG
GTGAGCGTACTCGTGAGGGTAACGACTTCTACCACGAGATGAAGGATTCCAACGTTCT
GGACAAAGTGGCACTGGTATACGGCCAGATGAACGAGCCGCCGGGAAACCGTCTGCG
CGTAGCTCTGACCGGCCTGACCATGGCCGAGAAGTTCCGTGACGAAGGTAACGACGTT
CTGCTGTTCGTCGACAACATCTATCGTTACACCCTGGCCGGTACCGAAGTATCCGCACT
GCTGGGCCGTATGCCTTCCGCAGTAGGTTACCAGCCGACCCTGGCTGAAGAGATGGGC
GTTCTGCAAGAACGTATCACTTCGACCAAGCAAGGCTCGATCACCTCGATCCAAGCGG
TATACGTACCTGCGGACGACTTGACCGACCCGTCGCCAGCGACCACCTTCGCCCACTTG
GACGCCACCGTCGTACTGTCCCGTGACATCGCTTCCCTGGGTATCTACCCAGCGGTAGA
TCCACTGGACTCGACTTCCCGTCAGCTGGACCCGAACGTGATCGGCAACGAGCACTAT
GAAACCGCTCGCGGCGTTCAGTACGTGCTGCAGCGCTACAAAGAGCTGAAGGACATCA
TTGCGATCCTGGGTATGGACGAACTGTCCGAAGCCGACAAGCAACTGGTATCCCGCGC
TCGTAAGATCCAGCGCTTCCTGTCGCAGCCGTTCTTCGTGGCTGAAGTCTTCACTGGTT
CTCCAGGCAAATACGTTTCCCTGAAAGATACCATCGCTGGCTTCAAAGGCATCCTCAAC
GGTGACTACGACCACCTGCCAGAACAAGCGTTCTACATGGTCGGCGGCATCGAAGAAG
CGATCGAGAAAGCCAAGAAACTGTAA

NCBI Reference Sequence: NZ_ CP027714.1
Strain: ATCC17415
Chained genes: 16S rRNA, recA, gyrB, rpoD, carA, atpD
>NZ_CP027714_ATCC17415
GAACTGAAGAGTTTGATCATGGCTCAGATTGAACGCTGGCGGCAGGCCTAACACATGC
AAGTCGAGCGGTAGAGAGAAGCTTGCTTCTCTTGAGAGCGGCGGACGGGTGAGTAATG
CCTAGGAATCTGCCTGGTAGTGGGGGATAACGTCCGGAAACGGACGCTAATACCGCAT
ACGTCCTACGGGAGAAAGCAGGGGACCTTCGGGCCTTGCGCTATCAGATGAGCCTAGG
TCGGATTAGCTAGTTGGTGAGGTAATGGCTCACCAAGGCGACGATCCGTAACTGGTCT
GAGAGGATGATCAGTCACACTGGAACTGAGACACGGTCCAGACTCCTACGGGAGGCA
GCAGTGGGGAATATTGGACAATGGGCGAAAGCCTGATCCAGCCATGCCGCGTGTGTGA
AGAAGGTCTTCGGATTGTAAAGCACTTTAAGTTGGGAGGAAGGGTACTTACCTAATAC
GTGAGTATTTTGACGTTACCGACAGAATAAGCACCGGCTAACTCTGTGCCAGCAGCCG
CGGTAATACAGAGGGTGCAAGCGTTAATCGGAATTACTGGGCGTAAAGCGCGCGTAGG
TGGTTCGTTAAGTTGGATGTGAAATCCCCGGGCTCAACCTGGGAACTGCATCCAAAAC
TGGCGAGCTAGAGTATGGTAGAGGGTGGTGGAATTTCCTGTGTAGCGGTGAAATGCGT
AGATATAGGAAGGAACACCAGTGGCGAAGGCGACCACCTGGACTGATACTGACACTG
AGGTGCGAAAGCGTGGGGAGCAAACAGGATTAGATACCCTGGTAGTCCACGCCGTAA
ACGATGTCAACTAGCCGTTGGGAGCCTTGAGCTCTTAGTGGCGCAGCTAACGCATTAA
GTTGACCGCCTGGGGAGTACGGCCGCAAGGTTAAAACTCAAATGAATTGACGGGGGCC
CGCACAAGCGGTGGAGCATGTGGTTTAATTCGAAGCAACGCGAAGAACCTTACCAGGC
CTTGACATCCAATGAACTTTCCAGAGATGGATTGGTGCCTTCGGGAACATTGAGACAG
GTGCTGCATGGCTGTCGTCAGCTCGTGTCGTGAGATGTTGGGTTAAGTCCCGTAACGAG
CGCAACCCTTGTCCTTAGTTACCAGCACATAATGGTGGGCACTCTAAGGAGACTGCCG
GTGACAAACCGGAGGAAGGTGGGGATGACGTCAAGTCATCATGGCCCTTACGGCCTGG
GCTACACACGTGCTACAATGGTCGGTACAGAGGGTTGCCAAGCCGCGAGGTGGAGCTA
ATCCCACAAAACCGATCGTAGTCCGGATCGCAGTCTGCAACTCGACTGCGTGAAGTCG
GAATCGCTAGTAATCGCGAATCAGAATGTCGCGGTGAATACGTTCCCGGGCCTTGTAC
ACACCGCCCGTCACACCATGGGAGTGGGTTGCACCAGAAGTAGCTAGTCTAACCTTCG
GGAGGACGGTTACCACGGTGTGATTCATGACTGGGGTGAAGTCGTAACAAGGTAGCCG

TAGGGGAACCTGCGGCTGGATCACCTCCTTAAATGGACGACAACAAGAAGAAAGCCTT
GGCTGCGGCCCTGGGTCAGATCGAACGTCAATTCGGCAAGGGTGCCGTAATGCGTATG
GGCGATCACGACCGCCAGGCGATCCCGGCCATTTCCACTGGCTCTCTGGGTCTGGACAT
CGCACTCGGCATCGGCGGCCTGCCAAAGGGCCGTATTGTTGAAATCTACGGTCCGGAA
TCGTCCGGTAAAACCACCCTGACCCTGTCCGTGATTGCCCAGGCACAGAAGATGGGCG
CCACTTGCGCCTTCGTCGACGCCGAGCACGCACTGGACCCGGAATACGCCGGCAAGCT
GGGGGTCAATGTTGACGACCTGCTGGTTTCCCAGCCGGACACCGGTGAACAGGCACTG
GAAATCACCGACATGCTGGTGCGCTCCAATGCCATCGACGTGATCGTGATCGACTCCG
TGGCGGCACTGGTGCCCAAGGCCGAGATCGAAGGCGAGATGGGCGACATGCACGTGG
GCCTGCAGGCCCGCCTGATGTCCCAGGCGCTGCGCAAGATCACCGGCAACATCAAGAA
CGCCAACTGCCTGGTGATCTTCATCAACCAGATCCGTATGAAAATCGGCGTGATGTTCG
GCAGCCCGGAAACCACCACCGGTGGTAACGCGCTGAAGTTCTACGCTTCGGTTCGTCT
GGATATCCGTCGTACTGGCGCGGTGAAGGAAGGTGACGAAGTCGTCGGTAGCGAAACC
CGGGTCAAGATCGTCAAGAACAAGGTGGCTCCACCGTTCCGCCAGGCTGAATTCCAGA
TCCTGTACGGCAAGGGTATCTACCTGAACGGCGAGATCATCGATCTGGGCGTGCTGCA
CGGTTTCCTCGAGAAGTCCGGTGCCTGGTACAGCTACCAGGGCAACAAGATCGGTCAG
GGCAAGGCCAACTCGGCCAAGTTCCTGCAGGACAACCCGGAAATCGGTAACGCCCTCG
AGAAGCAGATTCGCGACAAGCTGCTGGCTCCGACCGCTGATGTCAAAGCTTCGCCGGT
CAACGAGACCATCGATGACATGGCCGACGCGGATATCTGAATGAGCGAAGAAAACAC
GTACGACTCGAGCAGCATTAAAGTGCTGAAAGGTTTGGATGCCGTACGCAAACGTCCC
GGTATGTACATTGGTGACACCGACGATGGCAGCGGTCTGCACCATATGGTGTTCGAGG
TGGTCGATAACTCGATCGACGAAGCTCTGGCCGGCCATTGCGACGACATCAGCATCAT
CATCCACCCGGACGAATCCATTACCGTGCGTGACAACGGTCGCGGCATCCCGGTAGAC
GTGCATAAAGAAGAAGGCGTTTCCGCAGCCGAGGTCATCATGACCGTACTGCACGCCG
GCGGTAAGTTCGACGATAACTCCTACAAAGTATCCGGCGGTCTGCACGGTGTGGGTGT
GTCGGTAGTGAACGCCCTGTCCGAAGAACTGGTCCTGACCGTTCGCCGCAGTGGCAAG
ATCTGGGAACAGACCTACGTTCATGGTGTGCCTCAGGCACCTATGGCGATCGTCGGTG
ACAGCGAAACCACTGGTACCCAGATTCACTTCAAGGCTTCCAGCGAGACCTTCAAGAA
CATCCACTTCAGCTGGGACATCCTGGCCAAGCGGATTCGTGAACTGTCCTTCCTCAACT
CCGGTGTCGGTATCGTTCTGAAGGACGAGCGCAGCGGCAAGGAAGAGCTGTTCAAGTA
CGAAGGCGGCTTGCGTGCGTTCGTTGAATACCTGAACACCAACAAGACTGCGGTCAAC
CAGGTGTTCCACTTCAATGTGCAGCGTGAAGACGGCATCGGCGTGGAAATCGCCCTGC
AGTGGAACGACAGCTTCAACGAGAACCTGCAGTGCTTTACCAACAACATTCCGCAGCG
CGACGGCGGCACCCACCTGGTGGGCTTCCGTTCGGCGCTTACGCGTAACCTGAACAAC
TACATCGAGCAGGAAGGCCTGGCGAAGAAGCACAAGGTCGCCACCACCGGTGACGAT
GCCCGCGAAGGCCTGACCGCGATCATTTCGGTCAAGGTGCCGGATCCGAAGTTCAGCT
CCCAGACCAAAGACAAGCTGGTTTCTTCCGAAGTGAAGACCGCAGTCGAACAGGAAAT
GGGCAAGTACTTCTCCGACTTCCTGCTGGAAAACCCGAACGAAGCCAAGCTGGTGGTC
GGCAAGATGCTCGACGCCGCCCGTGCCCGTGAAGCGGCGCGTAAAGCCCGTGAGATGA
CCCGTCGTAAAGGCGCGCTGGATATCGCCGGCCTGCCGGGCAAACTGGCGGACTGCCA
GGAAAAAGACCCTGCCCTTTCCGAACTCTACCTGGTGGAAGGTGACTCTGCTGGCGGC
TCCGCCAAGCAGGGACGCAACCGTAAGACCCAGGCGATCCTGCCGCTCAAGGGCAAG
ATCCTCAACGTCGAGAAAGCGCGCTTCGACAAGATGATTTCCTCGCAAGAGGTCGGCA
CCTTGATCACTGCACTCGGTTGCGGCATCGGTCGCGAAGAGTACAACATCGACAAGCT
GCGTTATCACAACATCATCATCATGACCGACGCCGACGTCGACGGTTCGCACATCCGC
ACCCTGCTGCTGACCTTCTTCTTCCGTCAGTTGCCGGAGCTGATCGAGCGTGGCTACAT
CTACATCGCTCAACCGCCGCTGTACAAGGTCAAGAAAGGCAAGCAAGAGCAATACATC
AAAGACGACGACGCCATGGAAGAGTACATGACGCAGTCGGCCCTGGAAGATGCGAGC
CTGCACCTGAACGAAGAAGCACCGGGTATTTCCGGCGAGGCGCTGGAGCGCCTGGTGA
ACGACTTCCGCATGGTCATGAAGACCCTCAAGCGTCTGTCGCGCCTCTACCCTCAAGAG
CTGACCGAGCACTTCATCTACCTGCCGGCCGTGAGCCTGGAGCAACTCTCCGATCACGC

GGCGATGCAGGATTGGCTGGCCCAATATGAAGTCCGCCTGCGCACCGTCGAGAAGTCC
GGCCTGGTCTACAAGGCCAGCCTGCGTGAAGACCGTGAACGTAATGTGTGGCTGCCAG
AGGTCGAACTGATCTCCCACGGTCTGTCGAACTACGTCACCTTCAACCGCGACTTCTTC
GGCAGCAATGACTACAAGACCGTCGTCACCCTCGGCGCTCAACTGAGCTCCCTGCTGG
ACGAAGGCGCTTATATTCAGCGCGGCGAGCGCAAGAAGGCGGTGACCGAGTTCAAGG
AAGCCCTGGACTGGCTGATGACCGAAAGCACCAAGCGCCACACCATCCAGCGATACAA
AGGTCTGGGCGAGATGAACCCGGATCAGCTGTGGGAAACCACCATGGACCCAAGCGT
GCGCCGCATGCTCAAGGTCACCATCGAAGACGCCATCGGCGCCGACCAGATCTTCAAC
ACCCTGATGGGTGATGCGGTCGAGCCTCGTCGTGACTTCATCGAAAGCAACGCTCTGG
CGGTATCCAACCTGGACTTCTGAATGTCCGGAAAAGCGCAACAGCAGTCTCGCCTCAA
AGAGTTGATCAGCCGTGGTCGTGAGCAGGGTTACCTGACTTACGCGGAGGTCAACGAC
CACCTGCCGGAGGATATTTCAGATCCGGAACAGGTGGAAGACATCATCCGCATGATCA
ACGACATGGGGATCAACGTATTCGAGAGTGCTCCGGATGCGGATGCCCTTTTGTTGGC
CGAAGCCGATACCGACGAAGCAGCAGCTGAAGAAGCCGCCGCAGCGTTGGCGGCTGT
GGAAACCGACATTGGGCGCACTACCGACCCAGTGCGTATGTACATGCGCGAAATGGGT
ACGGTAGAGCTGCTCACACGTGAAGGCGAAATCGAAATCGCCAAGCGTATCGAAGAG
GGTATCCGTGAAGTGATGGGCGCGATCGCGCACTTCCCTGGCACGGTTGAGCACATCC
TCTCCGAATACACTCGCGTCACCACCGAAGGTGGTCGCCTGTCCGACGTCCTGAGCGGT
TACATCGACCCGGACGACGGCATCGCGCCGCCTGCCGCCGAAGTACCACCGCCTGTCG
ATGCCAAGGCCGCGAAAGCGGACGACGACACCGACGACGATGACGCCGAAGCCAGTG
ACGACGAAGAAGAAGCCGAAAGCGGTCCGGATCCGGTCATTGCAGCCCAGCGCTTTGG
CGCCGTTGCCGACCAGATGGAAATCACTCGCAAGGCGCTGAAAAAGCACGGTCGTGAA
CACAAGCAAGCCCTGGCTGAAATGCTGGCCCTGGCTGAACTGTTCATGCCGATCAAAC
TGGTTCCGAAGCAATTCGAAGGCCTGGTTGAACGCGTTCGTAGCGCCCTGGATCGCCT
GCGTCAGCAAGAGCGCGCGATCATGCAACTCTGTGTTCGTGATGCTCGCATGCCACGC
GCCGACTTCCTGCGCCAGTTCCCTGGCAATGAAGTGGACGAAAGCTGGTCCGACGCAC
TGGCCAAAGGCAAGGCCAAGTACGCCGAAGCCATCGGTCGCCTGCAGCCGGACATCAT
CCGTTGCCAGCAGAAGCTGACCGCGCTCGAGACCGAGACCGGCCTGACGATCGCCGAG
ATCAAGGACATCAACCGTCGCATGTCGATCGGCGAGGCCAAGGCCCGTCGCGCGAAGA
AAGAGATGGTCGAAGCCAACTTGCGTCTGGTGATCTCCATCGCCAAGAAGTACACCAA
CCGTGGCCTGCAGTTCCTCGACCTGATCCAGGAAGGCAACATCGGCTTGATGAAAGCG
GTAGACAAGTTCGAATACCGTCGCGGCTACAAGTTCTCGACTTATGCCACCTGGTGGAT
CCGTCAGGCGATCACTCGCTCGATCGCCGACCAGGCCCGCACCATCCGTATTCCGGTGC
ACATGATCGAGACGATCAACAAGCTCAACCGTATTTCCCGGCAGATGCTGCAGGAAAT
GGGTCGCGAACCGACCCCGGAAGAGCTGGGCGAACGCATGGAAATGCCTGAGGACAA
GATCCGCAAGGTATTGAAGATCGCTAAAGAGCCGATCTCCATGGAAACCCCGATCGGT
GATGACGAAGACTCCCATCTGGGCGACTTCATCGAAGACTCGACCATGCAGTCGCCAA
TCGATGTCGCCACCGTTGAGAGCCTCAAGGAAGCGACTCGCGAAGTTCTCTCCGGCCT
CACTGCCCGTGAAGCCAAGGTACTGCGCATGCGCTTCGGCATCGACATGAATACCGAC
CACACCCTCGAGGAAGTCGGTAAGCAGTTCGACGTTACCCGTGAGCGGATTCGCCAGA
TCGAAGCCAAGGCGCTGCGCAAGCTACGCCATCCGACGAGAAGCGAGCACCTGCGCTC
CTTCCTCGACGAGTAATTGACTAAGCCAGCCATACTCGCCCTTGCTGATGGCAGCATTT
TTCGCGGCGAAGCCATTGGAGCCGACGGTCAAACCGTTGGTGAGGTGGTGTTTAACAC
CGCAATGACCGGCTATCAGGAAATCCTTACCGATCCTTCCTACGCCCAACAGATCGTTA
CCCTGACTTACCCGCATATCGGCAATACCGGCACCACGCCGGAAGACGCCGAGTCCGA
TCGTGTCTGGTCGGCCGGTCTGGTGATTCGCGACCTGCCACTGGTTGCGAGCAACTGGC
GTAATACCCTGTCCCTGTCCGACTACCTGAAAGCCAACAACGTTGTGGCGATCGCCGGT
ATCGACACCCGTCGCCTGACGCGCATCCTGCGCGAGAAAGGCGCGCAGAACGGCTGCA
TCATGGCCGGCGACAATATCTCCGACGAAGCGGCGATTGCCGCAGCGCGCGGCTTCCC
TGGTCTGAAAGGCATGGATCTGGCGAAGGTCGTCAGCACCAAGAAAACCTACGAGTGG
CGCTCCAGCGTCTGGAGCCTGAAGACCGACAGTCATCCGACTATCGAGGCTTCCGAGC

TGCCGTACCACGTGGTCGCCTACGACTACGGCGTCAAGCTGAACATCCTGCGCATGCT
GGTCGAGCGTGGTTGCCGCGTGACCGTAGTGCCTGCGCAAACTCCGGCCAGCGACGTC
CTGGCACTCAAGCCTGACGGTGTGTTCCTGTCCAACGGCCCTGGCGACCCCGAGCCTTG
CGATTACGCCATCCAGGCGATTAAGGACGTGCTGGAAACCGAGATCCCGGTCTTCGGT
ATCTGCCTGGGCCACCAACTGCTGGCATTGGCCTCCGGCGCCAAGACGGTGAAGATGG
GCCACGGCCACCACGGTGCCAACCACCCGGTCCAGGACCTGGACAGCGGTGTAGTGAT
GATCACCAGCCAGAACCACGGTTTTGCGGTGGACGAAGCCACCCTGCCAGGCAACGTG
CGGGCGATCCACAAATCGCTGTTCGACGGCACCCTGCAAGGCATCGAACGTACCGACA
AGAGCGCATTCAGCTTCCAGGGCCACCCTGAAGCGAGCCCGGGCCCGAACGATGTGGC
GCCGCTGTTCGATCGTTTCATCAACGAGATGGCCAAGCGACGCTGAATGAGTAGCGGA
CGTATCGTTCAAATCATCGGCGCCGTTATCGACGTGGAATTTCCACGCGACAGCGTACC
GAGCATCTACGACGCCTTGAAGGTTCAAGGCGCCGAAACCACTCTGGAAGTTCAGCAG
CAGCTGGGCGACGGCGTGGTACGTACCATTGCGATGGGCTCCACCGAGGGCTTGAAGC
GCGGTCTGGACGTCAACAACACTGGCGCAGCCATCTCCGTACCGGTCGGTAAAGCGAC
CCTGGGCCGGATCATGGACGTACTGGGCAACCCGATCGACGAAGCTGGCCCGATCGGC
GAAGAAGAGCGTTGGGGCATTCACCGTCCTGCGCCGTCCTTCGCTGAACAAGCTGGCG
GCAACGACCTCCTGGAAACCGGCATCAAGGTTATCGACCTGGTTTGCCCGTTCGCCAA
GGGCGGTAAAGTCGGTCTGTTCGGTGGTGCCGGTGTGGGCAAAACCGTAAACATGATG
GAACTGATCCGTAACATCGCCATCGAGCACAGCGGTTATTCCGTGTTCGCCGGTGTGG
GTGAGCGTACTCGTGAGGGTAACGACTTCTACCACGAGATGAAGGATTCCAACGTTCT
GGACAAAGTGGCACTGGTATACGGCCAGATGAACGAGCCGCCGGGAAACCGTCTGCG
CGTAGCTCTGACCGGCCTGACCATGGCCGAGAAGTTCCGTGACGAAGGTAACGACGTT
CTGCTGTTCGTCGACAACATCTATCGTTACACCCTGGCCGGTACCGAAGTATCCGCACT
GCTGGGCCGTATGCCTTCCGCAGTAGGTTACCAGCCGACCCTGGCTGAAGAGATGGGC
GTTCTGCAAGAACGTATCACTTCGACCAAGCAAGGCTCGATCACCTCGATCCAAGCGG
TATACGTACCTGCGGACGACTTGACCGACCCGTCGCCAGCGACCACCTTCGCCCACTTG
GACGCCACCGTCGTACTGTCCCGTGACATCGCTTCCCTGGGTATCTACCCAGCGGTAGA
TCCACTGGACTCGACTTCCCGTCAGCTGGACCCGAACGTGATCGGCAACGAGCACTAT
GAAACCGCTCGCGGCGTTCAGTACGTGCTGCAGCGCTACAAAGAGCTGAAGGACATCA
TTGCGATCCTGGGTATGGACGAACTGTCCGAAGCCGACAAGCAACTGGTATCCCGCGC
TCGTAAGATCCAGCGCTTCCTGTCGCAGCCGTTCTTCGTGGCTGAAGTCTTCACTGGTT
CTCCAGGCAAATACGTTTCCCTGAAAGACACCATCGCTGGCTTCAAAGGCATCCTCAA
CGGTGACTACGACCACCTGCCAGAACAAGCGTTCTACATGGTCGGCGGCATCGAAGAA
GCGATCGAGAAAGCCAAGAAACTGTAA

NCBI Reference Sequence: NZ_CP027735.1
Strain: DTR133
Chained genes: 16S rRNA, recA, gyrB, rpoD, carA, atpD
>NZ_CP027735_DTR133
GAACTGAAGAGTTTGATCATGGCTCAGATTGAACGCTGGCGGCAGGCCTAACACATGC
AAGTCGAGCGGTAGAGAGGTGCTTGCACCTCTTGAGAGCGGCGGACGGGTGAGTAATG
CCTAGGAATCTGCCTGGTAGTGGGGGATAACGTTCGGAAACGGACGCTAATACCGCAT
ACGTCCTACGGGAGAAAGCAGGGGACCTTCGGGCCTTGCGCTATCAGATGAGCCTAGG
TCGGATTAGCTAGTTGGTGAGGTAATGGCTCACCAAGGCGACGATCCGTAACTGGTCT
GAGAGGATGATCAGTCACACTGGAACTGAGACACGGTCCAGACTCCTACGGGAGGCA
GCAGTGGGGAATATTGGACAATGGGCGAAAGCCTGATCCAGCCATGCCGCGTGTGTGA
AGAAGGTCTTCGGATTGTAAAGCACTTTAAGTTGGGAGGAAGGGTACTTACCTAATAC
GTGAGTATTTTGACGTTACCGACAGAATAAGCACCGGCTAACTCTGTGCCAGCAGCCG
CGGTAATACAGAGGGTGCAAGCGTTAATCGGAATTACTGGGCGTAAAGCGCGCGTAGG
TGGTTCGTTAAGTTGAATGTGAAATCCCCGGGCTCAACCTGGGAACTGCATCCAAAAC
TGGCGAGCTAGAGTATGGTAGAGGGTGGTGGAATTTCCTGTGTAGCGGTGAAATGCGT

AGATATAGGAAGGAACACCAGTGGCGAAGGCGACCACCTGGACTGATACTGACACTG
AGGTGCGAAAGCGTGGGGAGCAAACAGGATTAGATACCCTGGTAGTCCACGCCGTAA
ACGATGTCAACTAGCCGTTGGGAGCCTTGAGCTCTTAGTGGCGCAGCTAACGCATTAA
GTTGACCGCCTGGGGAGTACGGCCGCAAGGTTAAAACTCAAATGAATTGACGGGGGCC
CGCACAAGCGGTGGAGCATGTGGTTTAATTCGAAGCAACGCGAAGAACCTTACCAGGC
CTTGACATCCAATGAACTTTCCAGAGATGGATTGGTGCCTTCGGGAACATTGAGACAG
GTGCTGCATGGCTGTCGTCAGCTCGTGTCGTGAGATGTTGGGTTAAGTCCCGTAACGAG
CGCAACCCTTGTCCTTAGTTACCAGCACGTAATGGTGGGCACTCTAAGGAGACTGCCG
GTGACAAACCGGAGGAAGGTGGGGATGACGTCAAGTCATCATGGCCCTTACGGCCTGG
GCTACACACGTGCTACAATGGTCGGTACAGAGGGTTGCCAAGCCGCGAGGTGGAGCTA
ATCCCATAAAACCGATCGTAGTCCGGATCGCAGTCTGCAACTCGACTGCGTGAAGTCG
GAATCGCTAGTAATCGCGAATCAGAATGTCGCGGTGAATACGTTCCCGGGCCTTGTAC
ACACCGCCCGTCACACCATGGGAGTGGGTTGCACCAGAAGTAGCTAGTCTAACCTTCG
GGAGGACGGTTACCACGGTGTGATTCATGACTGGGGTGAAGTCGTAACAAGGTAGCCG
TAGGGGAACCTGCGGCTGGATCACCTCCTTAAATGGACGACAACAAGAAGAAAGCCTT
GGCTGCGGCCCTGGGTCAGATCGAACGTCAATTCGGCAAGGGTGCCGTAATGCGTATG
GGCGATCACGATCGCCAGGCGATCCCGGCCATTTCCACTGGCTCTCTGGGTCTGGACAT
CGCACTCGGCATCGGCGGCCTGCCAAAAGGCCGTATTGTTGAAATCTACGGTCCGGAA
TCGTCCGGTAAAACCACCCTGACCCTGTCGGTGATTGCCCAGGCACAGAAGATGGGCG
CCACCTGCGCCTTCGTCGACGCCGAGCACGCACTGGACCCGGAGTACGCCGGCAAACT
GGGGGTCAACGTTGACGACCTGCTGGTTTCCCAGCCGGACACCGGCGAACAGGCGCTG
GAAATCACCGACATGCTGGTGCGCTCCAATGCCATCGACGTGATCGTGATCGACTCCG
TGGCGGCGCTGGTACCCAAGGCCGAGATCGAAGGCGAGATGGGCGACATGCACGTGG
GCCTGCAGGCTCGCCTGATGTCCCAGGCGCTGCGCAAGATCACCGGTAACATCAAGAA
CGCCAACTGCCTGGTGATCTTCATCAACCAGATCCGTATGAAAATCGGCGTGATGTTCG
GCAGCCCGGAAACCACCACCGGTGGTAACGCGCTGAAGTTCTACGCTTCGGTTCGTCT
GGATATCCGTCGTACTGGCGCGGTGAAGGAAGGTGACGAAGTCGTCGGTAGCGAAACC
CGGGTCAAGATCGTCAAGAACAAGGTGGCTCCACCCTTCCGTCAAGCTGAGTTCCAGA
TCCTGTACGGCAAGGGTATCTACCTGAACGGCGAGATCATCGATCTGGGCGTGCTGCA
CGGTTTCCTAGAGAAGTCCGGTGCCTGGTACAGCTACCAGGGCAACAAGATCGGTCAG
GGCAAGGCCAACTCGGCCAAGTTCCTGCAGGACAATCCGGAAATCGGTAATGCCCTCG
AGAAGCAGATTCGCGACAAGCTGCTGGCTCCGAGCGGAGATACCAAGGCTCTGCCCGT
CAACGAGACCATCGATGACATGGCCGACGCGGATATCTGAATGAGCGAAGAAAACAC
GTACGACTCGAGCAGCATTAAAGTGCTGAAAGGTTTGGATGCCGTACGCAAACGTCCC
GGTATGTACATTGGTGACACCGACGATGGCAGCGGTCTGCACCATATGGTGTTCGAGG
TGGTCGACAACTCGATCGACGAAGCTCTGGCCGGCCACTGCGACGACATCAGCATCAT
CATCCACCCGGACGAATCCATTACCGTGCGTGACAACGGTCGCGGCATCCCGGTAGAC
GTGCATAAAGAAGAAGGCGTTTCCGCAGCCGAGGTCATCATGACCGTGCTGCACGCCG
GCGGTAAGTTCGACGACAACTCCTACAAGGTATCCGGCGGTCTGCACGGTGTGGGTGT
GTCGGTAGTGAATGCCCTGTCCGAAGAACTGGTGCTGACCGTTCGCCGCAGTGGCAAG
ATCTGGGAACAGACCTACGTTCACGGTGTGCCTCAGGCGCCTATGGCGATCGTCGGTG
ACAGCGAGACCACTGGTACCCAGATTCACTTCAAGGCTTCCAGCGAGACCTTCAAGAA
CATCCATTTCAGCTGGGACATCCTGGCCAAGCGGATTCGTGAACTGTCCTTCCTCAACT
CCGGTGTCGGTATCGTTCTGAAGGACGAGCGCAGCGGCAAGGAAGAACTGTTCAAATA
CGAAGGCGGCCTGCGCGCGTTCGTCGAATACCTGAACACCAACAAGACTGCGGTCAAC
CAGGTGTTCCACTTCAACGTGCAGCGTGAAGACGGCATCGGCGTGGAAATCGCCCTGC
AGTGGAACGACAGCTTCAACGAAAACCTGCAGTGCTTCACCAACAACATTCCGCAGCG
CGACGGCGGTACTCACCTGGTGGGCTTCCGCTCGGCACTGACGCGTAACCTGAACAAC
TACATCGAGCAGGAAGGTCTGGCGAAGAAGCACAAGGTCGCCACCACCGGTGACGAT
GCCCGCGAAGGCCTGACCGCGATCATTTCGGTCAAGGTGCCGGATCCGAAGTTCAGCT
CCCAGACCAAAGACAAGCTGGTGTCTTCCGAAGTAAAGACCGCGGTCGAACAGGAAA

TGGGCAAGTACTTCTCCGACTTCCTGCTGGAAAACCCGAACGAAGCCAAGCTGGTGGT
CGGCAAGATGCTCGACGCCGCCCGCGCCCGTGAAGCGGCGCGTAAGGCCCGTGAGATG
ACCCGCCGTAAAGGTGCGCTGGATATCGCCGGCCTGCCGGGCAAACTGGCGGACTGCC
AGGAAAAGGACCCTGCCCTTTCCGAACTCTACCTGGTGGAAGGTGACTCTGCTGGCGG
CTCCGCCAAGCAGGGACGCAACCGCAAGACCCAGGCGATTCTGCCGCTCAAGGGCAA
GATTCTTAACGTCGAGAAAGCGCGCTTCGACAAGATGATTTCCTCGCAAGAGGTCGGC
ACCTTGATCACTGCACTCGGCTGCGGCATCGGCCGCGAAGAGTACAACATCGACAAGC
TGCGTTATCACAACATCATCATCATGACCGACGCCGACGTCGACGGTTCGCACATCCGC
ACCCTGCTGCTGACTTTCTTCTTCCGTCAGCTGCCGGAGCTGATCGAGCGTGGCTACAT
CTACATCGCTCAACCGCCGCTGTACAAGGTCAAGAAAGGCAAGCAAGAGCAATACATC
AAAGACGACGACGCCATGGAAGAGTACATGACGCAGTCGGCCCTGGAAGATGCGAGC
CTGCACCTGAACGAAGAAGCACCGGGTATTTCCGGCGAGGCGCTGGAGCGCCTGGTGA
ACGACTTCCGCATGGTCATGAAAACCCTCAAGCGTCTGTCGCGCCTGTACCCTCAGGA
GCTGACCGAACACTTCATCTACCTGCCAGCCGTGAGCCTGGAGCAACTCTCCGATCAC
GCAGCGATGCAGGATTGGTTGGCCCAATATGAAGTCCGCCTGCGCACCGTCGAGAAGT
CCGGCCTGGTCTACAAGGCCAGCCTGCGTGAAGACCGTGAACGTAATGTCTGGCTGCC
AGAGGTCGAACTGATCTCCCACGGCCTGTCGAACTACGTCACCTTCAACCGCGACTTCT
TCGGCAGCAATGACTACAAGACCGTCGTCACCCTCGGCGCTCAACTGAGCTCCCTGCT
GGACGAAGGCGCTTATATTCAGCGCGGCGAACGCAAGAAGGCGGTGACCGAGTTCAA
GGAAGCCCTGGACTGGCTGATGACCGAAAGCACCAAGCGCCACACCATCCAGCGATAC
AAAGGTCTGGGCGAGATGAACCCGGACCAGCTGTGGGAAACCACCATGGACCCAAGC
GTGCGCCGCATGCTCAAGGTCACCATCGAAGACGCCATCGGCGCCGACCAGATCTTCA
ACACCCTGATGGGTGATGCGGTCGAGCCTCGTCGCGACTTCATCGAAAGCAACGCCCT
GGCGGTATCCAACCTGGACTTCTGAATGTCCGGAAAAGCGCAACAGCAGTCTCGCCTC
AAAGAGTTGATCAGCCGTGGTCGTGAGCAGGGTTACCTGACTTACGCGGAGGTCAACG
ACCACCTGCCGGAGGATATTTCAGATCCGGAACAGGTGGAAGACATCATCCGCATGAT
CAACGACATGGGGATCAACGTATTCGAGAGTGCTCCGGATGCGGATGCCCTTTTGTTG
GCCGAAGCCGATACCGACGAAGCAGCAGCTGAAGAAGCCGCCGCAGCGTTGGCGGCT
GTGGAAACCGACATTGGTCGCACTACCGACCCAGTGCGTATGTACATGCGCGAAATGG
GCACGGTAGAGCTGCTCACACGTGAAGGCGAAATCGAAATCGCCAAGCGTATCGAAG
AGGGCATCCGTGAAGTGATGGGCGCGATCGCGCACTTCCCTGGCACGGTTGAGCACAT
CCTCTCCGAATACACTCGCGTCACCACCGAAGGTGGCCGCCTGTCCGACGTCCTGAGC
GGTTACATCGACCCGGATGACGGCATTGCGCCGCCTGCCGCCGAAGTACCACCGCCTG
TCGATGCCAAGGCCGCGAAAGCGGACGACGACACCGACGACGATGACGCCGAAGCCA
GTGACGACGAAGAAGAAGCCGAAAGCGGTCCGGATCCGGTCATCGCAGCCCAGCGCT
TTGGCGCCGTTGCCGACCAGATGGAAATCACCCGCAAGGCGCTGAAAAAGCACGGTCG
TGAACACAAGCAAGCCCTGGCTGAAATGCTGGCCCTGGCTGAACTGTTCATGCCAATC
AAACTGGTTCCGAAGCAATTCGAAGGCCTGGTTGAACGTGTTCGTAGCGCCCTGGATC
GCCTGCGTCAGCAAGAGCGCGCGATCATGCAGCTCTGTGTTCGTGATGCCCGCATGCC
ACGCGCCGACTTCCTGCGCCAGTTCCCTGGCAATGAAGTGGACGAAAGCTGGTCCGAC
GCGCTGGCCAAAGGCAAGGCCAAGTACGCCGAAGCCATCGGCCGCCTGCAGCCGGAC
ATCATCCGTTGCCAGCAGAAGCTGACCGCGCTCGAGACCGAGACCGGCCTGACGATTG
CCGAGATCAAGGACATCAACCGTCGCATGTCGATCGGCGAGGCCAAGGCCCGTCGCGC
GAAGAAAGAGATGGTCGAAGCCAACTTGCGCCTGGTGATCTCCATCGCCAAGAAGTAC
ACCAACCGTGGCCTGCAGTTCCTCGACCTGATCCAGGAAGGCAACATCGGTTTGATGA
AAGCGGTAGACAAGTTCGAATACCGTCGCGGCTACAAGTTCTCGACTTATGCCACCTG
GTGGATCCGTCAGGCGATCACTCGCTCGATCGCCGACCAGGCCCGCACCATCCGTATTC
CGGTGCACATGATCGAGACGATCAACAAGCTCAACCGTATTTCCCGGCAGATGTTGCA
GGAAATGGGTCGCGAACCGACTCCGGAAGAGCTGGGCGAACGCATGGAAATGCCTGA
GGACAAGATCCGCAAGGTATTGAAGATCGCTAAAGAGCCGATCTCCATGGAAACCCCG
ATCGGTGATGACGAAGACTCCCATCTGGGTGACTTCATCGAAGACTCGACCATGCAGT

CGCCAATCGATGTCGCCACCGTTGAGAGCCTCAAGGAAGCGACTCGCGAAGTCCTCTC
CGGCCTCACTGCCCGTGAAGCCAAGGTACTGCGCATGCGCTTCGGCATCGACATGAAT
ACCGATCACACCCTTGAGGAAGTCGGTAAGCAGTTCGACGTTACCCGTGAGCGGATTC
GTCAGATCGAAGCCAAGGCGCTGCGCAAGCTGCGCCACCCGACGAGAAGCGAGCATC
TGCGCTCCTTCCTCGACGAGTAATTGACTAAGCCAGCCATACTCGCCCTTGCTGATGGC
AGCATTTTTCGCGGCGAAGCCATTGGAGCCGACGGTCAAACCGTTGGTGAGGTGGTGT
TTAACACCGCAATGACCGGCTATCAGGAAATCCTTACCGATCCTTCCTACGCCCAACAG
ATCGTTACCCTGACTTACCCGCACATCGGCAACACTGGCACCACGCCGGAAGACGCCG
AGTCCGATCGTGTCTGGTCGGCCGGTCTGGTGATTCGCGACCTGCCACTGGTTGCGAGC
AACTGGCGTAACACCCTGTCCCTGTCCGACTACCTGAAAGCCAACAATGTCGTGGCGA
TCGCCGGTATCGACACCCGTCGCCTGACGCGCATCCTGCGTGAAAAAGGCGCGCAGAA
CGGCTGCATCATGGCCGGCGACAATATCTCCGACGAAGCAGCGATTGCCGCAGCGCGC
GGCTTCCCTGGCCTGAAAGGCATGGATCTGGCGAAGGTCGTCAGCACCAAGGAAAGCT
ACGAGTGGCGCTCCAGCGTCTGGAGCCTGAAGACCGACAGTCATCCGACTATCGAAGC
TTCCGAGCTGCCTTACCACGTGGTTGCCTACGACTACGGCGTCAAGCTGAACATCCTGC
GCATGTTGGTCGAGCGCGGCTGCCGCGTGACCGTAGTGCCTGCGCAAACTCCGGCCAG
CGACGTCCTGGCACTCAAGCCTGACGGTGTGTTCCTGTCCAACGGTCCTGGCGACCCCG
AGCCTTGTGATTACGCCATCCAGGCGATCAAGGACGTGCTGGAAACCGAGATCCCGGT
CTTCGGTATCTGTCTGGGTCACCAATTGCTGGCTCTGGCCTCCGGTGCCAAGACAGTGA
AGATGGGCCACGGCCACCATGGCGCCAACCACCCGGTCCAGGACCTGGACAGCGGTGT
AGTGATGATCACCAGCCAAAACCACGGTTTTGCGGTGGACGAAACTACCCTGCCAGGC
AACGTGCGGGCGATCCACAAGTCGCTGTTCGATGGCACCCTGCAAGGCATCGAGCGTA
CCGACAAGAGCGCATTCAGCTTCCAGGGCCACCCTGAAGCGAGCCCGGGCCCGAACGA
TGTGGCGCCGCTGTTCGATCGTTTCATCAACGAGATGGCCAAGCGACGCTGAATGAGT
AGCGGACGTATCGTTCAAATCATCGGCGCCGTTATCGACGTGGAATTTCCACGCGACA
GCGTACCGAGCATCTACGACGCCTTGAAGGTTCAAGGCGCCGAAACCACTCTGGAAGT
TCAGCAGCAGCTGGGCGACGGCGTGGTACGTACCATTGCGATGGGCTCCACCGAGGGC
TTGAAGCGCGGTCTGGACGTCAACAACACTGGCGCAGCCATCTCCGTACCGGTCGGTA
AAGCGACCCTGGGCCGGATCATGGACGTACTGGGCAACCCGATCGACGAAGCTGGCCC
GATCGGCGAAGAAGAGCGTTGGGGCATTCACCGTCCTGCGCCGACCTTCGCTGAACAA
GCTGGCGGCAACGACCTCCTGGAAACCGGCATCAAGGTTATCGACCTGGTTTGCCCGT
TCGCCAAGGGCGGTAAAGTCGGTCTGTTCGGTGGTGCCGGTGTGGGCAAAACCGTAAA
CATGATGGAACTGATCCGTAACATCGCCATCGAGCACAGCGGTTATTCCGTGTTCGCCG
GTGTGGGTGAGCGTACTCGTGAGGGTAACGACTTCTACCACGAGATGAAGGATTCCAA
CGTTCTGGACAAAGTGGCACTGGTATACGGCCAGATGAACGAGCCGCCGGGAAACCGT
CTGCGCGTAGCTCTGACCGGCCTGACCATGGCCGAGAAGTTCCGTGACGAAGGTAACG
ACGTTCTGCTGTTCGTCGACAACATCTATCGTTACACCCTGGCCGGTACCGAAGTATCC
GCACTGCTGGGCCGTATGCCTTCGGCAGTAGGTTACCAGCCGACCCTGGCTGAAGAGA
TGGGCGTTCTGCAAGAACGTATCACTTCGACCAAGCAAGGCTCGATCACCTCGATCCA
AGCGGTATACGTACCTGCGGACGACTTGACCGACCCGTCGCCAGCGACCACCTTCGCC
CACTTGGACGCCACCGTCGTACTGTCCCGTGACATCGCTTCCCTGGGTATCTACCCAGC
GGTAGACCCACTGGATTCGACTTCCCGTCAGCTGGACCCGAACGTGATCGGCAACGAG
CACTACGAAACCGCTCGCGGCGTTCAGTACGTGCTGCAGCGCTACAAAGAGCTGAAGG
ACATCATTGCGATCCTGGGTATGGACGAACTGTCCGAAGCCGACAAGCAACTGGTATC
CCGCGCTCGTAAGATCCAGCGCTTCCTGTCGCAGCCGTTCTTCGTGGCTGAAGTCTTCA
CTGGTTCTCCAGGCAAATACGTTTCCCTGAAAGACACCATCGCTGGCTTCAAAGGCATC
CTCAACGGTGACTACGACCACCTGCCAGAACAAGCGTTCTACATGGTCGGCGGCATCG
AAGAAGCGATCGAGAAAGCCAAGAAACTGTAA

NCBI Reference Sequence: NZ_CP027748.1
Strain: ChPhzS23

Chained genes: 16S rRNA, recA, gyrB, rpoD, carA, atpD
>NZ_CP027748_ChPhzS23
GAACTGAAGAGTTTGATCATGGCTCAGATTGAACGCTGGCGGCAGGCCTAACACATGC
AAGTCGAGCGGTAGAGAGAAGCTTGCTTCTCTTGAGAGCGGCGGACGGGTGAGTAATG
CCTAGGAATCTGCCTGGTAGTGGGGGATAACGTTCGGAAACGGACGCTAATACCGCAT
ACGTCCTACGGGAGAAAGCAGGGGACCTTCGGGCCTTGCGCTATCAGATGAGCCTAGG
TCGGATTAGCTAGTTGGTGAGGTAATGGCTCACCAAGGCGACGATCCGTAACTGGTCT
GAGAGGATGATCAGTCACACTGGAACTGAGACACGGTCCAGACTCCTACGGGAGGCA
GCAGTGGGGAATATTGGACAATGGGCGAAAGCCTGATCCAGCCATGCCGCGTGTGTGA
AGAAGGTCTTCGGATTGTAAAGCACTTTAAGTTGGGAGGAAGGGTACTTACCTAATAC
GTGAGTATTTTGACGTTACCGACAGAATAAGCACCGGCTAACTCTGTGCCAGCAGCCG
CGGTAATACAGAGGGTGCAAGCGTTAATCGGAATTACTGGGCGTAAAGCGCGCGTAGG
TGGTTCGTTAAGTTGGATGTGAAATCCCCGGGCTCAACCTGGGAACTGCATCCAAAAC
TGGCGAGCTAGAGTATGGTAGAGGGTGGTGGAATTTCCTGTGTAGCGGTGAAATGCGT
AGATATAGGAAGGAACACCAGTGGCGAAGGCGACCACCTGGACTGATACTGACACTG
AGGTGCGAAAGCGTGGGGAGCAAACAGGATTAGATACCCTGGTAGTCCACGCCGTAA
ACGATGTCAACTAGCCGTTGGGAGCCTTGAGCTCTTAGTGGCGCAGCTAACGCATTAA
GTTGACCGCCTGGGGAGTACGGCCGCAAGGTTAAAACTCAAATGAATTGACGGGGGCC
CGCACAAGCGGTGGAGCATGTGGTTTAATTCGAAGCAACGCGAAGAACCTTACCAGGC
CTTGACATCCAATGAACTTTCCAGAGATGGATTGGTGCCTTCGGGAACATTGAGACAG
GTGCTGCATGGCTGTCGTCAGCTCGTGTCGTGAGATGTTGGGTTAAGTCCCGTAACGAG
CGCAACCCTTGTCCTTAGTTACCAGCACGTCATGGTGGGCACTCTAAGGAGACTGCCG
GTGACAAACCGGAGGAAGGTGGGGATGACGTCAAGTCATCATGGCCCTTACGGCCTGG
GCTACACACGTGCTACAATGGTCGGTACAGAGGGTTGCCAAGCCGCGAGGTGGAGCTA
ATCCCATAAAACCGATCGTAGTCCGGATCGCAGTCTGCAACTCGACTGCGTGAAGTCG
GAATCGCTAGTAATCGCGAATCAGAATGTCGCGGTGAATACGTTCCCGGGCCTTGTAC
ACACCGCCCGTCACACCATGGGAGTGGGTTGCACCAGAAGTAGCTAGTCTAACCTTCG
GGAGGACGGTTACCACGGTGTGATTCATGACTGGGGTGAAGTCGTAACAAGGTAGCCG
TAGGGGAACCTGCGGCTGGATCACCTCCTTAATATGGACGACAACAAGAAGAAAGCCT
TGGCTGCGGCCCTGGGTCAGATCGAACGTCAATTCGGCAAGGGTGCCGTAATGCGTAT
GGGCGATCACGACCGCCAGGCGATCCCGGCCATTTCCACTGGCTCTCTGGGTCTGGAC
ATCGCACTCGGCATCGGCGGCCTGCCAAAAGGTCGTATTGTTGAAATCTACGGTCCGG
AATCGTCCGGTAAAACCACCCTGACCCTGTCCGTGATTGCCCAGGCACAGAAGATGGG
CGCCACCTGCGCCTTCGTCGACGCCGAGCACGCACTGGACCCGGAATACGCCGGCAAA
CTGGGGGTCAACGTTGACGACCTGCTGGTTTCCCAGCCGGACACCGGCGAACAGGCGC
TGGAAATCACCGACATGCTGGTGCGCTCCAATGCCATCGACGTGATCGTGATCGACTC
CGTGGCGGCACTGGTACCCAAGGCCGAGATCGAAGGCGAGATGGGCGACATGCACGT
GGGCCTGCAGGCCCGCCTGATGTCCCAGGCGCTGCGCAAGATCACCGGTAACATCAAG
AACGCCAACTGCCTGGTGATCTTCATCAACCAGATCCGTATGAAAATCGGCGTGATGTT
CGGCAGCCCGGAAACCACCACCGGCGGTAACGCGCTGAAGTTCTACGCTTCGGTTCGT
CTGGACATCCGTCGTACTGGCGCGGTGAAGGAAGGCGACGAAGTCGTCGGTAGCGAA
ACCCGGGTCAAGATCGTCAAGAACAAGGTGGCTCCACCGTTCCGCCAGGCTGAATTCC
AGATCCTGTACGGCAAGGGTATCTACCTGAACGGCGAGATCATCGATCTGGGCGTGCT
GCACGGTTTCCTCGAGAAGTCCGGTGCCTGGTACAGCTACCAGGGCAACAAGATCGGT
CAGGGCAAGGCCAACTCGGCCAAGTTCCTGCAGGACAATCCGGAAATCGGCAATGCCC
TCGAGAAGCAGATTCGCGACAAGCTGCTGGCTCCAAGCGCTGATGTCAAAGCTTCGCC
GGTCAACGAGACCATCGATGACATGGCTGACGCGGATATCTGAATGAGCGAAGAAAA
CACGTACGACTCGAGCAGCATTAAAGTGCTGAAAGGTTTGGATGCCGTACGCAAACGT
CCCGGTATGTACATTGGTGACACCGACGATGGCAGCGGTCTGCACCATATGGTGTTCG
AGGTGGTCGATAACTCGATCGACGAAGCTCTGGCCGGCCATTGCGACGACATCAGCAT
CATCATCCACCCGGACGAATCCATTACCGTGCGTGACAACGGTCGCGGCATCCCGGTA

GACGTGCATAAAGAAGAAGGCGTTTCCGCGGCCGAGGTCATCATGACCGTACTGCACG
CCGGCGGTAAGTTCGACGATAACTCCTACAAAGTATCCGGCGGTCTGCACGGTGTGGG
TGTGTCGGTAGTGAACGCCCTGTCCGAAGAACTGGTCCTGACCGTTCGCCGCAGCGGA
AAGATCTGGGAACAGACCTACGTTCACGGTGTGCCTCAGGCGCCTATGGCGATCGTCG
GTGACAGCGAAACCACCGGTACCCAGATTCACTTCAAGGCGTCCAGCGAGACCTTCAA
GAACATCCATTTCAGCTGGGACATCCTGGCCAAGCGGATTCGTGAACTGTCCTTCCTCA
ACTCCGGTGTCGGTATCGTTCTGAAGGACGAACGCAGTGGCAAGGAAGAGCTGTTCAA
GTACGAAGGCGGCCTGCGTGCGTTCGTTGAATACCTGAACACCAACAAGACCGCGGTC
AACCAGGTGTTCCACTTCAATGTGCAGCGTGAAGATGGCATCGGCGTGGAAATCGCCC
TGCAGTGGAACGACAGCTTCAACGAAAACCTGCAGTGCTTCACCAACAACATTCCGCA
GCGCGATGGCGGCACCCACTTGGTGGGCTTCCGTTCGGCACTGACGCGTAACCTGAAC
AACTACATCGAACAGGAAGGTCTGGCGAAGAAGCACAAGGTCGCCACCACCGGTGAC
GATGCCCGCGAAGGCCTGACCGCGATCATTTCGGTCAAGGTGCCGGATCCGAAGTTCA
GCTCCCAGACCAAAGACAAGCTGGTGTCTTCCGAAGTGAAGACCGCGGTTGAACAGGA
AATGGGCAAGTACTTCTCCGACTTCCTGCTGGAAAACCCGAACGAAGCCAAGCTGGTG
GTCGGCAAGATGCTCGACGCCGCCCGTGCCCGTGAAGCGGCGCGTAAGGCTCGTGAGA
TGACCCGCCGTAAAGGCGCGCTGGATATCGCCGGCCTGCCGGGCAAACTGGCGGACTG
CCAGGAAAAAGACCCTGCCCTTTCCGAACTCTACTTGGTGGAAGGTGACTCTGCTGGC
GGCTCCGCCAAGCAGGGACGCAACCGTAAGACCCAGGCGATTCTGCCGCTCAAGGGCA
AGATCCTTAACGTCGAGAAAGCGCGCTTCGACAAGATGATTTCCTCGCAAGAGGTCGG
CACCTTGATCACTGCACTCGGTTGCGGCATCGGCCGCGAAGAGTACAACATCGACAAG
CTGCGTTATCACAACATCATCATCATGACCGACGCTGACGTCGACGGTTCGCACATCCG
TACCCTGCTGCTGACCTTCTTCTTCCGTCAGCTGCCGGAGCTGATCGAGCGTGGCTACA
TCTACATCGCTCAACCGCCGCTGTACAAGGTCAAGAAAGGCAAGCAAGAGCAATACAT
CAAAGACGACGACGCCATGGAAGAGTACATGACGCAGTCGGCCCTGGAAGATGCGAG
CCTGCACCTGAACGAAGAAGCACCGGGTATTTCCGGCGAGGCGCTGGAGCGCCTGGTG
AACGACTTCCGCATGGTCATGAAAACCCTCAAGCGTCTGTCGCGCCTGTACCCTCAGG
AGCTGACCGAGCACTTCATCTACCTGCCGGCCGTGAGCCTGGAGCAACTCTCCGATCA
CGCGGCCATGCAGGATTGGCTGGCCCAATATGAAGTCCGCCTGCGCACCGTCGAGAAG
TCCGGCCTGGTCTACAAGGCCAGCCTGCGTGAAGACCGTGAACGTAATGTCTGGCTGC
CAGAGGTCGAACTGATCTCCCACGGCCTGTCGAACTACGTCACCTTCAACCGCGACTTC
TTCGGTAGCAATGACTACAAGACCGTCGTTACCCTCGGCGCTCAACTGAGCTCCCTGCT
GGACGAAGGCGCTTATATTCAGCGTGGCGAACGCAAGAAGGCGGTGACCGAGTTCAA
GGAAGCCCTGGACTGGCTGATGACCGAAAGCACCAAGCGCCACACCATCCAGCGATAC
AAAGGTCTGGGCGAGATGAACCCGGATCAGCTGTGGGAAACCACCATGGACCCAAGC
GTGCGCCGTATGCTCAAGGTCACGATTGAAGATGCCATCGGCGCCGACCAGATCTTCA
ACACCCTGATGGGGGATGCGGTCGAGCCTCGTCGCGACTTCATCGAAAGCAACGCCCT
GGCGGTATCCAATCTGGACTTCTGAATGTCCGGAAAAGCGCAACAGCAGTCTCGCCTC
AAAGAGTTGATCAGCCGTGGTCGTGAGCAGGGTTACCTGACTTACGCGGAGGTCAACG
ACCACCTGCCGGAGGATATTTCAGATCCGGAACAGGTGGAAGACATCATCCGCATGAT
CAACGACATGGGGATCAACGTATTCGAGAGTGCTCCGGATGCGGATGCCCTTTTGTTG
GCCGAAGCCGATACCGACGAAGCAGCAGCTGAAGAAGCCGCCGCAGCGTTGGCGGCC
GTGGAAACCGACATTGGTCGCACTACCGACCCCGTGCGTATGTACATGCGCGAAATGG
GAACGGTAGAGCTGCTCACACGTGAAGGCGAAATCGAAATCGCCAAGCGTATCGAAG
AGGGCATCCGTGAAGTGATGGGCGCGATCGCGCACTTCCCTGGCACGGTTGAGCACAT
CCTCTCCGAATACACTCGCGTCACCACCGAAGGTGGCCGCCTGTCCGACGTCCTGAGC
GGTTACATCGACCCGGACGACGGCATCGCGCCGCCTGCCGCCGAAGTACCACCGCCTG
TCGATGCCAAGGCCGCAAAAGCGGACGACGACACCGACGACGATGACGCCGAAGCCA
GTGACGACGAAGAAGAAGCCGAAAGCGGTCCGGATCCGGTCATCGCAGCCCAGCGCT
TTGGCGCCGTTGCCGACCAGATGGAAATCACCCGCAAGGCGCTGAAGAAGCACGGTCG
CGAACACAAGCAAGCCCTGGCTGAAATGCTGGCCCTGGCTGAACTGTTCATGCCGATC

AAACTGGTTCCGAAGCAATTCGAAGGCCTGGTTGAACGTGTTCGTAGCGCCCTGGATC
GCCTGCGTCAGCAAGAGCGCGCGATCATGCAGCTCTGTGTTCGTGATGCCCGCATGCC
ACGCGCCGACTTCCTGCGCCAGTTCCCTGGCAATGAAGTGGACGAAAGCTGGTCCGAC
GCGCTGGCCAAAGGCAAGGCCAAGTACGCCGAAGCCATCGGCCGCCTGCAGCCGGAC
ATTATCCGTTGCCAGCAGAAGCTGACCGCGCTTGAGACCGAGACCGGCCTGACGATCG
CCGAGATCAAGGACATCAACCGTCGCATGTCGATCGGCGAGGCCAAGGCCCGTCGCGC
GAAGAAAGAGATGGTCGAAGCCAACCTGCGTCTGGTGATCTCCATCGCCAAGAAGTAC
ACCAACCGTGGCTTGCAATTCCTCGACCTGATCCAGGAAGGCAACATCGGTTTGATGA
AAGCGGTAGACAAGTTCGAATACCGTCGCGGCTACAAATTCTCGACTTATGCCACCTG
GTGGATCCGTCAGGCGATCACTCGCTCGATCGCCGACCAGGCCCGCACCATCCGTATTC
CGGTGCACATGATCGAGACGATCAACAAGCTCAACCGTATTTCCCGGCAGATGTTGCA
GGAAATGGGTCGCGAACCGACTCCGGAAGAGCTGGGCGAACGCATGGAAATGCCTGA
GGACAAGATCCGCAAGGTATTGAAGATCGCTAAAGAGCCGATCTCCATGGAAACCCCG
ATCGGTGATGACGAAGACTCCCATCTGGGTGACTTCATCGAAGACTCGACCATGCAGT
CGCCAATCGATGTCGCCACCGTCGAGAGTCTTAAAGAAGCGACTCGCGAAGTACTCTC
CGGCCTCACTGCCCGTGAAGCCAAGGTACTGCGCATGCGCTTCGGCATCGACATGAAT
ACCGACCACACCCTCGAGGAAGTCGGTAAGCAGTTTGACGTTACCCGTGAGCGGATTC
GTCAGATCGAAGCCAAGGCGCTGCGCAAGCTGCGCCACCCGACGCGAAGCGAGCACC
TGCGCTCCTTCCTCGACGAGTAATTGACTAAGCCAGCCATACTCGCCCTTGCTGATGGC
AGCATTTTTCGCGGCGAAGCCATTGGAGCCGACGGTCAAACCGTTGGTGAGGTGGTGT
TTAACACCGCAATGACCGGCTATCAGGAAATCCTTACCGATCCTTCCTACGCCCAACAG
ATCGTTACCCTGACTTACCCGCATATCGGCAATACCGGCACCACGCCGGAAGACGCCG
AGTCCGATCGTGTCTGGTCGGCCGGTCTGGTGATTCGCGACCTGCCTCTGGTTGCGAGC
AACTGGCGTAACACCCTGTCCCTGTCCGACTACCTGAAAGCCAACAATGTCGTGGCAA
TCGCCGGTATCGACACCCGTCGCCTGACGCGCATCCTGCGCGAGAAAGGTGCGCAGAA
CGGCTGCATCATGGCCGGCGACAATATCTCCGACGAAGCGGCGATTGCCGCTGCACGC
GGCTTCCCGGGCCTGAAAGGCATGGATCTGGCGAAGGTCGTCAGCACCAAGGAAAGCT
ACGAGTGGCGCTCCAGTGTCTGGAACCTGAAGACCGACAGTCATCCGACCATCGAAGC
TTCCGAGCTGCCTTACCACGTGGTTGCCTACGACTACGGCGTCAAGCTGAACATCCTGC
GCATGCTGGTCGAACGCGGTTGCCGCGTGACCGTGGTGCCTGCGCAAACCCCGGCCAG
CGAAGCGCTGGCGCTCAAGCCTGACGGTGTGTTCCTGTCCAACGGCCCTGGCGACCCC
GAGCCTTGCGATTACGCCATCCAGGCGATCAAGGACGTGCTGGAGACCGAGATTCCGG
TCTTCGGTATCTGTCTGGGCCACCAACTGCTGGCACTGGCCGCCGGCGCCAAGACAGT
GAAGATGGGCCACGGCCACCACGGCGCCAACCACCCGGTCCAGGACCTGGACAGCGG
TGTGGTGATGATCACCAGCCAGAACCACGGTTTTGCGGTGGACGAAGCCACCCTGCCG
GGCAACGTGCGGGCGATCCACAAGTCGCTGTTCGACGGCACCCTGCAAGGCATCGAGC
TGATCGACAAGAGCGCATTCAGCTTCCAGGGCCACCCTGAAGCGAGCCCGGGCCCGAA
CGATGTGGCGCCGCTGTTCGATCGTTTCATCAACGAGATGGCCAAGCGACGCTGAATG
AGTAGCGGACGTATCGTTCAAATCATCGGCGCCGTTATCGACGTGGAATTTCCACGCG
ACAGCGTACCGAGCATCTACGACGCCTTGAAGGTTCAAGGCGCCGAAACCACTCTGGA
AGTTCAGCAGCAGCTGGGCGACGGCGTGGTACGTACCATTGCGATGGGCTCCACCGAG
GGCTTGAAGCGCGGTCTGGACGTCAACAACACTGGCGCAGCCATCTCCGTACCGGTCG
GTAAAGCGACCCTGGGCCGGATCATGGACGTACTGGGCAACCCGATCGACGAAGCTGG
CCCGATCGGCGAAGAAGAGCGTTGGGGCATTCACCGTCCTGCGCCGACCTTCGCTGAA
CAAGCTGGCGGCAACGACCTGCTGGAAACCGGCATCAAGGTTATCGACCTGGTTTGCC
CGTTCGCCAAGGGCGGTAAAGTCGGTCTGTTCGGTGGTGCCGGTGTGGGCAAAACCGT
AAACATGATGGAACTGATCCGTAACATCGCCATCGAGCACAGCGGTTATTCCGTGTTC
GCCGGTGTGGGTGAGCGTACTCGTGAGGGTAACGACTTCTACCACGAGATGAAGGACT
CCAACGTTCTGGACAAAGTGGCACTGGTATACGGCCAGATGAACGAGCCGCCGGGAA
ACCGTCTGCGCGTAGCTCTGACCGGCCTGACCATGGCCGAGAAGTTCCGTGACGAAGG
TAACGACGTTCTGCTGTTCGTCGACAACATCTATCGTTACACCCTGGCCGGTACCGAAG

TATCCGCACTGCTGGGCCGTATGCCTTCGGCAGTAGGTTACCAGCCGACCCTGGCTGAA
GAAATGGGCGTTCTGCAAGAACGTATCACTTCGACCAAGCAAGGCTCGATCACCTCGA
TCCAAGCGGTATACGTACCTGCGGACGACTTGACCGACCCGTCGCCAGCGACCACCTT
CGCCCACTTGGACGCCACCGTCGTTCTGTCCCGTGACATCGCTTCCCTGGGTATCTACC
CAGCGGTAGACCCACTGGACTCGACTTCCCGTCAGCTGGACCCGAACGTGATCGGCAC
CGAGCACTACGAAACCGCTCGTGGCGTTCAGTACGTGCTGCAGCGCTACAAAGAGCTG
AAGGACATCATTGCGATCCTGGGTATGGACGAACTGTCCGAAGCCGACAAGCAACTGG
TATCCCGCGCTCGTAAGATCCAGCGCTTCCTGTCGCAGCCGTTCTTCGTGGCTGAAGTC
TTCACTGGTTCTCCAGGCAAATACGTTTCCCTGAAAGACACCATCGCTGGCTTCAAAGG
CATCCTCAACGGTGACTACGACCATCTGCCAGAACAAGCGTTCTACATGGTTGGTGGC
ATCGAAGAAGCGATCGAGAAAGCCAAGAAACTGTAA

NCBI Reference Sequence: NZ_CP027720.1
Strain: DSM6698
Chained genes: 16S rRNA, recA, gyrB, rpoD, carA, atpD
>NZ_CP027720_DSM6698
GAACTGAAGAGTTTGATCATGGCTCAGATTGAACGCTGGCGGCAGGCCTAACACATGC
AAGTCGAGCGGTAGAGAGAAGCTTGCTTCTCTTGAGAGCGGCGGACGGGTGAGTAATG
CCTAGGAATCTGCCTGGTAGTGGGGGATAACGTTCGGAAACGGACGCTAATACCGCAT
ACGTCCTACGGGAGAAAGCAGGGGACCTTCGGGCCTTGCGCTATCAGATGAGCCTAGG
TCGGATTAGCTAGTTGGTGAGGTAATGGCTCACCAAGGCGACGATCCGTAACTGGTCT
GAGAGGATGATCAGTCACACTGGAACTGAGACACGGTCCAGACTCCTACGGGAGGCA
GCAGTGGGGAATATTGGACAATGGGCGAAAGCCTGATCCAGCCATGCCGCGTGTGTGA
AGAAGGTCTTCGGATTGTAAAGCACTTTAAGTTGGGAGGAAGGGTACTTACCTAATAC
GTGAGTATTTTGACGTTACCGACAGAATAAGCACCGGCTAACTCTGTGCCAGCAGCCG
CGGTAATACAGAGGGTGCAAGCGTTAATCGGAATTACTGGGCGTAAAGCGCGCGTAGG
TGGTTCGTTAAGTTGGATGTGAAATCCCCGGGCTCAACCTGGGAACTGCATCCAAAAC
TGGCGAGCTAGAGTATGGTAGAGGGTGGTGGAATTTCCTGTGTAGCGGTGAAATGCGT
AGATATAGGAAGGAACACCAGTGGCGAAGGCGACCACCTGGACTGATACTGACACTG
AGGTGCGAAAGCGTGGGGAGCAAACAGGATTAGATACCCTGGTAGTCCACGCCGTAA
ACGATGTCAACTAGCCGTTGGGAGCCTTGAGCTCTTAGTGGCGCAGCTAACGCATTAA
GTTGACCGCCTGGGGAGTACGGCCGCAAGGTTAAAACTCAAATGAATTGACGGGGGCC
CGCACAAGCGGTGGAGCATGTGGTTTAATTCGAAGCAACGCGAAGAACCTTACCAGGC
CTTGACATCCAATGAACTTTCCAGAGATGGATTGGTGCCTTCGGGAACATTGAGACAG
GTGCTGCATGGCTGTCGTCAGCTCGTGTCGTGAGATGTTGGGTTAAGTCCCGTAACGAG
CGCAACCCTTGTCCTTAGTTACCAGCACGTCATGGTGGGCACTCTAAGGAGACTGCCG
GTGACAAACCGGAGGAAGGTGGGGATGACGTCAAGTCATCATGGCCCTTACGGCCTGG
GCTACACACGTGCTACAATGGTCGGTACAGAGGGTTGCCAAGCCGCGAGGTGGAGCTA
ATCCCATAAAACCGATCGTAGTCCGGATCGCAGTCTGCAACTCGACTGCGTGAAGTCG
GAATCGCTAGTAATCGCGAATCAGAATGTCGCGGTGAATACGTTCCCGGGCCTTGTAC
ACACCGCCCGTCACACCATGGGAGTGGGTTGCACCAGAAGTAGCTAGTCTAACCTTCG
GGAGGACGGTTACCACGGTGTGATTCATGACTGGGGTGAAGTCGTAACAAGGTAGCCG
TAGGGGAACCTGCGGCTGGATCACCTCCTTAAATGGACGACAACAAGAAGAAAGCCTT
GGCTGCGGCCCTGGGTCAGATCGAACGTCAATTCGGCAAGGGTGCCGTAATGCGTATG
GGCGATCACGACCGCCAGGCGATCCCGGCCATTTCCACTGGCTCTCTGGGTCTGGACAT
CGCACTCGGCATCGGCGGCCTGCCAAAAGGTCGTATTGTTGAAATCTACGGTCCGGAA
TCGTCCGGTAAAACCACCCTGACCCTGTCCGTGATTGCCCAGGCACAGAAGATGGGCG
CCACCTGCGCCTTCGTCGACGCCGAGCACGCACTGGACCCGGAATACGCCGGCAAACT
GGGGGTCAACGTTGACGACCTGCTGGTTTCCCAGCCGGACACCGGCGAACAGGCGCTG
GAAATCACCGACATGCTGGTGCGCTCCAATGCCATCGACGTGATCGTGATCGACTCCG
TGGCGGCACTGGTACCCAAGGCCGAGATCGAAGGCGAGATGGGCGACATGCACGTGG

GCCTGCAGGCCCGCCTGATGTCCCAGGCACTGCGCAAGATCACCGGTAACATCAAGAA
CGCCAACTGCCTGGTGATCTTCATCAACCAGATCCGTATGAAAATCGGCGTGATGTTCG
GCAGCCCGGAAACCACCACCGGCGGTAACGCGCTGAAGTTCTACGCTTCGGTTCGTCT
GGACATCCGTCGTACTGGCGCGGTGAAGGAAGGCGACGAAGTCGTCGGTAGCGAAAC
CCGGGTCAAGATCGTCAAGAACAAGGTGGCTCCACCGTTCCGCCAGGCTGAATTCCAG
ATCCTGTACGGCAAGGGTATCTACCTGAACGGCGAGATCATCGATCTGGGCGTGCTGC
ACGGTTTCCTCGAGAAGTCCGGTGCCTGGTACAGCTACCAGGGCAACAAGATCGGTCA
GGGCAAGGCCAACTCGGCCAAGTTCCTGCAGGACAATCCGGAAATCGGCAATGCCCTC
GAGAAGCAGATTCGCGACAAGCTGCTGGCTCCAACCGCTGATGTCAAAGCTTCGCCGG
TCAACGAGACCATCGATGACATGGCTGACGCGGATATCTGAATGAGCGAAGAAAACA
CGTACGACTCGAGCAGCATTAAAGTGCTGAAAGGTTTGGATGCCGTACGCAAACGTCC
CGGTATGTACATTGGTGACACCGACGATGGCAGCGGTCTGCACCATATGGTGTTCGAG
GTGGTCGATAACTCGATCGACGAAGCTCTGGCCGGCCATTGCGACGACATCAGCATCA
TCATCCACCCGGACGAATCCATTACCGTGCGTGACAACGGTCGCGGCATCCCGGTAGA
CGTGCATAAAGAAGAAGGCGTTTCCGCGGCCGAGGTCATCATGACCGTACTGCACGCC
GGCGGTAAGTTCGACGATAACTCCTACAAAGTATCCGGCGGTCTGCACGGTGTGGGTG
TGTCGGTAGTGAACGCCCTGTCCGAAGAACTGGTCCTGACCGTTCGCCGCAGCGGAAA
GATCTGGGAACAGACCTACGTTCACGGTGTGCCTCAGGCGCCTATGGCGATCGTCGGT
GACAGCGAAACCACCGGTACCCAGATTCACTTCAAGGCGTCCAGCGAGACCTTCAAGA
ACATCCATTTCAGCTGGGACATCCTGGCCAAGCGGATTCGTGAACTGTCCTTCCTCAAC
TCCGGTGTCGGTATCGTTCTGAAGGACGAACGCAGTGGCAAGGAAGAGCTGTTCAAGT
ACGAAGGCGGCCTGCGTGCGTTCGTTGAATACCTGAACACCAACAAGACCGCGGTCAA
CCAGGTGTTCCACTTCAATGTGCAGCGTGAAGATGGCATCGGCGTGGAAATCGCCCTG
CAGTGGAACGACAGCTTCAACGAAAACCTGCAGTGCTTCACCAACAACATTCCGCAGC
GCGATGGCGGCACCCACTTGGTGGGCTTCCGTTCGGCACTGACGCGTAACCTGAACAA
CTACATCGAACAGGAAGGTCTGGCGAAGAAGCACAAGGTCGCCACCACCGGTGACGA
TGCCCGCGAAGGCCTGACCGCGATCATTTCGGTCAAGGTGCCGGATCCGAAGTTCAGC
TCCCAGACCAAAGACAAGCTGGTGTCTTCCGAAGTGAAGACCGCGGTTGAACAGGAAA
TGGGCAAGTACTTCTCCGACTTCCTGCTGGAAAACCCGAACGAAGCCAAGCTGGTGGT
CGGCAAGATGCTCGACGCCGCCCGTGCCCGTGAAGCGGCGCGTAAGGCTCGTGAGATG
ACCCGCCGTAAAGGCGCGCTGGATATCGCCGGCCTGCCGGGCAAACTGGCGGACTGCC
AGGAAAAAGACCCTGCCCTTTCCGAACTCTACCTGGTGGAAGGTGACTCTGCTGGCGG
CTCCGCCAAGCAGGGACGCAACCGTAAGACCCAGGCGATTCTGCCGCTCAAGGGCAAG
ATCCTTAACGTCGAGAAAGCGCGCTTCGACAAGATGATTTCCTCGCAAGAGGTCGGCA
CCTTGATCACTGCACTCGGTTGCGGCATCGGCCGCGAAGAGTACAACATCGACAAGCT
GCGTTATCACAACATCATCATCATGACCGACGCTGACGTCGACGGTTCGCACATCCGTA
CCCTGCTGCTGACCTTCTTCTTCCGTCAGCTGCCGGAGCTGATCGAGCGTGGCTACATC
TACATCGCTCAACCGCCGCTGTACAAGGTCAAGAAAGGCAAGCAAGAGCAATACATCA
AAGACGACGACGCCATGGAAGAGTACATGACGCAGTCGGCCCTGGAAGATGCGAGCC
TGCACCTGAACGAAGAAGCACCGGGTATTTCCGGCGAGGCGCTGGAGCGCCTGGTGAA
CGACTTCCGCATGGTCATGAAAACCCTCAAGCGTCTGTCGCGCCTGTACCCTCAGGAGC
TGACCGAGCACTTCATCTACCTGCCGGCCGTGAGCCTGGAGCAACTCTCCGATCACGC
GGCCATGCAGGATTGGCTGGCCCAATATGAAGTCCGCCTGCGCACCGTCGAGAAGTCC
GGCCTGGTCTACAAGGCCAGCCTGCGTGAAGACCGTGAACGTAATGTCTGGCTGCCAG
AGGTCGAACTGATCTCCCACGGCCTGTCGAACTACGTCACCTTCAACCGCGACTTCTTC
GGTAGCAATGACTACAAGACCGTCGTTACCCTCGGCGCTCAACTGAGCTCCCTGCTGG
ACGAAGGCGCTTATATTCAGCGTGGCGAACGCAAGAAGGCGGTGACCGAGTTCAAGG
AAGCCCTGGACTGGCTGATGACCGAAAGCACCAAGCGCCACACCATCCAGCGATACAA
AGGTCTGGGCGAGATGAACCCGGATCAGCTGTGGGAAACCACCATGGACCCAAGCGT
GCGCCGTATGCTCAAGGTCACGATTGAAGATGCCATCGGCGCCGACCAGATCTTCAAC
ACCCTGATGGGGGATGCGGTCGAGCCTCGTCGCGACTTCATCGAAAGCAACGCCCTGG

CGGTATCCAATCTGGACTTCTGAATGTCCGGAAAAGCGCAACAGCAGTCTCGCCTCAA
AGAGTTGATCAGCCGTGGTCGTGAGCAGGGTTACCTGACTTACGCGGAGGTCAACGAC
CACCTGCCGGAGGATATTTCAGATCCGGAACAGGTGGAAGACATCATCCGCATGATCA
ACGACATGGGGATCAACGTATTCGAGAGTGCTCCGGATGCGGATGCCCTTTTGTTGGC
CGAAGCCGATACCGACGAAGCAGCAGCTGAAGAAGCCGCCGCAGCGTTGGCGGCTGT
GGAAACCGACATTGGTCGCACTACCGACCCCGTGCGTATGTACATGCGCGAAATGGGA
ACGGTAGAGCTGCTCACACGTGAAGGCGAAATCGAAATCGCCAAGCGTATCGAAGAG
GGCATCCGTGAAGTGATGGGCGCGATCGCGCACTTCCCTGGCACGGTTGAGCACATCC
TCTCCGAATACACTCGCGTCACCACCGAAGGTGGCCGCCTGTCCGACGTCCTGAGCGG
TTACATCGACCCGGACGACGGCATTGCGCCGCCTGCCGCCGAAGTACCACCGCCTGTC
GATGCCAAGGCTGCAAAAGCGGACGACGACACCGACGACGATGACGCCGAAGCCAGT
GACGACGAAGAAGAAGCCGAAAGCGGTCCGGATCCGGTCATCGCAGCCCAGCGCTTT
GGCGCCGTTGCCGACCAGATGGAAATCACCCGCAAGGCGCTGAAGAAGCACGGTCGC
GAACACAAGCAAGCCCTGGCCGAAATGCTGGCCCTGGCTGAACTGTTCATGCCGATCA
AACTGGTTCCGAAGCAATTCGAAGGCCTGGTTGAACGTGTTCGTAGCGCCCTGGATCG
CCTGCGTCAGCAAGAGCGCGCGATCATGCAGCTCTGTGTTCGTGATGCCCGCATGCCA
CGCGCCGACTTCCTGCGCCAGTTCCCTGGCAATGAAGTGGACGAAAGCTGGTCCGACG
CGCTGGCCAAAGGCAAGGCCAAGTACGCCGAAGCCATCGGCCGCCTGCAGCCGGACA
TCATCCGTTGCCAGCAGAAGCTGACCGCGCTCGAGACCGAGACCGGCCTGACGATCGC
CGAGATCAAGGACATCAACCGTCGCATGTCGATCGGCGAGGCCAAGGCCCGTCGCGCG
AAGAAAGAGATGGTCGAAGCCAACCTGCGTCTGGTGATCTCCATCGCCAAGAAGTACA
CCAACCGTGGCTTGCAATTCCTCGACCTGATCCAGGAAGGCAACATCGGTTTGATGAA
AGCGGTAGACAAGTTCGAATACCGTCGCGGCTACAAATTCTCGACTTATGCCACCTGG
TGGATCCGTCAGGCGATCACTCGCTCGATCGCCGACCAGGCCCGCACCATCCGTATTCC
GGTGCACATGATCGAGACGATCAACAAGCTCAACCGTATTTCCCGGCAGATGTTGCAG
GAAATGGGTCGCGAACCGACTCCGGAAGAGCTGGGCGAACGCATGGAAATGCCTGAG
GACAAGATCCGCAAGGTATTGAAGATCGCTAAAGAGCCGATCTCCATGGAAACCCCGA
TCGGTGATGACGAAGACTCCCATCTGGGTGACTTCATCGAAGACTCGACCATGCAGTC
GCCAATCGATGTCGCCACCGTTGAGAGCCTTAAAGAAGCGACTCGCGAAGTACTCTCC
GGCCTCACTGCCCGTGAAGCCAAGGTACTGCGCATGCGCTTCGGCATCGACATGAATA
CCGACCACACCCTCGAGGAAGTCGGTAAGCAGTTCGACGTTACCCGTGAGCGGATTCG
TCAGATCGAAGCCAAGGCGCTGCGCAAGCTGCGCCACCCGACGCGAAGCGAGCACCT
GCGCTCCTTCCTCGACGAGTAATTGACTAAGCCAGCCATACTCGCCCTTGCTGATGGCA
GCATTTTTCGCGGCGAAGCCATTGGAGCCGACGGTCAAACCGTTGGTGAGGTGGTGTT
TAACACCGCAATGACCGGCTATCAGGAAATCCTTACCGATCCTTCCTACGCCCAACAG
ATCGTTACCCTGACTTACCCGCATATCGGCAATACCGGCACCACGCCGGAAGACGCCG
AGTCCGATCGTGTCTGGTCGGCCGGTCTGGTGATTCGCGACCTGCCTCTGGTTGCGAGC
AACTGGCGTAACACCCTGTCCCTGTCCGACTACCTGAAAGCCAACAATGTCGTGGCGA
TCGCCGGTATCGACACCCGTCGCCTGACGCGCATCCTGCGCGAGAAAGGTGCGCAGAA
CGGCTGCATCATGGCCGGCGACAATATCTCCGACGAAGCGGCGATTGCCGCTGCACGC
GGCTTCCCGGGCCTGAAAGGCATGGATCTGGCGAAGGTCGTCAGTACCAAGGAAAGCT
ACGAGTGGCGCTCCAGTGTCTGGAACCTGAAGACCGACAGTCATCCGACCATCGAAGC
TTCCGAGCTGCCTTACCACGTGGTTGCCTACGACTACGGCGTCAAGCTGAACATCCTGC
GCATGCTGGTCGAACGCGGTTGCCGCGTGACCGTGGTGCCTGCGCAAACCCCGGCCAG
CGAAGCTCTGGCGCTCAAGCCTGACGGTGTGTTCCTGTCCAACGGCCCTGGCGACCCC
GAGCCTTGCGATTACGCCATCCAGGCGATCAAGGACGTGCTGGAGACCGAGATTCCGG
TCTTCGGTATCTGTCTGGGCCACCAACTGCTGGCACTGGCCGCCGGCGCCAAGACAGT
GAAGATGGGCCACGGCCACCACGGCGCCAACCACCCGGTCCAGGACCTGGACAGCGG
TGTGGTGATGATCACCAGCCAGAACCACGGTTTTGCGGTGGACGAAGCCACCCTGCCG
GGCAACGTGCGGGCGATCCACAAGTCGCTGTTCGACGGCACCCTGCAAGGCATCGAGC
TGACCGACAAGAGCGCATTCAGCTTCCAGGGCCACCCTGAAGCGAGCCCGGGCCCGAA

CGATGTGGCGCCGCTGTTCGATCGTTTCATCAACGAGATGGCCAAGCGACGCTGAATG
AGTAGCGGACGTATCGTTCAAATCATCGGCGCCGTTATCGACGTGGAATTTCCACGCG
ACAGCGTACCGAGCATCTACGACGCCTTGAAGGTTCAAGGCGCCGAAACCACTCTGGA
AGTTCAGCAGCAGCTGGGCGACGGCGTGGTACGTACCATTGCGATGGGCTCCACCGAG
GGCTTGAAGCGCGGTCTGGACGTCAACAACACTGGCGCAGCCATCTCCGTACCGGTCG
GTAAAGCGACCCTGGGCCGGATCATGGACGTACTGGGCAACCCGATCGACGAAGCTGG
CCCGATCGGTGAAGAAGAGCGTTGGGGCATTCACCGTCCTGCGCCGACCTTCGCTGAA
CAAGCTGGCGGCAACGACCTGCTGGAAACCGGCATCAAGGTTATCGACCTGGTTTGCC
CGTTCGCCAAGGGCGGTAAAGTCGGTCTGTTCGGTGGTGCCGGTGTGGGCAAAACCGT
AAACATGATGGAACTGATCCGTAACATCGCCATCGAGCACAGCGGTTATTCCGTGTTC
GCCGGTGTGGGTGAGCGTACTCGTGAGGGTAACGACTTCTACCACGAGATGAAGGATT
CCAACGTTCTGGACAAAGTGGCACTGGTATACGGCCAGATGAACGAGCCGCCGGGAA
ACCGTCTGCGCGTAGCTCTGACCGGCCTGACCATGGCCGAGAAGTTCCGTGACGAAGG
TAACGACGTTCTGCTGTTCGTCGACAACATCTATCGTTACACCCTGGCCGGTACCGAAG
TATCCGCACTGCTGGGCCGTATGCCTTCGGCAGTAGGTTACCAGCCGACCCTGGCTGAA
GAGATGGGCGTTCTGCAAGAACGTATCACTTCGACCAAGCAAGGCTCGATCACCTCGA
TCCAAGCGGTATACGTACCTGCGGACGACTTGACCGACCCGTCGCCAGCGACCACCTT
CGCCCACTTGGACGCCACCGTCGTTCTGTCCCGTGACATCGCTTCCCTGGGTATCTACC
CAGCGGTAGACCCACTGGACTCGACTTCCCGTCAGCTGGACCCGAACGTGATCGGCAC
CGAGCACTACGAAACCGCTCGTGGCGTTCAGTACGTGCTGCAGCGCTACAAAGAGCTG
AAGGACATCATTGCGATCCTGGGTATGGACGAACTGTCCGAAGCCGACAAGCAACTGG
TATCCCGCGCTCGTAAGATCCAGCGCTTCCTGTCGCAGCCGTTCTTCGTGGCTGAAGTC
TTCACTGGTTCTCCAGGCAAATACGTTTCCCTGAAAGACACCATCGCTGGCTTCAAAGG
CATCCTCAACGGTGACTACGACCATCTGCCAGAACAAGCGTTCTACATGGTTGGTGGC
ATCGAAGAAGCGATCGAGAAAGCCAAGAAACTGTAA

NCBI Reference Sequence: NZ_CP027737.1
Strain: PCL1607
Chained genes: 16S rRNA, recA, gyrB, rpoD, carA, atpD
>NZ_CP027737_PCL1607
GAACTGAAGAGTTTGATCATGGCTCAGATTGAACGCTGGCGGCAGGCCTAACACATGC
AAGTCGAGCGGTAGAGAGGTGCTTGCACCTCTTGAGAGCGGCGGACGGGTGAGTAATG
CCTAGGAATCTGCCTGGTAGTGGGGGATAACGTTCGGAAACGGACGCTAATACCGCAT
ACGTCCTACGGGAGAAAGCAGGGGACCTTCGGGCCTTGCGCTATCAGATGAGCCTAGG
TCGGATTAGCTAGTTGGTGAGGTAATGGCTCACCAAGGCGACGATCCGTAACTGGTCT
GAGAGGATGATCAGTCACACTGGAACTGAGACACGGTCCAGACTCCTACGGGAGGCA
GCAGTGGGGAATATTGGACAATGGGCGAAAGCCTGATCCAGCCATGCCGCGTGTGTGA
AGAAGGTCTTCGGATTGTAAAGCACTTTAAGTTGGGAGGAAGGGTACTTACCTAATAC
GTGAGTATTTTGACGTTACCGACAGAATAAGCACCGGCTAACTCTGTGCCAGCAGCCG
CGGTAATACAGAGGGTGCAAGCGTTAATCGGAATTACTGGGCGTAAAGCGCGCGTAGG
TGGTTCGTTAAGTTGAATGTGAAATCCCCGGGCTCAACCTGGGAACTGCATCCAAAAC
TGGCGAGCTAGAGTATGGTAGAGGGTGGTGGAATTTCCTGTGTAGCGGTGAAATGCGT
AGATATAGGAAGGAACACCAGTGGCGAAGGCGACCACCTGGACTGATACTGACACTG
AGGTGCGAAAGCGTGGGGAGCAAACAGGATTAGATACCCTGGTAGTCCACGCCGTAA
ACGATGTCAACTAGCCGTTGGGAGCCTTGAGCTCTTAGTGGCGCAGCTAACGCATTAA
GTTGACCGCCTGGGGAGTACGGCCGCAAGGTTAAAACTCAAATGAATTGACGGGGGCC
CGCACAAGCGGTGGAGCATGTGGTTTAATTCGAAGCAACGCGAAGAACCTTACCAGGC
CTTGACATCCAATGAACTTTCCAGAGATGGATTGGTGCCTTCGGGAACATTGAGACAG
GTGCTGCATGGCTGTCGTCAGCTCGTGTCGTGAGATGTTGGGTTAAGTCCCGTAACGAG
CGCAACCCTTGTCCTTAGTTACCAGCACGTAATGGTGGGCACTCTAAGGAGACTGCCG
GTGACAAACCGGAGGAAGGTGGGGATGACGTCAAGTCATCATGGCCCTTACGGCCTGG

GCTACACACGTGCTACAATGGTCGGTACAGAGGGTTGCCAAGCCGCGAGGTGGAGCTA
ATCCCATAAAACCGATCGTAGTCCGGATCGCAGTCTGCAACTCGACTGCGTGAAGTCG
GAATCGCTAGTAATCGCGAATCAGAATGTCGCGGTGAATACGTTCCCGGGCCTTGTAC
ACACCGCCCGTCACACCATGGGAGTGGGTTGCACCAGAAGTAGCTAGTCTAACCTTCG
GGAGGACGGTTACCACGGTGTGATTCATGACTGGGGTGAAGTCGTAACAAGGTAGCCG
TAGGGGAACCTGCGGCTGGATCACCTCCTTAAATGGACGACAACAAGAAGAAAGCCTT
GGCTGCGGCCCTGGGTCAGATCGAACGTCAATTCGGCAAGGGTGCCGTAATGCGTATG
GGCGATCACGATCGCCAGGCGATCCCGGCCATTTCCACTGGCTCTCTGGGTCTGGACAT
CGCACTCGGCATCGGCGGCCTGCCAAAAGGCCGTATTGTTGAAATCTACGGTCCGGAA
TCGTCCGGTAAAACCACCCTGACCCTGTCGGTGATTGCCCAGGCACAGAAGATGGGCG
CCACCTGCGCCTTCGTCGACGCCGAGCACGCACTGGACCCGGAGTACGCCGGCAAACT
GGGGGTCAACGTTGACGACCTGCTGGTTTCCCAGCCGGACACCGGCGAACAGGCGCTG
GAAATCACCGACATGCTGGTGCGCTCCAATGCCATCGACGTGATCGTGATCGACTCCG
TGGCGGCGCTGGTACCCAAGGCCGAGATCGAAGGCGAGATGGGCGACATGCACGTGG
GCCTGCAGGCTCGCCTGATGTCCCAGGCGCTGCGCAAGATCACCGGTAACATCAAGAA
CGCCAACTGCCTGGTGATCTTCATCAACCAGATCCGTATGAAAATCGGCGTGATGTTCG
GCAGCCCGGAAACCACCACCGGTGGTAACGCGCTGAAGTTCTACGCTTCGGTTCGTCT
GGATATCCGTCGTACTGGCGCGGTGAAGGAAGGTGACGAAGTCGTCGGTAGCGAAACC
CGGGTCAAGATCGTCAAGAACAAGGTGGCTCCACCCTTCCGTCAAGCTGAGTTCCAGA
TCCTGTACGGCAAGGGTATCTACCTGAACGGCGAGATCATCGATCTGGGCGTGCTGCA
CGGTTTCCTAGAGAAGTCCGGTGCCTGGTACAGCTACCAGGGCAACAAGATCGGTCAG
GGCAAGGCCAACTCGGCCAAGTTCCTGCAGGACAATCCGGAAATCGGTAATGCCCTCG
AGAAGCAGATTCGCGACAAGCTGCTGGCTCCGAGCGGAGATACCAAGGCTCTGCCCGT
CAACGAGACCATCGATGACATGGCCGACGCGGATATCTGAATGAGCGAAGAAAACAC
GTACGACTCGAGCAGCATTAAAGTGCTGAAAGGTTTGGATGCCGTACGCAAACGTCCC
GGTATGTACATTGGTGACACCGACGATGGCAGCGGTCTGCACCATATGGTGTTCGAGG
TGGTCGACAACTCGATCGACGAAGCTCTGGCCGGCCACTGCGACGACATCAGCATCAT
CATCCACCCGGACGAATCCATTACCGTGCGTGACAACGGTCGCGGCATCCCGGTAGAC
GTGCATAAAGAAGAAGGCGTTTCCGCAGCCGAGGTCATCATGACCGTGCTGCACGCCG
GCGGTAAGTTCGACGACAACTCCTACAAGGTATCCGGCGGTCTGCACGGTGTGGGTGT
GTCGGTAGTGAATGCCCTGTCCGAAGAACTGGTGCTGACCGTTCGCCGCAGTGGCAAG
ATCTGGGAACAGACCTACGTTCACGGTGTGCCTCAGGCGCCTATGGCGATCGTCGGTG
ACAGCGAGACCACTGGTACCCAGATTCACTTCAAGGCTTCCAGCGAGACCTTCAAGAA
CATCCATTTCAGCTGGGACATCCTGGCCAAGCGGATTCGTGAACTGTCCTTCCTCAACT
CCGGTGTCGGTATCGTTCTGAAGGACGAGCGCAGCGGCAAGGAAGAACTGTTCAAATA
CGAAGGCGGCCTGCGCGCGTTCGTCGAATACCTGAACACCAACAAGACTGCGGTCAAC
CAGGTGTTCCACTTCAACGTGCAGCGTGAAGACGGCATCGGCGTGGAAATCGCCCTGC
AGTGGAACGACAGCTTCAACGAAAACCTGCAGTGCTTCACCAACAACATTCCGCAGCG
CGACGGCGGTACTCACCTGGTGGGCTTCCGCTCGGCACTGACGCGTAACCTGAACAAC
TACATCGAGCAGGAAGGTCTGGCGAAGAAGCACAAGGTCGCCACCACCGGTGACGAT
GCCCGCGAAGGCCTGACCGCGATCATTTCGGTCAAGGTGCCGGATCCGAAGTTCAGCT
CCCAGACCAAAGACAAGCTGGTGTCTTCCGAAGTAAAGACCGCGGTCGAACAGGAAA
TGGGCAAGTACTTCTCCGACTTCCTGCTGGAAAACCCGAACGAAGCCAAGCTGGTGGT
CGGCAAGATGCTCGACGCCGCCCGCGCCCGTGAAGCGGCGCGTAAGGCCCGTGAGATG
ACCCGCCGTAAAGGTGCGCTGGATATCGCCGGCCTGCCGGGCAAACTGGCGGACTGCC
AGGAAAAGGACCCTGCCCTTTCCGAACTCTACCTGGTGGAAGGTGACTCTGCTGGCGG
CTCCGCCAAGCAGGGACGCAACCGCAAGACCCAGGCGATTCTGCCGCTCAAGGGCAA
GATTCTTAACGTCGAGAAAGCGCGCTTCGACAAGATGATTTCCTCGCAAGAGGTCGGC
ACCTTGATCACTGCACTCGGCTGCGGCATCGGCCGCGAAGAGTACAACATCGACAAGC
TGCGTTATCACAACATCATCATCATGACCGACGCCGACGTCGACGGTTCGCACATCCGC
ACCCTGCTGCTGACTTTCTTCTTCCGTCAGCTGCCGGAGCTGATCGAGCGTGGCTACAT

CTACATCGCTCAACCGCCGCTGTACAAGGTCAAGAAAGGCAAGCAAGAGCAATACATC
AAAGACGACGACGCCATGGAAGAGTACATGACGCAGTCGGCCCTGGAAGATGCGAGC
CTGCACCTGAACGAAGAAGCACCGGGTATTTCCGGCGAGGCGCTGGAGCGCCTGGTGA
ACGACTTCCGCATGGTCATGAAAACCCTCAAGCGTCTGTCGCGCCTGTACCCTCAGGA
GCTGACCGAACACTTCATCTACCTGCCAGCCGTGAGCCTGGAGCAACTCTCCGATCAC
GCAGCGATGCAGGATTGGTTGGCCCAATATGAAGTCCGCCTGCGCACCGTCGAGAAGT
CCGGCCTGGTCTACAAGGCCAGCCTGCGTGAAGACCGTGAACGTAATGTCTGGCTGCC
AGAGGTCGAACTGATCTCCCACGGCCTGTCGAACTACGTCACCTTCAACCGCGACTTCT
TCGGCAGCAATGACTACAAGACCGTCGTCACCCTCGGCGCTCAACTGAGCTCCCTGCT
GGACGAAGGCGCTTATATTCAGCGCGGCGAACGCAAGAAGGCGGTGACCGAGTTCAA
GGAAGCCCTGGACTGGCTGATGACCGAAAGCACCAAGCGCCACACCATCCAGCGATAC
AAAGGTCTGGGCGAGATGAACCCGGACCAGCTGTGGGAAACCACCATGGACCCAAGC
GTGCGCCGCATGCTCAAGGTCACCATCGAAGACGCCATCGGCGCCGACCAGATCTTCA
ACACCCTGATGGGTGATGCGGTCGAGCCTCGTCGCGACTTCATCGAAAGCAACGCCCT
GGCGGTATCCAACCTGGACTTCTGAATGTCCGGAAAAGCGCAACAGCAGTCTCGCCTC
AAAGAGTTGATCAGCCGTGGTCGTGAGCAGGGTTACCTGACTTACGCGGAGGTCAACG
ACCACCTGCCGGAGGATATTTCAGATCCGGAACAGGTGGAAGACATCATCCGCATGAT
CAACGACATGGGGATCAACGTATTCGAGAGTGCTCCGGATGCGGATGCCCTTTTGTTG
GCCGAAGCCGATACCGACGAAGCAGCAGCTGAAGAAGCCGCCGCAGCGTTGGCGGCT
GTGGAAACCGACATTGGTCGCACTACCGACCCAGTGCGTATGTACATGCGCGAAATGG
GCACGGTAGAGCTGCTCACACGTGAAGGCGAAATCGAAATCGCCAAGCGTATCGAAG
AGGGCATCCGTGAAGTGATGGGCGCGATCGCGCACTTCCCTGGCACGGTTGAGCACAT
CCTCTCCGAATACACTCGCGTCACCACCGAAGGTGGCCGCCTGTCCGACGTCCTGAGC
GGTTACATCGACCCGGATGACGGCATTGCGCCGCCTGCCGCCGAAGTACCACCGCCTG
TCGATGCCAAGGCCGCGAAAGCGGACGACGACACCGACGACGATGACGCCGAAGCCA
GTGACGACGAAGAAGAAGCCGAAAGCGGTCCGGATCCGGTCATCGCAGCCCAGCGCT
TTGGCGCCGTTGCCGACCAGATGGAAATCACCCGCAAGGCGCTGAAAAAGCACGGTCG
TGAACACAAGCAAGCCCTGGCTGAAATGCTGGCCCTGGCTGAACTGTTCATGCCAATC
AAACTGGTTCCGAAGCAATTCGAAGGCCTGGTTGAACGTGTTCGTAGCGCCCTGGATC
GCCTGCGTCAGCAAGAGCGCGCGATCATGCAGCTCTGTGTTCGTGATGCCCGCATGCC
ACGCGCCGACTTCCTGCGCCAGTTCCCTGGCAATGAAGTGGACGAAAGCTGGTCCGAC
GCGCTGGCCAAAGGCAAGGCCAAGTACGCCGAAGCCATCGGCCGCCTGCAGCCGGAC
ATCATCCGTTGCCAGCAGAAGCTGACCGCGCTCGAGACCGAGACCGGCCTGACGATTG
CCGAGATCAAGGACATCAACCGTCGCATGTCGATCGGCGAGGCCAAGGCCCGTCGCGC
GAAGAAAGAGATGGTCGAAGCCAACTTGCGCCTGGTGATCTCCATCGCCAAGAAGTAC
ACCAACCGTGGCCTGCAGTTCCTCGACCTGATCCAGGAAGGCAACATCGGTTTGATGA
AAGCGGTAGACAAGTTCGAATACCGTCGCGGCTACAAGTTCTCGACTTATGCCACCTG
GTGGATCCGTCAGGCGATCACTCGCTCGATCGCCGACCAGGCCCGCACCATCCGTATTC
CGGTGCACATGATCGAGACGATCAACAAGCTCAACCGTATTTCCCGGCAGATGTTGCA
GGAAATGGGTCGCGAACCGACTCCGGAAGAGCTGGGCGAACGCATGGAAATGCCTGA
GGACAAGATCCGCAAGGTATTGAAGATCGCTAAAGAGCCGATCTCCATGGAAACCCCG
ATCGGTGATGACGAAGACTCCCATCTGGGTGACTTCATCGAAGACTCGACCATGCAGT
CGCCAATCGATGTCGCCACCGTTGAGAGCCTCAAGGAAGCGACTCGCGAAGTCCTCTC
CGGCCTCACTGCCCGTGAAGCCAAGGTACTGCGCATGCGCTTCGGCATCGACATGAAT
ACCGATCACACCCTTGAGGAAGTCGGTAAGCAGTTCGACGTTACCCGTGAGCGGATTC
GTCAGATCGAAGCCAAGGCGCTGCGCAAGCTGCGCCACCCGACGAGAAGCGAGCATC
TGCGCTCCTTCCTCGACGAGTAATTGACTAAGCCAGCCATACTCGCCCTTGCTGATGGC
AGCATTTTTCGCGGCGAAGCCATTGGAGCCGACGGTCAAACCGTTGGTGAGGTGGTGT
TTAACACCGCAATGACCGGCTATCAGGAAATCCTTACCGATCCTTCCTACGCCCAACAG
ATCGTTACCCTGACTTACCCGCACATCGGCAACACTGGCACCACGCCGGAAGACGCCG
AGTCCGATCGTGTCTGGTCGGCCGGTCTGGTGATTCGCGACCTGCCACTGGTTGCGAGC

AACTGGCGTAACACCCTGTCCCTGTCCGACTACCTGAAAGCCAACAATGTCGTGGCGA
TCGCCGGTATCGACACCCGTCGCCTGACGCGCATCCTGCGTGAAAAAGGCGCGCAGAA
CGGCTGCATCATGGCCGGCGACAATATCTCCGACGAAGCAGCGATTGCCGCAGCGCGC
GGCTTCCCTGGCCTGAAAGGCATGGATCTGGCGAAGGTCGTCAGCACCAAGGAAAGCT
ACGAGTGGCGCTCCAGCGTCTGGAGCCTGAAGACCGACAGTCATCCGACTATCGAGGC
TTCCGAGCTGCCTTACCACGTGGTTGCCTACGACTACGGCGTCAAGCTGAACATCCTGC
GCATGCTGGTCGAGCGCGGTTGCCGCGTGACCGTAGTGCCTGCGCAAACCCCGGCCAG
CGACGTCCTGGCACTCAAGCCTGACGGTGTGTTCCTGTCCAACGGTCCTGGTGACCCCG
AGCCTTGCGATTACGCCATCCAGGCGATCAAGGACGTGCTGGAAACCGAGATTCCGGT
CTTCGGTATCTGCCTGGGCCACCAACTGCTGGCACTGGCCTCCGGCGCCAAGACGGTG
AAGATGGGCCACGGTCACCACGGTGCCAACCACCCGGTCCAGGACCTGGACAGCGGTG
TAGTGATGATCACTAGCCAGAACCACGGTTTTGCGGTGGACGAAGCCACCCTGCCGGG
CAACGTGCGGGCGATCCACAAGTCGCTGTTCGACGGCACCCTGCAAGGCATCGAGCGT
ACCGACAAGAGTGCATTCAGCTTCCAGGGCCACCCTGAAGCGAGCCCGGGCCCGAACG
ATGTGGCGCCGCTGTTCGATCGTTTCATCAACGAGATGGTCAAGCGACGCTGAATGAG
TAGCGGACGTATCGTTCAAATCATCGGCGCCGTTATCGACGTGGAATTTCCACGCGAC
AGCGTACCGAGCATCTACGACGCCTTGAAGGTTCAAGGCGCCGAAACCACTCTGGAAG
TTCAGCAGCAGCTGGGCGACGGCGTGGTACGTACCATTGCGATGGGCTCCACCGAGGG
CTTGAAGCGCGGTCTGGACGTCAACAACACTGGCGCAGCCATCTCCGTACCGGTCGGT
AAAGCGACCCTGGGCCGGATCATGGACGTACTGGGCAACCCGATCGACGAAGCTGGCC
CGATCGGCGAAGAAGAGCGTTGGGGCATTCACCGTCCTGCGCCGACCTTCGCTGAACA
AGCTGGCGGCAACGACCTCCTGGAAACCGGCATCAAGGTTATCGACCTGGTTTGCCCG
TTCGCCAAGGGCGGTAAAGTCGGTCTGTTCGGTGGTGCCGGTGTGGGCAAAACCGTAA
ACATGATGGAACTGATCCGTAACATCGCCATCGAGCACAGCGGTTATTCCGTGTTCGCC
GGTGTGGGTGAGCGTACTCGTGAGGGTAACGACTTCTACCACGAGATGAAGGATTCCA
ACGTTCTGGACAAAGTGGCACTGGTATACGGCCAGATGAACGAGCCGCCGGGAAACC
GTCTGCGCGTAGCTCTGACCGGCCTGACCATGGCCGAGAAGTTCCGTGACGAAGGTAA
CGACGTTCTGCTGTTCGTCGACAACATCTATCGTTACACCCTGGCCGGTACCGAAGTAT
CCGCACTGCTGGGCCGTATGCCTTCGGCAGTAGGTTACCAGCCGACCCTGGCTGAAGA
GATGGGCGTTCTGCAAGAACGTATCACTTCGACCAAGCAAGGCTCGATCACCTCGATC
CAAGCGGTATACGTACCTGCGGACGACTTGACCGACCCGTCGCCAGCGACCACCTTCG
CCCACTTGGACGCCACCGTCGTACTGTCCCGTGACATCGCTTCCCTGGGTATCTACCCA
GCGGTAGACCCACTGGATTCGACTTCCCGTCAGCTGGACCCGAACGTGATCGGCAACG
AGCACTACGAAACTGCTCGCGGCGTTCAGTACGTGCTGCAGCGCTACAAAGAGCTGAA
GGACATCATTGCGATCCTGGGTATGGACGAACTGTCCGAAGCCGACAAGCAACTGGTA
TCCCGCGCTCGTAAGATCCAGCGCTTCCTGTCGCAGCCGTTCTTCGTGGCTGAAGTCTT
CACTGGTTCTCCAGGCAAATACGTTTCCCTGAAAGACACCATCGCTGGCTTCAAAGGC
ATCCTCAACGGTGACTACGACCACCTGCCAGAACAAGCGTTCTACATGGTCGGCGGCA
TCGAAGAAGCGATCGAGAAAGCCAAGAAACTGTAA

RAST server Genome ID: 286.2086
Strain: ST9
Chained genes: 16S rRNA, recA, gyrB, rpoD, carA, atpD
>ID_286.2086_ST9
GAACTGAAGAGTTTGATCATGGCTCAGATTGAACGCTGGCGGCAGGCCTAACACATGC
AAGTCGAGCGGTAGAGAGAAGCTTGCTTCTCTTGAGAGCGGCGGACGGGTGAGTAATG
CCTAGGAATCTGCCTGGTAGTGGGGGATAACGTCCGGAAACGGACGCTAATACCGCAT
ACGTCCTACGGGAGAAAGCAGGGGACCTTCGGGCCTTGCGCTATCAGATGAGCCTAGG
TCGGATTAGCTAGTTGGTGAGGTAATGGCTCACCAAGGCGACGATCCGTAACTGGTCT
GAGAGGATGATCAGTCACACTGGAACTGAGACACGGTCCAGACTCCTACGGGAGGCA
GCAGTGGGGAATATTGGACAATGGGCGAAAGCCTGATCCAGCCATGCCGCGTGTGTGA

AGAAGGTCTTCGGATTGTAAAGCACTTTAAGTTGGGAGGAAGGGTACTTACCTAATAC
GTGAGTATTTTGACGTTACCGACAGAATAAGCACCGGCTAACTCTGTGCCAGCAGCCG
CGGTAATACAGAGGGTGCAAGCGTTAATCGGAATTACTGGGCGTAAAGCGCGCGTAGG
TGGTTCGTTAAGTTGGATGTGAAATCCCCGGGCTCAACCTGGGAACTGCATCCAAAAC
TGGCGAGCTAGAGTATGGTAGAGGGTGGTGGAATTTCCTGTGTAGCGGTGAAATGCGT
AGATATAGGAAGGAACACCAGTGGCGAAGGCGACCACCTGGACTGATACTGACACTG
AGGTGCGAAAGCGTGGGGAGCAAACAGGATTAGATACCCTGGTAGTCCACGCCGTAA
ACGATGTCAACTAGCCGTTGGGAGCCTTGAGCTCTTAGTGGCGCAGCTAACGCATTAA
GTTGACCGCCTGGGGAGTACGGCCGCAAGGTTAAAACTCAAATGAATTGACGGGGGCC
CGCACAAGCGGTGGAGCATGTGGTTTAATTCGAAGCAACGCGAAGAACCTTACCAGGC
CTTGACATCCAATGAACTTTCCAGAGATGGATTGGTGCCTTCGGGAACATTGAGACAG
GTGCTGCATGGCTGTCGTCAGCTCGTGTCGTGAGATGTTGGGTTAAGTCCCGTAACGAG
CGCAACCCTTGTCCTTAGTTACCAGCACGTTATGGTGGGCACTCTAAGGAGACTGCCGG
TGACAAACCGGAGGAAGGTGGGGATGACGTCAAGTCATCATGGCCCTTACGGCCTGGG
CTACACACGTGCTACAATGGTCGGTACAGAGGGTTGCCAAGCCGCGAGGTGGAGCTAA
TCCCATAAAACCGATCGTAGTCCGGATCGCAGTCTGCAACTCGACTGCGTGAAGTCGG
AATCGCTAGTAATCGCGAATCAGAATGTCGCGGTGAATACGTTCCCGGGCCTTGTACA
CACCGCCCGTCACACCATGGGAGTGGGTTGCACCAGAAGTAGCTAGTCTAACCTTCGG
GAGGACGGTTACCACGGTGTGATTCATGACTGGGGTGAAGTCGTAACAAGGTAGCCGT
AGGGGAACCTGCGGCTGGATCACCTCCTTAAATGGACGACAACAAGAAGAAAGCCTTG
GCTGCGGCCCTGGGTCAGATCGAACGTCAATTCGGCAAGGGTGCCGTAATGCGTATGG
GCGATCACGACCGCCAGGCGATCCCGGCCATTTCCACTGGCTCTCTGGGTCTGGACATC
GCACTCGGCATCGGCGGCCTGCCAAAAGGCCGTATTGTTGAAATCTACGGTCCGGAAT
CGTCCGGTAAAACCACCCTGACCCTGTCGGTGATTGCCCAGGCACAGAAGATGGGCGC
CACCTGCGCCTTCGTCGACGCCGAGCACGCACTGGACCCGGAATACGCCGGCAAACTG
GGGGTCAACGTTGACGACCTGCTGGTTTCCCAGCCGGACACCGGCGAACAGGCGCTGG
AAATCACCGACATGCTGGTGCGCTCCAATGCCATCGACGTGATCGTGATCGACTCCGT
GGCGGCACTGGTACCCAAGGCCGAGATCGAAGGCGAGATGGGCGACATGCACGTGGG
CCTGCAGGCCCGCCTGATGTCCCAGGCGCTGCGCAAGATCACCGGTAACATCAAGAAC
GCCAACTGCCTGGTGATCTTCATCAACCAGATCCGTATGAAAATCGGCGTGATGTTCGG
CAGCCCGGAAACCACCACCGGTGGTAACGCGCTGAAGTTCTACGCTTCGGTTCGTCTG
GACATCCGTCGTACTGGCGCGGTGAAGGAAGGCGACGAAGTCGTCGGTAGCGAAACC
CGGGTCAAGATCGTCAAGAACAAGGTGGCTCCACCGTTCCGTCAGGCTGAATTCCAGA
TCCTGTACGGCAAGGGTATCTACCTGAACGGCGAGATCATCGATCTGGGCGTGCTGCA
CGGTTTCCTCGAGAAGTCCGGTGCCTGGTACAGCTACCAGGGCAACAAGATCGGTCAG
GGCAAGGCCAACTCGGCCAAGTTCCTGCAGGACAATCCGGAAATCGGCAATGCCCTCG
AGAAGCAGATTCGCGACAAGCTGCTGGCTCCAACCGCTGATGTCAAAGCTTCGCCGGT
CAACGAGACCATCGATGACATGGCTGACGCGGATATCTGAATGAGCGAAGAAAACAC
GTACGACTCGAGCAGCATTAAAGTGCTGAAAGGTTTGGATGCCGTACGCAAACGTCCC
GGTATGTACATTGGTGACACCGACGATGGCAGCGGTCTGCACCATATGGTGTTCGAGG
TGGTCGATAACTCGATCGACGAAGCTCTGGCCGGCCACTGCGACGACATCAGCATCAT
CATCCACCCAGACGAATCCATTACCGTGCGTGACAACGGTCGCGGCATCCCGGTAGAC
GTGCATAAAGAAGAAGGCGTTTCCGCGGCCGAGGTCATCATGACTGTGCTGCACGCCG
GCGGTAAGTTCGACGACAACTCCTACAAAGTATCCGGCGGTCTGCACGGTGTGGGTGT
GTCGGTAGTGAACGCCCTGTCCGAAGAACTGGTCCTGACCGTTCGCCGCAGTGGCAAG
ATCTGGGAACAGACCTACGTTCACGGTGTGCCTCAGGCGCCTATGGCGATCGTCGGTG
ACAGTGAAACCACCGGTACCCAGATTCACTTCAAGGCTTCCAGCGAGACCTTCAAGAA
CATCCATTTCAGCTGGGACATCCTGGCCAAGCGGATTCGTGAACTGTCCTTCCTCAACT
CCGGTGTCGGTATCGTTCTGAAGGACGAGCGCAGCGGCAAGGAAGAACTGTTCAAGTA
CGAAGGCGGTCTGCGTGCGTTCGTTGAATACCTGAACACCAACAAGACCGCGGTCAAC
CAGGTGTTCCACTTCAATGTGCAGCGTGAAGATGGCATCGGCGTGGAAATCGCCCTGC

AGTGGAACGACAGCTTCAACGAAAACCTGCAGTGCTTCACCAACAACATTCCGCAGCG
CGACGGCGGCACCCACTTGGTGGGCTTCCGTTCGGCACTGACACGTAACCTGAACAAC
TACATCGAACAGGAAGGTCTGGCGAAGAAGCACAAGGTCGCCACCACCGGTGACGAT
GCCCGCGAAGGCCTGACCGCGATCATTTCGGTCAAGGTGCCGGATCCGAAGTTCAGCT
CCCAGACCAAAGACAAGCTGGTGTCTTCCGAAGTGAAGACCGCGGTCGAACAGGAAA
TGGGCAAGTACTTCTCCGACTTCCTGCTGGAAAACCCGAACGAAGCCAAGCTGGTGGT
CGGCAAGATGCTCGACGCCGCCCGTGCCCGTGAAGCGGCGCGTAAGGCTCGCGAGATG
ACCCGCCGTAAAGGTGCGCTGGATATCGCCGGCCTGCCGGGCAAACTGGCGGACTGCC
AGGAAAAAGACCCTGCCCTTTCCGAACTCTACCTGGTGGAAGGTGACTCTGCTGGCGG
CTCCGCCAAGCAGGGACGCAACCGTAAGACCCAGGCGATTCTGCCGCTCAAGGGCAAG
ATCCTTAACGTCGAGAAAGCGCGTTTCGACAAGATGATTTCCTCGCAAGAGGTCGGCA
CCTTGATCACTGCACTCGGTTGCGGCATCGGCCGCGAAGAGTACAACATCGACAAGCT
GCGTTATCACAACATCATCATCATGACCGACGCCGACGTCGACGGTTCGCACATCCGT
ACCCTGCTGCTGACCTTCTTCTTCCGTCAGCTGCCGGAGCTGATCGAGCGTGGCTACAT
CTACATCGCTCAACCGCCGCTGTACAAGGTCAAGAAAGGCAAGCAAGAGCAATACATC
AAAGACGACGACGCCATGGAAGAGTACATGACGCAGTCGGCCCTGGAAGATGCGAGC
CTGCACCTGAACGAAGACGCACCGGGTATTTCCGGCGAGGCGCTGGAGCGCCTGGTGA
ACGACTTCCGCATGGTCATGAAAACCCTCAAGCGTCTGTCGCGCCTGTACCCTCAGGA
GCTGACCGAGCACTTCATCTACCTGCCGGCCGTGAGCCTGGAGCAGCTCTCCGATCAC
GCGGCGATGCAGGATTGGCTGGCCCAATATGAAGTCCGCCTGCGCACCGTCGAGAAGT
CCGGCCTGGTCTACAAGGCCAGCCTGCGTGAAGACCGTGAACGTAATGTCTGGCTGCC
AGAGGTCGAACTGATCTCCCACGGCCTGTCGAACTACGTCACCTTCAACCGCGACTTCT
TCGGCAGCAATGACTACAAGACCGTCGTCACCCTCGGTGCTCAACTGAGCTCCCTGCTG
GACGAAGGCGCTTATATTCAGCGTGGCGAACGCAAGAAGGCAGTGACCGAGTTCAAG
GAAGCCCTGGACTGGCTGATGACCGAAAGTACCAAGCGCCACACCATCCAGCGATACA
AAGGTCTGGGCGAGATGAACCCGGATCAGCTGTGGGAAACCACTATGGACCCAAGCGT
GCGCCGTATGCTCAAGGTCACCATCGAAGACGCCATCGGCGCCGACCAGATCTTCAAC
ACCCTGATGGGTGATGCGGTCGAGCCTCGTCGCGACTTCATCGAAAGCAACGCCCTGG
CGGTATCCAACCTGGACTTCTGAATGTCCGGAAAAGCGCAACAGCAGTCTCGCCTCAA
AGAGTTGATCAGCCGTGGTCGTGAGCAGGGTTACCTGACTTACGCGGAGGTCAACGAC
CACCTGCCGGAGGATATTTCAGATCCGGAACAGGTGGAAGACATCATCCGCATGATCA
ACGACATGGGGATCAACGTATTCGAGAGTGCTCCGGATGCGGATGCCCTTTTGTTGGC
CGAAGCCGATACCGACGAAGCAGCAGCTGAAGAAGCCGCCGCAGCGTTGGCGGCTGT
GGAAACCGACATTGGTCGCACTACCGACCCCGTGCGTATGTACATGCGCGAAATGGGT
ACGGTAGAGCTGCTCACACGTGAAGGCGAAATCGAAATCGCCAAGCGTATCGAAGAG
GGCATCCGTGAAGTGATGGGCGCGATCGCGCACTTCCCTGGCACGGTTGAGCACATCC
TCTCCGAATACACTCGCGTCACCACCGAAGGTGGCCGCCTGTCCGACGTCCTGAGCGG
TTACATCGACCCGGACGACGGTATTGCGCCGCCTGCCGCCGAAGTACCACCGCCTGTC
GATGCCAAGGCCGCAAAAGCGGACGACGACACCGACGACGATGACGCCGAAGCCAGT
GACGACGAAGAAGAAGCCGAAAGCGGTCCGGATCCGGTCATCGCAGCCCAGCGCTTT
GGCGCCGTTGCCGACCAGATGGAAATTACCCGCAAGGCGCTGAAAAAGCACGGTCGC
GAACACAAGCAAGCCCTGGCTGAAATGCTGGCCCTGGCTGAGCTGTTCATGCCGATCA
AACTGGTTCCGAAGCAATTCGAAGGCCTGGTTGAACGTGTTCGTAGCGCCCTGGATCG
CCTGCGTCAGCAAGAGCGCGCGATCATGCAGCTCTGTGTTCGTGATGCCCGCATGCCA
CGCGCCGACTTCCTGCGCCAGTTCCCTGGCAATGAAGTGGACGAAAGCTGGTCCGACG
CACTGGCCAAAGGCAAGGCCAAGTACGCCGAATCCATTGGCCGCCTGCAGCCGGATAT
CATCCGTTGCCAGCAGAAGCTGACCGCGCTCGAGACCGAGACCGGCCTGACGATCGCC
GAGATCAAGGACATCAACCGTCGCATGTCGATCGGCGAGGCCAAGGCCCGTCGCGCGA
AGAAAGAGATGGTCGAAGCCAACTTGCGTCTGGTGATCTCCATCGCCAAGAAGTACAC
CAACCGTGGCCTGCAATTCCTCGACCTGATCCAGGAAGGCAACATCGGTTTGATGAAA
GCGGTAGACAAGTTCGAATACCGTCGCGGCTACAAGTTCTCGACTTATGCCACCTGGT

GGATCCGTCAGGCGATCACTCGCTCGATCGCCGACCAGGCCCGCACCATCCGTATTCC
GGTGCACATGATCGAGACGATCAACAAGCTCAACCGTATTTCCCGGCAGATGTTGCAG
GAAATGGGTCGCGAACCGACTCCGGAAGAGCTGGGCGAACGCATGGAAATGCCTGAA
GACAAGATCCGCAAGGTATTGAAGATCGCTAAAGAGCCGATCTCCATGGAAACCCCGA
TCGGTGATGACGAAGACTCCCATCTGGGTGACTTCATCGAAGACTCGACCATGCAGTC
GCCAATCGATGTCGCCACCGTTGAGAGCCTTAAAGAAGCGACTCGCGAAGTACTCTCC
GGCCTCACTGCCCGTGAAGCCAAGGTACTGCGCATGCGCTTCGGCATCGACATGAATA
CCGACCACACCCTCGAGGAAGTCGGTAAGCAGTTCGACGTTACCCGTGAGCGGATTCG
TCAGATCGAAGCCAAGGCGCTGCGCAAGCTGCGCCACCCGACGCGAAGCGAGCACCT
GCGCTCCTTCCTCGACGAGTAATTGACTAAGCCAGCCATACTCGCCCTTGCTGATGGCA
GCATTTTTCGCGGCGAAGCCATTGGAGCCGACGGTCAGACCGTTGGTGAGGTGGTGTT
TAACACCGCAATGACCGGCTATCAGGAAATCCTTACCGATCCTTCCTACGCCCAACAG
ATCGTTACCCTGACTTACCCGCACATCGGCAATACCGGCACCACGCCGGAAGACGCCG
AGTCCGATCGTGTCTGGTCGGCCGGTCTGGTGATTCGCGACCTGCCACTGGTTGCGAGC
AACTGGCGTAACACCCTGTCCCTGTCCGACTACCTGAAAGCCAACAACGTTGTGGCGA
TCGCCGGTATCGACACCCGTCGCCTGACGCGCATCCTGCGCGAGAAAGGCGCGCAGAA
CGGCTGCATCATGGCCGGCGACAATATCTCCGACGAAGCGGCGATTGCCGCTGCGCGC
GGCTTCCCTGGCCTGAAAGGCATGGATCTGGCGAAGGTCGTCAGCACCAAGGAAAGCT
ACGAATGGCGTTCCAGCGTCTGGAGCCTGAAGACCGACAGTCATCCGACCATCGAGGC
TTCCGAGCTGCCTTACCACGTGGTTGCCTACGACTACGGCGTCAAGCTGAACATCCTGC
GCATGCTGGTCGAGCGCGGTTGCCGCGTGACCGTGGTACCTGCGCAAACCCCGGCCAG
CGACGTCCTGGCGCTCAAGCCTGACGGTGTGTTTCTGTCCAACGGTCCTGGCGACCCCG
AGCCTTGCGATTACGCGATCCAGGCGATCAAGGACGTGCTGGAGACCGAGATCCCGGT
CTTCGGTATCTGCCTGGGCCACCAACTGCTGGCGCTGGCCGCCGGCGCCAAGACAGTG
AAGATGGGCCACGGCCACCACGGTGCCAACCACCCGGTCCAGGACCTGGACAGCGGT
GTAGTGATGATCACCAGCCAGAACCACGGTTTTGCGGTGGACGAAACCACCCTGCCGG
GCAACGTGCGGGCGATCCACAAGTCGCTGTTCGACGGCACCCTGCAAGGCATCGAGTT
GACCGACAAGAGCGCATTCAGCTTCCAGGGGCACCCTGAAGCGAGCCCGGGCCCGAA
CGATGTGGCGCCGCTGTTCGATCGTTTCATCAACGAGATGGCCAAGCGACGCTGAATG
AGTAGCGGACGTATCGTTCAAATCATCGGCGCCGTTATCGACGTGGAATTTCCACGCG
ACAGCGTACCGAGCATCTACGACGCCTTGAAGGTTCAAGGCGCCGAAACCACTCTGGA
AGTTCAGCAGCAGCTGGGCGACGGCGTGGTACGTACCATTGCGATGGGCTCCACCGAG
GGCTTGAAGCGCGGTCTGGACGTCAACAACACTGGCGCAGCCATCTCCGTACCGGTCG
GTAAAGCGACCCTGGGCCGGATCATGGACGTACTGGGCAACCCGATCGACGAAGCTGG
CCCGATCGGCGAAGAAGAGCGTTGGGGCATTCACCGTCCTGCGCCGACCTTCGCTGAA
CAAGCTGGCGGCAACGACCTGCTGGAAACCGGCATCAAGGTTATCGACCTGGTTTGCC
CGTTCGCCAAGGGCGGTAAAGTCGGTCTGTTCGGTGGTGCCGGTGTGGGCAAAACCGT
AAACATGATGGAACTGATCCGTAACATCGCCATCGAGCACAGCGGTTATTCCGTGTTC
GCCGGTGTGGGTGAGCGTACTCGTGAGGGTAACGACTTCTACCACGAGATGAAGGATT
CCAACGTTCTGGACAAAGTGGCACTGGTATACGGCCAGATGAACGAGCCGCCGGGAA
ACCGTCTGCGCGTAGCTCTGACCGGCCTGACCATGGCCGAGAAGTTCCGTGACGAAGG
TAACGACGTTCTGCTGTTCGTCGACAACATCTATCGTTACACCCTGGCCGGTACCGAAG
TATCCGCACTGCTGGGCCGTATGCCTTCGGCAGTAGGTTACCAGCCGACCCTGGCTGAA
GAGATGGGCGTTCTGCAAGAACGTATCACTTCGACCAAGCAAGGCTCGATCACCTCGA
TCCAAGCGGTATACGTGCCTGCGGACGACTTGACCGACCCGTCGCCAGCGACCACCTT
CGCCCACTTGGACGCCACCGTCGTTCTGTCCCGTGACATCGCTTCCCTGGGTATCTACC
CAGCGGTAGACCCACTGGACTCGACTTCCCGTCAGCTGGACCCGAACGTGATCGGCAA
CGAGCACTACGAAACCGCTCGCGGCGTTCAGTACGTGCTGCAGCGCTACAAAGAGCTG
AAGGACATCATTGCGATCCTGGGTATGGACGAACTGTCCGAAGCCGACAAGCAACTGG
TATCCCGCGCTCGTAAGATCCAGCGCTTCCTGTCGCAGCCGTTCTTCGTGGCTGAAGTC
TTCACTGGTTCTCCAGGCAAATACGTTTCCCTGAAAGACACCATCGCTGGCTTCAAAGG

CATCCTCAACGGTGACTACGACCATCTGCCAGAACAAGCGTTCTACATGGTTGGTGGC
ATCGAAGAAGCGATCGAGAAAGCCAAGAAACTGTAA

NCBI Reference Sequence: NZ_CP027708.1
Strain: ATCC17411
Chained genes: 16S rRNA, recA, gyrB, rpoD, carA, atpD
>NZ_CP027708_ATCC17411
GAACTGAAGAGTTTGATCATGGCTCAGATTGAACGCTGGCGGCAGGCCTAACACATGC
AAGTCGAGCGGTAGAGAGGTGCTTGCACCTCTTGAGAGCGGCGGACGGGTGAGTAATG
CCTAGGAATCTGCCTGGTAGTGGGGGATAACGTTCGGAAACGGACGCTAATACCGCAT
ACGTCCTACGGGAGAAAGCAGGGGACCTTCGGGCCTTGCGCTATCAGATGAGCCTAGG
TCGGATTAGCTAGTTGGTGAGGTAATGGCTCACCAAGGCGACGATCCGTAACTGGTCT
GAGAGGATGATCAGTCACACTGGAACTGAGACACGGTCCAGACTCCTACGGGAGGCA
GCAGTGGGGAATATTGGACAATGGGCGAAAGCCTGATCCAGCCATGCCGCGTGTGTGA
AGAAGGTCTTCGGATTGTAAAGCACTTTAAGTTGGGAGGAAGGGTACTTACCTAATAC
GTGAGTATTTTGACGTTACCGACAGAATAAGCACCGGCTAACTCTGTGCCAGCAGCCG
CGGTAATACAGAGGGTGCAAGCGTTAATCGGAATTACTGGGCGTAAAGCGCGCGTAGG
TGGTTCGTTAAGTTGAATGTGAAATCCCCGGGCTCAACCTGGGAACTGCATCCAAAAC
TGGCGAGCTAGAGTATGGTAGAGGGTGGTGGAATTTCCTGTGTAGCGGTGAAATGCGT
AGATATAGGAAGGAACACCAGTGGCGAAGGCGACCACCTGGACTGATACTGACACTG
AGGTGCGAAAGCGTGGGGAGCAAACAGGATTAGATACCCTGGTAGTCCACGCCGTAA
ACGATGTCAACTAGCCGTTGGGAGCCTTGAGCTCTTAGTGGCGCAGCTAACGCATTAA
GTTGACCGCCTGGGGAGTACGGCCGCAAGGTTAAAACTCAAATGAATTGACGGGGGCC
CGCACAAGCGGTGGAGCATGTGGTTTAATTCGAAGCAACGCGAAGAACCTTACCAGGC
CTTGACATCCAATGAACTTTCCAGAGATGGATTGGTGCCTTCGGGAACATTGAGACAG
GTGCTGCATGGCTGTCGTCAGCTCGTGTCGTGAGATGTTGGGTTAAGTCCCGTAACGAG
CGCAACCCTTGTCCTTAGTTACCAGCACGTAATGGTGGGCACTCTAAGGAGACTGCCG
GTGACAAACCGGAGGAAGGTGGGGATGACGTCAAGTCATCATGGCCCTTACGGCCTGG
GCTACACACGTGCTACAATGGTCGGTACAGAGGGTTGCCAAGCCGCGAGGTGGAGCTA
ATCCCATAAAACCGATCGTAGTCCGGATCGCAGTCTGCAACTCGACTGCGTGAAGTCG
GAATCGCTAGTAATCGCGAATCAGAATGTCGCGGTGAATACGTTCCCGGGCCTTGTAC
ACACCGCCCGTCACACCATGGGAGTGGGTTGCACCAGAAGTAGCTAGTCTAACCTTCG
GGAGGACGGTTACCACGGTGTGATTCATGACTGGGGTGAAGTCGTAACAAGGTAGCCG
TAGGGGAACCTGCGGCTGGATCACCTCCTTAAATGGACGACAACAAGAAGAAAGCCTT
GGCTGCGGCCCTGGGTCAGATCGAACGTCAATTCGGCAAGGGTGCCGTAATGCGTATG
GGCGATCACGATCGCCAGGCGATCCCGGCCATTTCCACTGGCTCTCTGGGTCTGGACAT
CGCACTCGGCATCGGCGGCCTGCCAAAAGGCCGTATTGTTGAAATCTACGGTCCGGAA
TCGTCCGGTAAAACCACCCTGACCCTGTCGGTGATCGCCCAGGCACAGAAGATGGGCG
CCACCTGCGCCTTCGTCGACGCCGAGCACGCACTGGACCCGGAATACGCCGGCAAACT
GGGGGTCAACGTTGACGACCTGCTGGTTTCCCAGCCGGACACCGGCGAACAGGCGCTG
GAAATCACCGACATGCTGGTGCGCTCCAATGCCATCGACGTGATCGTGATCGACTCCG
TGGCGGCGCTGGTACCCAAGGCCGAGATCGAAGGCGAGATGGGCGACATGCACGTGG
GCCTGCAGGCTCGCCTGATGTCCCAGGCGCTGCGCAAGATCACCGGTAACATCAAGAA
CGCCAACTGCCTGGTGATCTTCATCAACCAGATCCGTATGAAAATCGGCGTGATGTTCG
GCAGCCCGGAAACCACCACCGGTGGTAACGCGCTGAAGTTCTACGCTTCGGTTCGTCT
GGACATCCGTCGTACTGGCGCGGTGAAGGAAGGTGACGAAGTCGTCGGTAGCGAAAC
CCGGGTCAAGATCGTCAAGAACAAGGTGGCTCCACCTTTCCGTCAAGCTGAGTTCCAG
ATCCTGTACGGCAAGGGTATCTACCTGAACGGCGAGATCATCGATCTGGGCGTGCTGC
ACGGTTTCCTCGAGAAGTCCGGTGCCTGGTACAGCTACCAGGGCAACAAGATCGGTCA
GGGCAAGGCCAACTCGGCCAAGTTCCTGCAGGACAATCCGGAAATCGGTAATGCCCTC
GAGAAGCAGATTCGCGACAAGCTGCTGGCTCCGAGCGGAGATACCAAGGCTCTGCCCG

TCAACGAGACCATCGATGACATGGCCGACGCGGATATCTGAATGAGCGAAGAAAACA
CGTACGACTCGAGCAGCATTAAAGTGCTGAAAGGTTTGGATGCCGTACGCAAACGTCC
CGGTATGTACATTGGTGACACCGACGATGGCAGCGGTCTGCACCATATGGTGTTCGAG
GTGGTCGACAACTCGATCGACGAAGCTCTGGCCGGCCACTGCGACGACATCAGCATCA
TCATCCACCCGGACGAATCCATTACCGTGCGTGACAACGGTCGCGGCATCCCGGTAGA
CGTGCATAAAGAAGAAGGCGTTTCCGCAGCCGAGGTCATCATGACCGTGCTGCACGCC
GGCGGTAAGTTCGACGACAACTCCTACAAGGTATCCGGCGGTCTGCACGGTGTGGGTG
TGTCGGTAGTGAACGCCCTGTCCGAAGAACTGGTGCTGACCGTTCGCCGCAGTGGCAA
GATCTGGGAACAGACCTACGTTCACGGTGTGCCTCAGGCGCCTATGGCAATTGTCGGT
GACAGCGAGACCACCGGTACCCAGATTCACTTCAAGGCTTCCAGCGAGACCTTCAAGA
ACATCCATTTCAGCTGGGACATCCTGGCCAAGCGGATTCGTGAACTGTCCTTCCTCAAC
TCCGGTGTCGGTATCGTTCTGAAGGACGAGCGCAGCGGCAAGGAAGAGCTGTTCAAGT
ACGAAGGCGGCCTGCGCGCATTCGTTGAATACCTGAACACCAACAAGACTGCGGTCAA
CCAGGTGTTCCACTTCAACGTGCAGCGTGAAGACGGCATCGGCGTGGAAATCGCCCTG
CAGTGGAACGACAGCTTCAACGAAAACCTGCAGTGCTTCACCAACAACATTCCGCAGC
GCGACGGCGGCACCCACCTGGTGGGCTTCCGCTCGGCACTGACGCGTAACCTGAACAA
CTACATCGAGCAGGAAGGTCTGGCGAAGAAGCACAAGGTGGCCACCACCGGTGACGA
TGCCCGCGAAGGCCTGACCGCGATCATTTCGGTCAAGGTGCCGGATCCGAAGTTCAGC
TCCCAGACCAAAGATAAGCTGGTGTCTTCCGAAGTGAAGACCGCAGTCGAACAGGAAA
TGGGCAAGTACTTCTCCGACTTCCTGCTGGAAAACCCGAACGAAGCCAAGCTGGTGGT
CGGCAAGATGCTCGACGCCGCCCGTGCCCGTGAAGCGGCGCGTAAGGCTCGTGAGATG
ACCCGCCGTAAAGGTGCGCTGGATATCGCCGGCCTGCCGGGCAAACTGGCGGACTGCC
AGGAAAAGGACCCTGCCCTTTCCGAACTCTACCTGGTGGAAGGTGACTCTGCTGGCGG
CTCCGCCAAGCAGGGACGCAACCGCAAGACCCAGGCGATTCTGCCGCTCAAGGGCAA
GATTCTTAACGTCGAGAAAGCGCGCTTCGACAAGATGATTTCCTCGCAAGAGGTCGGC
ACCTTGATCACTGCACTCGGCTGCGGCATCGGCCGCGAAGAGTACAACATCGACAAGC
TGCGTTATCACAACATCATCATCATGACCGACGCCGACGTCGACGGTTCGCACATCCGC
ACCCTGCTGCTGACTTTCTTCTTCCGTCAGCTGCCGGAGCTGATCGAGCGTGGCTACAT
CTACATCGCTCAACCGCCGCTGTACAAGGTCAAGAAAGGCAAGCAAGAGCAATACATC
AAAGACGACGACGCCATGGAAGAGTACATGACGCAGTCGGCCCTGGAAGATGCGAGC
CTGCACCTGAACGAAGAAGCACCGGGTATTTCCGGCGAGGCGCTGGAGCGCCTGGTGA
ACGACTTCCGCATGGTCATGAAAACCCTCAAGCGCCTGTCGCGCCTGTACCCTCAGGA
GCTGACCGAACACTTCATCTACCTGCCAGCCGTGAGCCTGGAGCAACTCTCCGATCAC
GCAGCGATGCAGGATTGGTTGGCCCAATATGAAGTCCGCCTGCGCACCGTCGAGAAGT
CCGGCCTGGTCTACAAGGCCAGCCTGCGTGAAGACCGTGAACGTAATGTCTGGCTGCC
AGAGGTCGAACTGATCTCCCACGGCCTGTCGAACTACGTCACCTTCAACCGCGACTTCT
TCGGCAGCAATGACTACAAGACCGTCGTCACCCTCGGCGCTCAACTGAGCTCCCTGCT
GGACGAAGGCGCTTATATTCAGCGTGGCGAACGCAAGAAGGCGGTGACCGAGTTCAA
GGAAGCCCTGGACTGGCTGATGACCGAAAGCACCAAGCGCCACACCATCCAGCGATAC
AAAGGTCTGGGCGAGATGAACCCGGATCAGCTGTGGGAAACCACCATGGACCCAAGC
GTGCGCCGCATGCTCAAGGTCACCATCGAAGACGCCATCGGCGCCGACCAGATCTTCA
ACACCCTGATGGGTGATGCGGTCGAGCCTCGTCGCGACTTCATCGAAAGCAACGCCCT
GGCGGTATCCAACCTGGACTTCTGAATGTCCGGAAAAGCGCAACAGCAGTCTCGCCTC
AAAGAGTTGATCAGCCGTGGTCGTGAGCAGGGTTACCTGACTTACGCGGAGGTCAACG
ACCACCTGCCGGAGGATATTTCAGATCCGGAACAGGTGGAAGACATCATCCGCATGAT
CAACGACATGGGGATCAACGTATTCGAGAGTGCTCCGGATGCGGATGCCCTTTTGTTG
GCCGAAGCCGATACCGACGAAGCAGCAGCTGAAGAAGCCGCCGCAGCGTTGGCGGCT
GTGGAAACCGACATTGGTCGCACTACCGACCCAGTGCGTATGTACATGCGCGAAATGG
GCACGGTAGAGCTGCTCACACGTGAAGGCGAAATCGAAATCGCCAAGCGTATCGAAG
AGGGCATCCGTGAAGTGATGGGCGCGATCGCGCACTTCCCTGGCACGGTTGAGCACAT
CCTCTCCGAATACACTCGCGTCACCACCGAAGGTGGCCGCCTGTCCGACGTCCTGAGC

GGTTACATCGACCCGGATGACGGCATTGCGCCGCCTGCCGCCGAAGTACCACCGCCTG
TCGATGCCAAGGCCGCGAAAGCGGACGACGACACCGACGACGATGACGCCGAAGCCA
GTGACGACGAAGAAGAAGCCGAAAGCGGTCCGGATCCGGTCATCGCAGCCCAGCGCT
TTGGCGCCGTTGCCGACCAGATGGAAATCACCCGCAAGGCGCTGAAAAAGCACGGTCG
CGAACACAAGCAAGCCCTGGCTGAAATGCTGGCCCTGGCTGAACTGTTCATGCCGATC
AAACTGGTTCCGAAGCAATTCGAAGGCCTGGTTGAACGTGTTCGTAGCGCCCTGGATC
GCCTGCGTCAGCAAGAGCGCGCGATCATGCAGCTCTGTGTTCGTGATGCCCGCATGCC
ACGCGCCGACTTCCTGCGCCAGTTCCCTGGCAATGAAGTGGACGAAAGCTGGTCCGAC
GCGCTGGCCAAAGGCAAGGCCAAGTACGCCGAAGCCATCGGCCGCCTGCAGCCGGAC
ATCATCCGTTGCCAGCAGAAGCTGACCGCGCTCGAGACCGAGACCGGCCTGACGATTG
CCGAGATCAAGGACATCAACCGTCGCATGTCGATCGGCGAGGCCAAGGCCCGTCGCGC
GAAGAAAGAGATGGTCGAAGCCAACTTGCGCCTGGTGATCTCCATCGCCAAGAAGTAC
ACCAACCGTGGCCTGCAGTTCCTCGACCTGATCCAGGAAGGCAACATCGGTTTGATGA
AAGCGGTAGACAAGTTCGAATACCGTCGCGGCTACAAGTTCTCGACTTATGCCACCTG
GTGGATCCGTCAGGCGATCACTCGCTCGATCGCCGACCAGGCCCGCACCATCCGTATTC
CGGTGCACATGATCGAGACGATCAACAAGCTCAACCGTATTTCCCGGCAGATGTTGCA
GGAAATGGGTCGCGAACCGACTCCGGAAGAGCTGGGCGAACGCATGGAAATGCCTGA
GGACAAGATCCGCAAGGTATTGAAGATCGCTAAAGAGCCGATCTCCATGGAAACCCCG
ATCGGTGATGACGAAGACTCCCATCTGGGTGACTTCATCGAAGACTCGACCATGCAGT
CGCCAATCGATGTCGCCACCGTTGAGAGCCTCAAGGAAGCGACTCGCGAAGTCCTCTC
CGGCCTCACTGCCCGTGAAGCCAAGGTACTGCGCATGCGCTTCGGCATCGACATGAAT
ACCGACCACACCCTTGAGGAAGTCGGTAAGCAGTTCGACGTTACCCGTGAGCGGATTC
GTCAGATCGAAGCCAAGGCGCTGCGCAAGCTGCGCCACCCGACGAGAAGCGAGCATC
TGCGCTCCTTCCTCGACGAGTAATTGACTAAGCCAGCCATACTCGCCCTTGCTGATGGC
AGCATTTTTCGCGGCGAAGCCATTGGAGCCGACGGTCAGACCGTTGGTGAGGTGGTGT
TTAACACCGCAATGACCGGCTATCAGGAAATCCTTACCGATCCTTCCTACGCCCAACAG
ATCGTTACCCTGACTTACCCGCACATCGGCAACACTGGCACCACGCCGGAAGACGCCG
AGTCCGATCGTGTCTGGTCGGCCGGTCTGGTGATTCGCGACCTGCCACTGGTTGCGAGC
AACTGGCGTAACACCCTGTCCCTGTCCGATTACCTGAAAGCCAACAATGTCGTGGCGA
TCGCCGGTATCGACACCCGTCGCCTGACGCGCATCCTGCGCGAGAAAGGCGCACAGAA
CGGCTGCATCATGGCCGGCGACAACATCTCCGACGAAGCGGCGATTGCTGCTGCACGC
GGCTTCCCTGGCCTGAAAGGCATGGATCTGGCGAAGGTCGTCAGCACCAAGGAAAGCT
ACGAGTGGCGCTCCAGCGTCTGGAGCCTGAAGACCGACAGTCATCCGACTATCGAGGC
TTCCGAGCTGCCTTACCACGTGGTTGCCTACGACTACGGCGTCAAGCTGAACATCCTGC
GCATGCTGGTCGAGCGCGGTTGCCGCGTGACCGTAGTGCCTGCGCAAACCCCGGCCAG
CGACGTCCTGGCACTCAAGCCTGACGGTGTGTTCCTGTCCAACGGTCCTGGCGACCCCG
AGCCTTGCGATTACGCCATCCAGGCGATCAAGGACGTGCTGGAAACCGAGATTCCGGT
CTTCGGTATCTGCCTGGGCCACCAACTGCTGGCACTGGCCTCCGGCGCCAAGACGGTG
AAAATGGGCCACGGCCACCACGGCGCCAACCACCCGGTCCAGGACCTGGACAGCGGT
GTGGTGATGATCACCAGCCAGAACCACGGTTTTGCGGTGGACGAAACCACCCTGCCGG
GCAACGTGCGGGCGATCCACAAGTCGCTGTTCGATGGCACCCTGCAAGGCATCGAGCG
TACCGACAAGAGCGCATTCAGCTTCCAGGGCCACCCTGAAGCGAGCCCGGGCCCGAAC
GATGTGGCGCCGCTGTTCGATCGTTTCATCAACGAGATGGCCAAGCGACGCTGAATGA
GTAGCGGACGTATCGTTCAAATCATCGGCGCCGTTATCGACGTGGAATTTCCACGCGA
CAGCGTACCGAGCATCTACGACGCCTTGAAGGTTCAAGGCGCCGAAACCACTCTGGAA
GTTCAGCAGCAGCTGGGCGACGGCGTGGTACGTACCATTGCGATGGGCTCCACCGAGG
GCTTGAAGCGCGGTCTGGACGTCAACAACACTGGCGCCGCCATCTCCGTACCGGTCGG
TAAAGCGACCCTGGGCCGGATCATGGACGTGCTGGGCAACCCGATCGACGAAGCTGGC
CCGATCGGCGAAGAAGAGCGTTGGGGCATTCACCGTCCTGCGCCGACCTTCGCTGAAC
AAGCTGGCGGCAACGACCTCCTGGAAACCGGCATCAAGGTTATCGACCTGGTTTGCCC
GTTCGCCAAGGGCGGTAAAGTCGGTCTGTTCGGTGGTGCCGGTGTGGGCAAAACCGTA

AACATGATGGAACTGATCCGTAACATCGCCATCGAGCACAGCGGTTATTCCGTGTTCG
CTGGTGTGGGTGAGCGTACTCGTGAGGGTAACGACTTCTACCACGAGATGAAGGATTC
CAACGTTCTGGACAAAGTGGCACTGGTATACGGCCAGATGAACGAGCCGCCGGGAAA
CCGTCTGCGCGTAGCTCTGACCGGCCTGACCATGGCCGAGAAGTTCCGTGACGAAGGT
AACGACGTTCTGCTGTTCGTCGACAACATCTATCGTTACACCCTGGCCGGTACCGAAGT
ATCCGCACTGCTGGGCCGTATGCCTTCGGCAGTAGGTTACCAGCCGACCCTGGCTGAA
GAGATGGGCGTTCTGCAAGAACGTATCACTTCGACCAAGCAAGGCTCGATCACCTCGA
TCCAAGCGGTATACGTACCTGCGGACGACTTGACCGACCCGTCGCCAGCGACCACCTT
CGCCCACTTGGACGCCACCGTCGTACTGTCCCGTGACATCGCTTCCCTGGGTATCTACC
CAGCGGTAGACCCACTGGATTCGACTTCCCGTCAGCTGGACCCGAACGTGATCGGCAA
CGAGCACTACGAAACCGCTCGCGGCGTTCAGTACGTGCTGCAGCGCTACAAAGAGCTG
AAGGACATCATTGCGATCCTGGGTATGGACGAACTGTCCGAAGCCGACAAGCAACTGG
TATCCCGCGCTCGTAAGATCCAGCGCTTCCTGTCGCAGCCGTTCTTCGTGGCTGAAGTC
TTCACTGGTTCTCCAGGCAAATACGTTTCCCTGAAAGACACCATCGCTGGCTTCAAAGG
CATCCTCAACGGTGACTACGACCACCTGCCAGAACAAGCGTTCTACATGGTCGGCGGC
ATCGAAGAAGCGATCGAGAAAGCCAAGAAACTGTAA

NCBI Reference Sequence: NZ_CP027722.1
Strain: C50
Chained genes: 16S rRNA, recA, gyrB, rpoD, carA, atpD
>NZ_CP027722_C50
GAACTGAAGAGTTTGATCATGGCTCAGATTGAACGCTGGCGGCAGGCCTAACACATGC
AAGTCGAGCGGTAGAGAGAAGCTTGCTTCTCTTGAGAGCGGCGGACGGGTGAGTAATG
CCTAGGAATCTGCCTGGTAGTGGGGGATAACGTTCGGAAACGGACGCTAATACCGCAT
ACGTCCTACGGGAGAAAGCAGGGGACCTTCGGGTCTTGCGCTATCAGATGAGCCTAGG
TCGGATTAGCTAGTTGGTGAGGTAATGGCTCACCAAGGCGACGATCCGTAACTGGTCT
GAGAGGATGATCAGTCACACTGGAACTGAGACACGGTCCAGACTCCTACGGGAGGCA
GCAGTGGGGAATATTGGACAATGGGCGAAAGCCTGATCCAGCCATGCCGCGTGTGTGA
AGAAGGTCTTCGGATTGTAAAGCACTTTAAGTTGGGAGGAAGGGTACTTACCTAATAC
GTGAGTATTTTGACGTTACCGACAGAATAAGCACCGGCTAACTCTGTGCCAGCAGCCG
CGGTAATACAGAGGGTGCAAGCGTTAATCGGAATTACTGGGCGTAAAGCGCGCGTAGG
TGGTTCGTTAAGTTGGATGTGAAATCCCCGGGCTCAACCTGGGAACTGCATCCAAAAC
TGGCGAGCTAGAGTATGGTAGAGGGTGGTGGAATTTCCTGTGTAGCGGTGAAATGCGT
AGATATAGGAAGGAACACCAGTGGCGAAGGCGACCACCTGGACTGATACTGACACTG
AGGTGCGAAAGCGTGGGGAGCAAACAGGATTAGATACCCTGGTAGTCCACGCCGTAA
ACGATGTCAACTAGCCGTTGGGAGCCTTGAGCTCTTAGTGGCGCAGCTAACGCATTAA
GTTGACCGCCTGGGGAGTACGGCCGCAAGGTTAAAACTCAAATGAATTGACGGGGGCC
CGCACAAGCGGTGGAGCATGTGGTTTAATTCGAAGCAACGCGAAGAACCTTACCAGGC
CTTGACATCCAATGAACTTTCCAGAGATGGATTGGTGCCTTCGGGAACATTGAGACAG
GTGCTGCATGGCTGTCGTCAGCTCGTGTCGTGAGATGTTGGGTTAAGTCCCGTAACGAG
CGCAACCCTTGTCCTTAGTTACCAGCACGTCATGGTGGGCACTCTAAGGAGACTGCCG
GTGACAAACCGGAGGAAGGTGGGGATGACGTCAAGTCATCATGGCCCTTACGGCCTGG
GCTACACACGTGCTACAATGGTCGGTACAGAGGGTTGCCAAGCCGCGAGGTGGAGCTA
ATCCCATAAAACCGATCGTAGTCCGGATCGCAGTCTGCAACTCGACTGCGTGAAGTCG
GAATCGCTAGTAATCGCGAATCAGAATGTCGCGGTGAATACGTTCCCGGGCCTTGTAC
ACACCGCCCGTCACACCATGGGAGTGGGTTGCACCAGAAGTAGCTAGTCTAACCTTCG
GGAGGACGGTTACCACGGTGTGATTCATGACTGGGGTGAAGTCGTAACAAGGTAGCCG
TAGGGGAACCTGCGGCTGGATCACCTCCTTAAATGGACGACAACAAGAAGAAAGCCTT
GGCTGCGGCCCTGGGTCAGATCGAACGTCAATTCGGCAAGGGTGCCGTAATGCGTATG
GGCGATCACGATCGCCAGGCGATCCCGGCCATTTCCACTGGCTCTCTGGGTCTGGACAT
CGCGCTCGGCATCGGCGGCCTGCCAAAAGGCCGTATTGTTGAAATCTACGGTCCGGAA

TCGTCCGGTAAAACCACCCTGACCCTGTCCGTGATTGCCCAGGCACAGAAGATGGGCG
CCACCTGCGCCTTCGTCGACGCCGAGCACGCACTGGACCCGGAATACGCCGGCAAACT
GGGGGTCAACGTTGACGACCTGCTGGTTTCCCAGCCGGACACCGGCGAACAGGCGCTG
GAAATCACCGACATGCTGGTGCGCTCCAATGCCATCGACGTGATCGTGATCGACTCCG
TGGCGGCACTGGTACCCAAGGCCGAGATCGAAGGCGAGATGGGCGACATGCACGTGG
GCCTGCAGGCCCGCCTGATGTCCCAGGCGCTGCGCAAGATCACCGGTAACATCAAGAA
CGCCAACTGTCTGGTGATCTTCATCAACCAGATCCGTATGAAAATCGGCGTGATGTTCG
GCAGCCCGGAAACCACCACCGGCGGTAACGCGCTGAAGTTCTACGCCTCGGTTCGTCT
GGACATCCGTCGTACTGGCGCGGTGAAGGAAGGCGACGAAGTCGTCGGTAGCGAAAC
CCGGGTCAAGATCGTCAAGAACAAGGTGGCTCCACCGTTCCGTCAGGCTGAATTCCAG
ATCCTGTACGGCAAGGGTATCTACCTGAACGGCGAGATCATCGATCTGGGCGTGCTGC
ACGGTTTCCTCGAGAAGTCCGGTGCCTGGTACAGCTACCAGGGCAACAAGATCGGTCA
GGGCAAGGCCAACTCGGCCAAGTTCCTGCAGGACAATCCGGAAATCGGCAATGCCCTC
GAGAAGCAGATTCGCGACAAGCTGCTGGCTCCAACCGCTGATGTCAAAGCTTCGCCGG
TCAACGAGACCATCGATGACATGGCTGACGCGGATATCTGAATGAGCGAAGAAAACA
CGTACGACTCGAGCAGCATTAAAGTGCTGAAAGGTTTGGATGCCGTACGCAAACGTCC
CGGTATGTACATTGGTGACACCGACGATGGCAGCGGTCTGCACCATATGGTGTTCGAG
GTGGTCGATAACTCGATCGACGAAGCTCTGGCCGGCCATTGCGACGACATCAGCATCA
TCATCCACCCGGACGAATCCATTACCGTGCGTGACAACGGTCGCGGCATCCCGGTAGA
CGTGCATAAAGAAGAAGGCGTTTCCGCGGCCGAGGTCATCATGACCGTACTGCACGCC
GGCGGTAAGTTCGACGATAACTCCTACAAAGTATCCGGCGGTCTGCACGGTGTGGGTG
TGTCGGTAGTGAACGCCCTGTCCGAAGAACTGGTCCTGACCGTTCGCCGCAGCGGAAA
GATCTGGGAACAGACCTACGTTCACGGTGTGCCTCAGGCGCCTATGGCGATCGTCGGT
GACAGCGAAACCACCGGTACCCAGATTCACTTCAAGGCGTCCAGCGAGACCTTCAAGA
ACATCCATTTCAGCTGGGACATCCTGGCCAAGCGGATTCGTGAACTGTCCTTCCTCAAC
TCCGGTGTCGGTATCGTTCTGAAGGACGAACGCAGTGGCAAGGAAGAGCTGTTCAAGT
ACGAAGGCGGCCTGCGTGCGTTCGTTGAATACCTGAACACCAACAAGACCGCGGTCAA
CCAGGTGTTCCACTTCAATGTGCAGCGTGAAGATGGCATCGGCGTGGAAATCGCCCTG
CAGTGGAACGACAGCTTCAACGAAAACCTGCAGTGCTTCACCAACAACATTCCGCAGC
GCGATGGCGGCACCCACTTGGTGGGCTTCCGTTCGGCACTGACGCGTAACCTGAACAA
CTACATCGAACAGGAAGGTCTGGCGAAGAAGCACAAGGTCGCCACCACCGGTGACGA
TGCCCGCGAAGGCCTGACCGCGATCATTTCGGTCAAGGTGCCGGATCCGAAGTTCAGC
TCCCAGACCAAAGACAAGCTGGTGTCTTCCGAAGTGAAGACCGCGGTTGAACAGGAAA
TGGGCAAGTACTTCTCCGACTTCCTGCTGGAAAACCCGAACGAAGCCAAGCTGGTGGT
CGGCAAGATGCTCGACGCCGCCCGTGCCCGTGAAGCGGCGCGTAAGGCTCGTGAGATG
ACCCGCCGTAAAGGCGCGCTGGATATCGCCGGCCTGCCGGGCAAACTGGCGGACTGCC
AGGAAAAAGACCCTGCCCTTTCCGAACTCTACCTGGTGGAAGGTGACTCTGCTGGCGG
CTCCGCCAAGCAGGGACGCAACCGTAAGACCCAGGCGATTCTGCCGCTCAAGGGCAAG
ATCCTTAACGTCGAGAAAGCGCGCTTCGACAAGATGATTTCCTCGCAAGAGGTCGGCA
CCTTGATCACTGCACTCGGTTGCGGCATCGGCCGCGAAGAGTACAACATCGACAAGCT
GCGTTATCACAACATCATCATCATGACCGACGCTGACGTCGACGGTTCGCACATCCGTA
CCCTGCTGCTGACCTTCTTCTTCCGTCAGCTGCCGGAGCTGATCGAGCGTGGCTACATC
TACATCGCTCAACCGCCGCTGTACAAGGTCAAGAAAGGCAAGCAAGAGCAATACATCA
AAGACGACGACGCCATGGAAGAGTACATGACGCAGTCGGCCCTGGAAGATGCGAGCC
TGCACCTGAACGAAGAAGCACCGGGTATTTCCGGCGAGGCGCTGGAGCGCCTGGTGAA
CGACTTCCGCATGGTCATGAAAACCCTCAAGCGTCTGTCGCGCCTGTACCCTCAGGAGC
TGACCGAGCACTTCATCTACCTGCCGGCCGTGAGCCTGGAGCAACTCTCCGATCACGC
GGCCATGCAGGATTGGCTGGCCCAATATGAAGTCCGCCTGCGCACCGTCGAGAAGTCC
GGCCTGGTCTACAAGGCCAGCCTGCGTGAAGACCGTGAACGTAATGTCTGGCTGCCAG
AGGTCGAACTGATCTCCCACGGCCTGTCGAACTACGTCACCTTCAACCGCGACTTCTTC
GGTAGCAATGACTACAAGACCGTCGTTACCCTCGGCGCTCAACTGAGCTCCCTGCTGG

ACGAAGGCGCTTATATTCAGCGTGGCGAACGCAAGAAGGCGGTGACCGAGTTCAAGG
AAGCCCTGGACTGGCTGATGACCGAAAGCACCAAGCGCCACACCATCCAGCGATACAA
AGGTCTGGGCGAGATGAACCCGGATCAGCTGTGGGAAACCACCATGGACCCAAGCGT
GCGCCGTATGCTCAAGGTCACGATTGAAGATGCCATCGGCGCCGACCAGATCTTCAAC
ACCCTGATGGGGGATGCGGTCGAGCCTCGTCGCGACTTCATCGAAAGCAACGCCCTGG
CGGTATCCAATCTGGACTTCTGAATGTCCGGAAAAGCGCAACAGCAGTCTCGCCTCAA
AGAGTTGATCAGCCGTGGTCGTGAGCAGGGTTACCTGACTTACGCGGAGGTCAACGAC
CACCTGCCGGAGGATATTTCAGATCCGGAACAGGTGGAAGACATCATCCGCATGATCA
ACGACATGGGGATCAACGTATTCGAGAGTGCTCCGGATGCGGATGCCCTTTTGTTGGC
CGAAGCCGATACCGACGAAGCAGCAGCTGAAGAAGCCGCCGCAGCGTTGGCGGCTGT
GGAAACCGACATTGGTCGCACTACCGACCCCGTGCGTATGTACATGCGCGAAATGGGA
ACGGTAGAGCTGCTCACACGTGAAGGCGAAATCGAAATCGCCAAGCGTATCGAAGAG
GGCATCCGTGAAGTGATGGGCGCGATCGCGCACTTCCCTGGCACGGTTGAGCACATCC
TCTCCGAATACACTCGCGTCACCACCGAAGGTGGCCGCCTGTCCGACGTCCTGAGCGG
TTACATCGACCCGGACGACGGCATTGCGCCGCCTGCCGCCGAAGTACCACCGCCTGTC
GATGCCAAGGCTGCAAAAGCGGACGACGACACCGACGACGATGACGCCGAAGCCAGT
GACGACGAAGAAGAAGCCGAAAGCGGTCCGGATCCGGTCATCGCAGCCCAGCGCTTT
GGCGCCGTTGCCGACCAGATGGAAATCACCCGCAAGGCGCTGAAGAAGCACGGTCGC
GAACACAAGCAAGCCCTGGCCGAAATGCTGGCCCTGGCTGAACTGTTCATGCCGATCA
AACTGGTTCCGAAGCAATTCGAAGGCCTGGTTGAACGTGTTCGTAGCGCCCTGGATCG
CCTGCGTCAGCAAGAGCGCGCGATCATGCAGCTCTGTGTTCGTGATGCCCGCATGCCA
CGCGCCGACTTCCTGCGCCAGTTCCCTGGCAATGAAGTGGACGAAAGCTGGTCCGACG
CGCTGGCCAAAGGCAAGGCCAAGTACGCCGAAGCCATCGGCCGCCTGCAGCCGGACA
TCATCCGTTGCCAGCAGAAGCTGACCGCGCTCGAGACCGAGACCGGCCTGACGATCGC
CGAGATCAAGGACATCAACCGTCGCATGTCGATCGGCGAGGCCAAGGCCCGTCGCGCG
AAGAAAGAGATGGTCGAAGCCAACCTGCGTCTGGTGATCTCCATCGCCAAGAAGTACA
CCAACCGTGGCTTGCAATTCCTCGACCTGATCCAGGAAGGCAACATCGGTTTGATGAA
AGCGGTAGACAAGTTCGAATACCGTCGCGGCTACAAATTCTCGACTTATGCCACCTGG
TGGATCCGTCAGGCGATCACTCGCTCGATCGCCGACCAGGCCCGCACCATCCGTATTCC
GGTGCACATGATCGAGACGATCAACAAGCTCAACCGTATTTCCCGGCAGATGTTGCAG
GAAATGGGTCGCGAACCGACTCCGGAAGAGCTGGGCGAACGCATGGAAATGCCTGAG
GACAAGATCCGCAAGGTATTGAAGATCGCTAAAGAGCCGATCTCCATGGAAACCCCGA
TCGGTGATGACGAAGACTCCCATCTGGGTGACTTCATCGAAGACTCGACCATGCAGTC
GCCAATCGATGTCGCCACCGTTGAGAGCCTTAAAGAAGCGACTCGCGAAGTACTCTCC
GGCCTCACTGCCCGTGAAGCCAAGGTACTGCGCATGCGCTTCGGCATCGACATGAATA
CCGACCACACCCTCGAGGAAGTCGGTAAGCAGTTCGACGTTACCCGTGAGCGGATTCG
TCAGATCGAAGCCAAGGCGCTGCGCAAGCTGCGCCACCCGACGCGAAGCGAGCACCT
GCGCTCCTTCCTCGACGAGTAATTGACTAAGCCAGCCATACTCGCCCTTGCTGATGGCA
GCATTTTTCGCGGCGAAGCCATTGGAGCCGACGGTCAAACCGTTGGTGAGGTGGTGTT
TAACACCGCAATGACCGGCTATCAGGAAATCCTTACCGATCCTTCCTACGCCCAACAG
ATCGTTACCCTGACTTACCCGCATATCGGCAATACCGGCACCACGCCGGAAGACGCCG
AGTCCGATCGTGTCTGGTCGGCCGGTCTGGTGATTCGCGACCTGCCTCTGGTTGCGAGC
AACTGGCGTAACACCCTGTCCCTGTCCGACTACCTGAAAGCCAACAATGTCGTGGCGA
TCGCCGGTATCGACACCCGTCGCCTGACGCGCATCCTGCGCGAGAAAGGTGCGCAGAA
CGGCTGCATCATGGCCGGCGACAATATCTCCGACGAAGCGGCGATTGCCGCTGCACGC
GGCTTCCCGGGCCTGAAAGGCATGGATCTGGCGAAGGTCGTCAGTACCAAGGAAAGCT
ACGAGTGGCGCTCCAGTGTCTGGAACCTGAAGACCGACAGTCATCCGACCATCGAAGC
TTCCGAGCTGCCTTACCACGTGGTTGCCTACGACTACGGCGTCAAGCTGAACATCCTGC
GCATGCTGGTCGAACGCGGTTGCCGCGTGACCGTGGTGCCTGCGCAAACCCCGGCCAG
CGAAGCTCTGGCGCTCAAGCCTGACGGTGTGTTCCTGTCCAACGGCCCTGGCGACCCC
GAGCCTTGCGATTACGCCATCCAGGCGATCAAGGACGTGCTGGAGACCGAGATTCCGG

TCTTCGGTATCTGTCTGGGCCACCAACTGCTGGCACTGGCCGCCGGCGCCAAGACAGT
GAAGATGGGCCACGGCCACCACGGCGCCAACCACCCGGTCCAGGACCTGGACAGCGG
TGTGGTGATGATCACCAGCCAGAACCACGGTTTTGCGGTGGACGAAGCCACCCTGCCG
GGCAACGTGCGGGCGATCCACAAGTCGCTGTTCGACGGCACCCTGCAAGGCATCGAGC
TGACCGACAAGAGCGCATTCAGCTTCCAGGGCCACCCTGAAGCGAGCCCGGGCCCGAA
CGATGTGGCGCCGCTGTTCGATCGTTTCATCAACGAGATGGCCAAGCGACGCTGAATG
AGTAGCGGACGTATCGTTCAAATCATCGGCGCCGTTATCGACGTGGAATTTCCACGCG
ACAGCGTACCGAGCATCTACGACGCCTTGAAGGTTCAAGGCGCCGAAACCACTCTGGA
AGTTCAGCAGCAGCTGGGCGACGGCGTGGTACGTACCATTGCGATGGGCTCCACCGAG
GGCTTGAAGCGCGGTCTGGACGTCAACAACACTGGCGCAGCCATCTCCGTACCGGTCG
GTAAAGCGACCCTGGGCCGGATCATGGACGTACTGGGCAACCCGATCGACGAAGCTGG
CCCGATCGGTGAAGAAGAGCGTTGGGGCATTCACCGTCCTGCGCCGACCTTCGCTGAA
CAAGCTGGCGGCAACGACCTGCTGGAAACCGGCATCAAGGTTATCGACCTGGTTTGCC
CGTTCGCCAAGGGCGGTAAAGTCGGTCTGTTCGGTGGTGCCGGTGTGGGCAAAACCGT
AAACATGATGGAACTGATCCGTAACATCGCCATCGAGCACAGCGGTTATTCCGTGTTC
GCCGGTGTGGGTGAGCGTACTCGTGAGGGTAACGACTTCTACCACGAGATGAAGGATT
CCAACGTTCTGGACAAAGTGGCACTGGTATACGGCCAGATGAACGAGCCGCCGGGAA
ACCGTCTGCGCGTAGCTCTGACCGGCCTGACCATGGCCGAGAAGTTCCGTGACGAAGG
TAACGACGTTCTGCTGTTCGTCGACAACATCTATCGTTACACCCTGGCCGGTACCGAAG
TATCCGCACTGCTGGGCCGTATGCCTTCGGCAGTAGGTTACCAGCCGACCCTGGCTGAA
GAGATGGGCGTTCTGCAAGAACGTATCACTTCGACCAAGCAAGGCTCGATCACCTCGA
TCCAAGCGGTATACGTACCTGCGGACGACTTGACCGACCCGTCGCCAGCGACCACCTT
CGCCCACTTGGACGCCACCGTCGTTCTGTCCCGTGACATCGCTTCCCTGGGTATCTACC
CAGCGGTAGACCCACTGGACTCGACTTCCCGTCAGCTGGACCCGAACGTGATCGGCAC
CGAGCACTACGAAACCGCTCGTGGCGTTCAGTACGTGCTGCAGCGCTACAAAGAGCTG
AAGGACATCATTGCGATCCTGGGTATGGACGAACTGTCCGAAGCCGACAAGCAACTGG
TATCCCGCGCTCGTAAGATCCAGCGCTTCCTGTCGCAGCCGTTCTTCGTGGCTGAAGTC
TTCACTGGTTCTCCAGGCAAATACGTTTCCCTGAAAGACACCATCGCTGGCTTCAAAGG
CATCCTCAACGGTGACTACGACCATCTGCCAGAACAAGCGTTCTACATGGTTGGTGGC
ATCGAAGAAGCGATCGAGAAAGCCAAGAAACTGTAA

NCBI Reference Sequence: NZ_CM001559.1
Strain: 30-84
Chained genes: 16S rRNA, recA, gyrB, rpoD, carA, atpD
>NZ_CM001559_30_84
GAACTGAAGAGTTTGATCATGGCTCAGATTGAACGCTGGCGGCAGGCCTAACACATGC
AAGTCGAGCGGTAGAGAGAAGCTTGCTTCTCTTGAGAGCGGCGGACGGGTGAGTAATG
CCTAGGAATCTGCCTGGTAGTGGGGGATAACGTCCGGAAACGGACGCTAATACCGCAT
ACGTCCTACGGGAGAAAGCAGGGGACCTTCGGGCCTTGCGCTATCAGATGAGCCTAGG
TCGGATTAGCTAGTTGGTGAGGTAATGGCTCACCAAGGCGACGATCCGTAACTGGTCT
GAGAGGATGATCAGTCACACTGGAACTGAGACACGGTCCAGACTCCTACGGGAGGCA
GCAGTGGGGAATATTGGACAATGGGCGAAAGCCTGATCCAGCCATGCCGCGTGTGTGA
AGAAGGTCTTCGGATTGTAAAGCACTTTAAGTTGGGAGGAAGGGTACTTACCTAATAC
GTGAGTATTTTGACGTTACCGACAGAATAAGCACCGGCTAACTCTGTGCCAGCAGCCG
CGGTAATACAGAGGGTGCAAGCGTTAATCGGAATTACTGGGCGTAAAGCGCGCGTAGG
TGGTTCGTTAAGTTGGATGTGAAATCCCCGGGCTCAACCTGGGAACTGCATCCAAAAC
TGGCGAGCTAGAGTATGGTAGAGGGTGGTGGAATTTCCTGTGTAGCGGTGAAATGCGT
AGATATAGGAAGGAACACCAGTGGCGAAGGCGACCACCTGGACTGATACTGACACTG
AGGTGCGAAAGCGTGGGGAGCAAACAGGATTAGATACCCTGGTAGTCCACGCCGTAA
ACGATGTCAACTAGCCGTTGGGAGCCTTGAGCTCTTAGTGGCGCAGCTAACGCATTAA
GTTGACCGCCTGGGGAGTACGGCCGCAAGGTTAAAACTCAAATGAATTGACGGGGGCC

CGCACAAGCGGTGGAGCATGTGGTTTAATTCGAAGCAACGCGAAGAACCTTACCAGGC
CTTGACATCCAATGAACTTTCCAGAGATGGATTGGTGCCTTCGGGAACATTGAGACAG
GTGCTGCATGGCTGTCGTCAGCTCGTGTCGTGAGATGTTGGGTTAAGTCCCGTAACGAG
CGCAACCCTTGTCCTTAGTTACCAGCACGTAATGGTGGGCACTCTAAGGAGACTGCCG
GTGACAAACCGGAGGAAGGTGGGGATGACGTCAAGTCATCATGGCCCTTACGGCCTGG
GCTACACACGTGCTACAATGGTCGGTACAGAGGGTTGCCAAGCCGCGAGGTGGAGCTA
ATCCCACAAAACCGATCGTAGTCCGGATCGCAGTCTGCAACTCGACTGCGTGAAGTCG
GAATCGCTAGTAATCGCGAATCAGAATGTCGCGGTGAATACGTTCCCGGGCCTTGTAC
ACACCGCCCGTCACACCATGGGAGTGGGTTGCACCAGAAGTAGCTAGTCTAACCTTCG
GGAGGACGGTTACCACGGTGTGATTCATGACTGGGGTGAAGTCGTAACAAGGTAGCCG
TAGGGGAACCTGCGGCTGGATCACCTCCTTAAATGGACGACAACAAGAAGAAAGCCTT
GGCTGCGGCCCTGGGTCAGATCGAACGTCAATTCGGCAAGGGTGCCGTAATGCGTATG
GGCGATCACGACCGCCAGGCGATCCCGGCCATTTCCACTGGCTCTCTGGGTCTGGACAT
CGCACTCGGCATCGGCGGCCTGCCAAAGGGCCGTATTGTTGAAATCTACGGTCCGGAA
TCGTCCGGTAAAACCACCCTGACCCTGTCCGTGATTGCCCAGGCACAGAAGATGGGCG
CCACCTGCGCCTTCGTCGACGCCGAGCACGCACTGGACCCGGAATACGCCGGCAAGCT
GGGGGTCAACGTTGACGACCTGCTGGTTTCCCAGCCGGACACCGGTGAACAGGCACTG
GAAATCACCGATATGCTGGTGCGCTCCAATGCCATTGACGTGATCGTGATCGACTCCGT
GGCGGCACTGGTGCCCAAGGCCGAGATCGAAGGCGAGATGGGCGACATGCACGTGGG
CCTGCAGGCCCGCCTGATGTCCCAGGCGCTGCGCAAGATCACCGGCAACATCAAGAAC
GCCAACTGCCTGGTGATCTTCATCAACCAGATCCGTATGAAAATCGGCGTGATGTTCGG
CAGCCCGGAAACCACCACCGGTGGTAACGCGCTGAAGTTCTACGCTTCGGTTCGTCTG
GATATCCGTCGTACTGGCGCGGTGAAGGAAGGTGACGAAGTCGTCGGTAGCGAAACCC
GGGTCAAGATCGTCAAGAACAAGGTGGCTCCACCGTTCCGCCAGGCTGAATTCCAGAT
CCTGTACGGCAAGGGTATCTACCTGAACGGCGAGATCATCGATCTGGGCGTGCTGCAC
GGTTTCCTCGAGAAGTCCGGTGCCTGGTACAGCTACCAGGGCAACAAGATCGGTCAGG
GCAAGGCCAACTCGGCCAAGTTCCTGCAGGACAACCCGGAAATCGGTAACGCCCTCGA
GAAGCAGATTCGCGACAAGCTGCTGGCTCCGACTGCTGATGTCAAAGCTTCGCCGGTC
AACGAGACCATCGATGACATGGCCGACGCGGATATCTGAATGAGCGAAGAAAACACG
TACGACTCGAGCAGCATTAAAGTGCTGAAAGGTTTGGATGCCGTACGCAAACGTCCCG
GTATGTACATTGGTGACACCGACGATGGCAGCGGTCTGCACCATATGGTGTTCGAGGT
GGTCGATAACTCGATCGACGAAGCTCTGGCCGGCCATTGTGACGACATCAGCATCATC
ATCCACCCGGACGAATCCATTACCGTGCGTGACAACGGTCGCGGCATCCCGGTAGACG
TGCATAAAGAAGAAGGCGTTTCCGCAGCCGAGGTCATCATGACTGTGCTGCACGCCGG
CGGTAAGTTCGACGATAACTCCTACAAAGTATCCGGCGGTCTGCACGGTGTGGGTGTG
TCGGTAGTAAACGCCCTGTCCGAAGAGCTGGTCTTGACCGTTCGCCGCAGTGGCAAGA
TCTGGGAACAGACCTACGTTCACGGTGTGCCTCAGGCGCCTATGGCGATCGTCGGTGA
CAGCGAAACCACCGGTACCCAGATTCACTTCAAGGCTTCCAGCGAGACCTTCAAGAAC
ATCCACTTCAGCTGGGACATCCTGGCCAAGCGGATTCGTGAACTGTCCTTCCTCAACTC
CGGTGTCGGTATCGTTCTGAAGGACGAGCGCAGCGGCAAGGAAGAGCTGTTCAAGTAC
GAAGGCGGCTTGCGTGCGTTCGTTGAATACCTGAACACCAACAAGACTGCGGTCAACC
AGGTGTTCCACTTCAACGTGCAGCGTGAAGATGGCATCGGCGTGGAAATCGCCCTGCA
GTGGAACGACAGCTTCAACGAGAACCTGCAGTGCTTCACCAACAACATTCCGCAGCGC
GACGGCGGCACCCACCTGGTGGGCTTCCGTTCGGCGCTGACGCGTAACCTGAACAACT
ACATCGAGCAGGAAGGCCTGGCGAAGAAGCACAAGGTCGCCACCACCGGTGACGATG
CCCGCGAAGGCCTGACCGCGATCATTTCGGTCAAGGTGCCGGATCCGAAGTTCAGCTC
CCAGACCAAAGACAAGCTGGTGTCTTCCGAAGTGAAGACCGCGGTCGAACAGGAAAT
GGGTAAGTACTTCTCCGACTTCCTGCTGGAAAACCCGAACGAAGCCAAACTGGTGGTC
GGCAAGATGCTCGACGCCGCCCGTGCCCGTGAAGCGGCGCGTAAAGCCCGTGAGATGA
CCCGTCGTAAAGGCGCGCTGGATATCGCCGGCCTGCCGGGCAAACTGGCGGACTGCCA
GGAAAAAGACCCTGCCCTTTCCGAACTCTACCTGGTGGAAGGTGACTCTGCTGGCGGC

TCCGCCAAGCAGGGACGCAACCGTAAGACCCAGGCGATCCTGCCGCTCAAGGGCAAG
ATCCTCAACGTCGAGAAAGCGCGCTTCGACAAGATGATTTCCTCGCAAGAGGTCGGCA
CCTTGATCACTGCACTCGGTTGCGGCATCGGCCGCGAAGAGTACAACATCGACAAGCT
GCGTTATCACAACATCATCATCATGACCGACGCCGACGTCGACGGTTCGCACATCCGT
ACCCTGCTGCTGACCTTCTTCTTCCGTCAGTTGCCGGAGCTGATCGAGCGTGGCTACAT
CTACATCGCTCAACCGCCGCTGTACAAGGTCAAGAAAGGCAAGCAAGAGCAATACATC
AAAGACGACGACGCCATGGAAGAGTACATGACGCAGTCGGCCCTGGAGGATGCGAGC
CTGCACCTGAACGAAGAAGCACCGGGTATTTCCGGCGAGGCGCTGGAGCGTCTGGTGA
ACGACTTCCGCATGGTCATGAAGACCCTCAAGCGTCTGTCGCGCCTGTACCCTCAAGA
GCTGACCGAGCACTTCATCTACCTGCCGGCCGTGAGCCTGGAGCAACTCTCCGATCAC
GCGGCCATGCAGGATTGGCTGGCCCAATATGAAGTCCGCCTGCGCACCGTCGAGAAGT
CCGGCCTGGTCTACAAGGCCAGCCTGCGTGAAGACCGTGAACGTAATGTGTGGCTGCC
AGAGGTCGAACTGATCTCCCACGGCCTGTCGAACTACGTCACCTTCAACCGCGACTTCT
TCGGCAGCAATGACTACAAGACCGTCGTCACCCTCGGTGCTCAACTGAGCTCCCTGCTG
GACGAAGGCGCTTATATTCAGCGCGGCGAGCGCAAGAAGGCGGTGACCGAGTTCAAG
GAAGCCCTGGACTGGCTGATGACCGAAAGCACCAAGCGCCACACCATCCAGCGATACA
AAGGTCTGGGCGAGATGAACCCGGATCAGTTGTGGGAAACCACCATGGACCCAAGCGT
GCGCCGCATGCTCAAGGTCACCATCGAAGACGCCATCGGCGCCGACCAGATCTTCAAC
ACCCTGATGGGTGATGCGGTCGAGCCTCGTCGTGACTTCATCGAAAGCAACGCCCTGG
CGGTATCCAACCTGGACTTCTGAATGTCCGGAAAAGCGCAACAGCAGTCTCGCCTCAA
AGAGTTGATCAGCCGTGGTCGTGAGCAGGGTTACCTGACTTACGCGGAGGTCAACGAC
CACCTGCCGGAGGATATTTCAGATCCGGAACAGGTGGAAGACATCATCCGCATGATCA
ACGACATGGGGATCAACGTATTCGAGAGTGCTCCGGATGCGGATGCCCTTTTGTTGGC
CGAAGCCGATACCGACGAAGCAGCAGCTGAAGAAGCCGCCGCAGCGTTGGCGGCTGT
GGAAACCGACATTGGTCGCACTACCGACCCAGTGCGTATGTACATGCGCGAAATGGGT
ACGGTAGAGCTGCTCACACGTGAAGGCGAAATCGAAATCGCCAAGCGTATCGAAGAG
GGCATCCGTGAAGTGATGGGCGCGATCGCGCACTTCCCTGGCACGGTTGAGCACATCC
TCTCCGAATACACTCGCGTCACCACCGAAGGTGGTCGCCTGTCCGACGTCCTGAGCGGT
TACATCGACCCGGACGACGGCATCGCGCCGCCTGCCGCCGAAGTACCACCGCCTGTCG
ATGCCAAGGCCGCGAAAGCGGACGACGACACCGACGACGATGACGCCGAAGCCAGTG
ACGACGAAGAAGAAGCCGAAAGCGGTCCGGATCCGGTCATCGCAGCCCAGCGCTTTG
GCGCCGTTGCCGACCAGATGGAAATCACCCGCAAGGCGCTGAAAAAGCACGGTCGTG
AACACAAGCAAGCCCTGGCTGAAATGCTGGCCCTGGCTGAACTGTTCATGCCGATCAA
ACTGGTTCCGAAGCAATTCGAAGGCCTGGTTGAACGCGTTCGTAGCGCCCTGGATCGC
CTGCGTCAGCAAGAGCGCGCGATCATGCAACTCTGTGTTCGTGATGCTCGCATGCCAC
GCGCCGACTTCCTGCGCCAGTTCCCTGGCAATGAAGTGGACGAAAGCTGGTCCGACGC
ACTGGCCAAAGGCAAGGCCAAGTACGCCGAAGCCATCGGTCGCCTGCAACCGGACATC
ATCCGTTGCCAGCAGAAGCTGACCGCGCTCGAGACCGAGACCGGCCTGACGATCGCCG
AGATCAAGGACATCAACCGTCGCATGTCGATCGGCGAAGCCAAGGCCCGTCGCGCGAA
GAAAGAGATGGTCGAAGCCAACTTGCGTCTGGTGATCTCCATCGCCAAGAAGTACACC
AACCGTGGCCTGCAGTTCCTCGACCTGATCCAGGAAGGCAACATCGGTTTGATGAAAG
CGGTAGACAAGTTCGAATACCGTCGCGGCTACAAGTTCTCGACTTATGCGACCTGGTG
GATCCGTCAGGCGATCACTCGCTCGATCGCCGACCAGGCCCGTACCATCCGTATTCCGG
TGCACATGATCGAGACGATCAACAAGCTCAACCGTATTTCCCGGCAGATGCTGCAGGA
AATGGGTCGCGAACCGACCCCGGAAGAGCTGGGCGAACGCATGGAAATGCCTGAGGA
CAAGATCCGCAAGGTATTGAAGATCGCTAAAGAGCCGATCTCCATGGAAACCCCGATC
GGTGATGACGAAGACTCCCATCTGGGCGACTTCATCGAAGACTCGACCATGCAGTCGC
CAATCGATGTCGCCACCGTTGAGAGCCTCAAGGAAGCGACTCGCGAAGTACTCTCCGG
CCTCACTGCCCGTGAAGCCAAGGTACTGCGCATGCGCTTCGGCATCGACATGAATACC
GACCACACCCTCGAGGAAGTCGGTAAGCAGTTCGACGTTACCCGTGAGCGGATTCGCC
AGATCGAAGCCAAGGCGCTGCGCAAGCTGCGCCACCCGACGAGAAGCGAGCACCTGC

GCTCCTTCCTCGACGAGTAATTGACTAAGCCAGCCATACTCGCCCTTGCTGATGGCAGC
ATTTTTCGCGGCGAAGCCATTGGAGCCGACGGTCAAACCGTTGGTGAGGTGGTGTTTA
ACACCGCAATGACCGGCTATCAGGAAATCCTTACCGATCCTTCCTACGCCCAACAGAT
CGTTACCCTGACTTACCCGCATATCGGCAATACCGGCACCACGCCGGAAGACGCCGAG
TCCGATCGTGTCTGGTCGGCCGGTCTGGTGATTCGCGACCTGCCACTGTTTGCGAGCAA
CTGGCGTAACACCCTGTCCCTGTCCGATTACCTGAAAGCCAACAACGTTGTGGCGATCG
CCGGTATCGACACCCGTCGCCTGACGCGCATCCTGCGCGAGAAAGGCGCGCAGAACGG
CTGCATCATGGCCGGCGACAACATTTCCGACGAAGCGGCGATTGCCGCAGCGCGCGGC
TTCCCTGGCCTGAAAGGCATGGATCTGGCGAAGGTCGTCAGCACCAAGGAAAGCTACG
AGTGGCGCTCCAGCGTCTGGAGCCTGAAGACCGACAGTCATCCGACCATCGAGGCTTC
CGAGCTGCCGTACCACGTGGTCGCCTACGATTACGGCGTCAAGCTGAACATCCTGCGC
ATGCTGGTCGAGCGCGGTTGCCGCGTGACCGTAGTGCCTGCGCAAACTCCGGCCAGCG
ACGTCCTGGCACTCAAGCCTGACGGTGTGTTCCTGTCCAACGGCCCTGGCGACCCCGA
GCCTTGCGATTACGCCATCCAGGCGATCAAGGATGTGCTGGAAACCGAGATCCCGGTC
TTCGGTATCTGCCTGGGCCACCAACTGCTGGCACTGGCCTCTGGCGCCAAGACGGTGA
AGATGGGCCACGGCCACCACGGTGCCAACCACCCGGTCCAGGACCTGGACAGCGGTGT
AGTGATGATCACCAGCCAGAACCACGGTTTTGCGGTGGACGAAGCCACCCTGCCAGGC
AACGTGCGGGCGATCCACAAATCGCTGTTCGACGGCACCCTGCAAGGCATCGAACGTA
CCGACAAGAGCGCATTCAGCTTCCAGGGCCACCCTGAAGCGAGCCCGGGCCCGAACGA
TGTGGCGCCGCTGTTCGATCGTTTCATCAACGAGATGGCCAAGCGACGCTGAATGAGT
AGCGGACGTATCGTTCAAATCATCGGCGCCGTTATCGACGTGGAATTTCCACGCGACA
GCGTACCGAGCATCTACGACGCCTTGAAGGTTCAAGGCGCCGAAACCACTCTGGAAGT
TCAGCAGCAGCTGGGCGACGGCGTGGTACGTACCATTGCGATGGGCTCCACCGAGGGC
TTGAAGCGCGGTCTGGACGTCAACAACACTGGCGCAGCCATCTCCGTACCGGTCGGTA
AAGCGACCCTGGGCCGGATCATGGACGTACTGGGCAACCCGATCGACGAAGCTGGTCC
GATCGGCGAAGAAGAGCGTTGGGGCATTCACCGTCCTGCGCCGACCTTCGCTGAACAA
GCTGGCGGCAACGACCTGCTGGAAACCGGCATCAAGGTTATCGACCTGGTTTGCCCGT
TCGCCAAGGGCGGTAAAGTCGGTCTGTTCGGTGGTGCCGGTGTGGGCAAAACCGTAAA
CATGATGGAACTGATCCGTAACATCGCCATCGAGCACAGCGGTTATTCCGTGTTCGCCG
GTGTGGGTGAGCGTACTCGTGAGGGTAACGACTTCTACCACGAGATGAAGGATTCCAA
CGTTCTGGACAAAGTGGCACTGGTATACGGCCAGATGAACGAGCCGCCGGGAAACCGT
CTGCGCGTAGCTCTGACCGGCCTGACCATGGCCGAGAAGTTCCGTGACGAAGGTAACG
ACGTTCTGCTGTTCGTCGACAACATCTATCGTTACACCCTGGCCGGTACCGAAGTATCC
GCACTGCTGGGCCGTATGCCTTCCGCAGTAGGTTACCAGCCGACCCTGGCTGAAGAGA
TGGGCGTTCTGCAAGAACGTATCACTTCGACCAAGCAAGGCTCGATCACCTCGATCCA
AGCGGTATACGTACCTGCGGACGACTTGACCGACCCGTCGCCAGCGACCACCTTCGCC
CACTTGGACGCCACCGTCGTACTGTCCCGTGACATCGCTTCCCTGGGTATCTACCCAGC
GGTAGATCCACTGGACTCGACTTCCCGTCAGCTGGACCCGAACGTGATCGGCAACGAG
CACTATGAAACCGCTCGCGGCGTTCAGTACGTGCTGCAGCGCTACAAAGAGCTGAAGG
ACATCATTGCGATCCTGGGTATGGACGAACTGTCCGAAGCCGACAAGCAACTGGTATC
CCGCGCTCGTAAGATCCAGCGCTTCCTGTCGCAGCCGTTCTTCGTGGCTGAAGTCTTCA
CTGGTTCTCCAGGCAAATACGTTTCCCTGAAAGACACCATCGCTGGCTTCAAAGGCATC
CTCAACGGTGACTACGACCACCTGCCAGAACAAGCGTTCTACATGGTCGGCGGCATCG
AAGAAGCGATCGAGAAAGCCAAGAAACTGTAA

NCBI Reference Sequence: NZ_CP027712.1
Strain: DSM50083
Chained genes: 16S rRNA, recA, gyrB, rpoD, carA, atpD
>NZ_CP027712_DSM50083
GAACTGAAGAGTTTGATCATGGCTCAGATTGAACGCTGGCGGCAGGCCTAACACATGC
AAGTCGAGCGGTAGAGAGGTGCTTGCACCTCTTGAGAGCGGCGGACGGGTGAGTAATG

CCTAGGAATCTGCCTGGTAGTGGGGGATAACGTTCGGAAACGGACGCTAATACCGCAT
ACGTCCTACGGGAGAAAGCAGGGGACCTTCGGGCCTTGCGCTATCAGATGAGCCTAGG
TCGGATTAGCTAGTTGGTGAGGTAATGGCTCACCAAGGCGACGATCCGTAACTGGTCT
GAGAGGATGATCAGTCACACTGGAACTGAGACACGGTCCAGACTCCTACGGGAGGCA
GCAGTGGGGAATATTGGACAATGGGCGAAAGCCTGATCCAGCCATGCCGCGTGTGTGA
AGAAGGTCTTCGGATTGTAAAGCACTTTAAGTTGGGAGGAAGGGTACTTACCTAATAC
GTGAGTATTTTGACGTTACCGACAGAATAAGCACCGGCTAACTCTGTGCCAGCAGCCG
CGGTAATACAGAGGGTGCAAGCGTTAATCGGAATTACTGGGCGTAAAGCGCGCGTAGG
TGGTTCGTTAAGTTGGATGTGAAATCCCCGGGCTCAACCTGGGAACTGCATCCAAAAC
TGGCGAGCTAGAGTATGGTAGAGGGTGGTGGAATTTCCTGTGTAGCGGTGAAATGCGT
AGATATAGGAAGGAACACCAGTGGCGAAGGCGACCACCTGGACTGATACTGACACTG
AGGTGCGAAAGCGTGGGGAGCAAACAGGATTAGATACCCTGGTAGTCCACGCCGTAA
ACGATGTCAACTAGCCGTTGGGAGCCTTGAGCTCTTAGTGGCGCAGCTAACGCATTAA
GTTGACCGCCTGGGGAGTACGGCCGCAAGGTTAAAACTCAAATGAATTGACGGGGGCC
CGCACAAGCGGTGGAGCATGTGGTTTAATTCGAAGCAACGCGAAGAACCTTACCAGGC
CTTGACATCCAATGAACTTTCCAGAGATGGATTGGTGCCTTCGGGAACATTGAGACAG
GTGCTGCATGGCTGTCGTCAGCTCGTGTCGTGAGATGTTGGGTTAAGTCCCGTAACGAG
CGCAACCCTTGTCCTTAGTTACCAGCACGTAATGGTGGGCACTCTAAGGAGACTGCCG
GTGACAAACCGGAGGAAGGTGGGGATGACGTCAAGTCATCATGGCCCTTACGGCCTGG
GCTACACACGTGCTACAATGGTCGGTACAGAGGGTTGCCAAGCCGCGAGGTGGAGCTA
ATCCCACAAAACCGATCGTAGTCCGGATCGCAGTCTGCAACTCGACTGCGTGAAGTCG
GAATCGCTAGTAATCGCGAATCAGAATGTCGCGGTGAATACGTTCCCGGGCCTTGTAC
ACACCGCCCGTCACACCATGGGAGTGGGTTGCACCAGAAGTAGCTAGTCTAACCTTCG
GGAGGACGGTTACCACGGTGTGATTCATGACTGGGGTGAAGTCGTAACAAGGTAGCCG
TAGGGGAACCTGCGGCTGGATCACCTCCTTAAATGGACGACAACAAGAAGAAAGCCTT
GGCTGCGGCCCTGGGTCAGATCGAACGTCAATTCGGCAAGGGTGCCGTAATGCGTATG
GGCGATCACGACCGCCAGGCGATCCCGGCCATTTCCACTGGCTCTCTGGGTCTGGACAT
CGCACTCGGCATCGGCGGCCTGCCAAAGGGCCGTATTGTCGAAATCTACGGTCCGGAA
TCGTCCGGTAAAACCACCCTGACCTTGTCCGTGATTGCCCAGGCACAGAAGATGGGCG
CCACTTGCGCCTTCGTCGACGCCGAGCACGCACTGGACCCGGAATACGCCGGCAAGCT
GGGGGTCAACGTTGACGACCTGCTGGTTTCCCAGCCGGACACCGGTGAACAGGCACTG
GAAATCACCGACATGTTGGTGCGCTCCAATGCCATCGACGTGATCGTGATCGACTCCGT
GGCAGCACTGGTGCCCAAGGCCGAGATCGAAGGCGAGATGGGCGACATGCACGTGGG
CCTGCAGGCCCGCCTGATGTCCCAGGCGCTGCGCAAGATCACCGGCAACATCAAGAAC
GCCAACTGCCTGGTGATCTTCATCAACCAGATCCGTATGAAAATCGGCGTGATGTTCGG
CAGCCCGGAAACCACCACCGGTGGTAACGCGCTGAAGTTCTACGCTTCGGTTCGCCTG
GATATCCGTCGTACTGGCGCGGTGAAGGAAGGTGACGAAGTCGTCGGTAGCGAAACCC
GGGTCAAGATCGTCAAGAACAAGGTGGCTCCACCGTTCCGCCAGGCTGAATTCCAGAT
CCTGTACGGCAAGGGTATCTACCTGAACGGCGAGATCATCGATCTGGGCGTGCTGCAC
GGTTTCCTCGAGAAGTCTGGTGCCTGGTACAGCTACCAGGGCAACAAGATCGGTCAGG
GCAAGGCCAACTCGGCCAAGTTCCTGCAGGACAACCCGGAAATCGGTAATGCCCTCGA
GAAGCAGATTCGCGACAAGCTGCTGGCTCCGACCGCTGATGTCAAAGCTTCGCCGGTC
AACGAGACCATCGATGACATGGCCGACGCGGATATCTGAATGAGCGAAGAAAACACG
TACGACTCGAGCAGCATTAAAGTGCTGAAAGGTTTGGATGCCGTACGCAAACGTCCCG
GTATGTACATTGGTGACACCGACGATGGCAGCGGTCTGCACCATATGGTGTTCGAGGT
GGTCGATAACTCGATCGACGAAGCTCTGGCCGGCCATTGCGACGACATCAGCATCATC
ATCCACCCGGACGAATCCATTACCGTGCGCGACAACGGTCGCGGCATCCCGGTAGACG
TGCATAAAGAAGAAGGCGTTTCCGCAGCCGAGGTCATCATGACCGTGCTGCACGCCGG
CGGTAAGTTCGACGATAACTCCTACAAAGTATCCGGCGGTCTGCACGGTGTGGGTGTG
TCGGTAGTGAACGCCCTGTCCGAAGAACTGGTCTTGACCGTTCGCCGCAGTGGCAAGA
TCTGGGAACAGACCTACGTTCACGGTGTGCCTCAGGCGCCTATGGCTATCGTCGGTGAC

AGCGAAACCACCGGTACCCAGATTCACTTCAAGGCTTCCAGCGAAACCTTCAAGAACA
TCCACTTCAGCTGGGACATCCTGGCCAAGCGGATTCGTGAACTGTCCTTCCTCAACTCC
GGTGTTGGTATCGTTCTGAAGGACGAGCGCAGCGGCAAGGAAGAACTGTTCAAGTACG
AAGGCGGCTTGCGTGCGTTCGTTGAATACCTGAATACCAACAAGACTGCGGTCAACCA
GGTGTTCCACTTCAATGTGCAGCGTGAAGACGGCATTGGCGTGGAAATCGCACTGCAG
TGGAACGACAGCTTCAACGAAAACCTGCAGTGCTTCACCAACAACATTCCGCAGCGCG
ACGGCGGCACCCACCTGGTGGGCTTCCGTTCGGCACTGACGCGTAACCTGAACAACTA
CATCGAACAGGAAGGCCTGGCGAAGAAGCACAAGGTCGCCACCACCGGTGACGATGC
CCGCGAAGGCCTGACCGCGATCATTTCGGTCAAGGTGCCGGATCCGAAGTTCAGCTCC
CAGACCAAAGACAAGCTGGTGTCTTCCGAAGTGAAGACCGCGGTCGAACAGGAAATG
GGCAAGTACTTCTCCGACTTCCTGCTGGAAAACCCGAACGAAGCCAAGCTGGTGGTCG
GCAAGATGCTCGACGCCGCCCGTGCCCGTGAAGCGGCGCGTAAAGCCCGTGAGATGAC
CCGTCGTAAAGGCGCGCTGGATATCGCCGGCCTGCCGGGCAAACTGGCGGACTGCCAG
GAAAAAGACCCTGCTCTTTCCGAACTCTACCTGGTGGAAGGTGACTCTGCTGGCGGCTC
CGCCAAGCAGGGACGCAACCGTAAGACCCAGGCGATCCTGCCGCTCAAGGGCAAGAT
CCTCAACGTCGAGAAAGCGCGCTTCGACAAGATGATTTCCTCGCAAGAGGTCGGCACC
TTGATCACTGCACTCGGTTGCGGCATCGGCCGCGAAGAGTACAACATCGACAAGCTGC
GTTATCACAACATCATCATCATGACCGACGCCGACGTCGACGGTTCGCACATCCGTACC
CTCCTGCTGACCTTCTTTTTCCGTCAGTTGCCGGAGCTGATCGAGCGTGGCTACATCTA
CATCGCTCAACCGCCGCTGTACAAGGTCAAGAAAGGCAAGCAAGAGCAATACATCAA
AGACGACGACGCCATGGAAGAGTACATGACGCAGTCGGCCCTGGAAGATGCGAGCCT
GCACCTGAACGAAGAAGCACCGGGTATTTCCGGCGAGGCGCTGGAGCGCCTGGTGAAC
GACTTCCGCATGGTCATGAAGACCCTCAAGCGTCTGTCGCGCCTGTACCCTCAAGAGCT
GACCGAGCACTTCATCTACCTGCCGGCCGTGAGCCTGGAGCAACTCTCCGATCACGCG
GCGATGCAGGATTGGCTGGCCCAGTATGAAGTCCGCCTGCGCACCGTCGAGAAGTCCG
GCCTGGTCTACAAGGCCAGTCTGCGTGAAGACCGTGAACGTAATGTCTGGCTGCCAGA
GGTCGAACTGATCTCCCACGGCCTGTCGAACTACGTCACCTTCAACCGCGATTTCTTCG
GCAGTAATGACTACAAGACCGTCGTCACCCTCGGGGCTCAACTGAGCTCCCTGCTGGA
CGAAGGCGCTTATATTCAGCGCGGCGAGCGCAAGAAGGCAGTGACCGAGTTCAAGGA
AGCCCTGGACTGGCTGATGACCGAAAGCACCAAACGCCACACCATCCAGCGATATAAA
GGTCTGGGTGAGATGAACCCGGACCAGCTGTGGGAAACCACCATGGACCCAAGCGTGC
GCCGCATGCTCAAGGTCACCATCGAAGACGCCATCGGCGCCGACCAGATCTTCAACAC
CCTGATGGGTGATGCGGTCGAGCCTCGTCGCGACTTCATCGAAAGCAACGCCCTGGCG
GTATCCAACCTGGACTTCTGAATGTCCGGAAAAGCGCAACAGCAGTCTCGCCTCAAAG
AGTTGATCAGCCGTGGTCGTGAGCAGGGTTACCTGACTTACGCGGAGGTCAACGACCA
CCTGCCGGAGGATATTTCAGATCCGGAACAGGTGGAAGACATCATCCGCATGATCAAC
GACATGGGGATCAACGTATTCGAGAGTGCTCCGGATGCGGATGCCCTTTTGTTGGCCG
AAGCCGATACCGACGAAGCAGCAGCTGAAGAAGCCGCCGCAGCGTTGGCGGCAGTGG
AAACCGACATTGGTCGCACTACCGACCCAGTGCGTATGTACATGCGCGAAATGGGTAC
GGTAGAGCTGCTCACACGTGAAGGCGAAATCGAAATCGCCAAGCGTATCGAAGAGGG
CATCCGTGAAGTGATGGGCGCGATCGCGCACTTCCCTGGCACGGTTGAGCACATCCTCT
CCGAATACACTCGCGTCACCACCGAAGGTGGTCGCCTGTCCGACGTCCTGAGCGGTTA
CATCGACCCGGACGACGGCATCGCGCCGCCTGCCGCCGAAGTACCACCGCCTGTCGAT
GCCAAGGCCGCGAAAGCGGACGACGACACCGACGACGATGACGCCGAAGCCAGTGAC
GACGAAGAAGAAGCCGAAAGCGGTCCGGATCCGGTCATCGCAGCCCAGCGCTTTGGC
GCCGTTGCCGACCAGATGGAAATCACCCGCAAGGCGCTGAAAAAGCACGGTCGTGAA
CACAAGCAAGCCCTGGCTGAAATGCTGGCCCTGGCTGAACTGTTCATGCCGATCAAAC
TGGTTCCGAAGCAATTCGAAGGCCTGGTTGAACGCGTTCGTAGCGCCCTGGATCGCCT
GCGTCAGCAAGAGCGCGCGATCATGCAGCTCTGTGTTCGTGATGCTCGCATGCCACGC
GCCGACTTCCTGCGCCAGTTCCCTGGCAATGAAGTGGACGAAAGCTGGTCCGACGCAC
TGGCCAAAGGCAAGGCCAAGTACGCCGAAGCCATCGGTCGCCTGCAGCCGGACATCAT

TCGTTGCCAGCAGAAGCTGACCGCGCTCGAGACCGAGACCGGCCTGACGATCGCCGAG
ATCAAGGACATCAACCGTCGCATGTCGATCGGCGAGGCCAAGGCCCGTCGCGCGAAGA
AAGAGATGGTCGAAGCCAACTTGCGTCTGGTGATCTCCATCGCCAAGAAGTACACCAA
CCGTGGCCTGCAGTTCCTCGACCTGATCCAGGAAGGCAACATCGGCTTGATGAAAGCG
GTAGACAAGTTCGAATACCGTCGCGGCTACAAGTTCTCGACTTATGCCACCTGGTGGAT
CCGTCAGGCGATCACTCGCTCGATCGCCGACCAGGCCCGCACCATCCGTATTCCGGTGC
ACATGATCGAGACGATCAACAAGCTCAACCGTATTTCCCGGCAGATGCTGCAGGAAAT
GGGCCGCGAACCGACCCCGGAAGAGCTGGGCGAACGCATGGAAATGCCTGAGGACAA
GATCCGCAAGGTATTGAAGATCGCTAAAGAGCCGATCTCCATGGAAACCCCGATCGGT
GATGACGAAGACTCCCATCTGGGCGACTTCATCGAAGACTCGACCATGCAGTCGCCAA
TCGATGTCGCTACCGTTGAGAGCCTCAAGGAAGCGACTCGCGAAGTACTCTCCGGCCT
CACTGCCCGTGAAGCCAAGGTACTGCGCATGCGCTTCGGTATCGACATGAATACCGAC
CATACCCTCGAGGAAGTCGGTAAGCAGTTCGACGTTACCCGTGAGCGGATTCGCCAGA
TCGAAGCCAAGGCGCTGCGCAAGCTGCGCCACCCGACGAGAAGCGAGCACCTGCGCTC
CTTCCTCGACGAGTAATTGACTAAGCCAGCCATACTCGCCCTTGCTGATGGCAGCATTT
TTCGCGGCGAAGCCATTGGAGCCGACGGTCAAACCGTTGGTGAGGTGGTGTTTAACAC
CGCAATGACCGGCTATCAGGAAATCCTTACCGATCCTTCCTACGCCCAACAGATCGTTA
CCCTGACTTACCCGCATATCGGCAATACCGGCACCACGCCGGAAGACGCCGAGTCCGA
TCGTGTCTGGTCGGCCGGTCTGGTGATTCGCGACCTGCCACTGGTTGCGAGCAACTGGC
GTAACACCCTGTCCCTGTCCGATTACCTGAAAGCCAACAATGTTGTGGCGATCGCCGGT
ATCGACACCCGTCGCCTGACGCGCATCCTGCGCGAGAAAGGCGCGCAGAACGGCTGCA
TCATGGCCGGTGACAATATCTCCGACGAAGCGGCGATTGCCGCAGCGCGCGGCTTCCC
TGGCCTGAAAGGCATGGATCTGGCGAAGGTCGTCAGCACCAAGGAAAGCTACGAGTG
GCGCTCCAGCGTCTGGAGCCTGAAGACCGACAGTCATCCGACCATCGAGGCTTCCGAG
CTGCCGTACCACGTGGTTGCCTACGACTACGGCGTCAAGCTGAACATCCTGCGCATGCT
GGTCGAGCGCGGTTGCCGCGTGACCGTAGTGCCTGCGCAAACTCCGGCCAGCGACGTC
CTGGCACTCAAGCCTGATGGCGTGTTCCTGTCCAACGGCCCTGGCGACCCCGAGCCTTG
CGATTACGCCATCCAGGCGATCAAGGACGTGCTGGAAACCGAGATCCCGGTCTTCGGT
ATCTGCCTGGGCCACCAACTGCTGGCACTGGCCTCCGGCGCCAAGACGGTGAAGATGG
GCCACGGCCACCACGGTGCCAACCACCCGGTCCAGGACCTGGACAGCGGTGTAGTGAT
GATCACCAGCCAGAACCACGGTTTTGCGGTGGACGAAGCCACCCTGCCAGGCAACGTG
CGGGCGATCCACAAATCGCTGTTCGACGGTACCCTGCAAGGCATCGAACGTACCGACA
AGAGCGCATTCAGCTTCCAGGGCCACCCTGAAGCGAGCCCGGGCCCGAACGATGTGGC
GCCGCTGTTCGATCGTTTCATCAACGAGATGGCCAAGCGACGCTGAATGAGTAGCGGA
CGTATCGTTCAAATCATCGGCGCCGTTATCGACGTGGAATTTCCACGCGACAGCGTACC
GAGCATCTACGACGCCTTGAAGGTTCAAGGCGCCGAAACCACTCTGGAAGTTCAGCAG
CAGCTGGGCGACGGCGTGGTACGTACCATTGCGATGGGCTCCACCGAGGGCTTGAAGC
GCGGTCTGGACGTCAACAACACTGGCGCAGCCATCTCCGTACCGGTCGGTAAAGCGAC
CCTGGGCCGGATCATGGACGTACTGGGCAACCCGATCGACGAAGCTGGCCCGATCGGC
GAAGAAGAGCGTTGGGGCATTCACCGTCCTGCGCCGTCCTTCGCTGAACAAGCTGGTG
GCAACGACCTCCTGGAAACCGGCATCAAGGTTATCGACCTGGTTTGCCCGTTCGCCAA
GGGCGGTAAAGTCGGTCTGTTCGGTGGTGCCGGTGTGGGCAAGACCGTAAACATGATG
GAACTGATCCGTAACATCGCCATCGAGCACAGCGGTTATTCCGTGTTCGCCGGTGTGG
GTGAGCGTACTCGTGAGGGTAACGACTTCTACCACGAGATGAAGGATTCCAACGTTCT
GGACAAAGTGGCACTGGTATACGGCCAGATGAACGAGCCGCCGGGAAACCGTCTGCG
CGTAGCTCTGACCGGCCTGACCATGGCCGAGAAGTTCCGTGACGAAGGTAACGACGTT
CTGCTGTTCGTCGACAACATCTATCGTTACACCCTGGCCGGTACCGAAGTATCCGCACT
GCTGGGCCGTATGCCTTCCGCAGTAGGTTACCAGCCGACCCTGGCTGAAGAGATGGGC
GTTCTGCAAGAACGTATCACTTCGACCAAGCAAGGCTCGATCACCTCGATCCAAGCGG
TATACGTACCTGCGGACGACTTGACCGACCCGTCGCCAGCGACCACCTTCGCCCACTTG
GACGCCACAGTCGTACTGTCCCGTGACATCGCTTCCCTGGGTATCTACCCAGCGGTAGA

TCCACTGGACTCGACTTCCCGTCAGCTGGACCCGAACGTGATCGGCAACGAGCACTAC
GAAACCGCTCGCGGCGTTCAGTACGTGCTGCAGCGCTACAAAGAGCTGAAGGACATCA
TTGCGATCCTGGGTATGGACGAACTGTCCGAAGCCGACAAGCAACTGGTATCCCGCGC
TCGTAAGATCCAGCGCTTCCTGTCGCAGCCGTTCTTCGTGGCTGAAGTCTTCACTGGTT
CTCCAGGCAAATACGTTTCCCTGAAAGACACCATCGCTGGCTTCAAAGGCATCCTCAA
CGGTGACTACGACCACCTGCCAGAACAAGCGTTCTACATGGTCGGCGGCATCGAAGAA
GCGATCGAGAAAGCCAAGAAACTGTAA

NCBI Reference Sequence: NZ_CP027717.1
Strain: PCM2210
Chained genes: 16S rRNA, recA, gyrB, rpoD, carA, atpD
>NZ_CP027717_PCM2210
GAACTGAAGAGTTTGATCATGGCTCAGATTGAACGCTGGCGGCAGGCCTAACACATGC
AAGTCGAGCGGTAGAGAGAAGCTTGCTTCTCTTGAGAGCGGCGGACGGGTGAGTAATG
CCTAGGAATCTGCCTGGTAGTGGGGGATAACGTCCGGAAACGGACGCTAATACCGCAT
ACGTCCTACGGGAGAAAGCAGGGGACCTTCGGGCCTTGCGCTATCAGATGAGCCTAGG
TCGGATTAGCTAGTTGGTGAGGTAATGGCTCACCAAGGCGACGATCCGTAACTGGTCT
GAGAGGATGATCAGTCACACTGGAACTGAGACACGGTCCAGACTCCTACGGGAGGCA
GCAGTGGGGAATATTGGACAATGGGCGAAAGCCTGATCCAGCCATGCCGCGTGTGTGA
AGAAGGTCTTCGGATTGTAAAGCACTTTAAGTTGGGAGGAAGGGTACTTACCTAATAC
GTGAGTATTTTGACGTTACCGACAGAATAAGCACCGGCTAACTCTGTGCCAGCAGCCG
CGGTAATACAGAGGGTGCAAGCGTTAATCGGAATTACTGGGCGTAAAGCGCGCGTAGG
TGGTTCGTTAAGTTGGATGTGAAATCCCCGGGCTCAACCTGGGAACTGCATCCAAAAC
TGGCGAGCTAGAGTATGGTAGAGGGTGGTGGAATTTCCTGTGTAGCGGTGAAATGCGT
AGATATAGGAAGGAACACCAGTGGCGAAGGCGACCACCTGGACTGATACTGACACTG
AGGTGCGAAAGCGTGGGGAGCAAACAGGATTAGATACCCTGGTAGTCCACGCCGTAA
ACGATGTCAACTAGCCGTTGGGAGCCTTGAGCTCTTAGTGGCGCAGCTAACGCATTAA
GTTGACCGCCTGGGGAGTACGGCCGCAAGGTTAAAACTCAAATGAATTGACGGGGGCC
CGCACAAGCGGTGGAGCATGTGGTTTAATTCGAAGCAACGCGAAGAACCTTACCAGGC
CTTGACATCCAATGAACTTTCCAGAGATGGATTGGTGCCTTCGGGAACATTGAGACAG
GTGCTGCATGGCTGTCGTCAGCTCGTGTCGTGAGATGTTGGGTTAAGTCCCGTAACGAG
CGCAACCCTTGTCCTTAGTTACCAGCACGTTATGGTGGGCACTCTAAGGAGACTGCCGG
TGACAAACCGGAGGAAGGTGGGGATGACGTCAAGTCATCATGGCCCTTACGGCCTGGG
CTACACACGTGCTACAATGGTCGGTACAGAGGGTTGCCAAGCCGCGAGGTGGAGCTAA
TCCCATAAAACCGATCGTAGTCCGGATCGCAGTCTGCAACTCGACTGCGTGAAGTCGG
AATCGCTAGTAATCGCGAATCAGAATGTCGCGGTGAATACGTTCCCGGGCCTTGTACA
CACCGCCCGTCACACCATGGGAGTGGGTTGCACCAGAAGTAGCTAGTCTAACCTTCGG
GAGGACGGTTACCACGGTGTGATTCATGACTGGGGTGAAGTCGTAACAAGGTAGCCGT
AGGGGAACCTGCGGCTGGATCACCTCCTTAAATGGACGACAACAAGAAGAAAGCCTTG
GCTGCGGCCCTGGGTCAGATCGAACGTCAATTCGGCAAGGGTGCCGTAATGCGTATGG
GCGATCACGACCGCCAGGCGATCCCGGCCATTTCCACTGGCTCTCTGGGCCTGGACATC
GCGCTCGGCATCGGCGGCCTGCCAAAAGGCCGTATTGTTGAAATCTACGGTCCGGAAT
CGTCCGGTAAAACCACCCTGACCCTGTCGGTGATTGCCCAGGCACAGAAGATGGGCGC
CACCTGCGCCTTCGTCGACGCCGAGCACGCACTGGACCCGGAATACGCCGGCAAACTG
GGGGTCAACGTTGACGACCTGCTGGTTTCCCAGCCGGACACCGGCGAACAGGCGCTGG
AAATCACCGACATGCTGGTGCGTTCCAATGCCATCGACGTGATCGTGATCGACTCCGTG
GCGGCACTGGTACCCAAGGCCGAGATCGAAGGCGAGATGGGCGACATGCACGTGGGC
CTGCAGGCCCGCCTGATGTCCCAGGCGCTGCGCAAGATCACCGGTAACATCAAGAACG
CCAACTGCCTGGTGATCTTCATCAACCAGATCCGTATGAAAATCGGCGTGATGTTCGGC
AGCCCGGAAACCACCACCGGCGGTAACGCGCTGAAGTTCTACGCCTCGGTTCGTCTGG
ACATCCGTCGTACTGGCGCGGTGAAGGAAGGCGACGAAGTCGTCGGTAGCGAAACCC

GGGTCAAGATCGTCAAGAACAAGGTGGCTCCACCGTTCCGTCAGGCTGAATTCCAGAT
CCTGTACGGCAAGGGTATCTACCTGAACGGCGAGATCATCGATCTGGGCGTGCTGCAC
GGTTTCCTCGAGAAGTCCGGTGCCTGGTACAGCTACCAGGGCAACAAGATCGGTCAGG
GCAAGGCCAACTCGGCCAAGTTCCTGCAGGACAATCCGGAAATCGGCAATGCCCTCGA
GAAGCAGATTCGCGACAAGCTGCTGGCTCCAACCGCTGATGTCAAAGCTTCGCCGGTC
AACGAGACCATCGATGACATGGCTGACGCGGATATCTGAATGAGCGAAGAAAACACG
TACGACTCGAGCAGCATTAAAGTGCTGAAAGGTTTGGATGCCGTACGCAAACGTCCCG
GTATGTACATTGGTGACACCGACGATGGCAGCGGTCTGCACCATATGGTGTTCGAGGT
GGTCGATAACTCGATCGACGAAGCTCTGGCCGGCCACTGCGACGACATCAGCATCATC
ATCCACCCGGACGAATCCATTACCGTGCGTGACAACGGTCGCGGCATCCCGGTAGACG
TGCATAAAGAAGAAGGCGTTTCCGCGGCCGAGGTCATCATGACTGTGCTGCACGCCGG
CGGTAAGTTCGACGACAACTCCTACAAAGTATCCGGCGGTCTGCACGGTGTGGGTGTG
TCGGTAGTGAACGCCCTGTCCGAAGAACTGGTCCTGACCGTTCGCCGCAGTGGCAAGA
TCTGGGAACAGACCTACGTTCACGGTGTGCCTCAGGCGCCTATGGCGATCGTCGGTGA
CAGTGAAACCACCGGTACCCAGATTCACTTCAAGGCTTCCAGCGAGACCTTCAAGAAC
ATCCATTTCAGCTGGGACATCCTGGCCAAGCGGATTCGTGAACTGTCCTTCCTCAACTC
CGGTGTCGGTATCGTTCTGAAGGACGAGCGCAGCGGCAAGGAAGAACTGTTCAAGTAC
GAAGGCGGTCTGCGTGCGTTCGTTGAATACCTGAACACCAACAAGACCGCGGTCAACC
AGGTGTTCCACTTCAATGTGCAGCGTGAAGATGGCATCGGCGTGGAAATCGCCCTGCA
GTGGAACGACAGCTTCAACGAAAACCTGCAGTGCTTCACCAACAACATTCCGCAGCGC
GACGGCGGCACCCACCTGGTGGGCTTCCGTTCGGCACTGACGCGTAACCTGAACAACT
ACATCGAACAGGAAGGTCTGGCGAAGAAGCACAAGGTCGCCACCACCGGTGACGATG
CCCGCGAAGGCCTGACCGCAATCATTTCGGTCAAGGTGCCGGATCCGAAGTTCAGCTC
CCAGACCAAAGACAAGCTGGTGTCTTCCGAAGTGAAGACCGCGGTCGAACAGGAAAT
GGGCAAGTACTTCTCCGACTTCCTGCTGGAAAACCCGAACGAAGCCAAGCTGGTGGTC
GGCAAGATGCTCGACGCCGCCCGTGCCCGTGAAGCGGCGCGTAAGGCTCGCGAGATGA
CCCGCCGTAAAGGTGCGCTGGATATCGCCGGCCTGCCGGGCAAACTGGCGGACTGCCA
GGAAAAAGACCCTGCCCTTTCCGAACTCTACCTGGTGGAAGGTGACTCTGCTGGCGGC
TCCGCCAAGCAGGGACGCAACCGTAAGACCCAGGCGATTCTGCCGCTCAAGGGCAAG
ATCCTTAACGTCGAGAAAGCGCGTTTCGACAAGATGATTTCCTCGCAAGAGGTCGGCA
CCTTGATCACTGCACTCGGTTGCGGCATCGGCCGCGAAGAGTACAACATCGACAAGCT
GCGTTATCACAACATCATCATCATGACCGACGCCGACGTCGACGGTTCGCACATCCGT
ACCCTGCTGCTGACCTTCTTCTTCCGTCAGCTGCCGGAGCTGATCGAGCGTGGCTACAT
CTACATCGCTCAACCGCCGCTGTACAAGGTCAAGAAAGGCAAGCAAGAGCAATACATC
AAAGACGACGACGCCATGGAAGAGTACATGACGCAGTCGGCCCTGGAAGATGCGAGC
CTGCACCTGAACGAAGACACACCGGGTATTTCCGGCGAGGCGCTGGAGCGCCTGGTGA
ACGACTTCCGCATGGTCATGAAAACCCTCAAGCGTCTGTCGCGCCTGTACCCTCAGGA
GCTGACCGAGCACTTCATCTACCTGCCGGCCGTGAGCCTGGAGCAGCTCTCCGATCAC
GCGGCCATGCAGGATTGGCTGGCCCAATATGAAGTCCGCCTGCGCACCGTCGAGAAGT
CCGGCCTGGTCTACAAGGCCAGCCTGCGTGAAGACCGTGAACGTAATGTCTGGCTGCC
AGAGGTCGAACTGATCTCCCACGGCCTGTCGAACTACGTCACCTTCAACCGCGACTTCT
TCGGCAGCAATGACTACAAGACCGTCGTCACACTCGGCGCTCAACTGAGCTCCCTGCT
GGACGAAGGCGCTTATATTCAGCGTGGCGAACGCAAGAAGGCAGTGACCGAGTTCAA
GGAAGCCCTGGACTGGCTGATGACCGAAAGCACCAAGCGCCACACCATCCAGCGATAC
AAAGGTCTGGGCGAGATGAACCCGGATCAGCTGTGGGAAACCACCATGGACCCAAGC
GTGCGCCGTATGCTCAAGGTCACCATCGAAGACGCCATCGGCGCCGACCAGATCTTCA
ACACCCTGATGGGTGATGCGGTCGAGCCTCGTCGCGACTTCATCGAAAGCAACGCCCT
GGCGGTATCCAACTTGGACTTCTGAATGTCCGGAAAAGCGCAACAGCAGTCTCGCCTC
AAAGAGTTGATCAGCCGTGGTCGTGAGCAGGGTTACCTGACTTACGCGGAGGTCAACG
ACCACCTGCCGGAGGATATTTCAGATCCGGAACAGGTGGAAGACATCATCCGCATGAT
CAACGACATGGGGATCAACGTATTCGAGAGTGCTCCGGATGCGGATGCCCTTTTGTTG

GCCGAAGCCGATACCGACGAAGCAGCAGCTGAAGAAGCCGCCGCAGCGTTGGCGGCT
GTGGAAACCGACATTGGTCGCACTACCGACCCCGTGCGTATGTACATGCGCGAAATGG
GTACGGTAGAGCTGCTCACACGTGAAGGCGAAATCGAAATCGCCAAGCGTATCGAAG
AGGGCATCCGTGAAGTGATGGGCGCGATCGCGCACTTCCCTGGCACGGTTGAGCACAT
CCTCTCCGAATACACTCGCGTCACCACCGAAGGTGGCCGCCTGTCCGACGTCCTGAGC
GGTTACATCGACCCGGACGACGGTATTGCGCCGCCTGCCGCCGAAGTACCACCGCCTG
TCGATGCCAAGGCCGCAAAAGCGGACGACGACACCGACGACGATGACGCCGAAGCCA
GTGACGACGAAGAAGAAGCCGAAAGCGGTCCGGATCCGGTCATCGCAGCCCAGCGCT
TTGGCGCCGTTGCCGACCAGATGGAAATTACCCGCAAGGCGCTGAAAAAGCACGGTCG
CGAACACAAGCAAGCCCTGGCTGAAATGCTGGCCCTGGCTGAGCTGTTCATGCCGATC
AAACTGGTTCCGAAGCAATTCGAAGGCCTGGTTGAACGTGTTCGTAGCGCCCTGGATC
GCCTGCGTCAGCAAGAGCGCGCGATCATGCAGCTCTGTGTTCGTGATGCCCGCATGCC
ACGCGCCGACTTCCTGCGCCAGTTCCCTGGCAATGAAGTGGACGAAAGCTGGTCCGAC
GCATTGGCCAAAGGCAAGGCCAAGTACGCCGAAGCCATCGGCCGCCTGCAGCCGGAT
ATCATCCGTTGCCAGCAGAAGCTGACAGCGCTCGAGACCGAGACTGGCCTGACGATCG
CCGAGATCAAGGACATCAACCGTCGCATGTCGATCGGCGAGGCCAAGGCCCGTCGCGC
GAAGAAAGAGATGGTCGAAGCCAACTTGCGTCTGGTGATCTCCATCGCCAAGAAGTAC
ACCAACCGTGGCCTGCAATTCCTCGACCTGATCCAGGAAGGCAACATCGGTTTGATGA
AAGCGGTAGACAAGTTCGAATACCGTCGCGGCTACAAATTCTCGACTTATGCCACCTG
GTGGATCCGTCAGGCGATCACTCGTTCGATCGCCGACCAGGCCCGCACCATCCGTATTC
CGGTGCACATGATCGAGACGATCAACAAGCTCAACCGTATTTCCCGGCAGATGTTGCA
GGAAATGGGTCGCGAACCGACTCCGGAAGAGCTGGGCGAACGCATGGAAATGCCTGA
GGACAAGATCCGCAAGGTATTGAAGATCGCTAAAGAGCCGATCTCCATGGAAACCCCG
ATCGGTGATGACGAAGACTCCCATCTGGGTGACTTCATCGAAGACTCGACCATGCAGT
CGCCAATCGATGTCGCCACCGTTGAGAGCCTTAAAGAAGCGACTCGCGAAGTACTCTC
CGGCCTCACTGCCCGTGAAGCCAAGGTACTGCGCATGCGCTTCGGCATCGACATGAAT
ACCGACCACACCCTCGAGGAAGTCGGTAAGCAGTTCGACGTTACCCGTGAGCGGATTC
GTCAGATCGAAGCCAAGGCGCTGCGCAAGCTGCGCCACCCGACGCGAAGCGAGCATCT
GCGCTCCTTCCTCGACGAGTAATTGACTAAGCCAGCCATACTCGCCCTTGCTGATGGCA
GCATTTTTCGCGGCGAAGCCATTGGAGCCGACGGTCAAACCGTTGGTGAGGTGGTGTT
TAACACCGCAATGACCGGCTATCAGGAAATCCTTACCGATCCTTCCTACGCCCAACAG
ATCGTTACCCTGACTTACCCGCACATCGGCAATACCGGCACCACGCCGGAAGACGCCG
AGTCCGATCGTGTCTGGTCGGCCGGTCTGGTGATTCGCGACCTGCCACTGGTTGCGAGC
AACTGGCGTAACACCTTGTCCCTGTCCGACTACCTGAAAGCCAACAATGTTGTGGCGAT
CGCCGGTATCGACACCCGTCGTCTGACGCGCATCCTGCGCGAGAAAGGCGCGCAGAAC
GGCTGCATCATGGCCGGCGACAATATCTCCGACGAAGCGGCGATTGCCGCTGCGCGCG
GCTTCCCGGGCCTGAAAGGCATGGATCTGGCGAAGGTCGTCAGCACCAAGGAAAGCTA
CGAGTGGCGCTCCAGCGTCTGGAGCCTGAAGACCGACAGTCACCCGACCATCGAGGCT
TCCGAGCTGCCTTACCACGTGGTTGCCTACGACTACGGCGTCAAGCTGAACATCCTGCG
CATGCTGGTCGAGCGCGGTTGCCGCGTGACCGTGGTACCTGCGCAAACCCCGGCCAGC
GACGTCCTGGCGCTCAAGCCTGACGGTGTGTTCCTGTCCAACGGTCCTGGCGACCCCGA
GCCTTGCGATTACGCGATCCAGGCGATCAAGGACGTGCTGGAGACCGAGATACCGGTC
TTCGGGATCTGCCTGGGCCACCAACTGCTGGCGCTGGCCGCCGGCGCCAAGACAGTGA
AGATGGGCCACGGCCACCACGGTGCCAACCACCCGGTCCAGGACCTGGACAGCGGTGT
AGTGATGATCACCAGTCAGAACCACGGTTTTGCGGTGGACGAAACCACCCTGCCGGGC
AACGTGCGGGCGATCCACAAGTCGTTGTTCGACGGCACCCTGCAAGGCATCGAGCTGA
CCGACAAGAGCGCATTCAGCTTCCAGGGCCACCCTGAAGCGAGCCCGGGCCCGAACGA
TGTGGCGCCGCTGTTCGATCGTTTCATCAACGAGATGGCCAAGCGACGCTGAATGAGT
AGCGGACGTATCGTTCAAATCATCGGCGCCGTTATCGACGTGGAATTTCCACGCGACA
GCGTACCGAGCATCTACGACGCCTTGAAGGTTCAAGGCGCCGAAACCACTCTGGAAGT
TCAGCAGCAGCTGGGCGACGGCGTGGTACGTACCATTGCGATGGGCTCCACCGAGGGC

TTGAAGCGCGGTCTGGACGTCAACAACACTGGCGCAGCCATCTCCGTACCGGTCGGTA
AAGCGACCCTGGGCCGGATCATGGACGTACTGGGCAACCCGATCGACGAAGCTGGCCC
GATCGGCGAAGAAGAGCGTTGGGGCATTCACCGTCCTGCGCCGACCTTCGCTGAACAA
GCTGGTGGCAACGACCTGCTGGAAACCGGCATCAAGGTTATCGACCTGGTTTGCCCGT
TCGCCAAGGGCGGTAAAGTTGGTCTGTTCGGTGGTGCCGGTGTGGGCAAAACCGTAAA
CATGATGGAACTGATCCGTAACATCGCCATCGAGCACAGCGGTTATTCCGTGTTCGCCG
GTGTGGGCGAGCGTACTCGTGAGGGTAACGACTTCTACCACGAGATGAAGGATTCCAA
CGTTCTGGACAAAGTGGCACTGGTATACGGCCAGATGAACGAGCCGCCGGGAAACCGT
CTGCGCGTAGCTCTGACCGGCCTGACCATGGCCGAGAAGTTCCGTGACGAAGGTAACG
ACGTTCTGCTGTTCGTCGACAACATCTATCGTTACACCCTGGCCGGTACCGAAGTATCC
GCACTGCTGGGCCGTATGCCTTCGGCAGTAGGTTACCAGCCGACCCTGGCTGAAGAGA
TGGGCGTTCTGCAAGAACGTATCACTTCGACCAAGCAAGGCTCGATCACCTCGATCCA
AGCGGTATACGTACCTGCGGACGACTTGACCGACCCGTCGCCAGCGACCACCTTCGCC
CACTTGGACGCCACCGTCGTTCTTTCCCGTGACATCGCTTCCCTGGGTATCTACCCAGC
GGTAGACCCACTGGACTCGACTTCCCGTCAGCTGGACCCGAACGTGATCGGCAACGAG
CACTATGAAACCGCTCGCGGCGTTCAGTACGTGCTGCAGCGCTACAAAGAGCTGAAGG
ACATCATTGCGATCCTGGGTATGGACGAACTGTCCGAAGCCGACAAGCAACTGGTATC
CCGCGCTCGTAAGATCCAGCGCTTCCTGTCGCAGCCGTTCTTCGTGGCTGAAGTCTTCA
CTGGTTCTCCAGGCAAATACGTTTCCCTGAAAGACACCATCGCTGGCTTCAAAGGCATC
CTCAACGGTGACTACGACCATCTGCCAGAACAAGCGTTCTACATGGTTGGTGGCATCG
AAGAAGCGATCGAGAA

NCBI Reference Sequence: NZ_CP027746.1
Strain: DSM19603
Chained genes: 16S rRNA, recA, gyrB, rpoD, carA, atpD >NZ_CP027746_DSM19603
GAACTGAAGAGTTTGATCATGGCTCAGATTGAACGCTGGCGGCAGGCCTAACACATGC
AAGTCGAGCGGTAGAGAGAAGCTTGCTTCTCTTGAGAGCGGCGGACGGGTGAGTAATG
CCTAGGAATCTGCCTGGTAGTGGGGGATAACGTCCGGAAACGGACGCTAATACCGCAT
ACGTCCTACGGGAGAAAGCAGGGGACCTTCGGGCCTTGCGCTATCAGATGAGCCTAGG
TCGGATTAGCTAGTTGGTGAGGTAATGGCTCACCAAGGCGACGATCCGTAACTGGTCT
GAGAGGATGATCAGTCACACTGGAACTGAGACACGGTCCAGACTCCTACGGGAGGCA
GCAGTGGGGAATATTGGACAATGGGCGAAAGCCTGATCCAGCCATGCCGCGTGTGTGA
AGAAGGTCTTCGGATTGTAAAGCACTTTAAGTTGGGAGGAAGGGTACTTACCTAATAC
GTGAGTATTTTGACGTTACCGACAGAATAAGCACCGGCTAACTCTGTGCCAGCAGCCG
CGGTAATACAGAGGGTGCAAGCGTTAATCGGAATTACTGGGCGTAAAGCGCGCGTAGG
TGGTTCGTTAAGTTGGATGTGAAATCCCCGGGCTCAACCTGGGAACTGCATCCAAAAC
TGGCGAGCTAGAGTATGGTAGAGGGTGGTGGAATTTCCTGTGTAGCGGTGAAATGCGT
AGATATAGGAAGGAACACCAGTGGCGAAGGCGACCACCTGGACTGATACTGACACTG
AGGTGCGAAAGCGTGGGGAGCAAACAGGATTAGATACCCTGGTAGTCCACGCCGTAA
ACGATGTCAACTAGCCGTTGGGAGCCTTGAGCTCTTAGTGGCGCAGCTAACGCATTAA
GTTGACCGCCTGGGGAGTACGGCCGCAAGGTTAAAACTCAAATGAATTGACGGGGGCC
CGCACAAGCGGTGGAGCATGTGGTTTAATTCGAAGCAACGCGAAGAACCTTACCAGGC
CTTGACATCCAATGAACTTTCCAGAGATGGATTGGTGCCTTCGGGAACATTGAGACAG
GTGCTGCATGGCTGTCGTCAGCTCGTGTCGTGAGATGTTGGGTTAAGTCCCGTAACGAG
CGCAACCCTTGTCCTTAGTTACCAGCACGTTATGGTGGGCACTCTAAGGAGACTGCCGG
TGACAAACCGGAGGAAGGTGGGGATGACGTCAAGTCATCATGGCCCTTACGGCCTGGG
CTACACACGTGCTACAATGGTCGGTACAGAGGGTTGCCAAGCCGCGAGGTGGAGCTAA
TCCCATAAAACCGATCGTAGTCCGGATCGCAGTCTGCAACTCGACTGCGTGAAGTCGG
AATCGCTAGTAATCGCGAATCAGAATGTCGCGGTGAATACGTTCCCGGGCCTTGTACA
CACCGCCCGTCACACCATGGGAGTGGGTTGCACCAGAAGTAGCTAGTCTAACCTTCGG
GAGGACGGTTACCACGGTGTGATTCATGACTGGGGTGAAGTCGTAACAAGGTAGCCGT

AGGGGAACCTGCGGCTGGATCACCTCCTTAAATGGACGACAACAAGAAGAAAGCCTTG
GCTGCGGCCCTGGGTCAGATCGAACGTCAATTCGGCAAGGGTGCCGTAATGCGTATGG
GCGATCACGACCGCCAGGCGATCCCGGCCATTTCCACTGGCTCTCTGGGCCTGGACATC
GCGCTCGGCATCGGCGGCCTGCCAAAAGGCCGTATTGTTGAAATCTACGGTCCGGAAT
CGTCCGGTAAAACCACCCTGACCCTGTCGGTGATTGCCCAGGCACAGAAGATGGGCGC
CACCTGCGCCTTCGTCGACGCCGAGCACGCACTGGACCCGGAATACGCCGGCAAACTG
GGGGTCAACGTTGACGACCTGCTGGTTTCCCAGCCGGACACCGGCGAACAGGCGCTGG
AAATCACCGACATGCTGGTGCGTTCCAATGCCATCGACGTGATCGTGATCGACTCCGTG
GCGGCACTGGTACCCAAGGCCGAGATCGAAGGCGAGATGGGCGACATGCACGTGGGC
CTGCAGGCCCGCCTGATGTCCCAGGCGCTGCGCAAGATCACCGGTAACATCAAGAACG
CCAACTGCCTGGTGATCTTCATCAACCAGATCCGTATGAAAATCGGCGTGATGTTCGGC
AGCCCGGAAACCACCACCGGCGGTAACGCGCTGAAGTTCTACGCCTCGGTTCGTCTGG
ACATCCGTCGTACTGGCGCGGTGAAGGAAGGCGACGAAGTCGTCGGTAGCGAAACCC
GGGTCAAGATCGTCAAGAACAAGGTGGCTCCACCGTTCCGTCAGGCTGAATTCCAGAT
CCTGTACGGCAAGGGTATCTACCTGAACGGCGAGATCATCGATCTGGGCGTGCTGCAC
GGTTTCCTCGAGAAGTCCGGTGCCTGGTACAGCTACCAGGGCAACAAGATCGGTCAGG
GCAAGGCCAACTCGGCCAAGTTCCTGCAGGACAATCCGGAAATCGGCAATGCCCTCGA
GAAGCAGATTCGCGACAAGCTGCTGGCTCCAACCGCTGATGTCAAAGCTTCGCCGGTC
AACGAGACCATCGATGACATGGCTGACGCGGATATCTGAATGAGCGAAGAAAACACG
TACGACTCGAGCAGCATTAAAGTGCTGAAAGGTTTGGATGCCGTACGCAAACGTCCCG
GTATGTACATTGGTGACACCGACGATGGCAGCGGTCTGCACCATATGGTGTTCGAGGT
GGTCGATAACTCGATCGACGAAGCTCTGGCCGGCCACTGCGACGACATCAGCATCATC
ATCCACCCGGACGAATCCATTACCGTGCGTGACAACGGTCGCGGCATCCCGGTAGACG
TGCATAAAGAAGAAGGCGTTTCCGCGGCCGAGGTCATCATGACTGTGCTGCACGCCGG
CGGTAAGTTCGACGACAACTCCTACAAAGTATCCGGCGGTCTGCACGGTGTGGGTGTG
TCGGTAGTGAACGCCCTGTCCGAAGAACTGGTCCTGACCGTTCGCCGCAGTGGCAAGA
TCTGGGAACAGACCTACGTTCACGGTGTGCCTCAGGCGCCTATGGCGATCGTCGGTGA
CAGTGAAACCACCGGTACCCAGATTCACTTCAAGGCTTCCAGCGAGACCTTCAAGAAC
ATCCATTTCAGCTGGGACATCCTGGCCAAGCGGATTCGTGAACTGTCCTTCCTCAACTC
CGGTGTCGGTATCGTTCTGAAGGACGAGCGTAGCGGCAAGGAAGAACTGTTCAAGTAC
GAAGGCGGTCTGCGTGCGTTCGTTGAATACCTGAACACCAACAAGACCGCGGTCAACC
AGGTGTTCCATTTCAATGTGCAGCGTGAAGATGGCATCGGCGTGGAAATCGCCCTGCA
GTGGAATGACAGCTTCAACGAAAACCTGCAGTGCTTCACCAACAACATTCCGCAGCGC
GATGGCGGCACCCACCTGGTGGGCTTCCGTTCGGCACTGACACGTAACCTGAACAACT
ACATCGAACAGGAAGGTCTGGCGAAGAAGCACAAGGTCGCCACCACCGGTGACGATG
CCCGCGAAGGCCTGACCGCAATCATTTCGGTCAAGGTGCCGGATCCGAAGTTCAGCTC
CCAGACCAAAGACAAGCTGGTGTCTTCCGAAGTGAAGACCGCGGTCGAACAGGAAAT
GGGCAAGTACTTCTCCGACTTCCTGCTGGAAAACCCGAACGAAGCCAAGCTGGTGGTC
GGCAAGATGCTCGACGCCGCCCGTGCCCGTGAAGCGGCGCGTAAGGCTCGTGAGATGA
CCCGCCGTAAAGGTGCGCTGGATATCGCCGGCCTGCCGGGCAAACTGGCGGACTGCCA
GGAAAAAGACCCTGCCCTTTCCGAACTCTACCTGGTGGAAGGTGACTCTGCTGGCGGC
TCCGCCAAGCAGGGACGCAACCGTAAGACCCAGGCGATTCTGCCGCTCAAGGGCAAG
ATCCTTAACGTCGAGAAAGCGCGTTTCGACAAGATGATTTCCTCGCAAGAGGTCGGCA
CCTTGATCACTGCACTCGGTTGCGGCATCGGCCGCGAAGAGTACAACATCGACAAGCT
GCGTTATCACAACATCATCATCATGACTGACGCCGACGTCGATGGTTCGCACATCCGTA
CCCTGCTGCTGACCTTCTTCTTCCGTCAGCTGCCGGAGCTGATCGAGCGTGGCTACATC
TACATCGCTCAACCGCCGCTGTACAAGGTCAAGAAAGGCAAGCAAGAGCAATACATCA
AAGACGACGACGCCATGGAAGAGTACATGACGCAGTCGGCCCTGGAAGATGCGAGCC
TGCACCTGAACGAAGACGCACCGGGTATTTCCGGCGAGGCGCTGGAGCGCCTGGTGAA
CGACTTCCGCATGGTCATGAAAACCCTCAAGCGTCTGTCGCGCCTGTACCCTCAGGAGC
TGACCGAGCACTTCATCTACCTGCCGGCCGTGAGCCTGGAGCAGCTCTCCGATCACGC

GGCGATGCAGGATTGGCTGGCCCAATATGAAGTCCGCCTGCGCACCGTCGAGAAGTCC
GGCCTGGTCTACAAGGCCAGCCTGCGTGAAGACCGTGAACGTAATGTCTGGCTGCCAG
AGGTCGAACTGATCTCCCACGGCCTGTCGAACTACGTCACCTTCAACCGCGACTTCTTC
GGCAGCAATGACTACAAGACCGTCGTCACCCTCGGTGCTCAACTGAGCTCCCTGCTGG
ACGAAGGCGCTTATATTCAGCGTGGCGAACGCAAGAAGGCAGTGACCGAGTTCAAGG
AAGCCCTGGACTGGCTGATGACCGAAAGTACCAAGCGCCACACCATCCAGCGATACAA
AGGTCTGGGCGAGATGAACCCGGATCAGCTGTGGGAAACCACCATGGACCCAAGCGT
GCGCCGTATGCTCAAGGTCACCATCGAAGACGCCATCGGCGCCGACCAGATCTTCAAC
ACCCTGATGGGTGATGCGGTCGAGCCTCGTCGCGACTTCATCGAAAGCAACGCCCTGG
CGGTATCCAACTTGGACTTCTGAATGTCCGGAAAAGCGCAACAGCAGTCTCGCCTCAA
AGAGTTGATCAGCCGTGGTCGTGAGCAGGGTTACCTGACTTACGCGGAGGTCAACGAC
CACCTGCCGGAGGATATTTCAGATCCGGAACAGGTGGAAGACATCATCCGCATGATCA
ACGACATGGGGATCAACGTATTCGAGAGTGCTCCGGATGCGGATGCCCTTTTGTTGGC
CGAAGCCGATACCGACGAAGCAGCAGCTGAAGAAGCCGCCGCAGCGTTGGCGGCTGT
GGAAACCGACATTGGTCGCACTACCGACCCCGTGCGTATGTACATGCGCGAAATGGGT
ACGGTAGAGCTGCTCACACGTGAAGGCGAAATCGAAATCGCCAAGCGTATCGAAGAG
GGCATCCGTGAAGTGATGGGCGCGATCGCGCACTTCCCTGGCACGGTTGAGCACATCC
TCTCCGAATACACTCGCGTCACCACCGAAGGTGGCCGCCTGTCCGACGTCCTGAGCGG
TTACATCGACCCGGACGACGGTATTGCGCCGCCTGCCGCCGAAGTACCACCGCCTGTC
GATGCCAAGGCCGCAAAAGCGGACGACGACACCGACGACGATGACGCCGAAGCCAGT
GACGACGAAGAAGAAGCCGAAAGCGGTCCGGATCCGGTCATCGCAGCCCAGCGCTTT
GGCGCCGTTGCCGACCAGATGGAAATTACCCGCAAGGCGCTGAAAAAGCACGGTCGC
GAACACAAGCAAGCCCTGGCTGAAATGCTGGCCCTGGCTGAGCTGTTCATGCCGATCA
AACTGGTTCCGAAGCAATTCGAAGGCCTGGTTGAACGTGTTCGTAGCGCCCTGGATCG
CCTGCGTCAGCAAGAGCGCGCGATCATGCAGCTCTGTGTTCGTGATGCCCGCATGCCA
CGCGCCGACTTCCTGCGCCAGTTCCCTGGCAATGAAGTGGACGAAAGCTGGTCCGACG
CATTGGCCAAAGGCAAGGCCAAGTACGCCGAAGCCATCGGCCGCCTGCAGCCGGATAT
CATCCGTTGCCAGCAGAAGCTGACAGCGCTCGAGACCGAGACTGGCCTGACGATCGCC
GAGATCAAGGACATCAACCGTCGCATGTCGATCGGCGAGGCCAAGGCCCGTCGCGCGA
AGAAAGAGATGGTCGAAGCCAACTTGCGTCTGGTGATCTCCATCGCCAAGAAGTACAC
CAACCGTGGCCTGCAATTCCTCGACCTGATCCAGGAAGGCAACATCGGTTTGATGAAA
GCGGTAGACAAGTTCGAATACCGTCGCGGCTACAAATTCTCGACTTATGCCACCTGGT
GGATCCGTCAGGCGATCACTCGTTCGATCGCCGACCAGGCCCGCACCATCCGTATTCCG
GTGCACATGATCGAGACGATCAACAAGCTCAACCGTATTTCCCGGCAGATGTTGCAGG
AAATGGGTCGCGAACCGACTCCGGAAGAGCTGGGCGAACGCATGGAAATGCCTGAGG
ACAAGATCCGCAAGGTATTGAAGATCGCTAAAGAGCCGATCTCCATGGAAACCCCGAT
CGGTGATGACGAAGACTCCCATCTGGGTGACTTCATCGAAGACTCGACCATGCAGTCG
CCAATCGATGTCGCCACCGTTGAGAGCCTTAAAGAAGCGACTCGCGAAGTACTCTCCG
GCCTCACTGCCCGTGAAGCCAAGGTACTGCGCATGCGCTTCGGCATCGACATGAATAC
CGACCACACCCTCGAGGAAGTCGGTAAGCAGTTCGACGTTACCCGTGAGCGGATTCGT
CAGATCGAAGCCAAGGCGCTGCGCAAGCTGCGCCACCCGACGCGAAGCGAGCATCTG
CGCTCCTTCCTCGACGAGTAATTGACTAAGCCAGCCATACTCGCCCTTGCTGATGGCAG
CATTTTTCGCGGCGAAGCCATTGGAGCCGACGGTCAAACCGTTGGTGAGGTGGTGTTT
AACACCGCAATGACCGGCTATCAGGAAATCCTTACCGATCCTTCCTACGCCCAACAGA
TCGTTACCCTGACTTACCCGCACATCGGCAATACCGGCACCACGCCGGAAGACGCCGA
GTCCGATCGTGTCTGGTCGGCCGGTCTGGTGATTCGCGACCTGCCACTGGTTGCGAGCA
ACTGGCGTAACACCTTGTCCCTGTCCGACTACCTGAAAGCCAACAATGTTGTGGCGATC
GCCGGTATCGACACCCGTCGTCTGACGCGCATCCTGCGCGAGAAAGGCGCGCAGAACG
GCTGCATCATGGCCGGCGACAATATCTCCGACGAAGCGGCGATTGCCGCTGCGCGCGG
CTTCCCGGGCCTGAAAGGCATGGATCTGGCGAAGGTCGTCAGCACCAAGGAAAGCTAC
GAGTGGCGCTCCAGCGTCTGGAGCCTGAAGACCGACAGTCACCCGACCATCGAGGCTT

CCGAGCTGCCTTACCACGTGGTTGCCTACGACTACGGCGTCAAGCTGAACATCCTGCGC
ATGCTGGTCGAGCGCGGTTGCCGCGTGACCGTGGTACCTGCGCAAACCCCGGCCAGCG
ACGTCCTGGCGCTCAAGCCTGACGGTGTGTTCCTGTCCAACGGTCCTGGCGACCCCGAG
CCTTGCGATTACGCGATCCAGGCGATCAAGGACGTGCTGGAGACCGAGATACCGGTCT
TCGGGATCTGCCTGGGCCACCAACTGCTGGCGCTGGCCGCCGGCGCCAAGACAGTGAA
GATGGGCCACGGCCACCACGGTGCCAACCACCCGGTCCAGGACCTGGACAGCGGTGTA
GTGATGATCACCAGTCAGAACCACGGTTTTGCGGTGGACGAAACCACCCTGCCGGGCA
ACGTGCGGGCGATCCACAAGTCGTTGTTCGACGGCACTCTGCAAGGCATCGAGCTGAC
CGACAAGAGCGCATTCAGCTTCCAGGGCCACCCTGAAGCGAGCCCGGGCCCGAACGAT
GTGGCGCCGCTGTTCGATCGTTTCATCAACGAGATGGCCAAGCGACGCTGAATGAGTA
GCGGACGTATCGTTCAAATCATCGGCGCCGTTATCGACGTGGAATTTCCACGCGACAG
CGTACCGAGCATCTACGACGCCTTGAAGGTTCAAGGCGCCGAAACCACTCTGGAAGTT
CAGCAGCAGCTGGGCGACGGCGTGGTACGTACCATTGCGATGGGCTCCACCGAGGGCT
TGAAGCGCGGTCTGGACGTCAACAACACTGGCGCAGCCATCTCCGTACCGGTCGGTAA
AGCGACCCTGGGCCGGATCATGGACGTACTGGGCAACCCGATCGACGAAGCTGGCCCG
ATCGGCGAAGAAGAGCGTTGGGGCATTCACCGTCCTGCGCCGACCTTCGCTGAACAAG
CTGGTGGCAACGACCTGCTGGAAACCGGCATCAAGGTTATCGACCTGGTTTGCCCGTTC
GCCAAGGGCGGTAAAGTTGGTCTGTTCGGTGGTGCCGGTGTGGGCAAAACCGTAAACA
TGATGGAACTGATCCGTAACATCGCCATCGAGCACAGCGGTTATTCCGTGTTCGCCGGT
GTGGGCGAGCGTACTCGTGAGGGTAACGACTTCTACCACGAGATGAAGGATTCCAACG
TTCTGGACAAAGTGGCACTGGTATACGGCCAGATGAACGAGCCGCCGGGAAACCGTCT
GCGCGTAGCTCTGACCGGCCTGACCATGGCCGAGAAGTTCCGTGACGAAGGTAACGAC
GTTCTGCTGTTCGTCGACAACATCTATCGTTACACCCTGGCCGGTACCGAAGTATCCGC
ACTGCTGGGCCGTATGCCTTCGGCAGTAGGTTACCAGCCGACCCTGGCTGAAGAGATG
GGCGTTCTGCAAGAACGTATCACTTCGACCAAGCAAGGCTCGATCACCTCGATCCAAG
CGGTATACGTACCTGCGGACGACTTGACCGACCCGTCGCCAGCGACCACCTTCGCCCA
CTTGGACGCCACCGTCGTTCTTTCCCGTGACATCGCTTCCCTGGGTATCTACCCAGCGG
TAGACCCACTGGACTCGACTTCCCGTCAGCTGGACCCGAACGTGATCGGCAACGAGCA
CTATGAAACCGCTCGCGGCGTTCAGTACGTGCTGCAGCGCTACAAAGAGCTGAAGGAC
ATCATTGCGATCCTGGGTATGGACGAACTGTCCGAAGCCGACAAGCAACTGGTATCCC
GCGCTCGTAAGATCCAGCGCTTCCTGTCGCAGCCGTTCTTCGTGGCTGAAGTCTTCACT
GGTTCTCCAGGCAAATACGTTTCCCTGAAAGACACCATCGCTGGCTTCAAAGGCATCCT
CAACGGTGACTACGACCATCTGCCAGAACAAGCGTTCTACATGGTTGGTGGCATCGAA
GAAGCGATCGAGAAAGCCAAGAAACTGTAA

NCBI Reference Sequence: NZ_CP027709.1
Strain: ATCC17809
Chained genes: 16S rRNA, recA, gyrB, rpoD, carA, atpD >NZ_CP027709_ATCC17809
GAACTGAAGAGTTTGATCATGGCTCAGATTGAACGCTGGCGGCAGGCCTAACACATGC
AAGTCGAGCGGTAGAGAGGTGCTTGCACCTCTTGAGAGCGGCGGACGGGTGAGTAATG
CCTAGGAATCTGCCTGGTAGTGGGGGATAACGTTCGGAAACGGACGCTAATACCGCAT
ACGTCCTACGGGAGAAAGCAGGGGACCTTCGGGCCTTGCGCTATCAGATGAGCCTAGG
TCGGATTAGCTAGTTGGTGAGGTAATGGCTCACCAAGGCGACGATCCGTAACTGGTCT
GAGAGGATGATCAGTCACACTGGAACTGAGACACGGTCCAGACTCCTACGGGAGGCA
GCAGTGGGGAATATTGGACAATGGGCGAAAGCCTGATCCAGCCATGCCGCGTGTGTGA
AGAAGGTCTTCGGATTGTAAAGCACTTTAAGTTGGGAGGAAGGGTACTTACCTAATAC
GTGAGTATTTTGACGTTACCGACAGAATAAGCACCGGCTAACTCTGTGCCAGCAGCCG
CGGTAATACAGAGGGTGCAAGCGTTAATCGGAATTACTGGGCGTAAAGCGCGCGTAGG
TGGTTCGTTAAGTTGAATGTGAAATCCCCGGGCTCAACCTGGGAACTGCATCCAAAAC
TGGCGAGCTAGAGTATGGTAGAGGGTGGTGGAATTTCCTGTGTAGCGGTGAAATGCGT
AGATATAGGAAGGAACACCAGTGGCGAAGGCGACCACCTGGACTGATACTGACACTG

AGGTGCGAAAGCGTGGGGAGCAAACAGGATTAGATACCCTGGTAGTCCACGCCGTAA
ACGATGTCAACTAGCCGTTGGGAGCCTTGAGCTCTTAGTGGCGCAGCTAACGCATTAA
GTTGACCGCCTGGGGAGTACGGCCGCAAGGTTAAAACTCAAATGAATTGACGGGGGCC
CGCACAAGCGGTGGAGCATGTGGTTTAATTCGAAGCAACGCGAAGAACCTTACCAGGC
CTTGACATCCAATGAACTTTCCAGAGATGGATTGGTGCCTTCGGGAACATTGAGACAG
GTGCTGCATGGCTGTCGTCAGCTCGTGTCGTGAGATGTTGGGTTAAGTCCCGTAACGAG
CGCAACCCTTGTCCTTAGTTACCAGCACGTAATGGTGGGCACTCTAAGGAGACTGCCG
GTGACAAACCGGAGGAAGGTGGGGATGACGTCAAGTCATCATGGCCCTTACGGCCTGG
GCTACACACGTGCTACAATGGTCGGTACAGAGGGTTGCCAAGCCGCGAGGTGGAGCTA
ATCCCATAAAACCGATCGTAGTCCGGATCGCAGTCTGCAACTCGACTGCGTGAAGTCG
GAATCGCTAGTAATCGCGAATCAGAATGTCGCGGTGAATACGTTCCCGGGCCTTGTAC
ACACCGCCCGTCACACCATGGGAGTGGGTTGCACCAGAAGTAGCTAGTCTAACCTTCG
GGAGGACGGTTACCACGGTGTGATTCATGACTGGGGTGAAGTCGTAACAAGGTAGCCG
TAGGGGAACCTGCGGCTGGATCACCTCCTTAAATGGACGACAACAAGAAGAAAGCCTT
GGCTGCGGCCCTGGGTCAGATCGAACGTCAATTCGGCAAGGGTGCCGTAATGCGTATG
GGCGATCACGATCGCCAGGCGATCCCGGCCATTTCCACTGGCTCTCTGGGTCTGGACAT
CGCACTCGGCATCGGCGGCCTGCCAAAAGGCCGTATTGTTGAAATCTACGGTCCGGAA
TCGTCCGGTAAAACCACCCTGACCCTGTCGGTGATCGCCCAGGCACAGAAGATGGGCG
CCACCTGCGCCTTCGTCGACGCCGAGCACGCACTGGACCCGGAATACGCCGGCAAACT
GGGGGTCAACGTTGACGACCTGCTGGTTTCCCAGCCGGACACCGGCGAACAGGCGCTG
GAAATCACCGACATGCTGGTGCGCTCCAATGCCATCGACGTGATCGTGATCGACTCCG
TGGCGGCGCTGGTACCCAAGGCCGAGATCGAAGGCGAGATGGGCGACATGCACGTGG
GCCTGCAGGCTCGCCTGATGTCCCAGGCGCTGCGCAAGATCACCGGTAACATCAAGAA
CGCCAACTGCCTGGTGATCTTCATCAACCAGATCCGTATGAAAATCGGCGTGATGTTCG
GCAGCCCGGAAACCACCACCGGTGGTAACGCGCTGAAGTTCTACGCTTCGGTTCGTCT
GGACATCCGTCGTACTGGCGCGGTGAAGGAAGGTGACGAAGTCGTCGGTAGCGAAAC
CCGGGTCAAGATCGTCAAGAACAAGGTGGCTCCACCTTTCCGTCAAGCTGAGTTCCAG
ATCCTGTACGGCAAGGGTATCTACCTGAACGGCGAGATCATCGATCTGGGCGTGCTGC
ACGGTTTCCTCGAGAAGTCCGGTGCCTGGTACAGCTACCAGGGCAACAAGATCGGTCA
GGGCAAGGCCAACTCGGCCAAGTTCCTGCAGGACAATCCGGAAATCGGTAATGCCCTC
GAGAAGCAGATTCGCGACAAGCTGCTGGCTCCGAGCGGAGATACCAAGGCTCTGCCCG
TCAACGAGACCATCGATGACATGGCCGACGCGGATATCTGAATGAGCGAAGAAAACA
CGTACGACTCGAGCAGCATTAAAGTGCTGAAAGGTTTGGATGCCGTACGCAAACGTCC
CGGTATGTACATTGGTGACACCGACGATGGCAGCGGTCTGCACCATATGGTGTTCGAG
GTGGTCGACAACTCGATCGACGAAGCTCTGGCCGGCCACTGCGACGACATCAGCATCA
TCATCCACCCGGACGAATCCATTACCGTGCGTGACAACGGTCGCGGCATCCCGGTAGA
CGTGCATAAAGAAGAAGGCGTTTCCGCAGCCGAGGTCATCATGACCGTGCTGCACGCC
GGCGGTAAGTTCGACGACAACTCCTACAAGGTATCCGGCGGTCTGCACGGTGTGGGTG
TGTCGGTAGTGAACGCCCTGTCCGAAGAACTGGTGCTGACCGTTCGCCGCAGTGGCAA
GATCTGGGAACAGACCTACGTTCACGGTGTGCCTCAGGCGCCTATGGCAATTGTCGGT
GACAGCGAGACCACCGGTACCCAGATTCACTTCAAGGCTTCCAGCGAGACCTTCAAGA
ACATCCATTTCAGCTGGGACATCCTGGCCAAGCGGATTCGTGAACTGTCCTTCCTCAAC
TCCGGTGTCGGTATCGTTCTGAAGGACGAGCGCAGCGGCAAGGAAGAGCTGTTCAAGT
ACGAAGGCGGCCTGCGCGCATTCGTTGAATACCTGAACACCAACAAGACTGCGGTCAA
CCAGGTGTTCCACTTCAACGTGCAGCGTGAAGACGGCATCGGCGTGGAAATCGCCCTG
CAGTGGAACGACAGCTTCAACGAAAACCTGCAGTGCTTCACCAACAACATTCCGCAGC
GCGACGGCGGCACCCACCTGGTGGGCTTCCGCTCGGCACTGACGCGTAACCTGAACAA
CTACATCGAGCAGGAAGGTCTGGCGAAGAAGCACAAGGTGGCCACCACCGGTGACGA
TGCCCGCGAAGGCCTGACCGCGATCATTTCGGTCAAGGTGCCGGATCCGAAGTTCAGC
TCCCAGACCAAAGATAAGCTGGTGTCTTCCGAAGTGAAGACCGCAGTCGAACAGGAAA
TGGGCAAGTACTTCTCCGACTTCCTGCTGGAAAACCCGAACGAAGCCAAGCTGGTGGT

CGGCAAGATGCTCGACGCCGCCCGTGCCCGTGAAGCGGCGCGTAAGGCTCGTGAGATG
ACCCGCCGTAAAGGTGCGCTGGATATCGCCGGCCTGCCGGGCAAACTGGCGGACTGCC
AGGAAAAGGACCCTGCCCTTTCCGAACTCTACCTGGTGGAAGGTGACTCTGCTGGCGG
CTCCGCCAAGCAGGGACGCAACCGCAAGACCCAGGCGATTCTGCCGCTCAAGGGCAA
GATTCTTAACGTCGAGAAAGCGCGCTTCGACAAGATGATTTCCTCGCAAGAGGTCGGC
ACCTTGATCACTGCACTCGGCTGCGGCATCGGCCGCGAAGAGTACAACATCGACAAGC
TGCGTTATCACAACATCATCATCATGACCGACGCCGACGTCGACGGTTCGCACATCCGC
ACCCTGCTGCTGACTTTCTTCTTCCGTCAGCTGCCGGAGCTGATCGAGCGTGGCTACAT
CTACATCGCTCAACCGCCGCTGTACAAGGTCAAGAAAGGCAAGCAAGAGCAATACATC
AAAGACGACGACGCCATGGAAGAGTACATGACGCAGTCGGCCCTGGAAGATGCGAGC
CTGCACCTGAACGAAGAAGCACCGGGTATTTCCGGCGAGGCGCTGGAGCGCCTGGTGA
ACGACTTCCGCATGGTCATGAAAACCCTCAAGCGCCTGTCGCGCCTGTACCCTCAGGA
GCTGACCGAACACTTCATCTACCTGCCAGCCGTGAGCCTGGAGCAACTCTCCGATCAC
GCAGCGATGCAGGATTGGTTGGCCCAATATGAAGTCCGCCTGCGCACCGTCGAGAAGT
CCGGCCTGGTCTACAAGGCCAGCCTGCGTGAAGACCGTGAACGTAATGTCTGGCTGCC
AGAGGTCGAACTGATCTCCCACGGCCTGTCGAACTACGTCACCTTCAACCGCGACTTCT
TCGGCAGCAATGACTACAAGACCGTCGTCACCCTCGGCGCTCAACTGAGCTCCCTGCT
GGACGAAGGCGCTTATATTCAGCGTGGCGAACGCAAGAAGGCGGTGACCGAGTTCAA
GGAAGCCCTGGACTGGCTGATGACCGAAAGCACCAAGCGCCACACCATCCAGCGATAC
AAAGGTCTGGGCGAGATGAACCCGGATCAGCTGTGGGAAACCACCATGGACCCAAGC
GTGCGCCGCATGCTCAAGGTCACCATCGAAGACGCCATCGGCGCCGACCAGATCTTCA
ACACCCTGATGGGTGATGCGGTCGAGCCTCGTCGCGACTTCATCGAAAGCAACGCCCT
GGCGGTATCCAACCTGGACTTCTGAATGTCCGGAAAAGCGCAACAGCAGTCTCGCCTC
AAAGAGTTGATCAGCCGTGGTCGTGAGCAGGGTTACCTGACTTACGCGGAGGTCAACG
ACCACCTGCCGGAGGATATTTCAGATCCGGAACAGGTGGAAGACATCATCCGCATGAT
CAACGACATGGGGATCAACGTATTCGAGAGTGCTCCGGATGCGGATGCCCTTTTGTTG
GCCGAAGCCGATACCGACGAAGCAGCAGCTGAAGAAGCCGCCGCAGCGTTGGCGGCT
GTGGAAACCGACATTGGTCGCACTACCGACCCAGTGCGTATGTACATGCGCGAAATGG
GCACGGTAGAGCTGCTCACACGTGAAGGCGAAATCGAAATCGCCAAGCGTATCGAAG
AGGGCATCCGTGAAGTGATGGGCGCGATCGCGCACTTCCCTGGCACGGTTGAGCACAT
CCTCTCCGAATACACTCGCGTCACCACCGAAGGTGGCCGCCTGTCCGACGTCCTGAGC
GGTTACATCGACCCGGATGACGGCATTGCGCCGCCTGCCGCCGAAGTACCACCGCCTG
TCGATGCCAAGGCCGCGAAAGCGGACGACGACACCGACGACGATGACGCCGAAGCCA
GTGACGACGAAGAAGAAGCCGAAAGCGGTCCGGATCCGGTCATCGCAGCCCAGCGCT
TTGGCGCCGTTGCCGACCAGATGGAAATCACCCGCAAGGCGCTGAAAAAGCACGGTCG
CGAACACAAGCAAGCCCTGGCTGAAATGCTGGCCCTGGCTGAACTGTTCATGCCGATC
AAACTGGTTCCGAAGCAATTCGAAGGCCTGGTTGAACGTGTTCGTAGCGCCCTGGATC
GCCTGCGTCAGCAAGAGCGCGCGATCATGCAGCTCTGTGTTCGTGATGCCCGCATGCC
ACGCGCCGACTTCCTGCGCCAGTTCCCTGGCAATGAAGTGGACGAAAGCTGGTCCGAC
GCGCTGGCCAAAGGCAAGGCCAAGTACGCCGAAGCCATCGGCCGCCTGCAGCCGGAC
ATCATCCGTTGCCAGCAGAAGCTGACCGCGCTCGAGACCGAGACCGGCCTGACGATTG
CCGAGATCAAGGACATCAACCGTCGCATGTCGATCGGCGAGGCCAAGGCCCGTCGCGC
GAAGAAAGAGATGGTCGAAGCCAACTTGCGCCTGGTGATCTCCATCGCCAAGAAGTAC
ACCAACCGTGGCCTGCAGTTCCTCGACCTGATCCAGGAAGGCAACATCGGTTTGATGA
AAGCGGTAGACAAGTTCGAATACCGTCGCGGCTACAAGTTCTCGACTTATGCCACCTG
GTGGATCCGTCAGGCGATCACTCGCTCGATCGCCGACCAGGCCCGCACCATCCGTATTC
CGGTGCACATGATCGAGACGATCAACAAGCTCAACCGTATTTCCCGGCAGATGTTGCA
GGAAATGGGTCGCGAACCGACTCCGGAAGAGCTGGGCGAACGCATGGAAATGCCTGA
GGACAAGATCCGCAAGGTATTGAAGATCGCTAAAGAGCCGATCTCCATGGAAACCCCG
ATCGGTGATGACGAAGACTCCCATCTGGGTGACTTCATCGAAGACTCGACCATGCAGT
CGCCAATCGATGTCGCCACCGTTGAGAGCCTCAAGGAAGCGACTCGCGAAGTCCTCTC

CGGCCTCACTGCCCGTGAAGCCAAGGTACTGCGCATGCGCTTCGGCATCGACATGAAT
ACCGACCACACCCTTGAGGAAGTCGGTAAGCAGTTCGACGTTACCCGTGAGCGGATTC
GTCAGATCGAAGCCAAGGCGCTGCGCAAGCTGCGCCACCCGACGAGAAGCGAGCATC
TGCGCTCCTTCCTCGACGAGTAATTGACTAAGCCAGCCATACTCGCCCTTGCTGATGGC
AGCATTTTTCGCGGCGAAGCCATTGGAGCCGACGGTCAGACCGTTGGTGAGGTGGTGT
TTAACACCGCAATGACCGGCTATCAGGAAATCCTTACCGATCCTTCCTACGCCCAACAG
ATCGTTACCCTGACTTACCCGCACATCGGCAACACTGGCACCACGCCGGAAGACGCCG
AGTCCGATCGTGTCTGGTCGGCCGGTCTGGTGATTCGCGACCTGCCACTGGTTGCGAGC
AACTGGCGTAACACCCTGTCCCTGTCCGATTACCTGAAAGCCAACAATGTCGTGGCGA
TCGCCGGTATCGACACCCGTCGCCTGACGCGCATCCTGCGCGAGAAAGGCGCACAGAA
CGGCTGCATCATGGCCGGCGACAACATCTCCGACGAAGCGGCGATTGCTGCTGCACGC
GGCTTCCCTGGCCTGAAAGGCATGGATCTGGCGAAGGTCGTCAGCACCAAGGAAAGCT
ACGAGTGGCGCTCCAGCGTCTGGAGCCTGAAGACCGACAGTCATCCGACTATCGAGGC
TTCCGAGCTGCCTTACCACGTGGTTGCCTACGACTACGGCGTCAAGCTGAACATCCTGC
GCATGCTGGTCGAGCGCGGTTGCCGCGTGACCGTAGTGCCTGCGCAAACCCCGGCCAG
CGACGTCCTGGCACTCAAGCCTGACGGTGTGTTCCTGTCCAACGGTCCTGGCGACCCCG
AGCCTTGCGATTACGCCATCCAGGCGATCAAGGACGTGCTGGAAACCGAGATTCCGGT
CTTCGGTATCTGCCTGGGCCACCAACTGCTGGCACTGGCCTCCGGCGCCAAGACGGTG
AAAATGGGCCACGGCCACCACGGCGCCAACCACCCGGTCCAGGACCTGGACAGCGGT
GTGGTGATGATCACCAGCCAGAACCACGGTTTTGCGGTGGACGAAACCACCCTGCCGG
GCAACGTGCGGGCGATCCACAAGTCGCTGTTCGATGGCACCCTGCAAGGCATCGAGCG
TACCGACAAGAGCGCATTCAGCTTCCAGGGCCACCCTGAAGCGAGCCCGGGCCCGAAC
GATGTGGCGCCGCTGTTCGATCGTTTCATCAACGAGATGGCCAAGCGACGCTGAATGA
GTAGCGGACGTATCGTTCAAATCATCGGCGCCGTTATCGACGTGGAATTTCCACGCGA
CAGCGTACCGAGCATCTACGACGCCTTGAAGGTTCAAGGCGCCGAAACCACTCTGGAA
GTTCAGCAGCAGCTGGGCGACGGCGTGGTACGTACCATTGCGATGGGCTCCACCGAGG
GCTTGAAGCGCGGTCTGGACGTCAACAACACTGGCGCCGCCATCTCCGTACCGGTCGG
TAAAGCGACCCTGGGCCGGATCATGGACGTGCTGGGCAACCCGATCGACGAAGCTGGC
CCGATCGGCGAAGAAGAGCGTTGGGGCATTCACCGTCCTGCGCCGACCTTCGCTGAAC
AAGCTGGCGGCAACGACCTCCTGGAAACCGGCATCAAGGTTATCGACCTGGTTTGCCC
GTTCGCCAAGGGCGGTAAAGTCGGTCTGTTCGGTGGTGCCGGTGTGGGCAAAACCGTA
AACATGATGGAACTGATCCGTAACATCGCCATCGAGCACAGCGGTTATTCCGTGTTCG
CTGGTGTGGGTGAGCGTACTCGTGAGGGTAACGACTTCTACCACGAGATGAAGGATTC
CAACGTTCTGGACAAAGTGGCACTGGTATACGGCCAGATGAACGAGCCGCCGGGAAA
CCGTCTGCGCGTAGCTCTGACCGGCCTGACCATGGCCGAGAAGTTCCGTGACGAAGGT
AACGACGTTCTGCTGTTCGTCGACAACATCTATCGTTACACCCTGGCCGGTACCGAAGT
ATCCGCACTGCTGGGCCGTATGCCTTCGGCAGTAGGTTACCAGCCGACCCTGGCTGAA
GAGATGGGCGTTCTGCAAGAACGTATCACTTCGACCAAGCAAGGCTCGATCACCTCGA
TCCAAGCGGTATACGTACCTGCGGACGACTTGACCGACCCGTCGCCAGCGACCACCTT
CGCCCACTTGGACGCCACCGTCGTACTGTCCCGTGACATCGCTTCCCTGGGTATCTACC
CAGCGGTAGACCCACTGGATTCGACTTCCCGTCAGCTGGACCCGAACGTGATCGGCAA
CGAGCACTACGAAACCGCTCGCGGCGTTCAGTACGTGCTGCAGCGCTACAAAGAGCTG
AAGGACATCATTGCGATCCTGGGTATGGACGAACTGTCCGAAGCCGACAAGCAACTGG
TATCCCGCGCTCGTAAGATCCAGCGCTTCCTGTCGCAGCCGTTCTTCGTGGCTGAAGTC
TTCACTGGTTCTCCAGGCAAATACGTTTCCCTGAAAGACACCATCGCTGGCTTCAAAGG
CATCCTCAACGGTGACTACGACCACCTGCCAGAACAAGCGTTCTACATGGTCGGCGGC
ATCGAAGAAGCGATCGAGAAAGCCAAGAAACTGTAA

NCBI Reference Sequence: NZ_CP027719.1
Strain: P2
Chained genes: 16S rRNA, recA, gyrB, rpoD, carA, atpD

>NZ_CP027719_P2
GAACTGAAGAGTTTGATCATGGCTCAGATTGAACGCTGGCGGCAGGCCTAACACATGC
AAGTCGAGCGGTAGAGAGAAGCTTGCTTCTCTTGAGAGCGGCGGACGGGTGAGTAATG
CCTAGGAATCTGCCTGGTAGTGGGGGATAACGTTCGGAAACGGACGCTAATACCGCAT
ACGTCCTACGGGAGAAAGCAGGGGACCTTCGGGCCTTGCGCTATCAGATGAGCCTAGG
TCGGATTAGCTAGTTGGTGAGGTAATGGCTCACCAAGGCGACGATCCGTAACTGGTCT
GAGAGGATGATCAGTCACACTGGAACTGAGACACGGTCCAGACTCCTACGGGAGGCA
GCAGTGGGGAATATTGGACAATGGGCGAAAGCCTGATCCAGCCATGCCGCGTGTGTGA
AGAAGGTCTTCGGATTGTAAAGCACTTTAAGTTGGGAGGAAGGGTACTTACCTAATAC
GTGAGTATTTTGACGTTACCGACAGAATAAGCACCGGCTAACTCTGTGCCAGCAGCCG
CGGTAATACAGAGGGTGCAAGCGTTAATCGGAATTACTGGGCGTAAAGCGCGCGTAGG
TGGTTCGTTAAGTTGGATGTGAAATCCCCGGGCTCAACCTGGGAACTGCATCCAAAAC
TGGCGAGCTAGAGTATGGTAGAGGGTGGTGGAATTTCCTGTGTAGCGGTGAAATGCGT
AGATATAGGAAGGAACACCAGTGGCGAAGGCGACCACCTGGACTGATACTGACACTG
AGGTGCGAAAGCGTGGGGAGCAAACAGGATTAGATACCCTGGTAGTCCACGCCGTAA
ACGATGTCAACTAGCCGTTGGGAGCCTTGAGCTCTTAGTGGCGCAGCTAACGCATTAA
GTTGACCGCCTGGGGAGTACGGCCGCAAGGTTAAAACTCAAATGAATTGACGGGGGCC
CGCACAAGCGGTGGAGCATGTGGTTTAATTCGAAGCAACGCGAAGAACCTTACCAGGC
CTTGACATCCAATGAACTTTCCAGAGATGGATTGGTGCCTTCGGGAACATTGAGACAG
GTGCTGCATGGCTGTCGTCAGCTCGTGTCGTGAGATGTTGGGTTAAGTCCCGTAACGAG
CGCAACCCTTGTCCTTAGTTACCAGCACGTCATGGTGGGCACTCTAAGGAGACTGCCG
GTGACAAACCGGAGGAAGGTGGGGATGACGTCAAGTCATCATGGCCCTTACGGCCTGG
GCTACACACGTGCTACAATGGTCGGTACAGAGGGTTGCCAAGCCGCGAGGTGGAGCTA
ATCCCATAAAACCGATCGTAGTCCGGATCGCAGTCTGCAACTCGACTGCGTGAAGTCG
GAATCGCTAGTAATCGCGAATCAGAATGTCGCGGTGAATACGTTCCCGGGCCTTGTAC
ACACCGCCCGTCACACCATGGGAGTGGGTTGCACCAGAAGTAGCTAGTCTAACCTTCG
GGAGGACGGTTACCACGGTGTGATTCATGACTGGGGTGAAGTCGTAACAAGGTAGCCG
TAGGGGAACCTGCGGCTGGATCACCTCCTTAAATGGACGACAACAAGAAGAAAGCCTT
GGCTGCGGCCCTGGGTCAGATCGAACGTCAATTCGGCAAGGGTGCCGTAATGCGTATG
GGCGATCACGACCGCCAGGCGATCCCGGCCATTTCCACTGGCTCTCTGGGTCTGGACAT
CGCACTCGGCATCGGCGGCCTGCCAAAAGGTCGTATTGTTGAAATCTACGGTCCGGAA
TCGTCCGGTAAAACCACCCTGACCCTGTCCGTGATTGCCCAGGCACAGAAGATGGGCG
CCACCTGCGCCTTCGTCGACGCCGAGCACGCACTGGACCCGGAATACGCCGGCAAACT
GGGGGTCAACGTTGACGACCTGCTGGTTTCCCAGCCGGACACCGGCGAACAGGCGCTG
GAAATCACCGACATGCTGGTGCGCTCCAATGCCATCGACGTGATCGTGATCGACTCCG
TGGCGGCACTGGTACCCAAGGCCGAGATCGAAGGCGAGATGGGCGACATGCACGTGG
GTCTGCAGGCCCGCCTGATGTCCCAGGCGCTGCGCAAGATCACCGGTAACATCAAGAA
CGCCAACTGCCTGGTGATCTTCATCAACCAGATCCGAATGAAAATCGGCGTGATGTTC
GGCAGCCCGGAAACCACCACCGGCGGTAACGCGCTGAAGTTCTACGCTTCGGTTCGTC
TGGACATCCGTCGTACTGGCGCGGTGAAGGAAGGCGACGAAGTCGTCGGTAGCGAAA
CCCGGGTCAAGATCGTCAAGAACAAGGTGGCTCCACCGTTCCGCCAGGCTGAATTCCA
GATCCTGTACGGCAAGGGTATCTACCTGAACGGTGAGATCATCGATCTGGGCGTGCTG
CACGGTTTCCTCGAGAAGTCCGGTGCCTGGTACAGCTACCAGGGCAACAAGATCGGTC
AGGGCAAGGCCAACTCGGCCAAGTTCCTGCAGGACAATCCGGAAATCGGCAATGCCCT
CGAGAAGCAGATTCGCGACAAGCTGCTGGCTCCAACCGCTGATGTCAAAGCTTCGCCG
GTCAACGAGACCATCGATGACATGGCTGACGCGGATATCTGAATGAGCGAAGAAAAC
ACGTACGACTCGAGCAGCATTAAAGTGCTGAAAGGTTTGGATGCCGTACGCAAACGTC
CCGGTATGTACATTGGTGACACCGACGATGGCAGCGGTCTGCACCATATGGTGTTCGA
GGTGGTCGATAACTCGATCGACGAAGCTCTGGCCGGCCATTGCGACGACATCAGCATC
ATCATCCACCCGGACGAATCCATTACCGTGCGTGACAACGGTCGCGGCATCCCGGTAG
ACGTGCATAAAGAAGAAGGCGTTTCCGCGGCCGAGGTCATCATGACCGTACTGCACGC

CGGCGGTAAGTTCGACGATAACTCCTACAAAGTATCCGGCGGTCTGCACGGTGTGGGT
GTGTCGGTAGTGAACGCCCTGTCCGAAGAACTGGTCCTGACCGTTCGCCGCAGCGGAA
AGATCTGGGAACAGACCTACGTTCACGGTGTGCCTCAGGCGCCTATGGCGATCGTCGG
TGACAGCGAAACCACCGGTACCCAGATTCACTTCAAGGCGTCCAGCGAGACCTTCAAG
AACATCCATTTCAGCTGGGACATCCTGGCCAAGCGGATTCGTGAACTGTCCTTCCTCAA
CTCCGGTGTCGGTATCGTTCTGAAGGACGAACGCAGTGGCAAGGAAGAGCTGTTCAAG
TACGAAGGCGGCCTGCGTGCGTTCGTTGAATACCTGAACACCAACAAGACCGCGGTCA
ACCAGGTGTTCCACTTCAATGTGCAGCGTGAAGATGGCATCGGCGTGGAAATCGCCCT
GCAGTGGAACGACAGCTTCAACGAAAACCTGCAGTGCTTCACCAACAACATTCCGCAG
CGCGATGGCGGCACCCACTTGGTGGGCTTCCGTTCGGCACTGACGCGTAACCTGAACA
ACTACATCGAACAGGAAGGTCTGGCGAAGAAGCACAAGGTCGCCACCACCGGTGACG
ATGCCCGCGAAGGCCTGACCGCGATCATTTCGGTCAAGGTGCCGGATCCGAAGTTCAG
CTCCCAGACCAAAGACAAGCTGGTGTCTTCCGAAGTGAAGACCGCGGTTGAACAGGAA
ATGGGCAAGTACTTCTCCGACTTCCTGCTGGAAAACCCGAACGAAGCCAAGCTGGTGG
TCGGCAAGATGCTCGACGCCGCCCGTGCCCGTGAAGCGGCGCGTAAGGCTCGTGAGAT
GACCCGCCGTAAAGGCGCGCTGGATATCGCCGGCCTGCCGGGCAAACTGGCGGACTGC
CAGGAAAAAGACCCTGCCCTTTCCGAACTCTACCTGGTGGAAGGTGACTCTGCTGGCG
GCTCCGCCAAGCAGGGACGCAACCGTAAGACCCAGGCGATTCTGCCGCTCAAGGGCAA
GATCCTTAACGTCGAGAAAGCGCGCTTCGACAAGATGATTTCCTCGCAAGAGGTCGGC
ACCTTGATCACTGCACTCGGTTGCGGCATCGGCCGCGAAGAGTACAACATCGACAAGC
TGCGTTATCACAACATCATCATCATGACCGACGCTGACGTCGACGGTTCGCACATCCGT
ACCCTGCTGCTGACCTTCTTCTTCCGTCAGCTGCCGGAGCTGATCGAGCGTGGCTACAT
CTACATCGCTCAACCGCCGCTGTACAAGGTCAAGAAAGGCAAGCAAGAGCAATACATC
AAAGACGACGACGCCATGGAAGAGTACATGACGCAGTCGGCCCTGGAAGATGCGAGC
CTGCACCTGAACGAAGAAGCACCGGGTATTTCCGGCGAGGCGCTGGAGCGCCTGGTGA
ACGACTTCCGCATGGTCATGAAAACCCTCAAGCGTCTGTCGCGCCTGTACCCTCAGGA
GCTGACCGAGCACTTCATCTACCTGCCGGCCGTGAGCCTGGAGCAACTCTCCGATCAC
GCGGCCATGCAGGATTGGCTGGCCCAATATGAAGTCCGCCTGCGCACCGTCGAGAAGT
CCGGCCTGGTCTACAAGGCCAGCCTGCGTGAAGACCGTGAACGTAATGTCTGGCTGCC
AGAGGTCGAACTGATCTCCCACGGCCTGTCGAACTACGTCACCTTCAACCGCGACTTCT
TCGGTAGCAATGACTACAAGACCGTCGTTACCCTCGGCGCTCAACTGAGCTCCCTGCTG
GACGAAGGCGCTTATATTCAGCGTGGCGAACGCAAGAAGGCGGTGACCGAGTTCAAG
GAAGCCCTGGACTGGCTGATGACCGAAAGCACCAAGCGCCACACCATCCAGCGATACA
AAGGTCTGGGCGAGATGAACCCGGATCAGCTGTGGGAAACCACCATGGACCCAAGCG
TGCGCCGTATGCTCAAGGTCACGATTGAAGATGCCATCGGCGCCGACCAGATCTTCAA
CACCCTGATGGGGGATGCGGTCGAGCCTCGTCGCGACTTCATCGAAAGCAACGCCCTG
GCGGTATCCAATCTGGACTTCTGAATGTCCGGAAAAGCGCAACAGCAGTCTCGCCTCA
AAGAGTTGATCAGCCGTGGTCGTGAGCAGGGTTACCTGACTTACGCGGAGGTCAACGA
CCACCTGCCGGAGGATATTTCAGATCCGGAACAGGTGGAAGACATCATCCGCATGATC
AACGACATGGGGATCAACGTATTCGAGAGTGCTCCGGATGCGGATGCCCTTTTGTTGG
CCGAAGCCGATACCGACGAAGCAGCAGCTGAAGAAGCCGCCGCAGCGTTGGCGGCTG
TGGAAACCGACATTGGTCGCACTACCGACCCCGTGCGTATGTACATGCGCGAAATGGG
AACGGTAGAGCTGCTCACACGTGAAGGCGAAATCGAAATCGCCAAGCGTATCGAAGA
GGGCATCCGTGAAGTGATGGGCGCGATCGCGCACTTCCCTGGCACGGTTGAGCACATC
CTCTCCGAATACACTCGCGTCACCACCGAAGGTGGCCGCCTGTCCGACGTCCTGAGCG
GTTACATCGACCCGGACGACGGCATTGCGCCGCCTGCCGCCGAAGTACCACCGCCTGT
CGATGCCAAGGCTGCAAAAGCGGACGACGACACCGACGACGATGACGCCGAAGCCAG
TGACGACGAAGAAGAAGCCGAAAGCGGTCCGGATCCGGTCATCGCAGCCCAGCGCTTT
GGCGCCGTTGCCGACCAGATGGAAATCACCCGCAAGGCGCTGAAGAAGCACGGTCGC
GAACACAAGCAAGCCCTGGCCGAAATGCTGGCCCTGGCTGAACTGTTCATGCCGATCA
AACTGGTTCCGAAGCAATTCGAAGGCCTGGTTGAACGTGTTCGTAGCGCCCTGGATCG

CCTGCGTCAGCAAGAGCGCGCGATCATGCAGCTCTGTGTTCGTGATGCCCGCATGCCA
CGCGCCGACTTCCTGCGCCAGTTCCCTGGCAATGAAGTGGACGAAAGCTGGTCCGACG
CGCTGGCCAAAGGCAAGGCCAAGTACGCCGAAGCCATCGGCCGCCTGCAGCCGGACA
TCATCCGTTGCCAGCAGAAGCTGACCGCGCTCGAGACCGAGACCGGCCTGACGATCGC
CGAGATCAAGGACATCAACCGTCGCATGTCGATCGGCGAGGCCAAGGCCCGTCGCGCG
AAGAAAGAGATGGTCGAAGCCAACCTGCGTCTGGTGATCTCCATCGCCAAGAAGTACA
CCAACCGTGGCTTGCAATTCCTCGACCTGATCCAGGAAGGCAACATCGGTTTGATGAA
AGCGGTAGACAAGTTCGAATACCGTCGCGGCTACAAATTCTCGACTTATGCCACCTGG
TGGATCCGTCAGGCGATCACTCGCTCGATCGCCGACCAGGCCCGCACCATCCGTATTCC
GGTGCACATGATCGAGACGATCAACAAGCTCAACCGTATTTCCCGGCAGATGTTGCAG
GAAATGGGTCGCGAACCGACTCCGGAAGAGCTGGGCGAACGCATGGAAATGCCTGAG
GACAAGATCCGCAAGGTATTGAAGATCGCTAAAGAGCCGATCTCCATGGAAACCCCGA
TCGGTGATGACGAAGACTCCCATCTGGGTGACTTCATCGAAGACTCGACCATGCAGTC
GCCAATCGATGTCGCCACCGTTGAGAGCCTTAAAGAAGCGACTCGCGAAGTACTCTCC
GGCCTCACTGCCCGTGAAGCCAAGGTACTGCGCATGCGCTTCGGCATCGACATGAATA
CCGACCACACCCTCGAGGAAGTCGGTAAGCAGTTCGACGTTACCCGTGAGCGGATTCG
TCAGATCGAAGCCAAGGCGCTGCGCAAGCTGCGCCACCCGACGCGAAGCGAGCACCT
GCGCTCCTTCCTCGACGAGTAATTGACTAAGCCAGCCATACTCGCCCTTGCTGATGGCA
GCATTTTTCGCGGCGAAGCCATTGGAGCCGACGGTCAAACCGTTGGTGAGGTGGTGTT
TAACACCGCAATGACCGGCTATCAGGAAATCCTTACCGATCCTTCCTACGCCCAACAG
ATCGTTACCCTGACTTACCCGCATATCGGCAATACCGGCACCACGCCGGAAGACGCCG
AGTCCGATCGTGTCTGGTCGGCCGGTCTGGTGATTCGCGACCTGCCTCTGGTTGCGAGC
AACTGGCGTAACACCCTGTCCCTGTCCGACTACCTGAAAGCCAACAATGTCGTGGCGA
TCGCCGGTATCGACACCCGTCGCCTGACGCGCATCCTGCGCGAGAAAGGTGCGCAGAA
CGGCTGCATCATGGCCGGCGACAATATCTCCGACGAAGCGGCGATTGCCGCTGCACGC
GGCTTCCCGGGCCTGAAAGGCATGGATCTGGCGAAGGTCGTCAGTACCAAGGAAAGCT
ACGAGTGGCGCTCCAGTGTCTGGAACCTGAAGACCGACAGTCATCCGACCATCGAAGC
TTCCGAGCTGCCTTACCACGTGGTTGCCTACGACTACGGCGTCAAGCTGAACATCCTGC
GCATGCTGGTCGAACGCGGTTGCCGCGTGACCGTGGTGCCTGCGCAAACCCCGGCCAG
CGAAGCTCTGGCGCTCAAGCCTGACGGTGTGTTCCTGTCCAACGGCCCTGGCGACCCC
GAGCCTTGCGATTACGCCATCCAGGCGATCAAGGACGTGCTGGAGACCGAGATTCCGG
TCTTCGGTATCTGTCTGGGCCACCAACTGCTGGCACTGGCCGCCGGCGCCAAGACAGT
GAAGATGGGCCACGGCCACCACGGCGCCAACCACCCGGTCCAGGACCTGGACAGCGG
TGTGGTGATGATCACCAGCCAGAACCACGGTTTTGCGGTGGACGAAGCCACCCTGCCG
GGCAACGTGCGGGCGATCCACAAGTCGCTGTTCGACGGCACCCTGCAAGGCATCGAGC
TGACCGACAAGAGCGCATTCAGCTTCCAGGGCCACCCTGAAGCGAGCCCGGGCCCGAA
CGATGTGGCGCCGCTGTTCGATCGTTTCATCAACGAGATGGCCAAGCGACGCTGAATG
AGTAGCGGACGTATCGTTCAAATCATCGGCGCCGTTATCGACGTGGAATTTCCACGCG
ACAGCGTACCGAGCATCTACGACGCCTTGAAGGTTCAAGGCGCCGAAACCACTCTGGA
AGTTCAGCAGCAGCTGGGCGACGGCGTGGTACGTACCATTGCGATGGGCTCCACCGAG
GGCTTGAAGCGCGGTCTGGACGTCAACAACACTGGCGCAGCCATCTCCGTACCGGTCG
GTAAAGCGACCCTGGGCCGGATCATGGACGTACTGGGCAACCCGATCGACGAAGCTGG
CCCGATCGGTGAAGAAGAGCGTTGGGGCATTCACCGTCCTGCGCCGACCTTCGCTGAA
CAAGCTGGCGGCAACGACCTGCTGGAAACCGGCATCAAGGTTATCGACCTGGTTTGCC
CGTTCGCCAAGGGCGGTAAAGTCGGTCTGTTCGGTGGTGCCGGTGTGGGCAAAACCGT
AAACATGATGGAACTGATCCGTAACATCGCCATCGAGCACAGCGGTTATTCCGTGTTC
GCCGGTGTGGGTGAGCGTACTCGTGAGGGTAACGACTTCTACCACGAGATGAAGGATT
CCAACGTTCTGGACAAAGTGGCACTGGTATACGGCCAGATGAACGAGCCGCCGGGAA
ACCGTCTGCGCGTAGCTCTGACCGGCCTGACCATGGCCGAGAAGTTCCGTGACGAAGG
TAACGACGTTCTGCTGTTCGTCGACAACATCTATCGTTACACCCTGGCCGGTACCGAAG
TATCCGCACTGCTGGGCCGTATGCCTTCGGCAGTAGGTTACCAGCCGACCCTGGCTGAA

GAGATGGGCGTTCTGCAAGAACGTATCACTTCGACCAAGCAAGGCTCGATCACCTCGA
TCCAAGCGGTATACGTACCTGCGGACGACTTGACCGACCCGTCGCCAGCGACCACCTT
CGCCCACTTGGACGCCACCGTCGTTCTGTCCCGTGACATCGCTTCCCTGGGTATCTACC
CAGCGGTAGACCCACTGGACTCGACTTCCCGTCAGCTGGACCCGAACGTGATCGGCAC
CGAGCACTACGAAACCGCTCGTGGCGTTCAGTACGTGCTGCAGCGCTACAAAGAGCTG
AAGGACATCATTGCGATCCTGGGTATGGACGAACTGTCCGAAGCCGACAAGCAACTGG
TATCCCGCGCTCGTAAGATCCAGCGCTTCCTGTCGCAGCCGTTCTTCGTGGCTGAAGTC
TTCACTGGTTCTCCAGGCAAATACGTTTCCCTGAAAGACACCATCGCTGGCTTCAAAGG
CATCCTCAACGGTGACTACGACCATCTGCCAGAACAAGCGTTCTACATGGTTGGTGGC
ATCGAAGAAGCGATCGAGAAAGCCAAGAAACTGTAA

NCBI Reference Sequence: NZ_CP008696.1
Strain: PA23
Chained genes: 16S rRNA, recA, gyrB, rpoD, carA, atpD
>NZ_CP008696_PA23
AGAGTTTGATCATGGCTCAGATTGAACGCTGGCGGCAGGCCTAACACATGCAAGTCGA
GCGGTAGAGAGAAGCTTGCTTCTCTTGAGAGCGGCGGACGGGTGAGTAATGCCTAGGA
ATCTGCCTGGTAGTGGGGGATAACGTTCGGAAACGGACGCTAATACCGCATACGTCCT
ACGGGAGAAAGCAGGGGACCTTCGGGCCTTGCGCTATCAGATGAGCCTAGGTCGGATT
AGCTAGTTGGTGAGGTAATGGCTCACCAAGGCGACGATCCGTAACTGGTCTGAGAGGA
TGATCAGTCACACTGGAACTGAGACACGGTCCAGACTCCTACGGGAGGCAGCAGTGGG
GAATATTGGACAATGGGCGAAAGCCTGATCCAGCCATGCCGCGTGTGTGAAGAAGGTC
TTCGGATTGTAAAGCACTTTAAGTTGGGAGGAAGGGTACTTACCTAATACGTGAGTATT
TTGACGTTACCGACAGAATAAGCACCGGCTAACTCTGTGCCAGCAGCCGCGGTAATAC
AGAGGGTGCAAGCGTTAATCGGAATTACTGGGCGTAAAGCGCGCGTAGGTGGTTCGTT
AAGTTGGATGTGAAATCCCCGGGCTCAACCTGGGAACTGCATCCAAAACTGGCGAGCT
AGAGTATGGTAGAGGGTGGTGGAATTTCCTGTGTAGCGGTGAAATGCGTAGATATAGG
AAGGAACACCAGTGGCGAAGGCGACCACCTGGACTGATACTGACACTGAGGTGCGAA
AGCGTGGGGAGCAAACAGGATTAGATACCCTGGTAGTCCACGCCGTAAACGATGTCAA
CTAGCCGTTGGGAGCCTTGAGCTCTTAGTGGCGCAGCTAACGCATTAAGTTGACCGCCT
GGGGAGTACGGCCGCAAGGTTAAAACTCAAATGAATTGACGGGGGCCCGCACAAGCG
GTGGAGCATGTGGTTTAATTCGAAGCAACGCGAAGAACCTTACCAGGCCTTGACATCC
AATGAACTTTCCAGAGATGGATTGGTGCCTTCGGGAACATTGAGACAGGTGCTGCATG
GCTGTCGTCAGCTCGTGTCGTGAGATGTTGGGTTAAGTCCCGTAACGAGCGCAACCCTT
GTCCTTAGTTACCAGCACGTAATGGTGGGCACTCTAAGGAGACTGCCGGTGACAAACC
GGAGGAAGGTGGGGATGACGTCAAGTCATCATGGCCCTTACGGCCTGGGCTACACACG
TGCTACAATGGTCGGTACAGAGGGTTGCCAAGCCGCGAGGTGGAGCTAATCCCATAAA
ACCGATCGTAGTCCGGATCGCAGTCTGCAACTCGACTGCGTGAAGTCGGAATCGCTAG
TAATCGCGAATCAGAATGTCGCGGTGAATACGTTCCCGGGCCTTGTACACACCGCCCG
TCACACCATGGGAGTGGGTTGCACCAGAAGTAGCTAGTCTAACCTTCGGGAGGACGGT
TACCACGGTGTGATTCATGACTGGGGTGAAGTCGTAACAAGGTAGCCGTAGGGGAACC
TGCGGCTGGATCACCTCCTTAAATGGACGACAACAAGAAGAAAGCCTTGGCTGCGGCC
CTGGGTCAGATCGAACGTCAATTCGGCAAGGGTGCCGTAATGCGTATGGGCGATCACG
ACCGCCAGGCGATCCCGGCCATTTCCACTGGCTCTCTGGGTCTGGACATCGCACTCGGC
ATCGGCGGCCTGCCAAAAGGTCGTATTGTTGAAATCTACGGTCCGGAATCGTCCGGTA
AAACCACCCTGACCCTGTCCGTGATTGCCCAGGCACAGAAGATGGGCGCCACCTGCGC
CTTCGTCGACGCCGAGCACGCACTGGACCCGGAATACGCCGGCAAACTGGGGGTCAAC
GTTGACGACCTGCTGGTTTCCCAGCCGGACACCGGCGAACAGGCGCTGGAAATCACCG
ACATGCTGGTGCGCTCCAATGCCATCGACGTGATCGTGATCGACTCCGTGGCGGCACT
GGTACCCAAGGCCGAGATCGAAGGCGAGATGGGCGACATGCACGTGGGCCTGCAGGC
CCGCCTGATGTCCCAGGCGCTGCGCAAGATCACCGGTAACATCAAGAACGCCAACTGC

CTGGTGATCTTCATCAACCAGATCCGTATGAAAATCGGCGTGATGTTCGGCAGCCCGG
AAACCACCACCGGCGGTAACGCGCTGAAGTTCTACGCTTCGGTTCGTCTGGACATCCGT
CGTACTGGCGCGGTGAAGGAAGGCGACGAAGTCGTCGGTAGCGAAACCCGGGTCAAG
ATCGTCAAGAACAAGGTGGCTCCACCGTTCCGCCAGGCTGAATTCCAGATCCTGTACG
GCAAGGGTATCTACCTGAACGGTGAGATCATCGATCTGGGCGTGCTGCACGGTTTCCTC
GAGAAGTCCGGTGCCTGGTACAGCTACCAGGGCAACAAGATCGGTCAGGGCAAGGCC
AACTCGGCCAAGTTCCTGCAGGACAATCCGGAAATCGGCAATGCCCTCGAGAAGCAGA
TTCGCGACAAGCTGCTGGCTCCAACCGCTGATGTCAAAGCTTCGCCGGTCAACGAGAC
CATCGATGACATGGCTGACGCGGATATCTGAATGAGCGAAGAAAACACGTACGACTCG
AGCAGCATTAAAGTGCTGAAAGGTTTGGATGCCGTACGCAAACGTCCCGGTATGTACA
TTGGTGACACCGACGATGGCAGCGGTCTGCACCATATGGTGTTCGAGGTGGTCGATAA
CTCGATCGACGAAGCTCTGGCCGGCCATTGCGACGACATCAGCATCATCATCCACCCG
GACGAATCCATTACCGTGCGTGACAACGGTCGCGGCATCCCGGTAGACGTGCATAAAG
AAGAAGGCGTGTCCGCGGCCGAGGTCATCATGACCGTGCTGCACGCTGGTGGTAAGTT
CGACGACAACTCCTACAAAGTATCCGGCGGTCTGCACGGTGTGGGTGTGTCGGTAGTG
AACGCCCTGTCCGAAGAACTGGTCCTGACCGTTCGCCGCAGCGGAAAGATCTGGGAAC
AGACCTACGTTCACGGTGTGCCTCAGGCGCCTATGGCGATCGTCGGTGACAGCGAAAC
CACCGGTACCCAGATTCACTTCAAGGCGTCCAGCGAGACCTTCAAGAACATCCATTTC
AGCTGGGACATCCTGGCCAAGCGGATTCGTGAACTGTCCTTCCTCAACTCCGGTGTCGG
TATCGTTCTGAAGGACGAACGCAGCGGCAAGGAAGAGCTGTTCAAGTACGAAGGCGG
CCTGCGTGCGTTCGTTGAATACCTGAACACCAACAAGACCGCGGTCAACCAGGTGTTC
CACTTCAATGTGCAGCGTGAAGATGGCATCGGCGTGGAAATCGCCCTGCAGTGGAACG
ACAGCTTCAACGAAAACCTGCAGTGCTTCACCAACAACATTCCGCAGCGCGATGGCGG
CACCCACTTGGTGGGCTTCCGTTCGGCACTGACGCGTAACCTGAACAACTACATCGAA
CAGGAAGGTCTGGCGAAGAAGCACAAGGTCGCCACCACCGGTGACGATGCCCGCGAA
GGCCTGACCGCGATCATTTCGGTCAAGGTGCCGGATCCGAAGTTCAGCTCCCAGACCA
AAGACAAGCTGGTGTCTTCCGAAGTGAAGACCGCGGTTGAACAGGAAATGGGCAAGT
ACTTCTCCGACTTCCTGCTGGAAAACCCGAACGAAGCCAAGCTGGTGGTCGGCAAGAT
GCTCGACGCCGCCCGTGCCCGTGAAGCGGCGCGTAAGGCTCGTGAGATGACCCGCCGT
AAAGGCGCGCTGGATATCGCCGGCCTGCCGGGCAAACTGGCGGACTGCCAGGAAAAA
GACCCTGCCCTTTCCGAACTCTACCTGGTGGAAGGTGACTCTGCTGGCGGCTCCGCCAA
GCAGGGACGCAACCGTAAGACCCAGGCGATTCTGCCGCTCAAGGGCAAGATCCTTAAC
GTCGAGAAAGCGCGCTTCGACAAGATGATTTCCTCGCAAGAGGTCGGCACCTTGATCA
CTGCACTCGGTTGCGGCATCGGCCGCGAAGAGTACAACATCGACAAGCTGCGTTATCA
CAACATCATCATCATGACCGACGCCGACGTCGACGGTTCGCACATCCGCACCCTGCTG
CTGACCTTCTTCTTCCGTCAGCTGCCGGAGCTGATCGAGCGTGGCTACATCTACATCGC
TCAACCGCCGCTGTACAAGGTCAAGAAAGGCAAGCAAGAGCAATACATCAAAGACGA
CGACGCCATGGAAGAGTACATGACGCAGTCGGCCCTGGAAGATGCGAGCCTGCACCTG
AACGAAGAAGCACCGGGTATTTCCGGCGAGGCGCTGGAGCGCCTGGTGAACGACTTCC
GCATGGTCATGAAAACCCTCAAGCGTCTGTCGCGCCTGTACCCTCAGGAGCTGACCGA
ACACTTCATCTACCTGCCAGCCGTGAGCCTGGAGCAACTCTCCGATCACGCAGCGATG
CAGGATTGGTTGGCCCAATATGAAGTCCGCCTGCGCACCGTCGAGAAGTCCGGCCTGG
TCTACAAGGCCAGCCTGCGTGAAGACCGTGAACGTAATGTCTGGCTGCCAGAGGTCGA
ACTGATCTCCCATGGCCTGTCGAACTACGTCACCTTCAACCGCGACTTCTTCGGCAGCA
ATGACTACAAGACCGTCGTCACCCTCGGCGCTCAACTGAGCTCCCTGCTGGACGAAGG
CGCTTATATTCAGCGCGGCGAACGCAAGAAGGCGGTGACCGAGTTCAAGGAAGCCCTG
GACTGGCTGATGACCGAAAGCACCAAGCGCCACACCATCCAGCGATACAAAGGTCTGG
GCGAGATGAACCCGGACCAGCTGTGGGAAACCACCATGGATCCAAGCGTGCGCCGCAT
GCTCAAGGTCACCATCGAAGACGCCATCGGCGCCGACCAGATCTTCAACACCCTGATG
GGTGATGCGGTCGAGCCTCGTCGCGACTTCATCGAAAGCAACGCCCTGGCGGTATCCA
ACTTGGACTTCTGAATGTCCGGAAAAGCGCAACAGCAGTCTCGCCTCAAAGAGTTGAT

CAGCCGTGGTCGTGAGCAGGGTTACCTGACTTACGCGGAGGTCAACGACCACCTGCCG
GAGGATATTTCAGATCCGGAACAGGTGGAAGACATCATCCGCATGATCAACGACATGG
GGATCAACGTATTCGAGAGTGCTCCGGATGCGGATGCCCTTTTGTTGGCCGAAGCCGA
TACCGACGAAGCAGCAGCTGAAGAAGCCGCCGCAGCGTTGGCGGCCGTGGAAACCGA
CATTGGTCGCACTACCGACCCCGTGCGTATGTACATGCGCGAAATGGGAACGGTAGAG
CTGCTCACACGTGAAGGCGAAATCGAAATCGCCAAGCGTATCGAAGAGGGCATCCGTG
AAGTGATGGGCGCGATCGCGCACTTCCCTGGCACGGTTGAGCACATCCTCTCCGAATA
CACTCGCGTCACCACCGAAGGTGGCCGCCTGTCCGACGTCCTGAGCGGTTACATCGAC
CCGGACGACGGCATCGCGCCGCCTGCCGCCGAAGTACCACCGCCTGTCGATGCCAAGG
CCGCAAAAGCGGACGACGACACCGACGACGATGACGCCGAAGCCAGTGACGACGAAG
AAGAAGCCGAAAGCGGTCCGGATCCGGTCATCGCAGCCCAGCGCTTTGGCGCCGTTGC
CGACCAGATGGAAATCACCCGCAAGGCGCTGAAGAAGCACGGTCGCGAACACAAGCA
AGCCCTGGCTGAAATGCTGGCCCTGGCTGAACTGTTCATGCCGATCAAACTGGTTCCGA
AGCAATTCGAAGGCCTGGTTGAACGTGTTCGTAGCGCCCTGGATCGCCTGCGTCAGCA
AGAGCGCGCGATCATGCAGCTCTGTGTTCGTGATGCCCGCATGCCACGCGCCGACTTCC
TGCGCCAGTTCCCTGGCAATGAAGTGGACGAAAGCTGGTCCGACGCGCTGGCCAAAGG
CAAGGCCAAGTACGCCGAAGCCATCGGCCGCCTGCAGCCGGACATTATCCGTTGCCAG
CAGAAGCTGACCGCGCTTGAGACCGAGACCGGCCTGACGATCGCCGAGATCAAGGAC
ATCAACCGTCGCATGTCGATCGGCGAGGCCAAGGCCCGTCGCGCGAAGAAAGAGATG
GTCGAAGCCAACCTGCGTCTGGTGATCTCCATCGCCAAGAAGTACACCAACCGTGGCT
TGCAATTCCTCGACCTGATCCAGGAAGGCAACATCGGTTTGATGAAAGCGGTAGACAA
GTTCGAATACCGTCGCGGCTACAAATTCTCGACTTATGCCACCTGGTGGATCCGTCAGG
CGATCACTCGCTCGATCGCCGACCAGGCCCGCACCATCCGTATTCCGGTGCACATGATC
GAGACGATCAACAAGCTCAACCGTATTTCCCGGCAGATGTTGCAGGAAATGGGTCGCG
AACCGACTCCGGAAGAGCTGGGCGAACGCATGGAAATGCCTGAGGACAAGATCCGCA
AGGTATTGAAGATCGCTAAAGAGCCGATCTCCATGGAAACCCCGATCGGTGATGACGA
AGACTCCCATCTGGGTGACTTCATCGAAGACTCGACCATGCAGTCGCCAATCGATGTC
GCCACCGTCGAGAGTCTTAAAGAAGCGACTCGCGAAGTACTCTCCGGCCTCACTGCCC
GTGAAGCCAAGGTACTGCGCATGCGCTTCGGCATCGACATGAATACCGACCACACCCT
CGAGGAAGTCGGTAAGCAGTTTGACGTTACCCGTGAGCGGATTCGTCAGATCGAAGCC
AAGGCGCTGCGCAAGCTGCGCCACCCGACGCGAAGCGAGCACCTGCGCTCCTTCCTCG
ACGAGTAATTGACTAAGCCAGCCATACTCGCCCTTGCTGATGGCAGCATTTTTCGCGGC
GAAGCCATTGGAGCCGACGGTCAAACCGTTGGTGAGGTGGTGTTTAACACCGCAATGA
CCGGCTATCAGGAAATCCTTACCGATCCTTCCTACGCCCAACAGATCGTTACCCTGACT
TACCCGCATATCGGCAATACCGGCACCACGCCGGAAGACGCCGAGTCCGATCGTGTCT
GGTCGGCCGGTCTGGTGATTCGCGACCTGCCTCTGGTTGCGAGCAACTGGCGTAACAC
CCTGTCCCTGTCCGACTACCTGAAAGCCAACAATGTCGTGGCAATCGCCGGTATCGAC
ACCCGTCGCCTGACGCGCATCCTGCGCGAGAAAGGTGCGCAGAACGGCTGCATCATGG
CCGGCGACAATATCTCCGACGAAGCGGCGATTGCCGCTGCACGCGGCTTCCCGGGCCT
GAAAGGCATGGATCTGGCGAAGGTCGTCAGTACCAAGGAAAGCTACGAGTGGCGCTC
CAGTGTCTGGAACCTGAAGACCGACAGTCATCCGACCATCGAAGCTTCCGAGCTGCCT
TACCACGTGGTTGCCTACGACTACGGCGTCAAGCTGAACATCCTGCGCATGCTGGTCG
AACGCGGTTGCCGCGTGACCGTGGTGCCTGCGCAAACCCCGGCCAGCGAAGCTCTGGC
GCTCAAGCCTGACGGTGTGTTCCTGTCCAACGGCCCTGGCGACCCCGAGCCTTGCGATT
ACGCCATCCAGGCGATCAAGGACGTGCTGGAGACCGAGATTCCGGTCTTCGGTATCTG
TCTGGGCCACCAACTGCTGGCACTGGCCGCCGGCGCCAAGACAGTGAAGATGGGCCAC
GGCCACCACGGCGCCAACCACCCGGTCCAGGACCTGGACAGCGGTGTGGTGATGATCA
CCAGCCAGAACCACGGTTTTGCGGTGGACGAAGCCACCCTGCCGGGCAACGTGCGGGC
GATCCACAAGTCGCTGTTCGACGGCACCCTGCAAGGCATCGAGCTGATCGACAAGAGC
GCATTCAGCTTCCAGGGCCACCCTGAAGCGAGCCCGGGCCCGAACGATGTGGCGCCGC
TGTTCGATCGTTTCATCAACGAGATGGCCAAGCGACGCTGAATGAGTAGCGGACGTAT

CGTTCAAATCATCGGCGCCGTTATCGACGTGGAATTTCCACGCGACAGCGTACCGAGC
ATCTACGACGCCTTGAAGGTTCAAGGCGCCGAAACCACTCTGGAAGTTCAGCAGCAGC
TGGGCGACGGCGTGGTACGTACCATTGCGATGGGCTCCACCGAGGGCTTGAAGCGCGG
TCTGGACGTCAACAACACTGGCGCAGCCATCTCCGTACCGGTCGGTAAAGCGACCCTG
GGCCGGATCATGGACGTACTGGGCAACCCGATCGACGAAGCTGGCCCGATCGGCGAA
GAAGAGCGTTGGGGCATTCACCGTCCTGCGCCGACCTTCGCTGAACAAGCTGGCGGCA
ACGACCTGCTGGAAACCGGCATCAAGGTTATCGACCTGGTTTGCCCGTTCGCCAAGGG
CGGTAAAGTCGGTCTGTTCGGTGGTGCCGGTGTGGGCAAAACCGTAAACATGATGGAA
CTGATCCGTAACATCGCCATCGAGCACAGCGGTTATTCCGTGTTCGCCGGTGTGGGTGA
GCGTACTCGTGAGGGTAACGACTTCTACCACGAGATGAAGGATTCCAACGTTCTGGAC
AAAGTGGCACTGGTATACGGCCAGATGAACGAGCCGCCGGGAAACCGTCTGCGCGTA
GCTCTGACCGGCCTGACCATGGCCGAGAAGTTCCGTGACGAAGGTAACGACGTTCTGC
TGTTCGTCGACAACATCTATCGTTACACCCTGGCCGGTACCGAAGTATCCGCACTGCTG
GGCCGTATGCCTTCGGCAGTAGGTTACCAGCCGACCCTGGCTGAAGAGATGGGCGTTC
TGCAAGAACGTATCACTTCGACCAAGCAAGGCTCGATCACCTCGATCCAAGCGGTATA
CGTACCTGCGGACGACTTGACCGACCCGTCGCCAGCGACCACCTTCGCCCACTTGGAC
GCCACCGTCGTTCTGTCCCGTGACATCGCTTCCCTGGGTATCTACCCAGCGGTAGACCC
ACTGGACTCGACTTCCCGCCAGCTGGACCCGAACGTGATCGGCACCGAGCACTACGAA
ACCGCTCGTGGCGTTCAGTACGTGCTGCAGCGCTACAAAGAGCTGAAGGACATCATTG
CGATCCTGGGTATGGACGAACTGTCCGAAGCCGACAAGCAACTGGTATCCCGCGCTCG
TAAGATCCAGCGCTTCCTGTCGCAGCCGTTCTTCGTGGCTGAAGTCTTCACTGGTTCTC
CAGGCAAATACGTTTCCCTGAAAGACACCATCGCTGGCTTCAAAGGCATCCTCAACGG
TGACTACGACCATCTGCCAGAACAAGCGTTCTACATGGTTGGTGGCATCGAAGAAGCG
ATCGAGAAAGCCAAGAAACTGTAA

NCBI Reference Sequence: NC_007492.2
Strain: Pf01
Chained genes: 16S rRNA, recA, gyrB, rpoD, carA, atpD
>NC_007492_Pf01
GAACTGAAGAGTTTGATCATGGCTCAGATTGAACGCTGGCGGCAGGCCTAACACATGC
AAGTCGAGCGGATGAAGGGAGCTTGCTCCTGGATTCAGCGGCGGACGGGTGAGTAATG
CCTAGGAATCTGCCTGGTAGTGGGGGACAACGTTTCGAAAGGAACGCTAATACCGCAT
ACGTCCTACGGGAGAAAGCAGGGGACCTTCGGGCCTTGCGCTATCAGATGAGCCTAGG
TCGGATTAGCTAGTTGGTGAGGTAATGGCTCACCAAGGCGACGATCCGTAACTGGTCT
GAGAGGATGATCAGTCACACTGGAACTGAGACACGGTCCAGACTCCTACGGGAGGCA
GCAGTGGGGAATATTGGACAATGGGCGAAAGCCTGATCCAGCCATGCCGCGTGTGTGA
AGAAGGTCTTCGGATTGTAAAGCACTTTAAGTTGGGAGGAAGGGTTGTAGATTAATAC
TCTGCAATTTTGACGTTACCGACAGAATAAGCACCGGCTAACTCTGTGCCAGCAGCCG
CGGTAATACAGAGGGTGCAAGCGTTAATCGGAATTACTGGGCGTAAAGCGCGCGTAGG
TGGTTCGTTAAGTTGGATGTGAAATCCCCGGGCTCAACCTGGGAACTGCATCCAAAAC
TGGCGAGCTAGAGTATGGTAGAGGGTGGTGGAATTTCCTGTGTAGCGGTGAAATGCGT
AGATATAGGAAGGAACACCAGTGGCGAAGGCGACCACCTGGACTGATACTGACACTG
AGGTGCGAAAGCGTGGGGAGCAAACAGGATTAGATACCCTGGTAGTCCACGCCGTAA
ACGATGTCAACTAGCCGTTGGGAGCCTTGAGCTCTTAGTGGCGCAGCTAACGCATTAA
GTTGACCGCCTGGGGAGTACGGCCGCAAGGTTAAAACTCAAATGAATTGACGGGGGCC
CGCACAAGCGGTGGAGCATGTGGTTTAATTCGAAGCAACGCGAAGAACCTTACCAGGC
CTTGACATCCAATGAACTTTCCAGAGATGGATTGGTGCCTTCGGGAGCATTGAGACAG
GTGCTGCATGGCTGTCGTCAGCTCGTGTCGTGAGATGTTGGGTTAAGTCCCGTAACGAG
CGCAACCCTTGTCCTTAGTTACCAGCACGTTATGGTGGGCACTCTAAGGAGACTGCCGG
TGACAAACCGGAGGAAGGTGGGGATGACGTCAAGTCATCATGGCCCTTACGGCCTGGG
CTACACACGTGCTACAATGGTCGGTACAAAGGGTTGCCAAGCCGCGAGGTGGAGCTAA

TCCCATAAAACCGATCGTAGTCCGGATCGCAGTCTGCAACTCGACTGCGTGAAGTCGG
AATCGCTAGTAATCGCGAATCAGAATGTCGCGGTGAATACGTTCCCGGGCCTTGTACA
CACCGCCCGTCACACCATGGGAGTGGGTTGCACCAGAAGTAGCTAGTCTAACCTTCGG
GAGGACGGTTACCACGGTGTGATTCATGACTGGGGTGAAGTCGTAACAAGGTAGCCGT
AGGGGAACCTGCGGCTGGATCACCTCCTTAAATGGACGACAACAAGAAGAAAGCCTTG
GCTGCGGCCCTGGGTCAGATCGAACGTCAATTCGGCAAGGGTGCCGTAATGCGTATGG
GCGATCAGGACCGTCAGGCGATCCCGGCCATTTCCACCGGCTCTCTGGGTCTGGACATC
GCACTCGGCATCGGCGGCCTGCCAAAAGGCCGTATCGTTGAAATCTACGGTCCTGAAT
CTTCCGGTAAAACCACACTGACGCTGTCCGTGATCGCCCAGGCTCAAAAAGCCGGTGC
GACCTGCGCCTTCGTCGACGCCGAACACGCCCTCGACCCAGAGTACGCCGGCAAGCTG
GGCGTCAATGTCGACGACCTGCTGGTTTCCCAGCCGGACACCGGCGAGCAGGCCCTGG
AAATCACCGACATGCTGGTGCGCTCCAACGCCGTTGACGTGATCATCGTCGACTCCGTG
GCCGCTCTGGTACCGAAGGCAGAAATCGAAGGCGAAATGGGTGACATGCACGTGGGC
CTGCAAGCCCGTCTGATGTCCCAGGCGCTGCGTAAAATCACCGGTAACATCAAGAACG
CCAACTGCCTGGTGATCTTCATCAACCAGATCCGCATGAAGATCGGCGTGATGTTCGGC
AGCCCGGAAACCACCACCGGTGGTAACGCGCTGAAGTTCTACGCCTCGGTTCGTCTCG
ACATCCGCCGTACCGGCGCGGTGAAGGAAGGCGACGAAGTGGTCGGCAGCGAAACCC
GCGTCAAGGTTGTGAAGAACAAGGTGGCTTCGCCGTTCCGTCAGGCCGAGTTCCAGAT
TCTCTACGGCAAGGGTATCTACCTGAACGGCGAGATGATCGACCTGGGCGTTCTGCAC
GGGTTCGTCGAGAAGTCCGGCGCCTGGTATGCCTACGAAGGCACCAAGATCGGTCAGG
GCAAGGCCAACTCGGCCAAGTTCCTGGCGGACAACCCGGAAGTCGCGGCCAAGCTCGA
GAAGCAACTGCGTGACAAGCTGCTGTCGCCAGCCGTGATCGCCGACTCCAAGGCTTCT
GCGGTCAAAGAGACCGAAGACGACCTGGCTGACGCTGACATCTGATTGAGCGAAGAA
AATACGTACGACTCATCGAGCATTAAAGTGCTGAAAGGCCTGGATGCCGTGCGCAAAC
GTCCCGGTATGTACATTGGTGACACCGACGATGGCAGCGGTCTGCACCACATGGTGTT
CGAGGTGGTCGACAACTCGATCGACGAAGCCCTCGCCGGCCACTGCGACGACATCAGC
ATCATCATCCACCCGGATGAGTCCATCACCGTTAAAGACAACGGCCGTGGCATCCCGG
TAGACGTGCACAAAGAGGAAGGCGTTTCCGCCGCCGAGGTCATCATGACCGTCCTCCA
CGCCGGCGGTAAGTTTGACGACAACTCCTACAAGGTATCCGGTGGTCTGCACGGTGTA
GGTGTTTCGGTCGTGAACGCGCTGTCTGAAGAACTGGTCCTGACCGTGCGCCGCAGCG
GCAAGATCTGGGAACAGACCTACGTCCACGGCGTGCCTCAGGCACCGATGGCGATCGT
TGGCGACAGCGAAACCACTGGCACCCAGATTCACTTCAAGGCTTCCAGCGAAACCTTC
AAGAACATTCACTTCAGCTGGGATATCCTGGCCAAGCGCATTCGTGAACTGTCTTTCCT
CAACTCCGGTGTAGGCATCGTCCTCAAGGACGAGCGCAGCGGCAAGGAAGAACTGTTC
AAGTACGAAGGCGGCCTGCGTGCGTTCGTTGAATACCTGAACACCAACAAGACTGCGG
TCAACCAGGTGTTCCACTTCAACATCCAGCGTGAAGACGGCATCGGCGTGGAAATCGC
CCTGCAGTGGAACGACAGCTTCAACGAGAACCTGTTGTGCTTCACCAACAACATTCCG
CAGCGCGACGGTGGCACTCACCTGGTGGGCTTCCGCTCGGCACTGACGCGTAACCTGA
ACAACTACATCGAGCAGGAAGGCCTGGCGAAGAAGCACAAAGTCGCCACCACCGGTG
ACGATGCCCGTGAAGGCCTGACCGCGATCATCTCGGTGAAGGTGCCGGATCCGAAGTT
CAGCTCCCAGACCAAAGACAAGCTGGTTTCTTCCGAAGTGAAGACCGCGGTCGAACAG
GAAATGGGCAAGTACTTCTCCGACTTCCTGCTGGAAAACCCGAACGAAGCCAAACTGG
TCGTCGGCAAGATGATCGACGCTGCCCGTGCCCGTGAAGCGGCGCGCAAGGCCCGTGA
GATGACCCGCCGCAAAGGCGCGCTGGACATCGCCGGCCTGCCGGGCAAGCTCGCTGAC
TGCCAGGAAAAAGACCCGGCGCTGTCCGAACTGTACCTCGTGGAAGGTGACTCCGCGG
GCGGCTCTGCCAAGCAGGGACGTAACCGCAAGACCCAGGCCATCCTGCCGCTGAAGGG
CAAGATCCTCAACGTCGAGAAAGCCCGTTTCGACAAGATGATCTCGTCCCAGGAAGTG
GGCACCCTGATCACCGCGCTGGGCTGCGGTATCGGTCGCGAAGAGTACAACATCGACA
AGCTGCGCTATCACAACATCATCATCATGACCGATGCTGACGTCGACGGTTCGCACATC
CGTACCCTGCTGCTGACCTTCTTCTTCCGTCAGTTGCCTGAGCTGATCGAGCGTGGCTA
CATCTACATCGCCCAGCCGCCGCTGTACAAGGTCAAGAAAGGCAAGCAAGAGCAATAC

ATCAAAGACGACGACGCCATGGAAGAGTACATGACGCAGTCGGCCCTGGAAGATGCG
AGCCTGCACCTGAACGAAGACGCCCCGGGCATTTCCGGCGAAGCGCTGGAACGTCTGG
TGAACGACTTCCGCATGGTGATGAAGACCCTCAAGCGTCTGTCGCGCCTGTACCCGCA
GGAGCTGACCGAGCACTTCATCTACCTGCCGGCCGTCAGCCTGGAAATGCTCGGCGAC
CACGCGAAGATGCAGGACTGGCTGGCCCAGTACGAAGTCCGTCTGCGCACCGTCGAGA
AGTCGGGCCTGGTCTACAAGGCCAGCCTGCGTGAAGATCGTGAACGTGGCGTTTGGCT
GCCAGAGGTCGAACTGATCTCCCACGGTCTGTCGAACTATGTCACCTTTAACCGCGACT
TCTTCGGCAGCAACGACTACAAGACCGTCGTCACCCTCGGCGCTCAACTGAGCACCCTT
CTTGATGAAGGCGCATACATCCAGCGTGGCGAGCGTAAAAAAGCGGTCACTGAGTTCA
AGGAAGCCCTGGACTGGCTGATGGCCGAAAGCACCAAGCGCCACACCATTCAGCGATA
CAAAGGTCTGGGCGAAATGAACCCGGATCAGCTGTGGGAAACCACCATGGACCCAAG
CGTGCGCCGCATGCTGAAAGTCACCATCGAAGACGCCATTGGCGCAGACCAGATCTTC
AACACCCTGATGGGTGATGCGGTCGAACCTCGCCGTGACTTCATCGAGAGCAACGCTC
TGGCGGTGTCCAACCTGGATTTCTGAATGTCCGGAAAAGCGCAACAGCAGTCTCGTAT
TATTGAGTTGATCAAACTGGGTCGTGAGCAGAAGTATCTGACTTACGCCGAGGTCAAC
GACCACCTGCCCGAGGATATTTCAGATCCGGAGCAGGTGGAAGACATCATCCGCATGA
TTAACGACATGGGGATCCCCGTACACGAGAGTGCTCCGGATGCGGACGCCCTTATGCT
GGCCGACGCCGATACCGACGAGGCCGCTGCGGAAGAAGCAGCCGCTGCGTTGGCGGC
GGTGGAGACCGATATCGGTCGCACCACTGACCCGGTGCGCATGTACATGCGTGAAATG
GGTACGGTCGAGCTTCTGACTCGTGAAGGCGAAATCGAAATCGCCAAGCGTATCGAAG
AAGGCATCCGTGAAGTGATGAGCGCCATCGCGCACTTCCCTGGCACGGTTGACCATAT
TCTCTCCGAGTACACTCGCGTCACCACCGAAGGTGGTCGCCTGTCCGACGTTCTGAGCG
GTTACATCGACCCGGACGACGGCATTACGCCGCCTGCCGCCGAAGTACCGCCACCGAT
CGACGCGAAAGCCGCGAAAGCGGATGACGACTCCGAGGACGATGACGCCGAAGCTTC
CGATGACGAAGAAGAAGCCGAAAGCGGTCCGGATCCGGTCATTGCCGCACAGCGCTTC
GGTGCTGTCGCCGACCAGATGGAAATCACCCGCAAGGCCCTGAAAAAGCACGGTCGTC
ACAACAAGGCGGCAATTGCCGAACTGTTGGCCCTGGCCGAGCTGTTCATGCCGATCAA
GCTGGTGCCGAAGCAGTTCGAAGCCCTGGTCGAGCGTGTTCGCAGCGCCCTGGATCGC
CTGCGTCAGCAAGAGCGCGCGATCATGCAACTGTGCGTACGTGATGCACGTATGCCTC
GTGCCGACTTCCTGCGCCAGTTCCCGGGCAACGAAGTCGACGAAAGCTGGTCCGACGC
CCTGGCCAAAGGCAAGAGCAAGTACGCCGAAGCCATCGCCCGCGTGCAACCGGACAT
CATCCGTTGCCAGCAAAAGCTGACCGCGCTGGAAACCGAGACCGGTTTGACCATCGCC
GAGATCAAGGACATCAACCGTCGCATGTCGATCGGTGAGGCGAAAGCCCGCCGCGCG
AAGAAAGAGATGGTTGAAGCGAACTTGCGTCTGGTGATCTCCATCGCCAAGAAGTACA
CCAACCGTGGCCTGCAATTCCTCGACCTGATCCAGGAAGGCAACATCGGTCTGATGAA
AGCGGTGGACAAGTTCGAATACCGTCGTGGTTACAAGTTCTCGACTTACGCCACCTGGT
GGATCCGTCAGGCGATCACTCGCTCGATCGCCGACCAGGCCCGCACCATCCGTATTCC
GGTGCACATGATCGAGACGATCAACAAGCTCAACCGTATTTCCCGGCAGATGCTGCAG
GAAATGGGTCGCGAACCGACCCCGGAAGAGCTGGGTGAACGCATGGAAATGCCTGAG
GACAAGATCCGCAAGGTATTGAAGATCGCCAAAGAGCCGATCTCCATGGAAACCCCGA
TCGGTGATGACGAAGACTCTCATCTGGGTGACTTCATCGAAGACTCGACCATGCAGTC
GCCAATCGATGTCGCCACTGTCGAGAGCCTGAAAGAAGCGACCCGCGAAGTGCTGTCC
GGCCTTACTGCCCGTGAAGCCAAGGTACTGCGCATGCGTTTCGGTATCGACATGAACA
CCGACCATACGCTTGAAGAAGTCGGCAAACAGTTTGACGTGACCCGCGAGCGGATCCG
TCAGATCGAAGCCAAGGCGCTGCGCAAGCTGCGCCACCCGACGCGAAGCGAGCATCTG
CGCTCCTTCCTCGACGAGTGATTGACTAAGCCAGCCATACTCGCCCTTGCTGATGGCAG
CATTTTTCGCGGCGAAGCCATTGGAGCCGACGGTCAGACCGTTGGTGAGGTGGTGTTC
AACACCGCAATGACCGGCTATCAGGAAATCCTTACCGATCCTTCCTACGCCCAACAGA
TCGTTACCCTGACTTACCCGCACATCGGCAACACCGGCACCACGCCGGAAGACGCCGA
GTCCGATCGCGTCTGGTCCGCTGGCCTGGTCATTCGTGACCTGCCGCTGGTAGCGAGCA
ACTGGCGTAACACGATGTCCCTGTCCGATTACCTGAAAGCCAACAATGTTGTGGCGAT

CGCCGGTATCGACACCCGCCGCCTGACCCGCATCCTGCGTGAAAAAGGCGCACAGAAC
GGCTGCATCATGGCCGGCGACAACATCTCCGAAGAGGCGGCCATCGCCGCGGCGCAAG
GCTTCCCGGGCCTGAAGGGCATGGATCTGGCGAAAGTCGTCAGCACCAAGACCCAATA
CGAATGGCGCTCCACTGTCTGGGATCTGAAAACCGACAGCCACGCGACCATCGAAGCC
TCCGAGCTGCCTTACCACGTGGTTGCCTACGACTACGGCGTCAAGGTCAACATCCTGCG
CATGTTGGTCGAGCGCGGCTGCCGCGTCACTGTCGTTCCGGCACAGACCCCGGCGGCC
GACGTGCTGGCCTTGAAGCCGGACGGCGTGTTCCTGTCCAACGGTCCTGGTGATCCGG
AGCCTTGCGACTACGCGATCCAAGCGATCAAGGAAGTGCTGGAAACCGAAATTCCAGT
CTTCGGCATCTGCCTCGGTCACCAGCTGCTGGCTCTGGCCTCCGGCGCCAAGACCCTGA
AAATGGGCCACGGCCACCACGGTGCCAACCACCCGGTGCAGGATCTGGACACTGGCGT
CGTGATGATCACCAGCCAGAACCACGGTTTCGCGGTTGACGAAGAAACCCTGCCAGCC
AACGTCCGCGCGATCCATAAATCGCTGTTCGACGGCACCCTGCAAGGCATCGAGCGCA
CCGACAAGAGCGCGTTCAGCTTCCAGGGTCACCCTGAGGCGAGCCCGGGCCCGAACGA
TGTGGCCCCTCTGTTTGACCGCTTCATCAACGAGATGGCCAAGCGACGCTAAATGAGT
AGCGGACGTATCGTTCAAATCATCGGCGCCGTTATCGACGTGGAATTTCCACGCGACA
GCGTACCGAGCATCTACGACGCCTTGAAGGTTCAAGGCGCCGAAACCACTCTGGAAGT
TCAGCAGCAGCTGGGCGACGGCGTGGTTCGTACCATTGCGATGGGCTCCACCGAAGGC
TTGAAGCGCGGTCTGGACGTCAACAACACTGGCGCAGCCATCTCCGTACCGGTCGGTA
AAGCGACTCTGGGCCGGATCATGGACGTACTGGGCAACCCGATCGACGAAGCTGGCCC
GATCGGCGAAGAAGAGCGCTGGGGTATCCACCGCGCCGCTCCTTCCTTCGCTGAACAA
GCCGGTGGCAACGAGCTGCTGGAAACAGGCATCAAGGTTATCGACCTGGTTTGCCCGT
TCGCCAAGGGCGGTAAAGTCGGTCTGTTCGGTGGTGCCGGTGTAGGCAAGACCGTAAA
CATGATGGAACTGATCCGTAACATCGCCATCGAGCACAGCGGTTATTCCGTGTTCGCCG
GTGTGGGTGAGCGTACTCGTGAGGGTAACGACTTCTACCACGAGATGAAGGACTCCAA
CGTTCTCGACAAGGTAGCCCTGGTCTACGGTCAGATGAACGAGCCACCGGGAAACCGT
CTGCGCGTAGCGCTGACCGGTCTGACCATGGCCGAGAAGTTCCGTGACGAAGGTAACG
ACGTTCTGCTGTTCGTCGACAACATCTATCGTTACACCCTGGCCGGTACCGAAGTATCC
GCACTGCTGGGCCGTATGCCTTCGGCAGTAGGTTACCAGCCGACCCTGGCTGAAGAGA
TGGGCGTGCTGCAAGAGCGCATCACTTCGACCAAGCAAGGTTCGATTACTTCGATCCA
GGCCGTATACGTACCAGCGGACGACTTGACTGACCCGTCGCCAGCGACCACGTTTGCT
CACCTGGACGCCACCGTCGTTCTGTCCCGTGACATCGCTTCTCTGGGTATCTACCCGGC
GGTAGACCCACTGGACTCGACTTCGCGTCAGCTGGACCCGAACGTGATCGGCAACGAT
CACTACGAGACCGCTCGTGGTGTTCAGTACGTGCTGCAGCGTTACAAAGAGCTGAAGG
ACATCATCGCGATCCTGGGTATGGACGAGCTGTCGGAAGCCGACAAGCAGTTGGTAAA
CCGTGCTCGTAAGATCCAGCGCTTCTTGTCGCAGCCGTTCTTCGTGGCTGAAGTCTTCA
CCGGTGCTTCGGGTAAATACGTTTCCCTGAAAGACACCATTGCTGGCTTCAAAGGCATC
CTCAACGGTGACTACGACCACCTGCCAGAACAAGCGTTCTACATGGTCGGCGGCATCG
AAGAAGCGATCGAGAAAGCCAAGAAACTGTAA

NCBI Reference Sequence: NZ_CP048051.1
Strain: zm-1
Chained genes: 16S rRNA, recA, gyrB, rpoD, carA, atpD
>NZ_CP048051_zm1
GAACTGAAGAGTTTGATCATGGCTCAGATTGAACGCTGGCGGCAGGCCTAACACATGC
AAGTCGAGCGGTAGAGAGAAGCTTGCTTCTCTTGAGAGCGGCGGACGGGTGAGTAATG
CCTAGGAATCTGCCTGGTAGTGGGGGATAACGTCCGGAAACGGACGCTAATACCGCAT
ACGTCCTACGGGAGAAAGCAGGGGACCTTCGGGCCTTGCGCTATCAGATGAGCCTAGG
TCGGATTAGCTAGTTGGTGAGGTAATGGCTCACCAAGGCGACGATCCGTAACTGGTCT
GAGAGGATGATCAGTCACACTGGAACTGAGACACGGTCCAGACTCCTACGGGAGGCA
GCAGTGGGGAATATTGGACAATGGGCGAAAGCCTGATCCAGCCATGCCGCGTGTGTGA
AGAAGGTCTTCGGATTGTAAAGCACTTTAAGTTGGGAGGAAGGGTACTTACCTAATAC

GTGAGTATTTTGACGTTACCGACAGAATAAGCACCGGCTAACTCTGTGCCAGCAGCCG
CGGTAATACAGAGGGTGCAAGCGTTAATCGGAATTACTGGGCGTAAAGCGCGCGTAGG
TGGTTCGTTAAGTTGGATGTGAAATCCCCGGGCTCAACCTGGGAACTGCATCCAAAAC
TGGCGAGCTAGAGTATGGTAGAGGGTGGTGGAATTTCCTGTGTAGCGGTGAAATGCGT
AGATATAGGAAGGAACACCAGTGGCGAAGGCGACCACCTGGACTGATACTGACACTG
AGGTGCGAAAGCGTGGGGAGCAAACAGGATTAGATACCCTGGTAGTCCACGCCGTAA
ACGATGTCAACTAGCCGTTGGGAGCCTTGAGCTCTTAGTGGCGCAGCTAACGCATTAA
GTTGACCGCCTGGGGAGTACGGCCGCAAGGTTAAAACTCAAATGAATTGACGGGGGCC
CGCACAAGCGGTGGAGCATGTGGTTTAATTCGAAGCAACGCGAAGAACCTTACCAGGC
CTTGACATCCAATGAACTTTCCAGAGATGGATTGGTGCCTTCGGGAACATTGAGACAG
GTGCTGCATGGCTGTCGTCAGCTCGTGTCGTGAGATGTTGGGTTAAGTCCCGTAACGAG
CGCAACCCTTGTCCTTAGTTACCAGCACGTTATGGTGGGCACTCTAAGGAGACTGCCGG
TGACAAACCGGAGGAAGGTGGGGATGACGTCAAGTCATCATGGCCCTTACGGCCTGGG
CTACACACGTGCTACAATGGTCGGTACAGAGGGTTGCCAAGCCGCGAGGTGGAGCTAA
TCCCATAAAACCGATCGTAGTCCGGATCGCAGTCTGCAACTCGACTGCGTGAAGTCGG
AATCGCTAGTAATCGCGAATCAGAATGTCGCGGTGAATACGTTCCCGGGCCTTGTACA
CACCGCCCGTCACACCATGGGAGTGGGTTGCACCAGAAGTAGCTAGTCTAACCTTCGG
GAGGACGGTTACCACGGTGTGATTCATGACTGGGGTGAAGTCGTAACAAGGTAGCCGT
AGGGGAACCTGCGGCTGGATCACCTCCTTAAATGGACGACAACAAGAAGAAAGCCTTG
GCTGCGGCCCTGGGTCAGATCGAACGTCAATTCGGCAAGGGTGCCGTAATGCGTATGG
GCGATCACGACCGCCAGGCGATCCCGGCCATTTCCACTGGCTCTCTGGGTCTGGACATC
GCACTCGGCATCGGCGGCCTGCCAAAAGGCCGTATTGTTGAAATCTACGGTCCGGAAT
CGTCCGGTAAAACCACCCTGACCCTGTCGGTGATTGCCCAGGCACAGAAGATGGGCGC
CACCTGCGCCTTCGTCGACGCCGAGCACGCACTGGACCCGGAATACGCAGGCAAACTG
GGGGTCAACGTTGACGACCTGCTGGTTTCCCAGCCGGACACCGGCGAACAGGCGCTGG
AAATCACCGACATGCTGGTGCGCTCCAATGCCATCGACGTGATCGTGATCGACTCCGT
GGCGGCACTGGTACCCAAGGCCGAGATCGAAGGCGAGATGGGCGACATGCACGTGGG
CCTGCAGGCCCGCCTGATGTCCCAGGCGCTGCGCAAGATCACCGGTAACATCAAGAAC
GCCAACTGCCTGGTGATCTTCATCAACCAGATCCGTATGAAAATCGGCGTGATGTTCGG
CAGCCCGGAAACCACCACCGGTGGTAACGCGCTGAAGTTCTACGCTTCGGTTCGTCTG
GACATCCGTCGTACTGGCGCGGTGAAGGAAGGCGACGAAGTCGTCGGTAGCGAAACC
CGGGTCAAGATCGTCAAGAACAAGGTGGCTCCACCGTTCCGTCAGGCTGAATTCCAGA
TCCTGTACGGCAAGGGTATCTACCTGAACGGCGAGATCATCGATCTGGGCGTGCTGCA
CGGTTTCCTCGAGAAGTCCGGTGCCTGGTACAGCTACCAGGGCAACAAGATCGGTCAG
GGCAAGGCCAACTCGGCCAAGTTCCTGCAGGACAATCCGGAAATCGGCAATGCCCTCG
AGAAGCAGATTCGCGACAAGCTGCTGGCTCCAACCGCTGATGTCAAAGCTTCGCCGGT
CAACGAGACCATCGATGACATGGCTGACGCGGATATCTGAATGAGCGAAGAAAACAC
GTACGACTCGAGCAGCATTAAAGTGCTGAAAGGTTTGGATGCCGTACGCAAACGTCCC
GGTATGTACATTGGTGACACCGACGATGGCAGCGGTCTGCACCATATGGTGTTCGAGG
TGGTCGATAACTCGATCGACGAAGCTCTGGCCGGCCACTGCGACGACATCAGCATCAT
CATCCACCCGGACGAATCCATTACCGTGCGTGACAACGGTCGCGGCATCCCGGTAGAC
GTGCATAAAGAAGAAGGCGTTTCCGCGGCCGAGGTCATCATGACTGTGCTGCACGCCG
GCGGTAAGTTCGACGACAACTCCTACAAAGTATCCGGCGGTCTGCACGGTGTGGGTGT
GTCGGTAGTGAACGCCCTGTCCGAAGAACTGGTCCTGACCGTTCGCCGCAGTGGCAAG
ATCTGGGAACAGACCTACGTTCACGGTGTGCCTCAGGCGCCTATGGCGATCGTCGGTG
ACAGTGAAACCACCGGTACCCAGATTCACTTCAAGGCTTCCAGCGAGACCTTCAAGAA
CATCCATTTCAGCTGGGACATCCTGGCCAAGCGGATTCGTGAACTGTCCTTCCTCAACT
CCGGTGTCGGTATCGTTCTGAAGGACGAGCGTAGCGGCAAGGAAGAACTGTTCAAGTA
CGAAGGCGGTCTGCGTGCGTTCGTTGAATACCTGAACACCAACAAGACCGCGGTCAAC
CAGGTGTTCCATTTCAATGTGCAGCGTGAAGATGGCATCGGCGTGGAAATCGCCCTGC
AGTGGAATGACAGCTTCAACGAAAACCTGCAGTGCTTCACCAACAACATTCCGCAGCG

CGATGGCGGCACCCACCTGGTGGGCTTCCGTTCGGCACTGACACGTAACCTGAACAAC
TACATCGAACAGGAAGGTCTGGCGAAGAAGCACAAGGTCGCCACCACCGGTGACGAT
GCCCGCGAAGGCCTGACCGCAATCATTTCGGTCAAGGTGCCGGATCCGAAGTTCAGCT
CCCAGACCAAAGACAAGCTGGTGTCTTCCGAAGTGAAGACCGCGGTCGAACAGGAAA
TGGGCAAGTACTTCTCCGACTTCCTGCTGGAAAACCCGAACGAAGCCAAGCTGGTGGT
CGGCAAGATGCTCGACGCCGCCCGTGCCCGTGAAGCGGCGCGTAAGGCTCGTGAGATG
ACCCGCCGTAAAGGTGCGCTGGATATCGCCGGCCTGCCGGGCAAACTGGCGGACTGCC
AGGAAAAAGACCCTGCCCTTTCCGAACTCTACCTGGTGGAAGGTGACTCTGCTGGCGG
CTCCGCCAAGCAGGGACGCAACCGTAAGACCCAGGCGATTCTGCCGCTCAAGGGCAAG
ATCCTTAACGTCGAGAAAGCGCGTTTCGACAAGATGATTTCCTCGCAAGAGGTCGGCA
CCTTGATCACTGCACTCGGTTGCGGCATCGGCCGCGAAGAGTACAACATCGACAAGCT
GCGTTATCACAACATCATCATCATGACCGACGCCGACGTCGACGGTTCGCACATCCGT
ACCCTGCTGCTGACCTTCTTCTTCCGTCAGCTGCCGGAGCTGATCGAGCGTGGCTACAT
CTACATCGCTCAACCGCCGCTGTACAAGGTCAAGAAAGGCAAGCAAGAGCAATACATC
AAAGACGACGACGCCATGGAAGAGTACATGACGCAGTCGGCCCTGGAAGATGCGAGC
CTGCACCTGAACGAAGACGCACCGGGTATTTCCGGCGAGGCGCTGGAGCGCCTGGTGA
ACGACTTCCGCATGGTCATGAAAACCCTCAAGCGTCTGTCGCGCCTGTACCCTCAGGA
GCTGACCGAGCACTTCATCTACCTGCCGGCCGTGAGCCTGGAGCAGCTCTCCGATCAC
GCGGCGATGCAGGATTGGCTGGCCCAATATGAAGTCCGCCTGCGCACCGTCGAGAAGT
CCGGCCTGGTCTACAAGGCCAGCCTGCGTGAAGACCGTGAACGTAATGTCTGGCTGCC
AGAGGTCGAACTGATCTCCCACGGCCTGTCGAACTACGTCACCTTCAACCGTGACTTCT
TCGGCAGCAATGACTACAAGACCGTCGTCACCCTCGGTGCTCAACTGAGCTCCCTGCTG
GACGAAGGCGCTTATATTCAGCGTGGCGAACGCAAGAAGGCAGTGACCGAGTTCAAG
GAAGCCCTGGACTGGCTGATGACCGAAAGTACCAAGCGCCACACCATCCAGCGATACA
AAGGTCTGGGCGAGATGAACCCGGATCAGCTGTGGGAAACCACCATGGACCCAAGCG
TGCGCCGTATGCTCAAGGTCACCATCGAAGACGCCATCGGCGCCGACCAGATCTTCAA
CACCCTGATGGGTGATGCGGTCGAGCCTCGTCGCGACTTCATCGAAAGCAACGCCCTG
GCGGTATCCAACCTGGACTTCTGAATGTCCGGAAAAGCGCAACAGCAGTCTCGCCTCA
AAGAGTTGATCAGCCGTGGTCGTGAGCAGGGTTACCTGACTTACGCGGAGGTCAACGA
CCACCTGCCGGAGGATATTTCAGATCCGGAACAGGTGGAAGACATCATCCGCATGATC
AACGACATGGGGATCAACGTATTCGAGAGTGCTCCGGATGCGGATGCCCTTTTGTTGG
CCGAAGCCGATACCGACGAAGCAGCAGCTGAAGAAGCCGCCGCAGCGTTGGCGGCTG
TGGAAACCGACATTGGTCGCACTACCGACCCCGTGCGTATGTACATGCGCGAAATGGG
TACGGTAGAGCTGCTCACACGTGAAGGCGAAATCGAAATCGCCAAGCGTATCGAAGA
GGGCATCCGTGAAGTGATGGGCGCGATCGCGCACTTCCCTGGCACGGTTGAGCACATC
CTCTCCGAATACACTCGCGTCACCACCGAAGGTGGCCGCCTGTCCGACGTCCTGAGCG
GTTACATCGACCCGGACGACGGTATTGCGCCGCCTGCCGCCGAAGTACCACCGCCTGT
CGATGCCAAGGCCGCAAAAGCGGACGACGACACCGACGACGATGACGCCGAAGCCAG
TGACGACGAAGAAGAAGCCGAAAGCGGTCCGGATCCGGTCATCGCAGCCCAGCGCTTT
GGCGCCGTTGCCGACCAGATGGAAATTACCCGCAAGGCGCTGAAAAAGCACGGTCGC
GAACACAAGCAAGCCCTGGCTGAAATGCTGGCCCTGGCTGAGCTGTTCATGCCGATCA
AACTGGTTCCGAAGCAATTCGAAGGCCTGGTTGAACGTGTTCGTAGTGCCCTGGATCG
CCTGCGTCAGCAAGAGCGCGCGATCATGCAGCTCTGTGTTCGTGATGCCCGCATGCCA
CGCGCCGACTTCCTGCGCCAGTTCCCTGGCAATGAAGTGGACGAAAGCTGGTCCGACG
CACTGGCCAAAGGCAAGGCCAAGTACGCCGAAGCCATTGGCCGCCTGCAGCCGGACAT
CATCCGTTGCCAGCAGAAGCTGACCGCACTCGAGACCGAGACCGGCCTGACGATCGCC
GAGATCAAGGACATCAACCGTCGCATGTCGATCGGCGAGGCCAAGGCCCGTCGCGCGA
AGAAAGAGATGGTCGAAGCCAACTTGCGTCTGGTGATCTCCATCGCCAAGAAGTACAC
CAACCGTGGCCTGCAATTCCTCGACCTGATCCAGGAAGGCAACATCGGTTTGATGAAA
GCAGTAGACAAGTTCGAATACCGTCGCGGCTACAAGTTCTCGACTTATGCCACCTGGT
GGATCCGTCAGGCGATCACTCGCTCGATCGCCGACCAGGCCCGCACCATCCGTATTCC

GGTGCACATGATCGAGACGATCAACAAGCTCAACCGTATTTCCCGGCAGATGTTGCAG
GAAATGGGTCGCGAACCGACTCCGGAAGAGCTGGGCGAACGCATGGAAATGCCTGAG
GACAAGATCCGCAAGGTATTGAAGATCGCTAAAGAGCCGATCTCCATGGAAACCCCGA
TCGGTGATGACGAAGACTCCCATCTGGGTGACTTCATCGAAGACTCGACCATGCAGTC
GCCAATCGATGTCGCCACCGTTGAGAGCCTTAAAGAAGCGACTCGCGAAGTACTCTCC
GGCCTCACTGCCCGTGAAGCCAAGGTACTGCGCATGCGCTTCGGCATCGACATGAATA
CCGACCACACCCTTGAGGAAGTCGGTAAGCAGTTCGATGTTACCCGTGAGCGGATTCG
TCAGATCGAAGCCAAGGCGCTGCGCAAGCTGCGCCACCCGACGCGAAGCGAGCATCTG
CGCTCCTTCCTCGACGAGTAATTGACTAAGCCAGCCATACTCGCCCTTGCTGATGGCAG
CATTTTTCGCGGCGAAGCCATTGGAGCCGACGGTCAAACCGTTGGTGAGGTGGTGTTT
AACACCGCAATGACCGGCTATCAGGAAATCCTTACCGATCCTTCCTACGCCCAACAGA
TCGTTACCCTGACTTACCCACATATCGGCAATACCGGCACCACGCCGGAAGACGCCGA
GTCCGATCGTGTCTGGTCGGCCGGTCTGGTGATTCGCGACCTGCCACTGGTTGCGAGCA
ACTGGCGTAACACCTTGTCCCTGTCCGACTACCTGAAAGCCAACAATGTTGTGGCGATC
GCCGGTATCGACACCCGTCGTCTGACGCGCATCCTGCGCGAGAAAGGCGCGCAGAACG
GCTGCATCATGGCCGGCGACAATATCTCCGACGAAGCGGCGATTGCCGCTGCGCGCGG
CTTCCCGGGCCTGAAAGGCATGGATCTGGCGAAGGTCGTCAGCACCAAGGAAAGCTAC
GAGTGGCGCTCCAGCGTCTGGAGCCTGAAGACCGACAGTCACCCGACCATCGAGGCTT
CCGAGCTGCCTTACCACGTGGTTGCCTACGACTACGGCGTCAAGCTGAACATCCTGCGC
ATGCTGGTCGAGCGCGGTTGCCGCGTGACCGTGGTACCTGCGCAAACCCCGGCCAGCG
ACGTCCTGGCGCTCAAGCCTGACGGTGTGTTCCTGTCCAACGGTCCTGGCGACCCCGAG
CCTTGCGATTACGCCATCCAGGCGATCAAGGACGTGCTGGAAACCGAGATTCCGGTCT
TCGGTATCTGCCTGGGCCACCAACTGCTGGCGCTGGCCGCCGGCGCCAAGACAGTGAA
GATGGGCCACGGCCACCACGGTGCCAACCACCCGGTCCAGGACCTGGACAGCGGTGTA
GTGATGATCACCAGCCAGAACCACGGTTTTGCGGTGGACGAAACCACCCTGCCGGGCA
ACGTGCGGGCGATCCACAAGTCGCTGTTCGACGGCACCCTGCAAGGCATCGAGTTGAC
CGACAAGAGCGCATTCAGCTTCCAGGGCCACCCTGAAGCGAGCCCGGGCCCGAACGAT
GTGGCGCCGCTGTTCGATCGTTTCATCAACGAGATGGCCAAGCGACGCTGAATGAGTA
GCGGACGTATCGTTCAAATCATCGGCGCCGTTATCGACGTGGAATTTCCACGCGACAG
CGTACCGAGCATCTACGACGCCTTGAAGGTTCAAGGCGCCGAAACCACTCTGGAAGTT
CAGCAGCAGCTGGGCGACGGCGTGGTACGTACCATTGCGATGGGCTCCACCGAGGGCT
TGAAGCGCGGTCTGGACGTCAACAACACTGGCGCAGCCATCTCCGTACCGGTCGGTAA
AGCGACCCTGGGCCGGATCATGGACGTACTGGGCAACCCGATCGACGAAGCTGGCCCG
ATCGGCGAAGAAGAGCGTTGGGGCATTCACCGTCCTGCGCCGACCTTCGCTGAACAAG
CTGGCGGCAACGACCTGCTGGAAACCGGCATCAAGGTTATCGACCTGGTTTGCCCGTT
CGCCAAGGGCGGTAAAGTCGGTCTGTTCGGTGGTGCCGGTGTGGGCAAAACCGTAAAC
ATGATGGAACTGATCCGTAACATCGCCATCGAGCACAGCGGTTATTCCGTGTTCGCCG
GTGTGGGTGAGCGTACTCGTGAGGGTAACGACTTCTACCACGAGATGAAGGATTCCAA
CGTTCTGGACAAAGTGGCACTGGTATACGGCCAGATGAACGAGCCGCCGGGAAACCGT
CTGCGCGTAGCTCTGACCGGCCTGACCATGGCCGAGAAGTTCCGTGACGAAGGTAACG
ACGTTCTGCTGTTCGTCGACAACATCTATCGTTACACCCTGGCCGGTACCGAAGTATCC
GCACTGCTGGGCCGTATGCCTTCGGCAGTAGGTTACCAGCCGACCCTGGCTGAAGAGA
TGGGCGTTCTGCAAGAACGTATCACTTCGACCAAGCAAGGCTCGATCACCTCGATCCA
AGCGGTATACGTGCCTGCGGACGACTTGACCGACCCGTCGCCAGCGACCACCTTCGCC
CACTTGGACGCCACCGTCGTTCTGTCCCGTGACATCGCTTCCCTGGGTATCTACCCAGC
GGTAGACCCACTGGACTCGACTTCCCGTCAGCTGGACCCGAACGTGATCGGCAACGAG
CACTACGAAACCGCTCGCGGCGTTCAGTACGTGCTGCAGCGCTACAAAGAGCTGAAGG
ACATCATTGCGATCCTGGGTATGGACGAACTGTCCGAAGCCGACAAGCAACTGGTATC
CCGCGCTCGTAAGATCCAGCGCTTCCTGTCGCAGCCGTTCTTCGTGGCTGAAGTCTTCA
CTGGTTCTCCAGGCAAATACGTTTCCCTGAAAGACACCATCGCTGGCTTCAAAGGCATC
CTCAACGGTGACTACGACCATCTGCCAGAACAAGCGTTCTACATGGTTGGTGGCATCG

AAGAAGCGATCGAGAAAGCCAAGAAACTGTAA

NCBI Reference Sequence: NZ_LT799039.1
Strain: KT2440
Chained genes: 16S rRNA, recA, gyrB, rpoD, carA, atpD
>NZ_LT799039_KT2440
GAACTGAAGAGTTTGATCATGGCTCAGATTGAACGCTGGCGGCAGGCCTAACACATGC
AAGTCGAGCGGATGACGGGAGCTTGCTCCTTGATTCAGCGGCGGACGGGTGAGTAATG
CCTAGGAATCTGCCTGGTAGTGGGGGACAACGTTTCGAAAGGAACGCTAATACCGCAT
ACGTCCTACGGGAGAAAGCAGGGGACCTTCGGGCCTTGCGCTATCAGATGAGCCTAGG
TCGGATTAGCTAGTTGGTGGGGTAATGGCTCACCAAGGCGACGATCCGTAACTGGTCT
GAGAGGATGATCAGTCACACTGGAACTGAGACACGGTCCAGACTCCTACGGGAGGCA
GCAGTGGGGAATATTGGACAATGGGCGAAAGCCTGATCCAGCCATGCCGCGTGTGTGA
AGAAGGTCTTCGGATTGTAAAGCACTTTAAGTTGGGAGGAAGGGCAGTAAGTTAATAC
CTTGCTGTTTTGACGTTACCGACAGAATAAGCACCGGCTAACTCTGTGCCAGCAGCCGC
GGTAATACAGAGGGTGCAAGCGTTAATCGGAATTACTGGGCGTAAAGCGCGCGTAGGT
GGTTTGTTAAGTTGGATGTGAAAGCCCCGGGCTCAACCTGGGAACTGCATCCAAAACT
GGCAAGCTAGAGTACGGTAGAGGGTGGTGGAATTTCCTGTGTAGCGGTGAAATGCGTA
GATATAGGAAGGAACACCAGTGGCGAAGGCGACCACCTGGACTGATACTGACACTGA
GGTGCGAAAGCGTGGGGAGCAAACAGGATTAGATACCCTGGTAGTCCACGCCGTAAA
CGATGTCAACTAGCCGTTGGAATCCTTGAGATTTTAGTGGCGCAGCTAACGCATTAAGT
TGACCGCCTGGGGAGTACGGCCGCAAGGTTAAAACTCAAATGAATTGACGGGGGCCCG
CACAAGCGGTGGAGCATGTGGTTTAATTCGAAGCAACGCGAAGAACCTTACCAGGCCT
TGACATGCAGAGAACTTTCCAGAGATGGATTGGTGCCTTCGGGAACTCTGACACAGGT
GCTGCATGGCTGTCGTCAGCTCGTGTCGTGAGATGTTGGGTTAAGTCCCGTAACGAGCG
CAACCCTTGTCCTTAGTTACCAGCACGTAATGGTGGGCACTCTAAGGAGACTGCCGGT
GACAAACCGGAGGAAGGTGGGGATGACGTCAAGTCATCATGGCCCTTACGGCCTGGGC
TACACACGTGCTACAATGGTCGGTACAGAGGGTTGCCAAGCCGCGAGGTGGAGCTAAT
CTCACAAAACCGATCGTAGTCCGGATCGCAGTCTGCAACTCGACTGCGTGAAGTCGGA
ATCGCTAGTAATCGCGAATCAGAATGTCGCGGTGAATACGTTCCCGGGCCTTGTACAC
ACCGCCCGTCACACCATGGGAGTGGGTTGCACCAGAAGTAGCTAGTCTAACCTTCGGG
GGGACGGTTACCACGGTGTGATTCATGACTGGGGTGAAGTCGTAACAAGGTAGCCGTA
GGGGAACCTGCGGCTGGATCACCTCCTTAAATGGACGACAACAAGAAGCGCGCCTTGG
CTGCGGCCCTGGGTCAGATCGAACGCCAATTCGGTAAAGGCGCGGTCATGCGCATGGG
TGACCATGAGCGTCAAGGCATTCCGGCCATCTCCACCGGCTCGCTGGGGCTGGATATC
GCCCTGGGCATCGGCGGTCTGCCAAAAGGCCGTATCGTCGAGATCTACGGCCCGGAAT
CGTCGGGTAAGACCACGCTGACCCTGTCTGTCATCGCTGAAGCGCAAAAGAACGGTGC
TACCTGCGCCTTCGTCGACGCCGAACATGCCCTTGACCCTGAGTACGCCGGCAAGCTG
GGCGTCAACGTCGATGACCTGCTGGTTTCGCAGCCGGACACCGGTGAGCAGGCCCTTG
AAATCACCGACATGCTGGTGCGTTCCAACGCGGTCGACGTGATCATTGTCGACTCCGTT
GCCGCGCTGGTACCGAAGGCCGAGATCGAAGGCGAGATGGGTGACATGCATGTGGGC
CTGCAGGCCCGCCTGATGTCCCAGGCACTGCGTAAGATCACCGGTAACATCAAGAACG
CGAACTGCCTGGTCATCTTCATCAACCAGATCCGTATGAAGATCGGTGTGATGTTCGGC
AGCCCGGAAACCACTACCGGTGGTAACGCCCTGAAGTTCTATGCTTCGGTCCGTCTGG
ACATCCGCCGTACCGGCGCGGTCAAGGAAGGCGACGAAGTGGTCGGCAGCGAAACCC
GCGTCAAGATCGTCAAGAACAAGGTCTCGCCTCCGTTCCGTCAGGCTGAGTTCCAGATT
CTTTACGGGAAAGGTATCTACCGTAACGGCGAGATCATTGATCTGGGGGTATCTCAGG
GCCTGGTCGAAAAGTCCGGCGCCTGGTACGCCTACCAAGGCAACAAGATCGGTCAAGG
CAAAGCCAACGCTGCCAAGTACCTGGCTGAGAACCCGGCTATTGGTGCCGAGATCGAG
AAGCAGATTCGTGAGAAGTTGCTGAAAGCGGGTGCTGCTGCTGAAGCCGGCAAAGCTG
CTGCTGCTGAAGCTGATGCCGATGACATGGCCGACGCTGACGCCGGTTATTGAATGAG

CGAAAATCAAACGTACGACTCCTCCAGCATCAAGGTGCTGAAAGGTTTGGATGCCGTA
CGCAAGCGTCCCGGCATGTACATTGGCGACACCGATGATGGTAGTGGCCTGCACCACA
TGGTCTTCGAGGTGGTCGACAACTCGATCGACGAAGCCCTCGCCGGTCACTGCGATGA
CATTACCGTCATCATCCACCCGGACGAATCTATCAGTGTGCGCGACAACGGTCGCGGC
ATTCCGGTCGATGTGCATAAGGAAGAAGGCGTTTCCGCAGCCGAGGTCATCATGACTG
TGCTGCACGCCGGCGGTAAGTTTGACGACAACTCCTACAAAGTATCCGGCGGTCTGCA
CGGTGTAGGTGTGTCGGTTGTGAACGCCCTGTCCGAGAAGCTGGTTTTGACTGTTCGCC
GTAGCGGCAAGATCTGGGAACAGACTTACGTTCACGGTGTTCCACAAGCGCCCATGGC
GGTTGTCGGTGACAGTGAAACCACGGGTACCCACATCCACTTCAAGCCATCGGCTGAA
ACCTTCAAGAACATTCACTTCAGCTGGGACATCCTGGCCAAGCGCATCCGCGAGCTGT
CGTTCCTCAACTCGGGCGTTGGCATTCTGCTGAAGGATGAGCGCAGCGGTAAGGAAGA
GTTCTTCAAGTACGAAGGCGGTCTGCGTGCGTTCGTCGAGTACTTGAACACCAACAAG
ACGCCGGTCAACTCCCAGGTGTTCCACTTCAACGTTCAGCGTGACGATGGCGTGGGTGT
TGAAGTCGCCCTGCAATGGAACGACAGCTTCAACGAAAACCTGCTGTGCTTTACCAAC
AATATTCCGCAGCGTGATGGCGGTACCCACCTGGTGGGTTTCCGTTCCTCGCTGACCCG
TAGCCTTAACAGCTACATCGAGCAGGAAGGCCTGGCCAAGAAGAACAAGGTGGCAAC
CACTGGCGACGACGCCCGTGAAGGCCTGACCGCGATCATCTCGGTGAAGGTACCGGAC
CCGAAGTTCAGCTCGCAGACCAAGGACAAGCTGGTCTCCTCGGAGGTGAAAACCGCCG
TGGAACAGGAGATGAACAAGTACTTCGCCGATTTCCTCCTGGAAAACCCGAACGAGGC
GAAGGCCGTCGTTGGCAAGATGATCGACGCCGCTCGCGCCCGTGAAGCCGCCCGTAAA
GCCCGTGAGATGACCCGCCGTAAAGGTGCGCTGGATATCGCGGGTCTGCCGGGCAAGC
TGGCCGACTGCCAAGAGAAGGATCCTGCTCTCTCCGAACTGTACCTGGTGGAGGGTGA
CTCCGCGGGTGGCTCGGCCAAGCAAGGCCGCAACCGTCGTACCCAGGCGATCTTGCCG
CTGAAGGGTAAAATCCTCAACGTCGAGAAAGCGCGCTTCGACAAGATGATTTCGTCCC
AGGAAGTGGGCACGCTGATCACTGCGCTGGGCTGTGGCATCGGCCGCGAAGAGTACAA
CATCGACAAACTGCGTTATCACAACATCATCATCATGACCGATGCTGACGTTGACGGTT
CGCACATCCGTACGCTGCTGCTGACCTTCTTCTTCCGTCAGCTGCCGGAGCTGGTCGAG
CGTGGCTACATCTATATTGCCCAGCCGCCGCTGTACAAGGTGAAACGAGGCAAGCAGG
AGCAGTACATCAAGGACGATGAGGCCATGGAAGAGTACATGACCCAGTCGGCTCTGG
AAGATGCCAGCCTGCACCTGGACGAATCGGCGCCAGCAGTTTCCGGCGTGCAGCTGGA
AGCGCTGGTGAATGAGTTCCGTAGTGTCATGAAGACTCTCAAGCGCCTGTCGCGCTTGT
ACCCGGAAGAGCTGACCGAGCACTTCGTCTACCTGCCTGAGGTGACCCTGGAGCAGTT
GGGTGACCACGCAGTGATGCAGGCCTGGCTGGCCCAGTTCCAGGCGCGTCTGAACTCC
AGCCAGAAGTCTGGCCTGGCTTACAACGCCAGCCTGCGTGAAGACAAAGAGCGCAAC
GTATGGCTGCCTGAAGTGGAAATTACCTCTCACGGTCTGGCCAGCTACATCACCTTCAA
CCGCGATTTCTTCGGCAGCAATGACTACCGTACCGTAGTCAACATTGGTGCCAAGCTTT
CGAGCCTGTTGGGTGAAGGTGCGTACGTGCAGCGCGGTGAACGCCGCAAGGCAATCGT
CGAGTTCAAAGAGGGCCTGGATTGGCTGATGAACGAGACCACCAAGCGCCATACGATT
CAGCGATACAAAGGGCTGGGTGAGATGAACCCGGATCAACTGTGGGAAACCACCATG
GACCCGACCGTTCGCCGTATGCTCAAGGTCACGATCGAAGATGCTATCGCCGCTGACC
AGATCTTCAACACCCTGATGGGTGATGCGGTCGAGCCGCGTCGTGACTTCATCGAAAG
TAACGCGCTGTCGGTGTCGAACCTGGACTTCTGAATGTCCGGAAAAGCGCAACAGCAG
TCTCGTATCAAAGAGTTGATCACCCGCGGTCGTGAGCAGGGCTACCTGACTTACGCGG
AGGTCAACGACCACCTGCCTGAGGATATTTCAGATCCGGAACAGGTGGAAGACATCAT
CCGCATGATCAACGACATGGGGATCAACGTATTCGAGAGTGCTCCGGATGCGGATGCC
CTTCTGTTGGCGGAAGCCGACACCGATGAAGCCGCGGCCGAAGAAGCCGCTGCTGCAT
TGGCGGCAGTTGAAACCGATATCGGCCGCACGACCGACCCGGTGCGCATGTATATGCG
CGAAATGGGTACTGTCGAGCTGCTGACCCGCGAAGGCGAGATCGAAATCGCCAAGCGT
ATCGAGGAAGGCATTCGTGAAGTCATGGGCGCCATCGCCCACTTCCCGGGCACTGTCG
ACTACATTCTCGGCGAATATGACCGCGTCACCACCGAGGGTGGCCGCCTGTCGGACGT
TCTCAGCGGTTACATCGACCCTGACGACAACATTGCCGCGCCAACCGAAGAAGTGCCG

ATCCCAGGTGCCAAGGCCGCTGCGGCGAAGGAAGAGTCCGACGACGACGAAGAAGAA
TCCGAAAGCGGTGACGACGAGGAAGAGGCCGAGAGCGGCCCGGATCCGGTCGTCGCA
GCCCAACGCTTCGGTGCGGTATCCGATCAGCTTCAGGCAACCTCCAAGGTCCTGAAGA
AAAATGGTCGCAACCACAAGGAAAGCATCGAGGCCCTGCAGGCCCTGGCTGACCTGTT
CATGCCGATCAAGCTGGTACCGAAGCAGTTCGAGGTACTGGTCGAGCGTGTCCGTGAC
GCCCTGAACCGTCTGCGACAGCAAGAACGCGCCATCATGCAGCTGTGCGTACGTGACG
CCCGCATGCCGCGAGCCGACTTCCTGCGCATGTTCCCAAGCAACGAAACCGACCAGAC
CTGGAGCGGTGACCTGGCCAAGCGCAACACCAAGTGGGCTGCCGCCCTGGGTGAAAA
GAACGCTGCCATCGTCGCTTGCCAACAGAAGCTGATCGACCTTGAGACCGAAACCGGC
CTGACCGTTGCCGAGATCAAGGAAATCAACCGTCGCATGTCGATCGGTGAAGCCAAGG
CCCGCCGCGCCAAGAAAGAAATGGTCGAGGCGAACCTGCGTCTGGTGATTTCCATCGC
CAAGAAGTACACCAACCGTGGCCTGCAGTTCCTCGACCTGATCCAGGAAGGCAACATC
GGTCTGATGAAAGCGGTGGACAAGTTCGAATACCGTCGCGGCTACAAGTTCTCGACCT
ATGCCACCTGGTGGATCCGTCAGGCGATCACCCGTTCGATCGCCGACCAGGCACGCAC
CATCCGCATTCCGGTGCACATGATCGAGACGATCAACAAGCTCAACCGTATTTCCCGCC
AGATGCTGCAGGAAATGGGTCGCGAACCGACTCCGGAAGAGCTGGGCGAGCGCATGG
AAATGCCTGAGGACAAAATCCGCAAGGTATTGAAGATCGCCAAAGAGCCGATCTCCAT
GGAAACCCCGATCGGTGACGACGAAGATTCGCACCTGGGCGACTTCATCGAGGACTCG
ACCATGCAGTCCCCGATCGACGTGGCCACGGTCGAAAGCCTCAAGGAAGCCACCCGTG
ACGTGCTCTCGGGCCTGACCGCACGTGAAGCCAAGGTGCTGCGCATGCGTTTCGGTAT
CGACATGAACACCGACCACACCCTCGAAGAGGTGGGCAAGCAGTTTGACGTAACGCGT
GAGCGGATCCGTCAGATCGAAGCGAAGGCGTTGCGCAAGCTGCGCCACCCGACTCGCA
GCGAGCACCTGCGCTCCTTCCTCGACGAGTGATTGACAAAGCCAGCCATACTCGCCCTT
GCCGACGGCAGTATTTTCCGCGGTGAAGCCATCGGTGCCGACGGTCAGACCGTTGGTG
AGGTGGTATTCAACACCGCTATGACCGGCTACCAGGAAATCCTTACAGACCCTTCCTAC
GCGCAGCAAATCGTTACCCTGACCTACCCGCACATCGGCAACACCGGTACTACCCCGG
AAGACGCCGAGTCGAGCCGCGTCTGGTCCGCTGGCCTGGTCATCCGTGACCTGCCGCT
GCTGGCCAGCAACTGGCGTAACACCCAGTCGCTGCCTGAGTACCTCAAGGCCAACAAC
GTCGTCGCCATCGCCGGCATCGACACCCGTCGCCTGACCCGTATCCTGCGTGAAAAGG
GCGCCCAGAACGGCTGCATTCTGGCGGGTGACAACATCAGCGAAGAAGCTGCCATCGC
TGCTGCCCGCGGCTTCCCGGGCCTGAAGGGCATGGACCTGGCCAAGGTCGTCTCCACC
AAGGAACGTTACGAGTGGCGCTCCAGCGTGTGGGAGCTGAAAACCGACAGCCACCCG
ACCATCGACGCTGCCGACCTGCCGTACCACGTGGTTGCCTTCGACTATGGCGTCAAGCT
GAACATCCTGCGCATGCTGGTGGCCCGCGGCTGCCGCGTGACCGTGGTACCAGCCCAG
ACCCCGGCCAGCGAAGTACTGGCACTCAACCCGGACGGCGTGTTCCTGTCCAACGGCC
CTGGTGACCCTGAGCCGTGCGACTACGCGATCCAGGCGATCAAGGAAATCCTCGAAAC
CGAGATCCCGGTATTCGGCATCTGCCTCGGCCACCAGCTGCTGGCCCTGGCGTCCGGCG
CCAAGACCGTGAAAATGGGCCACGGCCACCACGGTGCCAACCACCCGGTCCAGGACCT
GGATACTGGTGTGGTCATGATCACCAGCCAGAACCACGGTTTCGCCGTTGACGAGGCG
ACCCTGCCGGGCAACGTTCGCGCCATTCACAAGTCGCTGTTCGACGGCACCCTGCAGG
GTATCGAGCGTACCGACAAGAGCGCGTTCAGCTTCCAGGGCCACCCTGAAGCGAGCCC
GGGCCCGACCGACGTCGCGCCTCTGTTCGATCGTTTCACCGATGCCATGGCCAAGCGCC
GCTGAATGAGTAGCGGACGTATCGTTCAAATCATCGGCGCCGTCATCGACGTGGAATT
CCCACGTGACGTCGTGCCGAGTGTATACAACGCGCTTAAAGTACAAGGCGCGGAAACC
ACCCTGGAAGTTCAGCAGCAGCTGGGCGACGGCGTGGTTCGTACCATTGCGATGGGCT
CGACCGAAGGCCTGAAGCGCGGTCTGGATGTCGTCGACACCGGCGCTGCCATTTCCGT
TCCAGTTGGTAAGGCCACCCTGGGCCGTATCATGGACGTACTGGGCAACCCGATCGAC
GAAGCCGGCCCGATCGGCGAAGAAGAGCGTCGCGGTATCCACCAGCCAGCGCCTTCGT
TCGCTGACCAGGCAGGCGGCAACGACCTGCTGGAAACCGGCATCAAGGTTATCGACCT
GGTTTGCCCGTTCGCCAAGGGTGGTAAGGTTGGTCTGTTCGGTGGTGCCGGTGTCGGCA
AGACCGTAAACATGATGGAACTGATCCGTAACATCGCCATGGAACACAGCGGTTACTC

CGTGTTCGCTGGTGTGGGTGAGCGTACTCGTGAGGGTAACGACTTCTACCACGAGATG
AAGGACTCCAACGTTCTCGACAAAGTAGCGCTGGTCTACGGTCAGATGAACGAGCCAC
CAGGAAACCGTCTGCGTGTAGCGCTGACCGGCCTGACCATGGCCGAGAAGTTCCGTGA
CGAAGGTAACGACGTTCTGCTGTTCGTCGACAACATCTATCGTTACACCCTGGCCGGTA
CCGAAGTATCCGCACTGCTGGGCCGTATGCCTTCGGCAGTAGGTTACCAGCCGACCCT
GGCTGAAGAGATGGGCGTTCTGCAAGAGCGCATCACCTCCACCAAGGAAGGTTCGATC
ACCTCCGTACAGGCCGTATACGTACCTGCGGACGACTTGACCGACCCGTCGCCAGCGA
CCACCTTCGCCCACTTGGACGCCACCGTCGTTCTGTCCCGTGACATCGCCTCCCTGGGT
ATCTACCCAGCGGTCGACCCACTGGACTCGACTTCGCGCCAGCTGGACCCGAACGTGA
TCGGCAACGAGCACTACGAGACCGCTCGTGGCGTTCAGTATGTTCTGCAGCGCTATAA
AGAGCTGAAGGACATCATTGCGATCCTGGGTATGGACGAACTGTCCGAAGCCGACAAG
CAACTGGTAGCCCGCGCTCGTAAGATCCAGCGCTTCCTGTCGCAGCCGTTCTTCGTGGC
AGAAGTCTTCACCGGTTCGCCAGGCAAGTACGTTTCCCTGAAAGACACCATCGCTGGC
TTCAGCGGCATCCTCAAAGGTGACTACGACCACCTGCCAGAACAAGCGTTCTACATGG
TCGGCAGCATCGACGAAGCGATCGAGAAAGCCAAGAAACTGTAA

Reference

1. Love, M.I.; Huber, W.; Anders, S. Moderated estimation of fold change and dispersion for RNA-seq data with DESeq2. *Genome biology* **2014**, *15*, 1-21.
2. Weisburg, W.G.; Barns, S.M.; Pelletier, D.A.; Lane, D.J. 16S ribosomal DNA amplification for phylogenetic study. *Journal of bacteriology* **1991**, *173*, 697-703.
3. Vargas, L.; de Carvalho, T.L.G.; Ferreira, P.C.G.; Baldani, V.L.D.; Baldani, J.I.; Hemerly, A.S. Early responses of rice (*Oryza sativa L.)* seedlings to inoculation with beneficial diazotrophic bacteria are dependent on plant and bacterial genotypes. *Plant and soil* **2012**, *356*, 127-137.
4. Song, Y.; Wang, L.; Xiong, L. Comprehensive expression profiling analysis of *OsIAA* gene family in developmental processes and in response to phytohormone and stress treatments. *Planta* **2009**, *229*, 577-591.
5. Lee, H.; Hong, J.; Kim, S. First report of leaf blight caused by *Pantoea agglomerans* on rice in Korea. *Plant Disease* **2010**, *94*, 1372-1372.
6. Brusamarello-Santos, L.; Pacheco, F.; Aljanabi, S.; Monteiro, R.; Cruz, L.; Baura, V.; Pedrosa, F.; Souza, E.; Wassem, R. Differential gene expression of rice roots inoculated with the diazotroph *Herbaspirillum seropedicae*. *Plant and Soil* **2012**, *356*, 113-125.
